# Glutamine Starvation Induces Ferroptosis in NSCLC via AMPK/PDZD8-Mediated Ferritinophagy

**DOI:** 10.3390/nu18101596

**Published:** 2026-05-18

**Authors:** Hong Chen, Xiaoying Wu, Manting Zhu, Ying Cheng, Qing Feng

**Affiliations:** 1 Department of Nutrition and Food Hygiene, School of Public Health, Key Laboratory of Public Health Safety and Emergency Prevention and Control Technology of Higher Education Institutions in Jiangsu Province, Nanjing Medical University, Nanjing 211166, China; chenhong_00@126.com (H.C.); 19823944059@163.com (X.W.); 15062237050@163.com (M.Z.); yingcheng1013@163.com (Y.C.); 2 Key Laboratory of Toxicology, Department of Nutrition and Food Hygiene, School of Public Health, Nanjing Medical University, Nanjing 211166, China

**Keywords:** glutamine starvation, ferritinophagy, ferroptosis, NSCLC, AMPK, PDZD8

## Abstract

**Objectives**: The dependence of non-small cell lung cancer (NSCLC) on glutamine has made targeting glutamine metabolism an attractive therapeutic approach. Dietary interventions are increasingly considered as adjuvant cancer therapies. This study aims to explore the relationship between glutamine starvation and ferroptosis in NSCLC and to elucidate the underlying molecular mechanisms. **Methods**: The effects of glutamine starvation were evaluated both in A549 and H460 NSCLC cell lines and in vivo using xenograft models in SCID mice. Assessments included cell viability, migration, clonogenic capacity, and the expression of key proteins. To gain mechanistic insight, AMPK was either overexpressed or inhibited, and key markers of ferritinophagy (including ULK1, BECN1, NCOA4, and LC3-II/I) and ferroptosis (such as ACSL4, GPX4, and xCT) were analyzed. **Results**: Glutamine starvation markedly suppressed tumor growth in both in vitro and in vivo settings, while also reducing cell migration and clonogenicity in cultured cells. This intervention activated AMPK, as indicated by increases in both total and phosphorylated forms, and upregulated PDZD8 expression. Mechanistically, AMPK activation played a critical role in driving ferritinophagy and ferroptosis—manipulation of AMPK consistently altered key markers of these processes. Furthermore, AMPK levels influenced PDZD8 protein expression. Notably, overexpressing PDZD8 alone was sufficient in promoting both ferritinophagy and ferroptosis, indicating that PDZD8 acts as a critical downstream mediator of AMPK in this pathway. **Conclusions**: Our findings reveal that glutamine starvation triggers ferroptosis in NSCLC via activation of ferritinophagy, mediated by the AMPK/PDZD8 signaling pathway. These results support the potential of dietary glutamine restriction as a novel therapeutic approach for NSCLC.

## 1. Introduction

Recent cancer statistics indicate that lung cancer exhibits the highest incidence and mortality rates among all malignancies [1], with non-small cell lung cancer (NSCLC) accounting for over 80% of cases [2]. Despite significant improvements in the survival outcomes of some patients through targeted therapy and immunotherapy, the majority still face clinical challenges, including chemotherapy resistance, limited eligibility for targeted agents, and suboptimal response rates to immunotherapy. Consequently, novel therapeutic strategies for NSCLC are urgently needed. In recent years, nutritional therapeutic approaches targeting tumor metabolic reprogramming have garnered considerable scientific interest as a potential new strategy [3].

Glutamine (Gln), a conditionally essential amino acid, serves as a pivotal substrate in tumor metabolic reprogramming [4]. Research indicates that NSCLC cells exhibit glutamine addiction, characterized by upregulated Gln uptake and metabolic pathways to fulfill the heightened bioenergetic and biosynthetic demands of rapid proliferation [5]. This dependency is facilitated by the overexpression of the high-affinity glutamine transporter SLC1A5 in lung cancer and other malignancies [6]. Furthermore, elevated Gln levels are observed in lung tumor tissues compared to gastric and colorectal cancers, particularly in NSCLC [7]. Notably, the oncogenic KRAS mutation, prevalent and aggressive in NSCLC, drives metabolic reprogramming by enhancing glycolysis as well as the uptake of glucose, Gln, and fatty acids [8].

Ferroptosis, an iron-dependent form of regulated cell death driven by lipid peroxidation, is characterized by signature molecular events including glutathione (GSH) depletion, inactivation of glutathione peroxidase 4 (GPX4), and uncontrolled accumulation of lipid reactive oxygen species (ROS) [9]. Within the tumor microenvironment, a complex regulatory network exists between Gln metabolism and ferroptosis, exerting a dual role in modulating tumor progression and therapeutic resistance. Gln starvation, a key therapeutic intervention targeting this axis, regulates ferroptosis through dual mechanisms: it disrupts metabolic homeostasis by depriving cells of carbon and nitrogen sources, while simultaneously exacerbating oxidative stress [10]. Conversely, inhibition of glutaminolysis or glutamine uptake was enough to rescue glutathione peroxidase 4 blocker (RSL3)-treated melanoma cell lines from autophagy-dependent ferroptosis [11].

At the metabolic support level, Gln sustains mitochondrial functional homeostasis via the tricarboxylic acid (TCA) cycle. Critically, functional mitochondria are a prerequisite for ferroptosis induction by cystine deprivation [10]. At the redox regulation level, Gln metabolism supplies a crucial precursor for GSH synthesis, thereby sustaining the antioxidant function of GPX4 [3].

Autophagy, an evolutionarily conserved cellular process, involves the formation of a double-membrane structure that engulfs cytoplasmic components, organelles, and proteins destined for degradation. This structure, the autophagosome, fuses with the lysosome to form an autolysosome, where the encapsulated cargo is degraded [12]. Autophagy regulation is critically implicated in the development, progression, and metastatic potential of NSCLC. Under nutrient deprivation, AMP-activated protein kinase (AMPK) inhibits mTORC1, leading to ULK1 activation and subsequent initiation of autophagosome formation [13]. Autophagy is consistently recorded in response to glutamine starvation in several cancers [14,15]. Mounting evidence indicates molecular crosstalk between autophagy and ferroptosis, demonstrating that autophagy contributes to the regulation of iron-dependent lipid peroxidation and reactive oxygen species (ROS) accumulation during ferroptosis [16]. ROS directly boosts autophagy through the effect on P62 and Atg4 [17].

A key mechanism linking these processes is ferritinophagy—the selective autophagic degradation of the iron storage protein ferritin. Nuclear receptor coactivator 4 (NCOA4), enriched within autophagosomes and interacting with ATG8 family proteins (cargo receptors), is identified as the selective cargo receptor for ferritin. NCOA4 mediates ferritin delivery to the lysosome; consequently, NCOA4 deficiency impairs ferritin degradation, reducing intracellular iron bioavailability [18]. This NCOA4-dependent ferritinophagy promotes ferroptosis by releasing labile iron from ferritin [19]. Studies have shown that autophagy-deficient cells or those treated with the autophagic inhibitor such as chloroquine increased extracellular glutamine uptake via glutamine transporters to compensate for the absence of autophagy.

AMPK, a central regulator of cellular energy metabolism that coordinates metabolic pathways by sensing AMP/ATP ratios, exhibits a significant negative correlation with ferroptosis susceptibility. Mechanistically, ATM governs the intracellular labile iron pool by phosphorylating NCOA4, thereby promoting the NCOA4–ferritin interaction and sustaining ferritinophagy flux—a selective form of macroautophagy that specifically degrades ferritin for iron recycling [20]. Furthermore, AMPK activation promotes ferroptosis via phosphorylation of the core autophagy component BECN1/Beclin1. This phosphorylation enhances the interaction of BECN1 with the cystine/glutamate antiporter system Xc^−^, leading to its inhibition, reduced cystine uptake, and consequent suppression of glutathione (GSH) biosynthesis [21]. Collectively, this AMPK-mediated cascade drives ferroptotic cell death.

Mitochondria-associated endoplasmic reticulum membranes (MAMs) are specialized membrane domains formed by physical connections between mitochondria and the endoplasmic reticulum (ER), serving as critical hubs for inter-organellar communication. Research indicates that MAMs harbor numerous tethering and functional proteins. Among these, PDZD8 acts as an ER-mitochondria anchor protein, facilitating calcium ion transfer and regulating lipid metabolism [22]. Furthermore, emerging evidence implicates PDZD8, a key regulatory protein at MAM contact sites, in ferroptosis. Specifically, PDZD8 deficiency disrupts mitochondrial membrane lipid composition, potentiating lipid peroxidation and exacerbating ferroptosis susceptibility [23]. Recent investigations employing a series of experiments in mouse embryonic fibroblasts and murine models—including metabolic flux tracing with [U-^13^C]-glutamine and [U-^13^C]-palmitate, alongside knockout and mutagenesis of AMPK and PDZD8—revealed that under low glucose conditions, glutaminolysis is activated prior to fatty acid oxidation in an AMPK-dependent manner. PDZD8 was identified as a novel substrate of AMPK, with phosphorylation at threonine 527 (T527) being essential for glutaminolysis activation. It was further demonstrated that phosphorylated PDZD8 interacts with and enhances the activity of glutaminase 1 (GLS1), thereby promoting glutaminolysis. This regulatory axis was also shown to play a critical role in murine skeletal muscle and macrophages [24].

This study investigates the inhibitory effects of dietary glutamine starvation on NSCLC and determines whether its efficacy involves the activation of ferritinophagy/ferroptosis pathways. Furthermore, we elucidate the potential regulatory roles of AMPK and PDZD8 as key mediators in this process. Our findings aim to provide a critical scientific foundation for developing nutritional interventions for cancer patients.

## 2. Materials and Methods

### 2.1. Cell Culture and Reagents

A549 and NCI-H460 cell lines were acquired from the Chinese Academy of Sciences Committee on Type Culture Collection Cell Bank (Shanghai, China). Both cell types were maintained in RPMI-1640 medium (Procell Life Science & Technology Co., Ltd., Wuhan, China), supplemented with 10% heat-inactivated fetal bovine serum (Gibco, Grand Island, NY, USA), 100 U/mL of penicillin, and 100 µg/mL of streptomycin (Beyotime Biotechnology Co., Ltd., Shanghai, China),. Cells were cultured at 37 °C in a humidified atmosphere containing 5% CO_2_. To prepare media with varying glutamine concentrations, L-glutamine powder (purity ≥ 98.5%; SOLARBIO Science & Technology Co., Ltd., Beijing, China) was supplemented into glutamine-free RPMI-1640 medium (Procell Life Science & Technology Co., Ltd., Wuhan, China).

### 2.2. Western Blot Analysis

Proteins were extracted from cell samples via lysis in RIPA buffer (Beyotime Biotechnology Co., Ltd., Shanghai, China), containing PMSF (YI FEI XUE Biotechnology Co., Ltd., Nanjing, China) as a protease and phosphatase inhibitor. Protein concentration was determined using a BCA assay kit (Beyotime Biotechnology Co., Ltd., Shanghai, China). The proteins were resolved on 8% SDS-PAGE gels (Invitrogen, Life Technologies Corp, Carlsbad, CA, USA) and electrophoretically transferred to nitrocellulose membranes (GE Healthcare UK Ltd., Bucks, UK). Following blocking with 5% non-fat milk for 1 h, the membranes were incubated overnight at 4 °C with primary antibodies. Subsequently, the membranes were probed with horseradish peroxidase (HRP)-conjugated secondary antibodies for 1 h at room temperature. The primary antibodies used were anti-AMPKα1, anti-p-AMPKα1, anti-ULK1, anti-BECN1, anti-LC3B, anti-NCOA4, anti-ACSL4, anti-GPX4, anti-xCT, anti-PDZD8, anti-β-actin, and anti-GAPDH (all 1:1000, ZENBIO, Chengdu, China). Secondary antibodies consisted of HRP-conjugated AffiniPure goat anti-rabbit IgG and HRP-conjugated AffiniPure goat anti-mouse IgG (1:5000, ZSGB-BIO, Beijing, China).

### 2.3. Quantitative Real Time-PCR

Total RNA was isolated with the RNAiso Plus kit (YI FEI XUE Biotechnology Co., Ltd., Nanjing, China) according to the manufacturer’s protocol. Reverse transcription was performed using the Prime Script™ RT Master Mix (TaKaRa BioTechnology Co., Ltd., Dalian, China). Quantitative real-time PCR (qRT-PCR) was carried out using SYBR Premix Ex Taq II (Vazyme Biotech Co., Ltd, Nanjing, China) on a Roche Light Cycler 96 real-time PCR system (Roche, Basel, Switzerland). The sequences of all PCR primers employed are provided in Table 1. GAPDH served as the endogenous mRNA control. Relative expression fold changes were determined using the 2^−ΔΔCt^ method.

### 2.4. Plasmid Transfection

NSCLC cells were transfected with human AMPKα1 and PDZD8 plasmid or control plasmid (Guangzhou RiboBio, Co., Ltd., Guangzhou, China) using Lipofectamine 2000 (Invitrogen) as follows: (1) mix 200 μL of opti-MEM with 10 μL of Lipofectamine 2000; (2) mix 200 μL of opti-MEM with 10 μL of siRNA or plasmid; (3) after 5 min, combine the two mixtures and incubate for 20 min; (4) wash cells twice with PBS, and then add a mixture of 1600 μL of opti-MEM and 400 μL of the combined complexes to each well; (5) after 4–6 h of transfection, replace with normal medium for further culture or collection.

### 2.5. In Vivo Studies

Ten female SCID mice (4 weeks old, 18–20 g) were obtained from the Shanghai Animal Laboratory Center and housed in the Experimental Animal Center of Nanjing Medical University under controlled conditions (temperature: 22 ± 1 °C; humidity: 55 ± 5%). All mice received a subcutaneous injection of 5 × 10^6^ exponentially growing A549 sphere cells into the armpit. Tumor volumes were monitored every 2–3 days using a caliper to measure the longest diameter (L) and the shortest diameter (W), and the volume was calculated as V = (L × W^2^)/2. Each mouse was subcutaneously inoculated with one tumor. Two weeks post injection, 10 mice were randomly assigned to either a glutamine-free diet group or a normal diet group, with 5 mice in each group (the detailed composition of the diets is provided in Supplementary Table S1). Following 4 weeks of dietary treatment, all mice were euthanized by cervical dislocation, and tissue samples were collected for subsequent experiments.

### 2.6. Statical Analysis

All quantitative data are presented as the mean ± SD from a minimum of three independent replicates. Statistical comparisons were performed using Student’s *t*-test or one-way ANOVA, as appropriate. For multiple group comparisons, one-way ANOVA accompanied by Tukey’s post hoc test was applied. Analyses were carried out using SPSS 25.0 (SPSS Inc., Chicago, IL, USA), GraphPad Prism v8.0 (GraphPad Software Inc., La Jolla, CA, USA), and FlowJo V10. Data were considered statistically significant when *p* < 0.05.

## 3. Results

### 3.1. Glutamine Starvation Inhibited Proliferation in NSCLC Cells

**Figure 1:**
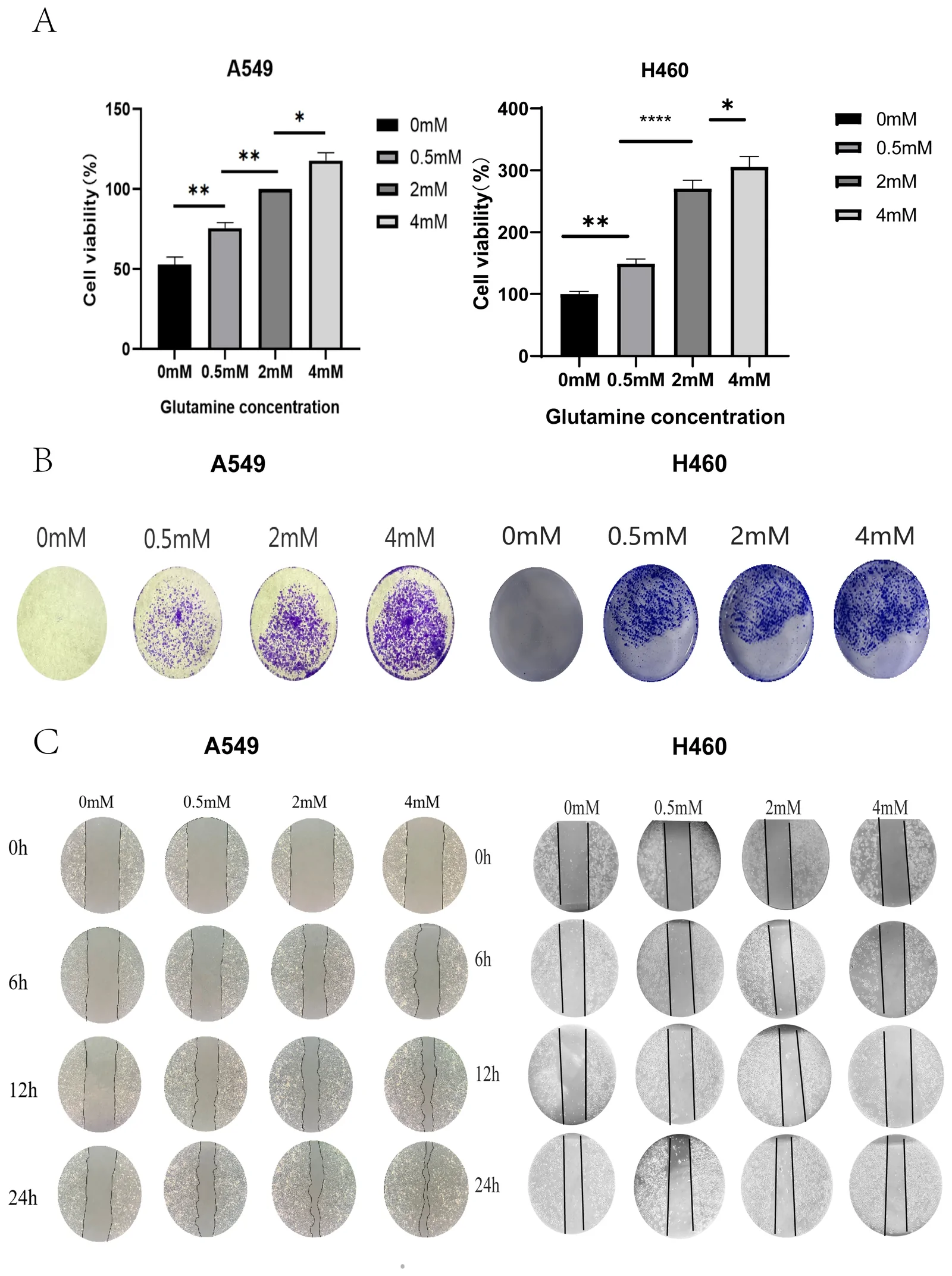
Glutamine inhibited NSCLC. (**A**) Cell viability was assessed by CCK-8 assay after culturing A549 and H460 cells for 48 h in media containing indicated concentrations of glutamine (4 mM, 2 mM, 0.5 mM, or 0 mM). The normal culture condition contains 4 mM glutamine, which served as the control. (**B**) Colony formation ability was evaluated by plate cloning assay under the same glutamine concentrations. (**C**) Plate scratch assays probed the cell migration ability after glutamine starvation treatment for 6 h, 12 h and 24 h. *n* = 3, * *p* < 0.05, ** *p* < 0.01, **** *p* < 0.0001 versus control group.

Glutamine is a conditionally essential amino acid in the body. This phenomenon of ‘glutamine addiction’ is frequently observed across various cancer types, particularly in non-small cell lung cancer (NSCLC). A549 and NCI-H460 NSCLC cells were utilized to assess the effects of glutamine starvation on tumor cell proliferation and survival. To this end, NSCLC cell lines A549 and NCI-H460 were treated with different concentrations (0, 0.5, 2, and 4 mM) of glutamine for 48 h. And then CCK-8 assays revealed a significant dose-dependent reduction in cell viability upon glutamine starvation, with the most pronounced suppression observed under complete starvation (Figure 1A). Consistent with this, clonogenic assays demonstrated marked inhibition of proliferative capacity in cells after glutamine starvation (Figure 1B). Furthermore, glutamine starvation substantially impaired the migratory ability of NSCLC cell lines (Figure 1C), suggesting a multifaceted role in suppressing tumor growth, metastasis, and disease progression.

### 3.2. Glutamine Starvation Triggered Ferritinophagy in NSCLC Cells

**Figure 2:**
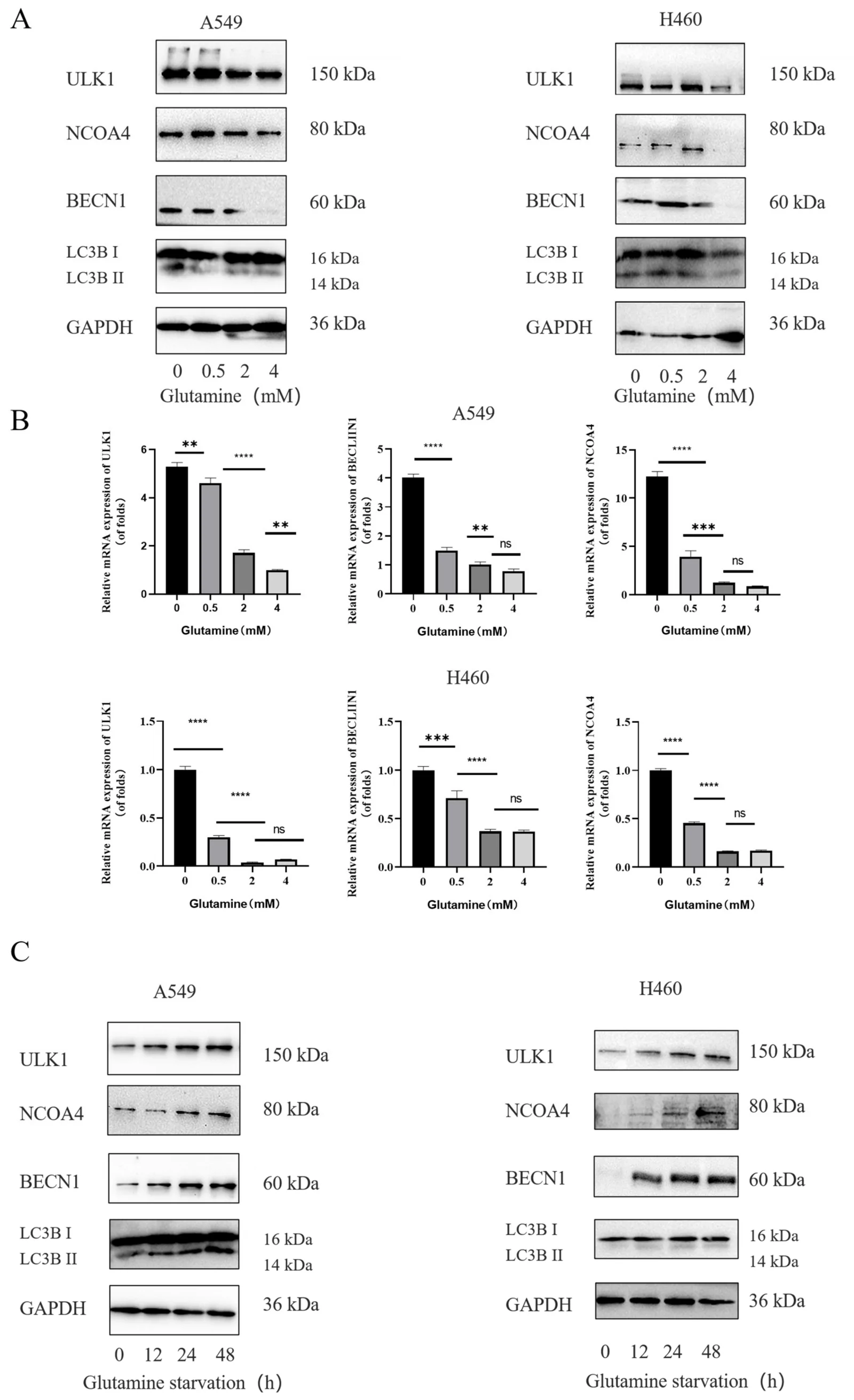
Effects of glutamine starvation on the ferritinophagy genes. (**A**) Western blot analysis of NCOA4, ULK1, BECN1, and LC3B expression in cells cultured with different concentrations of glutamine for 48 h. The 4 mM group represents normal culture condition and served as the control. (**B**) The mRNA levels of NCOA4, ULK1 and BECN1 in cells were detected by qRT-PCR after treatment with different concentrations of glutamine for 24 h. (**C**) Western blot analysis of NCOA4, ULK1, BECN1, and LC3B expression in cells subjected to glutamine starvation for the indicated durations (0, 12, 24, 48 h). The 48 h group served as the control. All data are presented as mean ± SD from three independent experiments. *n* = 3, ns: no significance. ** *p* < 0.01, *** *p* < 0.001, **** *p* < 0.0001 versus control group. Complete Western blot quantitative statistical analysis is provided in Supplementary File S1, Figures S1 and S2.

Research indicates that glutamine starvation reduces GSH synthesis and exacerbates oxidative stress, thereby activating autophagy. To investigate ferritinophagy under glutamine starvation, A549 and NCI-H460 cells were treated with progressively decreasing glutamine concentrations (4 mM, 2 mM, 0.5 mM, and 0 mM). Key markers of ferritinophagy (NCOA4) and autophagy initiation (ULK1, BECN1, and LC3B II/I) were assessed. As shown in Figure 2A,B, glutamine starvation significantly upregulated NCOA4, ULK1, BECN1, and LC3B II/I expression at both protein and mRNA levels. The effects were most pronounced under complete glutamine starvation. Furthermore, prolonged glutamine starvation (0 h, 12 h, 24 h, and 48 h) induced consistent alterations in these markers (Figure 2C). Collectively, these findings demonstrate that glutamine starvation triggers ferritinophagy in a dual dose- and time-dependent manner.

### 3.3. Glutamine Starvation Triggered Ferroptosis in NSCLC Cells

**Figure 3:**
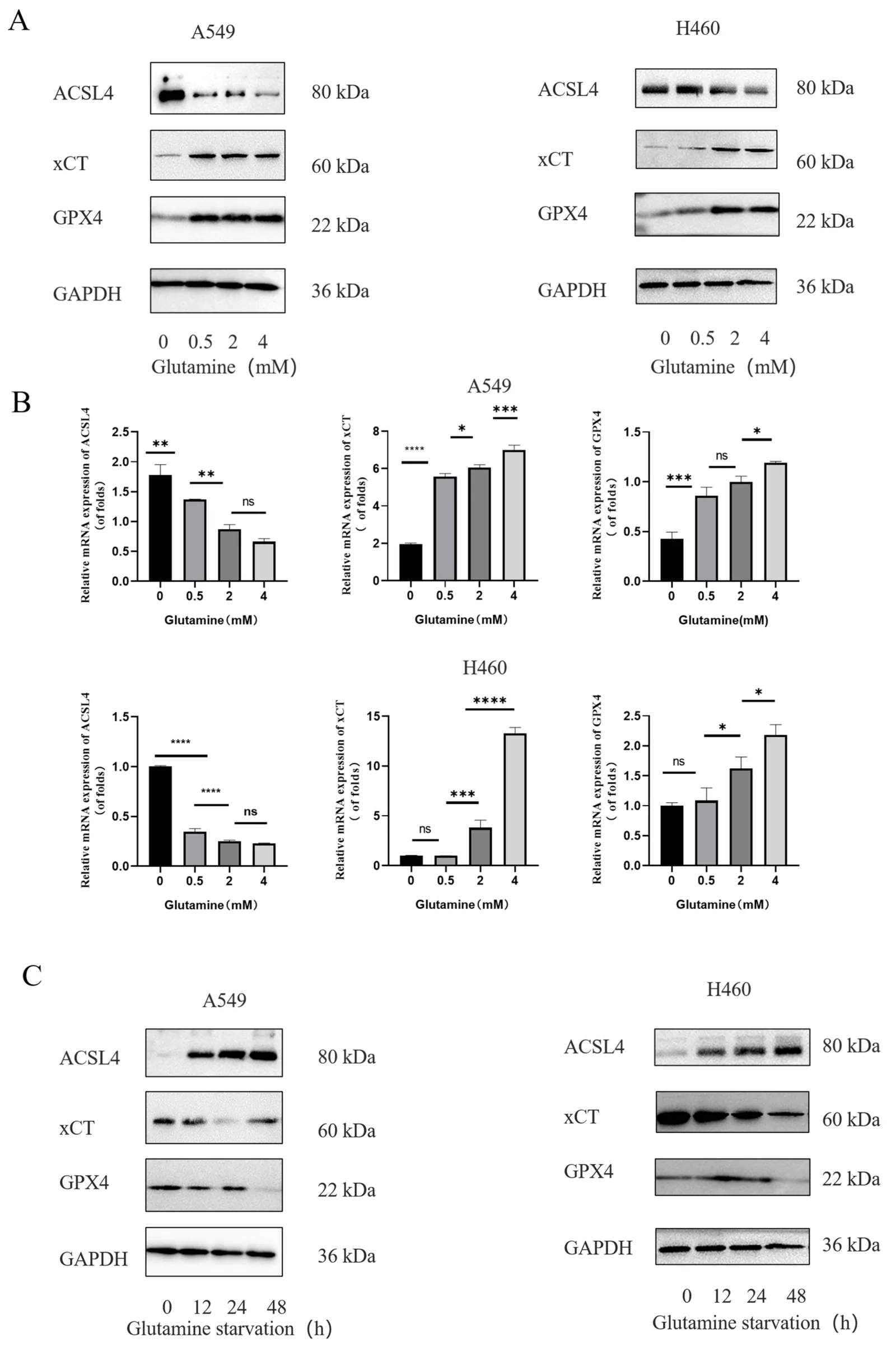
Effects of glutamine starvation on the ferroptosis genes. (**A**) Western blot analysis of ACSL4, xCT, and GPX4 protein expression in A549 and H460 cells cultured for 48 h in media containing indicated concentrations of glutamine (4, 2, 0.5, and 0 mM). The 4 mM group represents the normal culture condition and served as the control. (**B**) Quantitative real-time PCR (qRT-PCR) analysis of ACSL4, xCT, and GPX4 mRNA levels after 24 h of treatment with the same glutamine concentration gradient. (**C**) Western blot analysis of the same proteins in cells subjected to glutamine starvation (0 mM) for the indicated durations (0, 12, 24, and 48 h). All data are presented as mean ± SD from three independent experiments. *n* = 3, ns: no significance. * *p* < 0.05, ** *p* < 0.01, *** *p* < 0.001, **** *p* < 0.0001 versus control group. Complete Western blot quantitative statistical analysis is provided in Supplementary File S1, Figures S3 and S4.

Ferritinophagy activation can degrade ferritin to increase intracellular iron levels, and subsequently results in oxidative injury by the Fenton reaction [25]. We next investigated whether glutamine starvation could also induce ferroptosis, a non-apoptotic iron-dependent form of cell death triggered by peroxidation of polyunsaturated fatty acids (PUFAs) [26]. As demonstrated in our results, the protein and mRNA expression levels of ACSL4 were significantly upregulated in response to decreasing glutamine concentrations (4 mM, 2 mM, 0.5 mM, and 0 mM), whereas GPX4 and xCT exhibited opposing trends (Figure 3A,B). These findings, together with the observed ferritinophagy activation (Figure 2), indicate that glutamine starvation, known to induce ferritinophagy, promotes ferroptosis. Notably, similar to ferritinophagy, prolonged glutamine starvation (0 h, 12 h, 24 h, and 48 h) led to progressive changes in ferroptosis biomarkers, further supporting the functional link between these two processes (Figure 3C).

### 3.4. Glutamine Starvation Enhances the Expression of AMPK in NSCLC Cells

**Figure 4:**
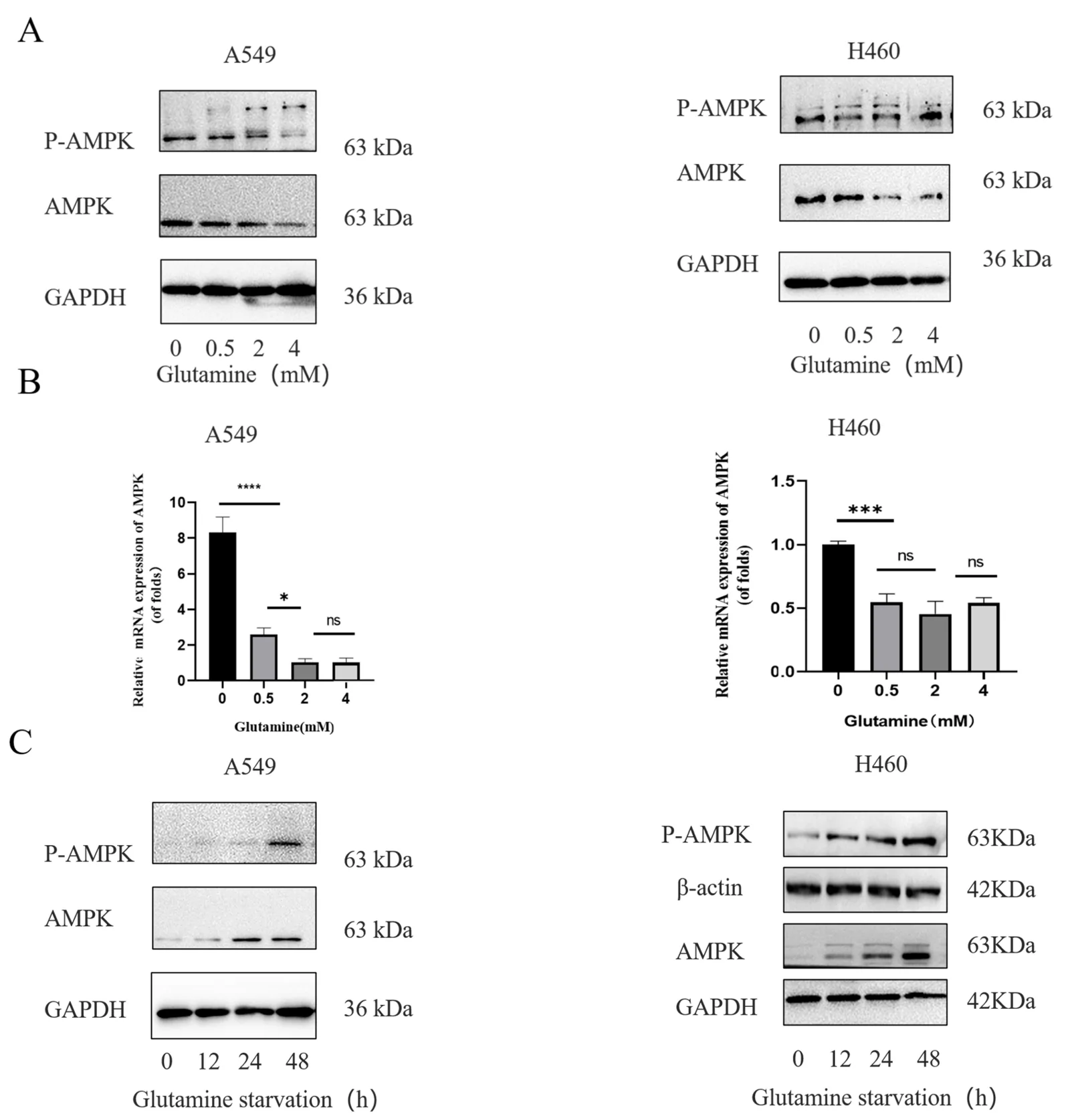
Effects of glutamine starvation on the AMPK and *p*-AMPK genes. (**A**) Western blot analysis of AMPK and *p*-AMPK protein expression in A549 and H460 cells cultured for 48 h in media containing indicated concentrations of glutamine (4, 2, 0.5, and 0 mM). The 4 mM group represents the normal culture condition and served as the control. (**B**) Quantitative real-time PCR (qRT-PCR) analysis of *AMPK* mRNA levels after 24 h of treatment with the same glutamine concentration gradient. (**C**) Western blot analysis of AMPK and *p*-AMPK expression in cells subjected to glutamine starvation (0 mM) for the indicated durations (0, 12, 24, and 48 h). All data are presented as mean ± SD from three independent experiments. *n* = 3, ns: no significance. * *p* < 0.05, *** *p* < 0.001, **** *p* < 0.0001 versus control group. Complete Western blot quantitative statistical analysis is provided in Supplementary File S1, Figure S5.

Existing studies have shown that AMPK responds to amino acid starvation. Here, we found that glutamine starvation markedly increased both protein and mRNA levels of AMPK in NSCLC cells, with the most significant upregulation observed under complete glutamine withdrawal (0 mM), as shown in Figure 4A,B. Furthermore, we found that as the duration of glutamine starvation extended (0 h, 12 h, 24 h, and 48 h), the protein expression of AMPK in NSCLC cells also increased (Figure 4C). Notably, the level of *p*-AMPK exhibited a synchronous upward trend with total AMPK. Together, these results demonstrate that in NSCLC cells, glutamine starvation not only regulates AMPK expression at both transcriptional and translational levels but also markedly enhances its phosphorylation modification, suggesting that glutamine starvation may involve cellular stress responses through the AMPK signaling pathway.

### 3.5. AMPK Stimulates Ferritinophagy

**Figure 5:**
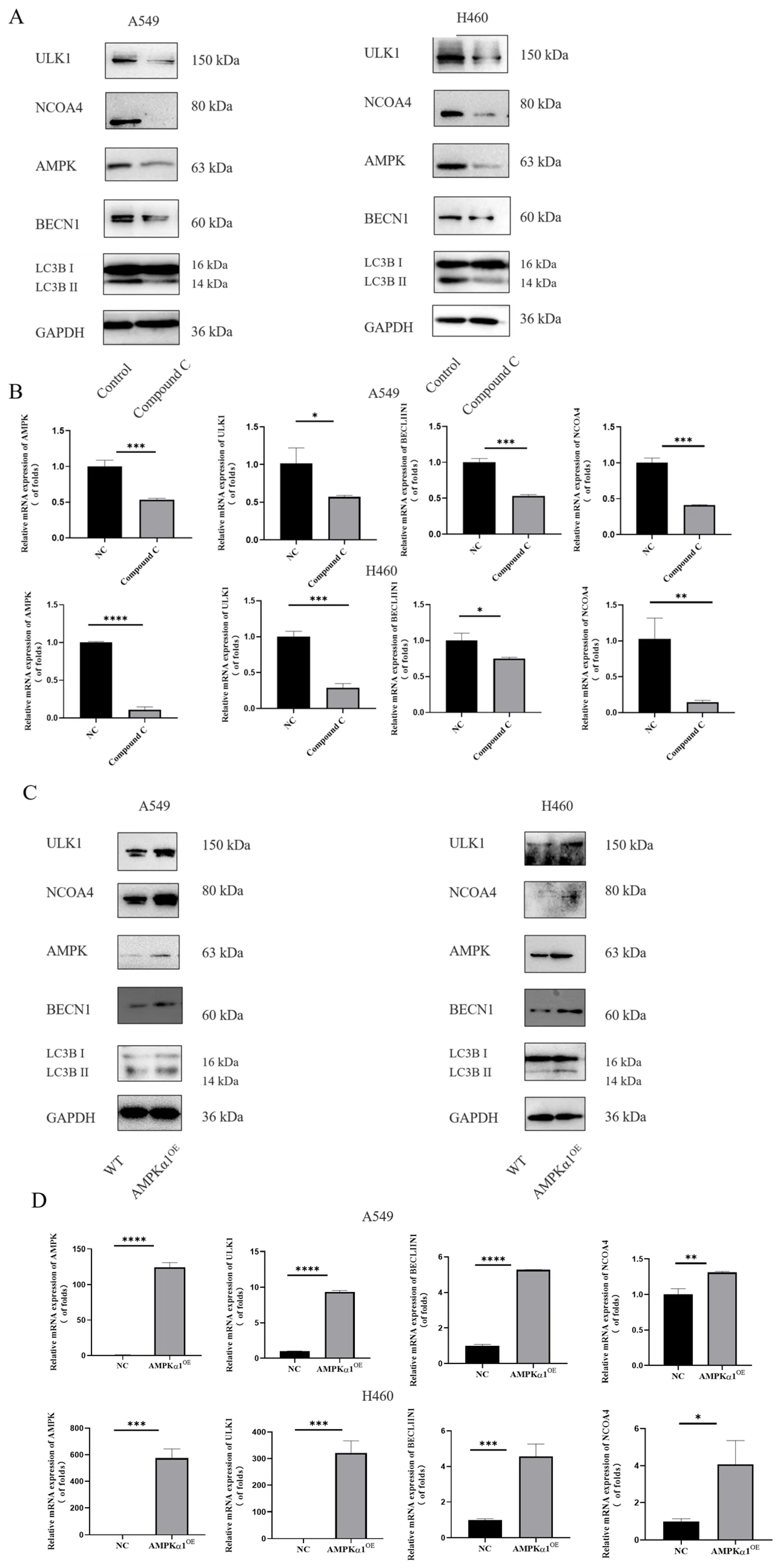
Role of AMPK in the regulation of ferritinophagy. (**A**) Following treatment of A549 and 460 cells with the AMPK inhibitor Compound C, the expression levels of NCOA4, ULK1, BECN1 and LC3B were analyzed by Western blot. (**B**) Following treatment of A549 and 460 cells with the AMPK inhibitor Compound C, the expression levels of NCOA4, ULK1 and BECN1 were analyzed by qRT-PCR. (**C**,**D**) After A549 and 460 cells were transfected with AMPKα1 overexpression plasmid, the expression levels of NCOA4, ULK1, BECN1, and LC3B were analyzed by qRT-PCR and Western blot. *n* = 3, * *p* < 0.05, ** *p* < 0.01, *** *p* < 0.001, **** *p* < 0.0001 versus control group. Complete Western blot quantitative statistical analysis is provided in Supplementary File S1, Figures S6 and S7.

To assess the role of AMPK in regulating ferritinophagy, we modulated its activity in A549 and NCI-H460 cells. Inhibition of AMPK using Compound C (10 μM) significantly suppressed the expression of key ferritinophagy markers, including ULK1, BECN1, NCOA4, and the LC3-II/I (Figure 5A,B). Conversely, AMPK overexpression enhanced intracellular ferritinophagy, as evidenced by increased levels of these same markers (Figure 5C,D). These results indicate that AMPK activation is a potent inducer of the ferritinophagy pathway.

### 3.6. AMPK Stimulates Ferritinophagy to Induce Ferroptosis

**Figure 6:**
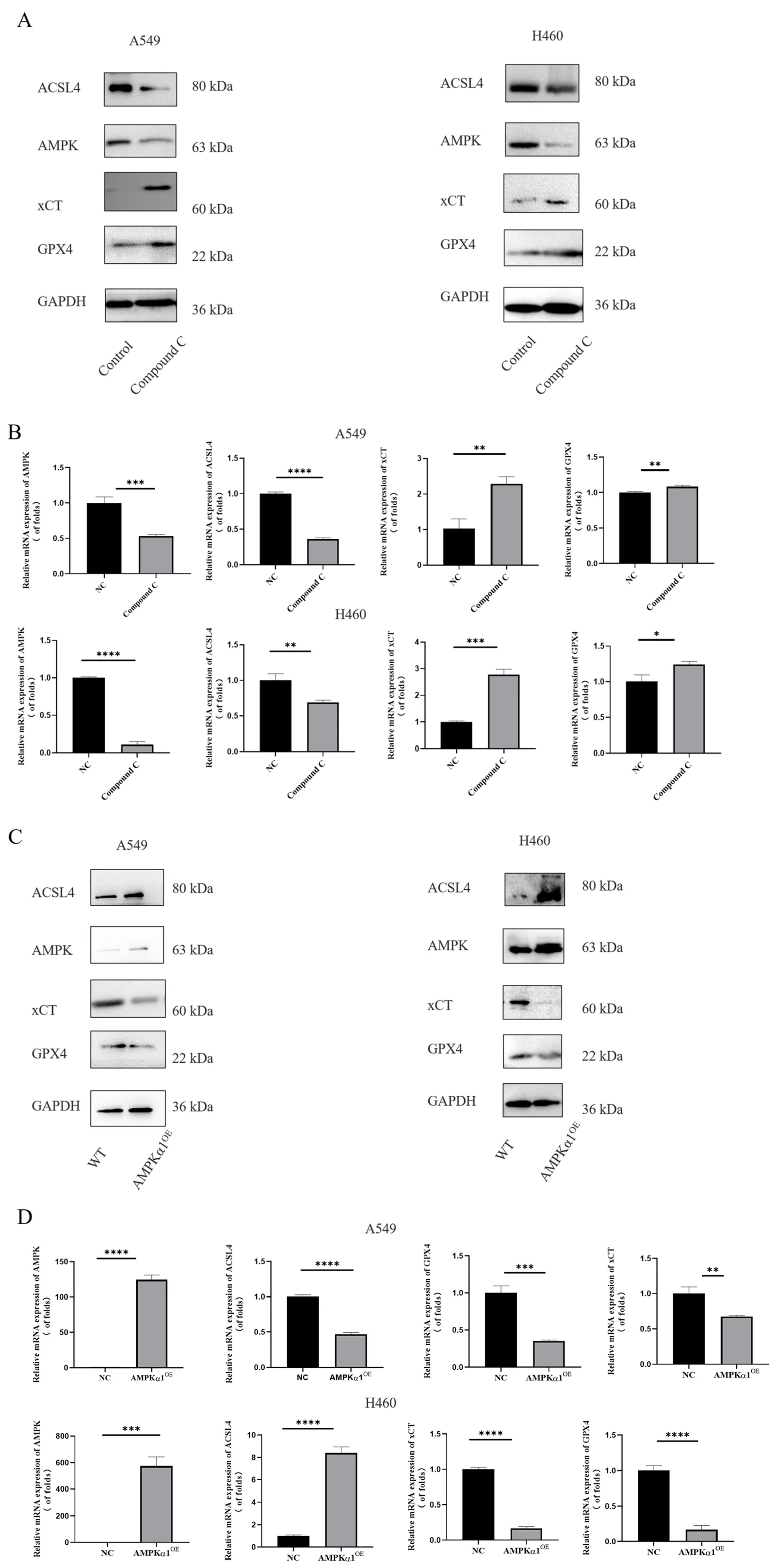
Role of AMPK in the regulation of ferroptosis. (**A**) Following treatment of A549 and 460 cells with the AMPK inhibitor Compound C, protein expression levels of AMPK, ACSL4, xCT, and GPX4 were analyzed by Western blot. (**B**) Following treatment of A549 and 460 cells with the AMPK inhibitor Compound C, corresponding mRNA levels of AMPK, ACSL4, xCT, and GPX4 were analyzed by qRT-PCR. (**C**) After A549 and 460 cells were transfected with AMPKα1 overexpression plasmid, protein expression levels of AMPK, ACSL4, xCT, and GPX4 were assessed by Western blot. (**D**) After A549 and 460 cells were transfected with AMPKα1 overexpression plasmid, mRNA levels of AMPK, ACSL4, xCT, and GPX4 were assessed by qRT-PCR. All data are presented as mean ± SD from three independent experiments. *n* = 3, * *p* < 0.05, ** *p* < 0.01, *** *p* < 0.001, **** *p* < 0.0001 versus control group. Complete Western blot quantitative statistical analysis is provided in Supplementary File S1, Figures S8 and S9.

We next sought to determine whether AMPK-driven ferritinophagy contributes to the induction of ferroptosis. Pharmacological inhibition of AMPK significantly increased the protein levels of the ferroptosis suppressors GPX4 and xCT, while decreasing the level of the pro-ferroptotic marker ACSL4 (Figure 6A,B). Conversely, overexpression of AMPK robustly upregulated ACSL4 expression and downregulated both GPX4 and xCT (Figure 6C,D). Collectively, these data indicate that AMPK activation promotes ferroptosis, most likely through its role in enhancing ferritinophagy.

### 3.7. Glutamine Starvation Enhances the Expression of PDZD8 via AMPK Activation in NSCLC Cells

**Figure 7:**
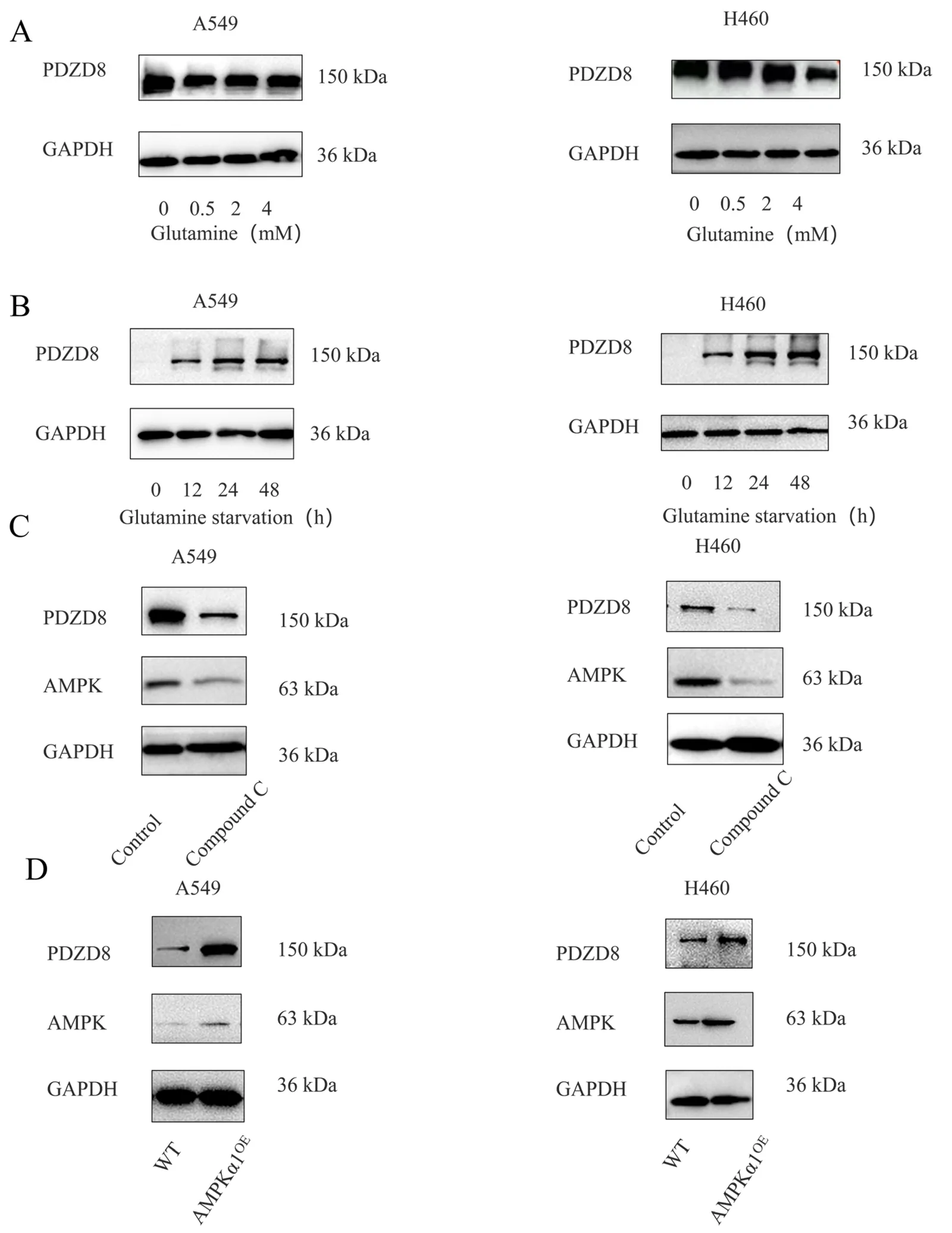
Role of AMPK in the regulation of PDZD8. (**A**) Western blot analysis of PDZD8 protein expression in A549 and H460 cells cultured for 48 h in media containing indicated concentrations of glutamine (4, 2, 0.5, and 0 mM). The 4 mM group represents the normal culture condition and served as the control. (**B**) Western blot analysis of PDZD8 expression in cells subjected to glutamine starvation (0 mM) for the indicated durations (0, 12, 24, and 48 h). (**C**) A549 and H460 cells were treated with the AMPK inhibitor Compound C and PDZD8 protein levels were analyzed by Western blot. (**D**) A549 and H460 cells were transfected with an AMPKα1 overexpression plasmid and, after 48 h, PDZD8 protein levels were analyzed by Western blot. All data are presented as mean ± SD from three independent experiments. *n* = 3. Complete Western blot quantitative statistical analysis is provided in Supplementary File S1, Figure S10.

PDZD8, as an anchor protein at endoplasmic reticulum-mitochondria contact sites, mediates lipid transport and Ca^2+^ signaling. Experimental results demonstrated that glutamine starvation induced an upregulation of PDZD8 expression in A549 and NCI-H460 cells in a time-dependent manner. The protein level of PDZD8 increased most significantly under 0 mM glutamine concentration and after 48 h of complete glutamine starvation (Figure 7A,B). Crucially, this increase in expression was dependent on AMPK, as evidenced by the fact that AMPK overexpression markedly enhanced PDZD8 protein levels, whereas treatment with an AMPK inhibitor significantly reduced its expression (Figure 7C,D). In summary, this study reveals that AMPK drives the upregulation of PDZD8 expression, providing a novel physiological mechanism for the suppression of NSCLC cell growth under glutamine starvation.

### 3.8. PDZD8 Stimulates Ferritinophagy to Induce Ferroptosis

**Figure 8:**
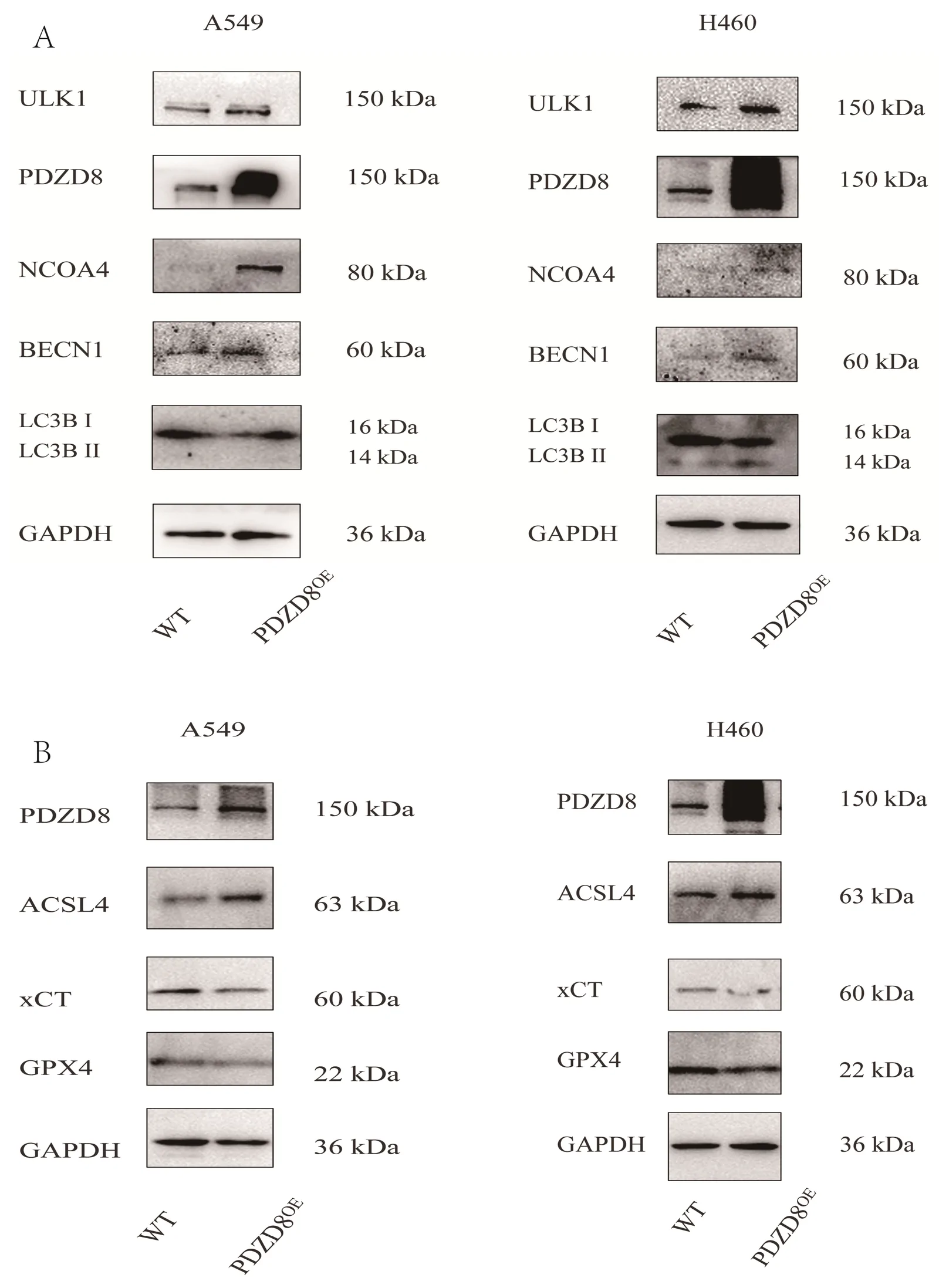
Role of PDZD8 in the regulation of ferritinophagy and ferroptosis. (**A**) A549 and H460 cells were transfected with a PDZD8 overexpression plasmid. After 48 h, protein expression levels of NCOA4, ULK1, BECN1, and LC3B were analyzed by Western blot. (**B**) Following the same transfection with PDZD8 overexpression plasmid, protein expression levels of ACSL4, GPX4, and xCT were analyzed by Western blot. All data are presented as mean ± SD from three independent experiments. *n* = 3. Complete Western blot quantitative statistical analysis is provided in Supplementary File S1, Figures S11 and S12.

Given that the above results indicate that AMPK activation significantly upregulates PDZD8 expression, we next investigated whether PDZD8 acts as a key downstream effector molecule of AMPK, mediating the occurrence of ferritinophagy and ferroptosis. To this end, we overexpressed PDZD8 in A549 and NCI-H460 cells and detected its effects on the protein expression of markers related to ferritinophagy and ferroptosis. The results showed an increase in ferritinophagy levels, specifically manifested as elevated levels of ULK1, BECN1, NCOA4, and an increased LC3-II/I ratio (Figure 8A). Similarly, ferroptosis was enhanced, as indicated by increased ACSL4 expression and decreased expression of GPX4 and xCT (Figure 8B).

### 3.9. Glutamine Starvation Inhibited the Growth of Tumor Xenografts and Enhanced the Expression of AMPK, PDZD8, Ferritinophagy and Ferroptosis Biomarkers In Vivo

**Figure 9:**
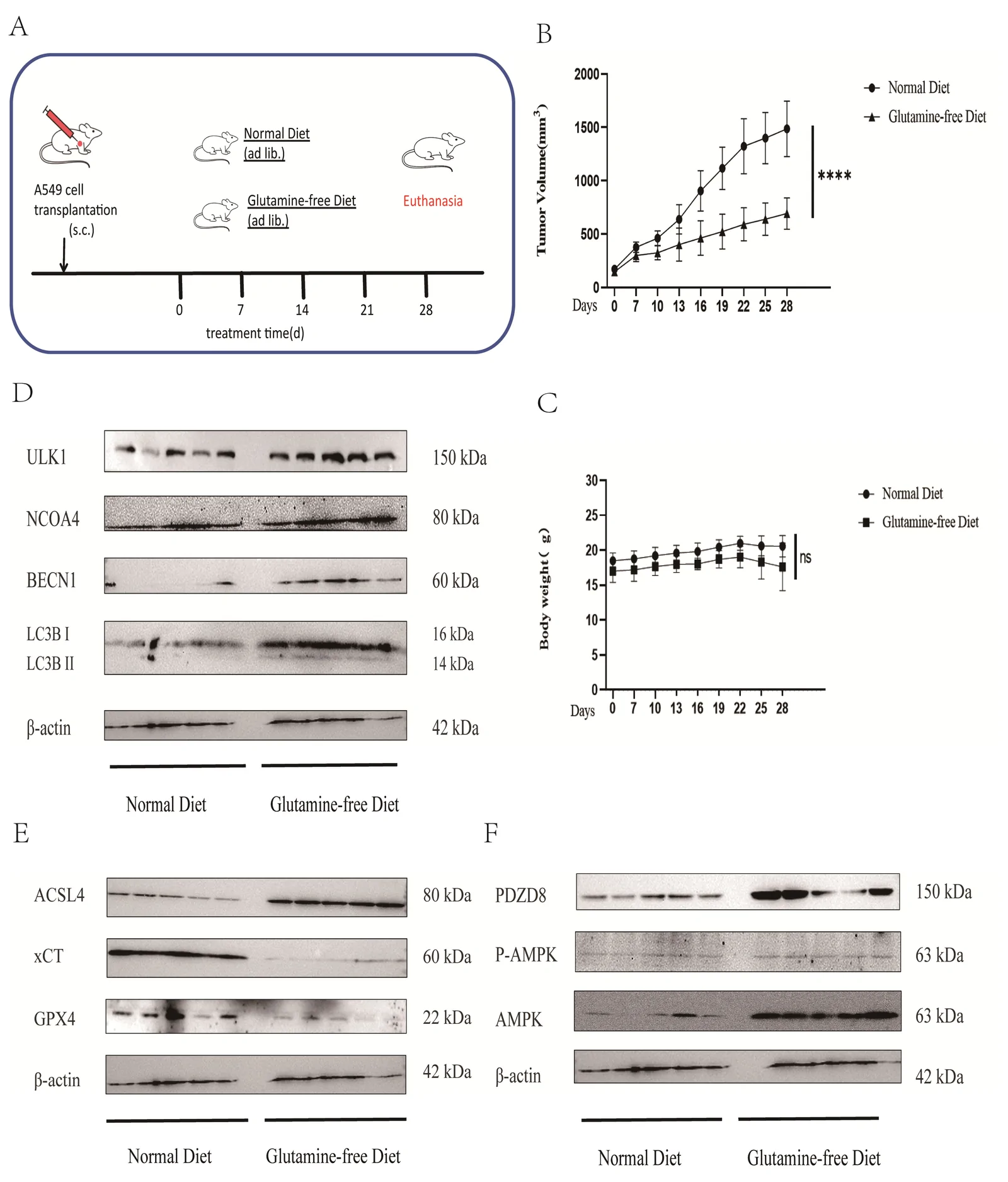
Glutamine starvation delays tumor growth and activates the expression of ferritinophagy, ferroptosis-related proteins, AMPK, and PDZD8 in xenograft tumor model mice. (**A**) Schematic diagram of the experimental grouping for the xenograft tumor model in SCID mice. Mice were subcutaneously injected with A549 or H460 cells and then fed a normal diet (control group) or a glutamine-free diet (glutamine starvation group). (**B**) Tumor volume measured at indicated time points post inoculation (e.g., every 2–3 days) in the two groups. (**C**) Body weight changes of mice in each group recorded over the same time course. (**D**) Western blot analysis of NCOA4, ULK1, BECN1, and LC3B protein expression in tumor tissue lysates from control versus glutamine-starved mice. (**E**) Western blot analysis of ACSL4, GPX4, and xCT protein expression in the same tumor tissues. (**F**) Western blot analysis of AMPK, *p*-AMPK, and PDZD8 protein expression. All Western blot data are representative of three independent tumor samples per group. Data are presented as mean ± SD. *n* = 5, ns: no significance. **** *p* < 0.0001 versus control group. Complete Western blot quantitative statistical analysis is provided in Supplementary File S1, Figure S13.

To investigate whether glutamine starvation affects tumor growth, we used a mouse xenograft model. Figure 9A outlines the experimental grouping. Figure 9B reveals that mice fed a glutamine-deficient diet exhibited a significant time-dependent reduction in tumor volume compared to those on a normal diet. Meanwhile, no notable differences in body weight trends were observed between the groups (Figure 9C). Upon termination of the treatment, tumor tissues were collected for further analysis. The results demonstrated a significant upregulation of ferritinophagy markers, including ULK1, BECN1, NCOA4, and the LC3B-II/I ratio, in the glutamine starvation group (Figure 9D). Additionally, the expression of the ferroptosis-related protein ACSL4 was markedly increased, while GPX4 and xCT levels were significantly decreased (Figure 9E). Consistent with these findings, the protein expression levels of AMPK, *p*-AMPK, and PDZD8 were also significantly elevated in the tumors from the glutamine-deficient group (Figure 9F).

## 4. Discussion

Glutamine metabolism is fundamental for maintaining cellular homeostasis and mitigating oxidative damage in cancer cells [27]. The cytotoxic consequences of glutamine starvation have been extensively observed across various tumor types [28,29,30]. The present study elucidates a previously unrecognized signaling cascade wherein glutamine starvation inhibits proliferation and survival in non-small cell lung cancer (NSCLC) by inducing ferroptosis through the activation of the AMPK/PDZD8 axis and subsequent ferritinophagy. While the cytotoxic effects of glutamine starvation on tumor cells are well-documented, and ferroptosis has emerged as a critical regulated cell death modality, the mechanistic link connecting glutamine availability, the energy sensor AMPK, the mitochondrial-associated protein PDZD8, ferritinophagy, and ferroptosis had remained unexplored. Our findings not only delineate this novel signaling pathway but also provide a compelling rationale for developing innovative therapeutic and dietary intervention strategies for NSCLC.

In the present study, A549 and NCI-H460 cells were chosen because both possess activating KRAS mutations that drive glutamine dependency for anaplerosis and maintenance of redox balance [31]. Moreover, they cover distinct genetic backgrounds, allowing us to test glutamine starvation efficacy across common NSCLC subtypes. Both lines have been well validated as glutamine-addicted and showed consistent responses in our pilot experiments.

Numerous inhibitors targeting glutamine metabolism have entered preclinical development or clinical trials. Chemical agents targeting GLSs have been studied, and CB-839, 968, and BPTES have been found to exhibit tumor-specific antiproliferative effects [32]. Among these agents, CB-839 is the only one to proceed to clinical trials; however, its selectivity toward GLS1 and failure to inhibit the compensatory effect of GLS2 require in-depth study [33]. The ASCT2 inhibitor V-9302 is theoretically more comprehensive than GLS1 inhibitors, as it blocks glutamine entry and downstream signaling while also affecting other ASCT2 substrates, thereby enhancing antitumor efficacy [34]. However, its clinical development has been hindered by poor water solubility, the need for nanoparticle-based delivery, and metabolic compensation in tumors [35]. In light of these limitations, this study chose to directly deprive the tumor microenvironment of glutamine. This strategy simultaneously blocks multiple metabolic pathways, confers a low risk of drug resistance, and is both feasible and manageable in terms of potential malnutrition.

Our data demonstrate that glutamine starvation promotes AMPK activation and upregulates PDZD8 expression. AMPK, a master regulator of cellular energy homeostasis, has previously been reported to be activated upon glutamine starvation, consistent with the role of glutamine in ATP production in various cell types [36]. This aligns with our observations. Critically, we identify AMPK as a key upstream regulator of PDZD8 in this context, establishing a direct link between energy sensing and the machinery involved in ferritinophagy and ferroptosis.

Ferritin serves as the primary intracellular iron storage complex, and its selective autophagic degradation (ferritinophagy) is mediated by the cargo receptor NCOA4. This process elevates labile Fe^2+^ levels, which can subsequently catalyze lipid peroxidation via the Fenton reaction, generating abundant lipid peroxides that compromise the integrity and function of cellular membranes, including the plasma membrane, mitochondrial membrane, and endoplasmic reticulum. Concomitant mitochondrial morphological changes, such as increased membrane density, cristae reduction or disappearance, and outer membrane rupture [37,38], are characteristic features culminating in ferroptosis [39], Ferroptosis is a form of programmed cell death intimately linked to amino acid metabolism. Previous studies have implicated ferroptosis in the suppression of pancreatic cancer growth upon glutamine starvation, potentially through reactive oxygen species (ROS) generation [10] or cysteine depletion [28]. Our results substantiate these findings by demonstrating that glutamine starvation concurrently induces both ferritinophagy and ferroptosis in A549 and NCI-H460 NSCLC cells.

Recent work by Thakur and O’Connor-Giles highlighted a role for PDZD8 at membrane contact sites between the endoplasmic reticulum (ER) and late endosomes/lysosomes, where it promotes autophagy by coupling lipid transfer to autolysosome maturation, a process critical for synaptic terminal formation [40]. The study found that AMPK can mediate autophagy and ferroptosis, which is consistent with existing research results. Notably, in this experimental model, overexpression of PDZD8 enhanced the occurrence of ferritinophagy and ferroptosis. Through overexpression experiments of AMPK and PDZD8 in A549 and NCI-H460 cells, this study demonstrated that the AMPK/PDZD8 axis can induce ferritinophagy and ferroptosis.

PDZD8 is also a recognized component of ER-mitochondria contact sites [41]. However, the precise molecular mechanisms by which AMPK activation enhances PDZD8 function remain to be fully elucidated. Potential mechanisms could include direct phosphorylation of PDZD8 by AMPK, AMPK-mediated transcriptional regulation of PDZD8, or other post-translational modifications. Unraveling the nature of this interaction represents a critical and exciting avenue for future research. It will be important to determine whether AMPK directly modifies PDZD8 to enhance its activity in ferritinophagy or whether indirect effects, such as alterations in the cellular metabolome or contact site dynamics, are involved.

In conclusion, our study demonstrates that glutamine starvation, both in vitro and in vivo, suppresses NSCLC growth by upregulating ferritinophagy and ferroptosis through the AMPK/PDZD8 axis. We show that reducing or eliminating glutamine from the culture medium inhibits the growth and migration of A549 and NCI-H460 cells. Consistently, in an SCID mouse xenograft model, dietary glutamine starvation significantly suppressed tumor growth compared to a normal diet, without inducing significant weight loss. Mechanistically, our in vivo and in vitro data confirm that glutamine starvation increases ferritinophagy and ferroptosis, concomitant with elevated AMPK and PDZD8 protein expression. Furthermore, we establish that AMPK and PDZD8 can modulate the occurrence of ferritinophagy and ferroptosis.

A limitation of this study is the lack of comparative experiments using normal lung epithelial cells (e.g., BEAS-2B). All mechanistic and phenotypic data were derived exclusively from NSCLC cell lines (A549 and NCI-H460). Therefore, future studies incorporating normal cell controls will be essential to assess the tumor selectivity and therapeutic window of glutamine-starvation-based strategies.

Currently, dietary-induced glutamine starvation restricts glutamine-rich foods (e.g., meat, eggs, dairy, soy). It is physiological and non-toxic but has limitations: poor compliance, incomplete depletion, and risk of malnutrition. Notably, in critically ill patients with multiorgan failure, early supplementation with exogenous glutamine not only failed to improve clinical outcomes but was instead associated with increased mortality [42]. Similarly, glutamine supplementation in patients with traumatic brain injury may be linked to glutamate accumulation and neurotoxicity [43]. In hepatocellular carcinoma (HCC), glutamine promotes cancer progression by enhancing cholesterol synthesis and platelet-mediated tumor growth [44]. Therefore, glutamine starvation may hold therapeutic potential in the oncological setting, supporting a tumor-suppressive strategy targeting glutamine starvation from a metabolic perspective [45].

## 5. Conclusions

This study demonstrates that glutamine starvation suppresses NSCLC tumor growth by activating ferritinophagy and ferroptosis through the AMPK/PDZD8 pathway. In vitro and in vivo experiments confirmed that glutamine starvation inhibits cancer cell proliferation and upregulates AMPK and PDZD8. PDZD8 was identified as a critical downstream effector, as its overexpression alone was sufficient in inducing both ferritinophagy and ferroptosis. These findings reveal a direct link between glutamine stress and ferroptosis in NSCLC, supporting the therapeutic potential of dietary glutamine starvation.

## Figures and Tables

**Table 1:** The primers for qPCR (human).

Gene	Forward Primer (5′-3′)	Reverse Primer (5′-3′)
*GPX4*	ACAAGAACGGCTGCGTGGTGAA	GAGCTAGAAATAGTGGGGCAGGT
*ACSL4*	TGGCAAAGGAGCAGATTAGTAGG	TCACTTAGGATTTCCCTGGTCC
*AMPK*	GGAGCCTTGATATGGTAGGA	CATCCAGCCTTCCATTCTTACAG
*GAPDH*	CAAGGTCATCCATGACAACTTTG	GTCCACCACCCTGTTGCTGTAG
*ULK1*	GGCAAGTTCGAGTTCTCCCG	CGACCTCCAAATCGTGCTTCT
*BECN1*	GGAGCTGCCGTTATACTGTTCTGG	TGCCTCCTGTGTCTTCAATCTTGC
*NCOA4*	CAGCAGCTCTACTCGTTATTGG	TCTCCAGGCACACAGAGACT
*xCT*	TGTCTCCAGGTTATTCTATGTTG	CCAGAGAAGAGCATTATCATTG

## Data Availability

The original contributions presented in this study are included in the article/Supplementary Materials. Further inquiries can be directed to the corresponding author.

## Supplementary Materials

The following supporting information can be downloaded at: https://www.mdpi.com/article/10.3390/nu18101596/s1, File S1: Densitometric quantification of Wetern Blot; File S2: Supplementary experimental data and corresponding original Western blot images; File S3: Original Western blot images of the manuscript. Table S1: Detailed mouse diet composition.
