# Supplementary material for: Glutamine Starvation Induces Ferroptosis in NSCLC via AMPK/PDZD8-Mediated Ferritinophagy
Source: Nutrients. 2026 May 18;18(10):1596. doi: 10.3390/nu18101596 (PMC13209610; doi:10.3390/nu18101596)
Supplement: Supplementary file 1 [file nutrients-18-01596-s001.zip › File S1.pdf]

FigureS1

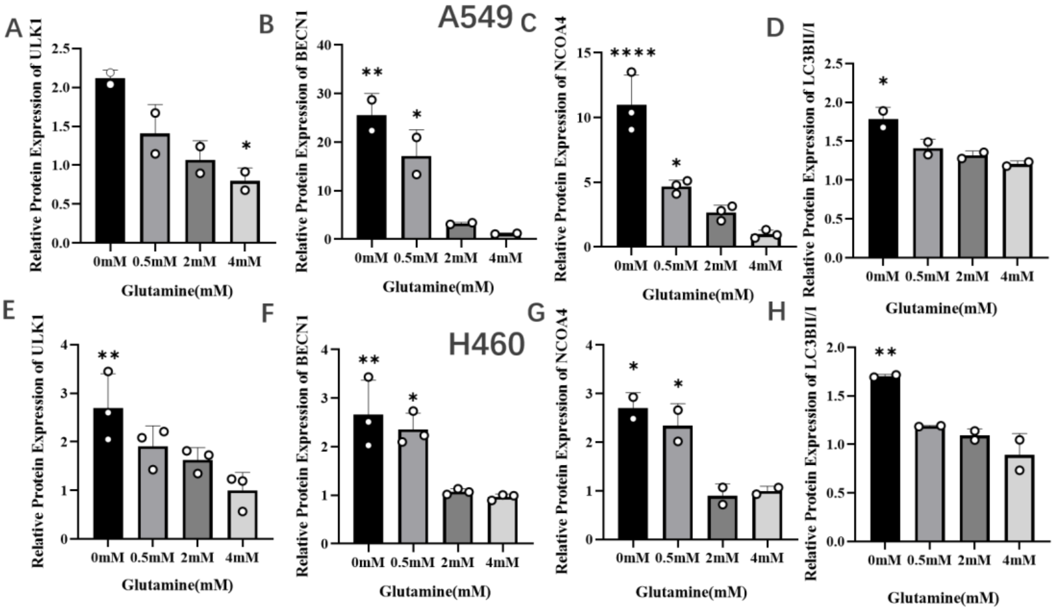

Figure S1. Densitometric quantification of ferritinophagy protein expression under glutamine starvation.

(A-H) Densitometric quantification of ULK1, BECN1, NCOA4 and LC3B-II/I in NSCLC cells treated with 0, 0.5, 2, and 4 mM glutamine (4 mM as control).

FigureS2

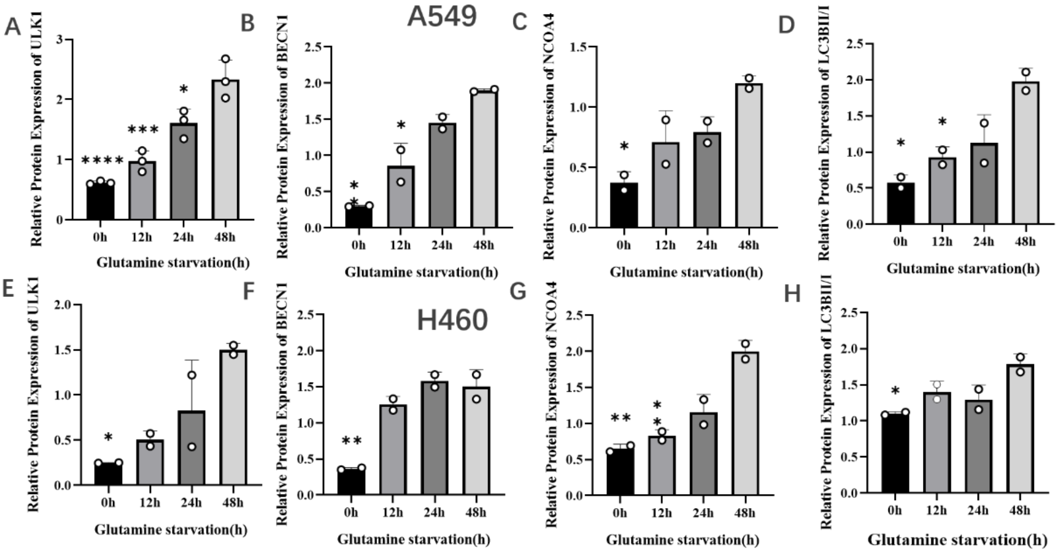

Figure S2. Densitometric quantification of ferritinophagy protein expression under glutamine starvation.

(A-H)Densitometric quantification of ULK1, BECN1, NCOA4 and LC3B-II/I in

NSCLC cells treated with 0, 12, 24, and 48 h complete glutamine starvation. (48h as control)

FigureS3

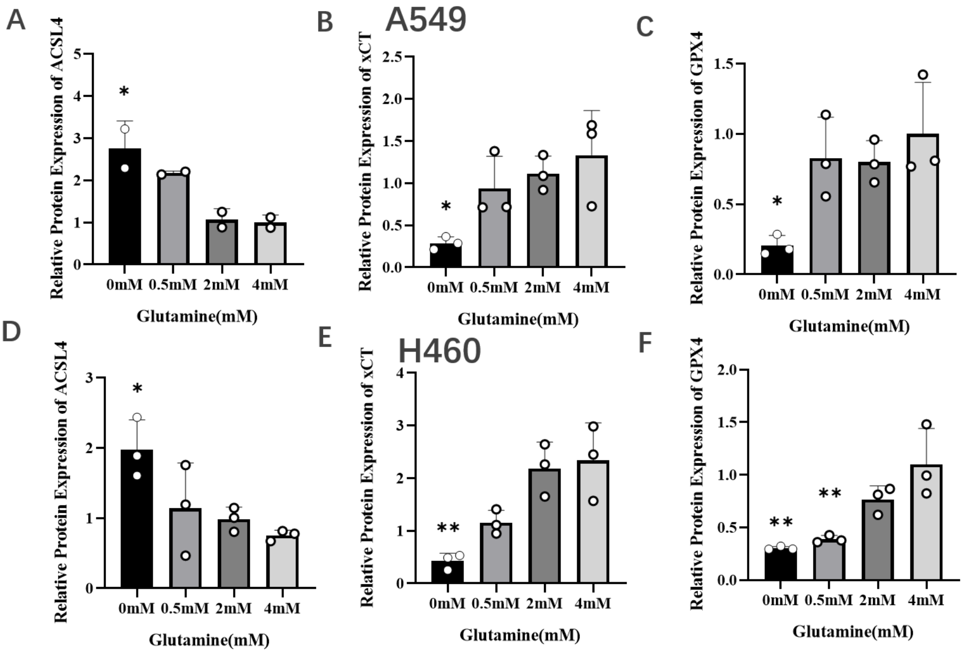

Figure S3. Densitometric quantification of ferroptosis protein expression under glutamine starvation.

(A-F) Densitometric quantification of ACSL4, xCT, and GPX4 in NSCLC cells treated with 0, 0.5, 2, and 4 mM glutamine (4 mM as control).

FigureS4

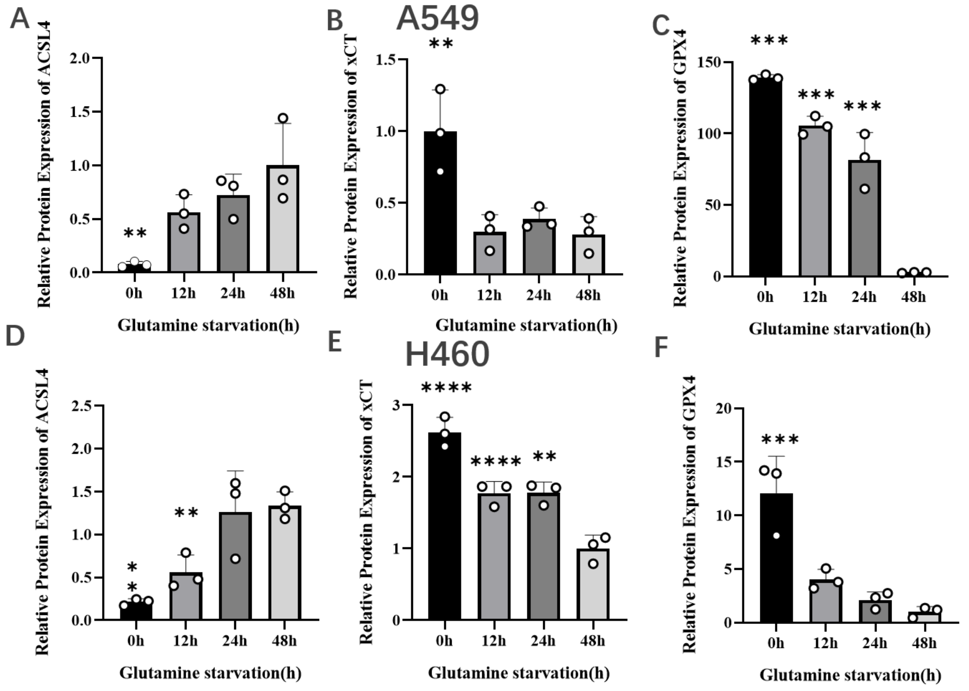

Figure S4. Densitometric quantification of ferroptosis protein expression under glutamine starvation.

(A-F) Densitometric quantification of ACSL4, xCT, and GPX4 in NSCLC cells treated with 0h, 12h, 24h, and 48h glutamine starvation (48h as control).

FigureS5

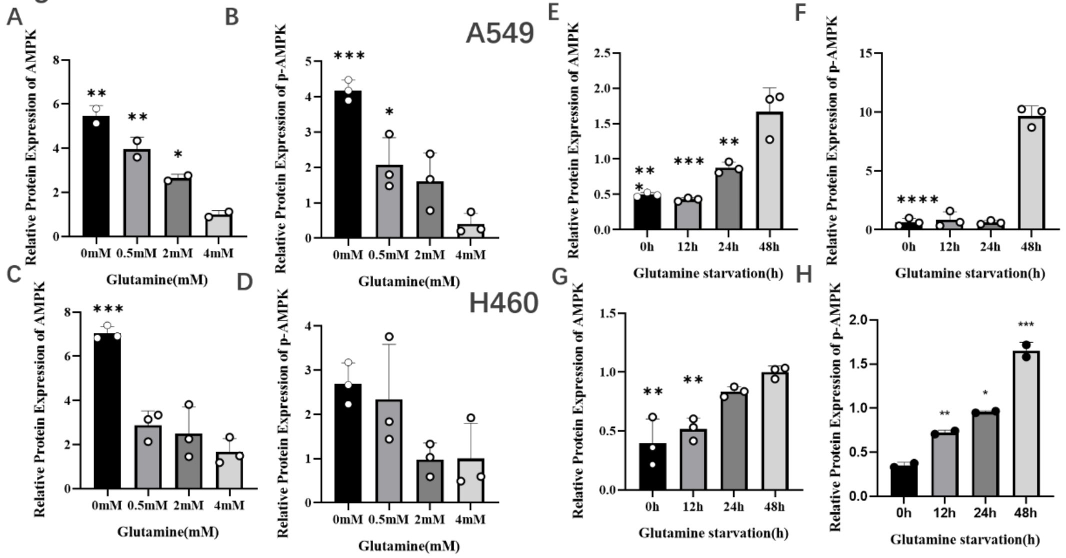

Figure S5. Densitometric quantification of AMPK protein expression under glutamine starvation.

(A-D)Densitometric quantification of AMPK and p-AMPK in NSCLC cells treated with 0, 0.5, 2, and 4 mM glutamine (4 mM as control).

(E-H) Densitometric quantification of AMPK and p-AMPK in NSCLC cells treated with 0, 12, 24, and 48 h complete glutamine starvation. (48h as control).

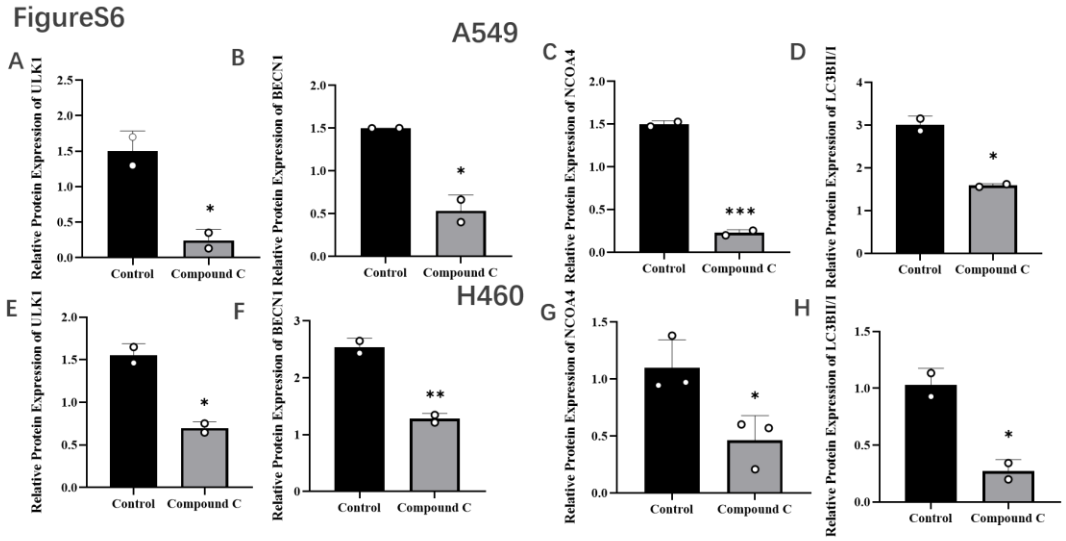

Figure S6. Densitometric quantification of ferritinophagy protein expression in response to AMPK modulation.

(A-H) Densitometric quantification of ULK1, BECN1, NCOA4 and LC3B-II/I in NSCLC cells treated with AMPK inhibitor.

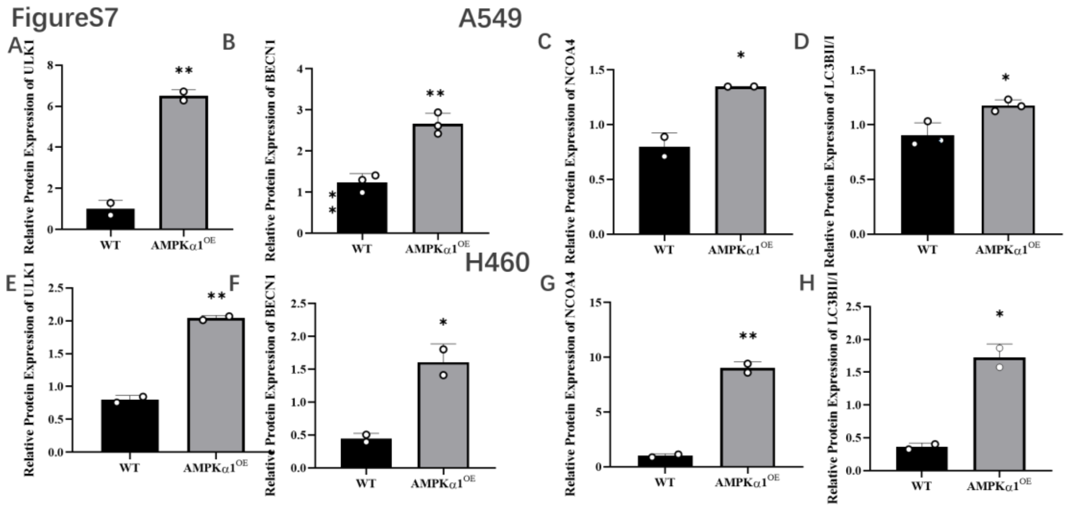

Figure S7. Densitometric quantification of ferritinophagy protein expression in response to AMPK modulation.

(A-H) Densitometric quantification of ULK1, BECN1, NCOA4 and LC3B-II/I in NSCLC cells treated with AMPK overexpression plasmid.

FigureS8

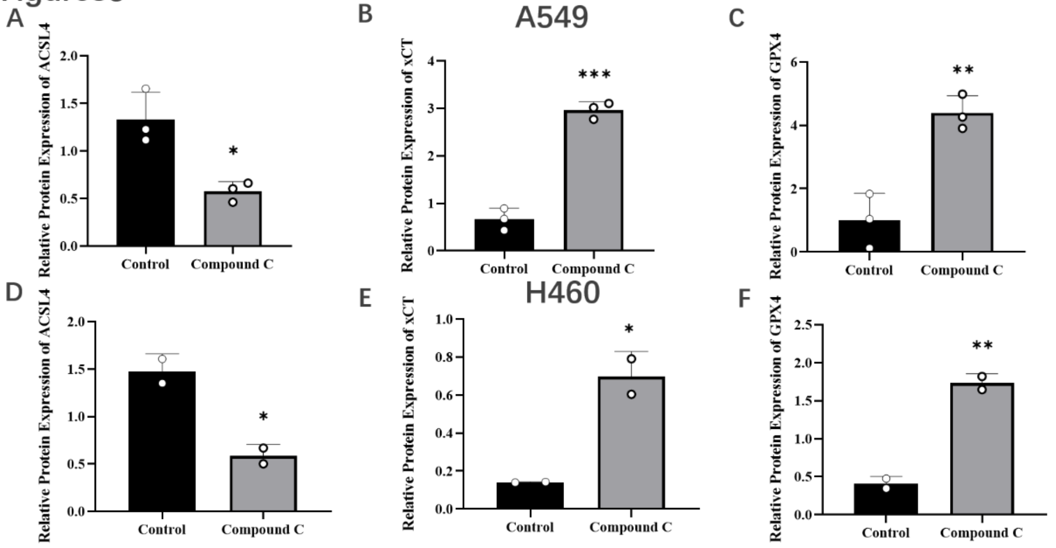

Figure S8. Densitometric quantification of ferroptosis protein expression in response to AMPK modulation.

(A-F) Densitometric quantification of ACSL4, xCT, and GPX4 in NSCLC cells treated with AMPK inhibitor.

FigureS9

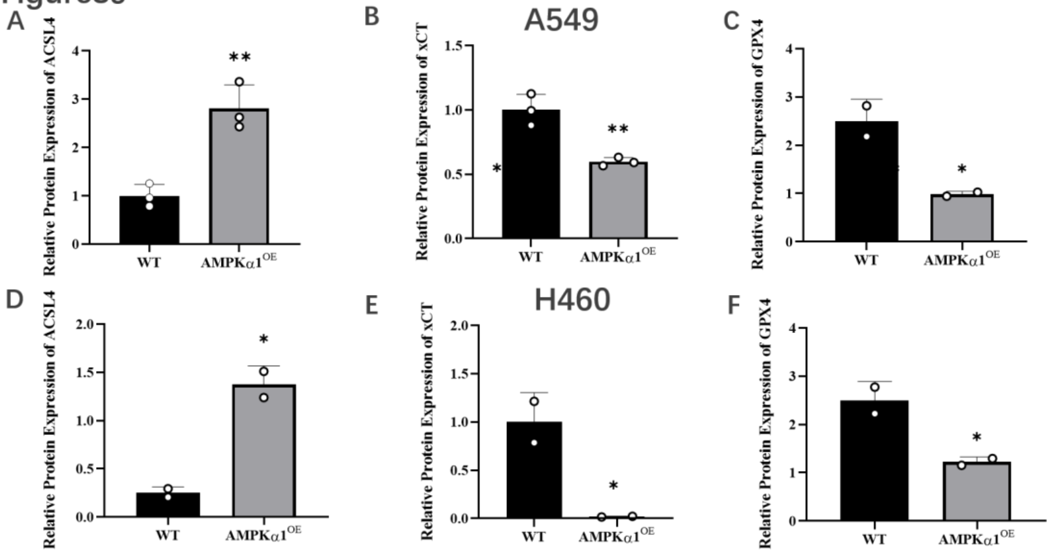

Figure S9. Densitometric quantification of ferroptosis protein expression in

response to AMPK modulation.

(A-F) Densitometric quantification of ACSL4, xCT, and GPX4 in NSCLC cells treated with AMPK overexpression plasmid.

FigureS10

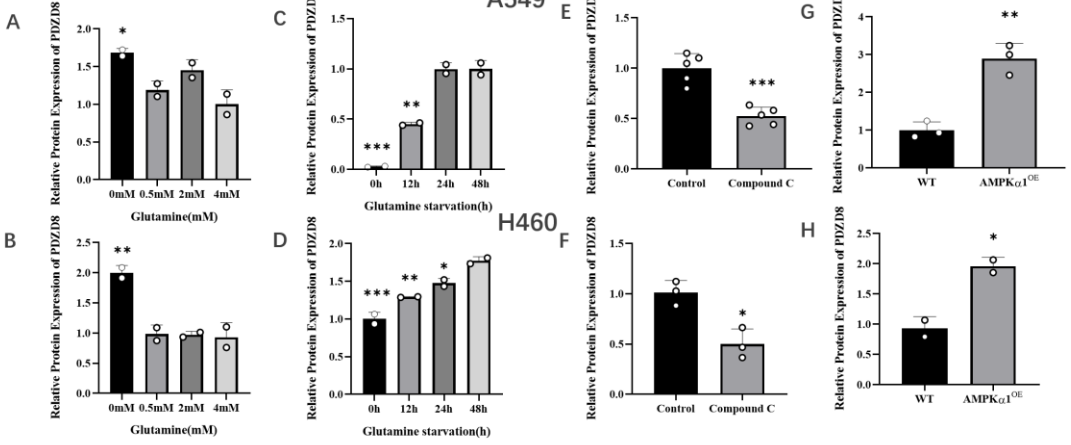

Figure S10. Densitometric quantification of PDZD8 protein expression.

(A-B) Densitometric quantification of PDZD8 in NSCLC cells treated with 0, 0.5, 2, and 4 mM glutamine (4 mM as control).

(C-D) Densitometric quantification of PDZD8 in NSCLC cells treated with0, 12, 24, and 48 h complete glutamine starvation. (48h as control).

(E-F) Densitometric quantification of PDZD8 in NSCLC cells treated with AMPK inhibitor.

(G-H) Densitometric quantification of ACSL4, xCT, and GPX4 in NSCLC cells treated with AMPK overexpression plasmid.

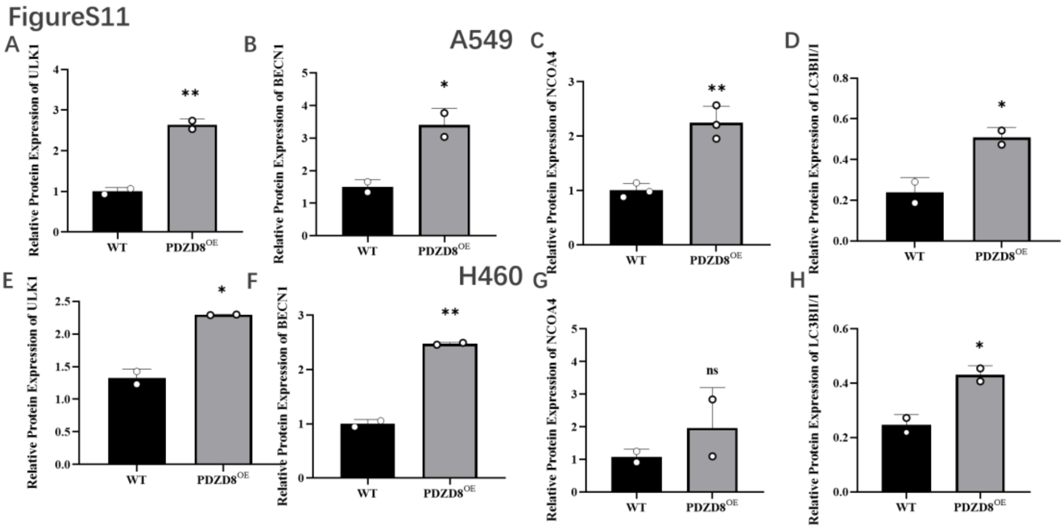

Figure S11. Densitometric quantification of ferritinophagy protein expression in response to PDZD8 modulation.

(A-H) Densitometric quantification of ULK1, BECN1, NCOA4 and LC3B-II/I in NSCLC cells treated with PDZD8 overexpression plasmid.

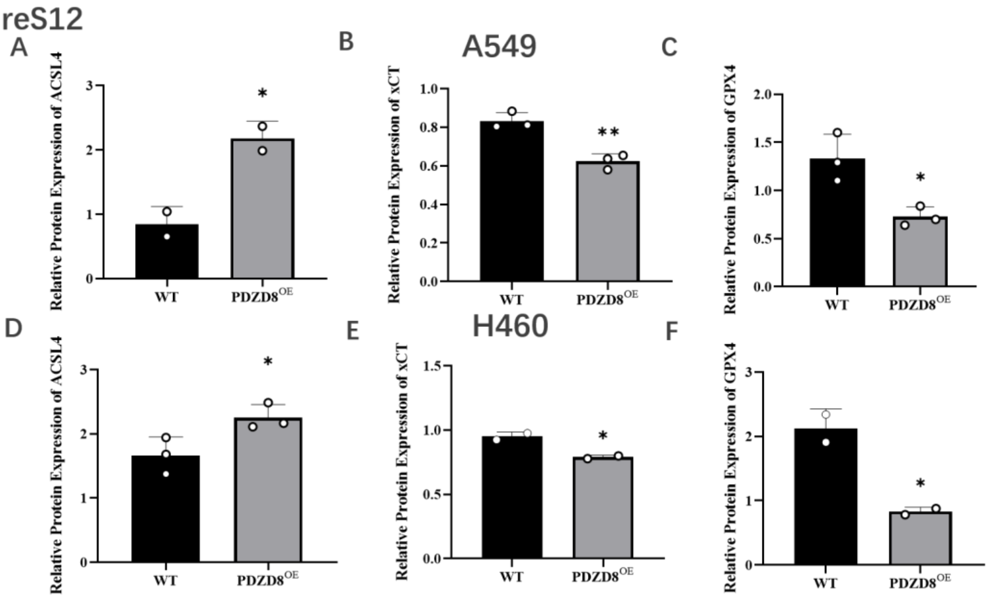

Figure S12. Densitometric quantification of ferritinophagy protein expression in response to PDZD8 modulation.

(A-F) Densitometric quantification of ACSL4, xCT, and GPX4 in NSCLC cells treated with AMPK overexpression plasmid.

FigureS13

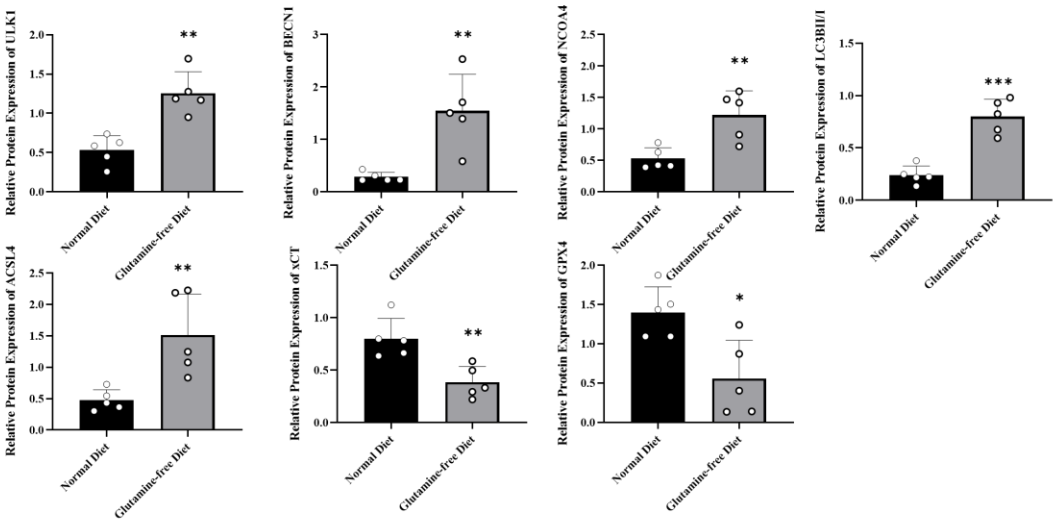

Figure S13. Densitometric quantification of ferritinophagy and ferroptosis protein expression in mice model.

Densitometric quantification of ULK1, BECN1, NCOA4 , LC3B-II/I ,ACSL4, xCT, and GPX4 in NSCLC cells treated with glutamine-free diet.(Normal diet as control)
