# Supplementary material for: Glutamine Starvation Induces Ferroptosis in NSCLC via AMPK/PDZD8-Mediated Ferritinophagy
Source: Nutrients. 2026 May 18;18(10):1596. doi: 10.3390/nu18101596 (PMC13209610; doi:10.3390/nu18101596)

Fig.Supplement

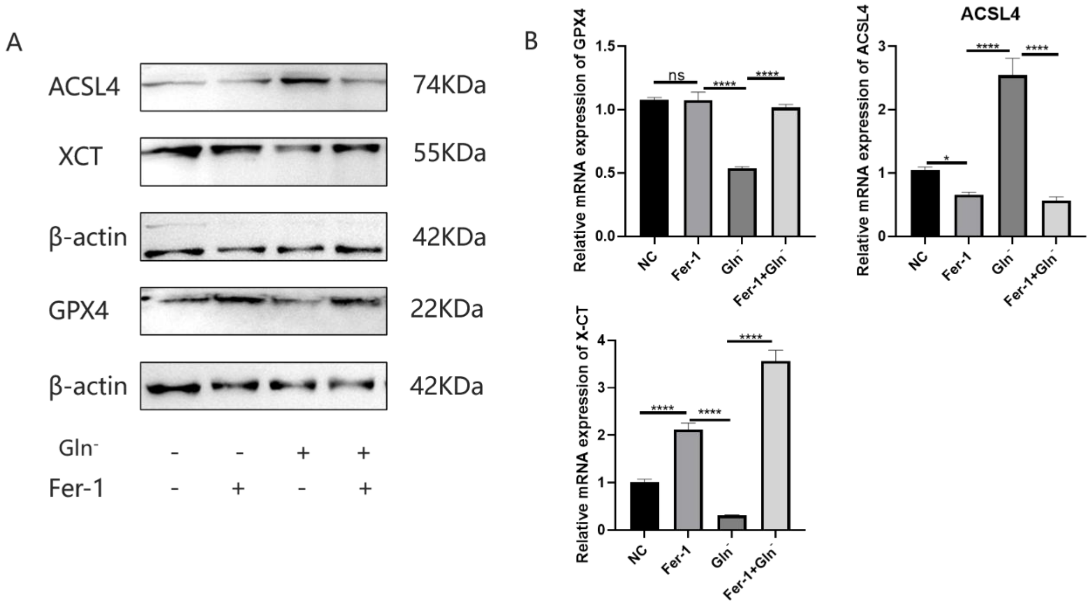

(A) The expression levels of the ferroptosis-related proteins ACSL4 (74 kDa), XCT (55 kDa), and GPX4 (22 kDa) were assessed in A549 cells under conditions of glutamine deprivation (Gln-) and/or treatment with the ferroptosis inhibitor Ferrostatin-1 (Fer-1). β-actin (42 kDa) served as a loading control. Blots are representative of at least three independent experiments.

(B) The mRNA expression levels of *GPX4*, *XCT*, and *ACSL4*were quantified in A549 cells under the indicated treatment conditions. Data are presented as mean ± s.e.m. Statistical significance is indicated (**P<0.01,***P<0.001,*****P<0.0001, ns, not significant). Gln-, glutamine deprivation; Fer-1, Ferrostatin-1.

Original data
(supplementary experiment)

Fer-1 A549-ACSL4

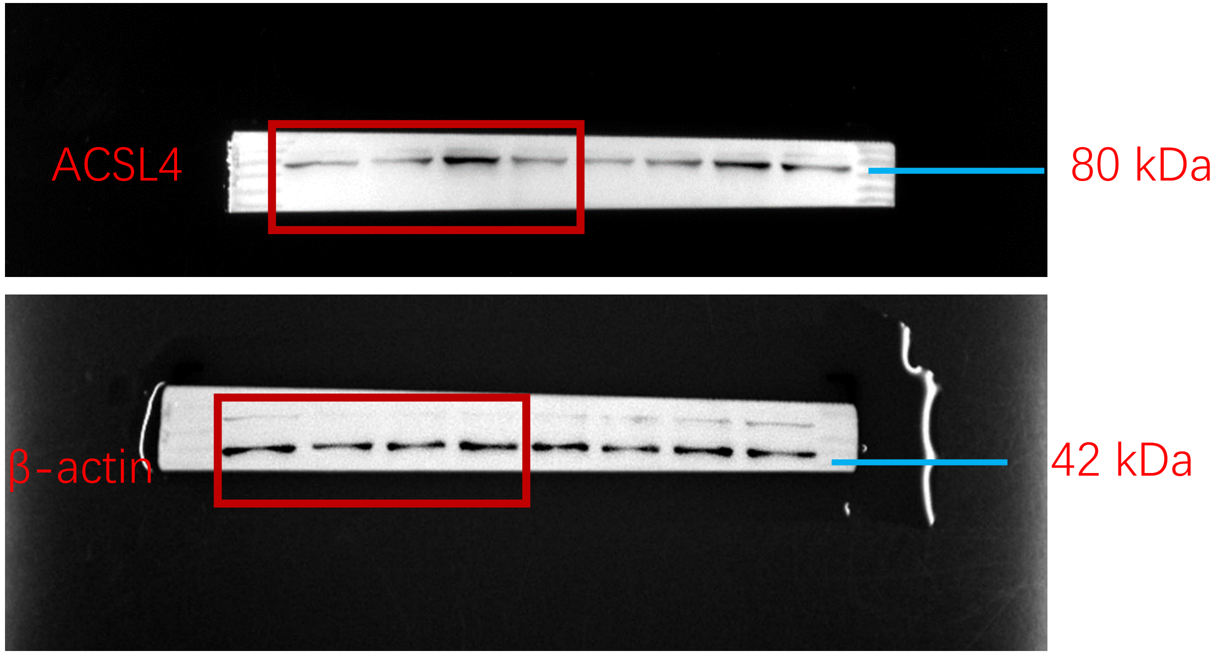

Fer-1 A549-X-CT

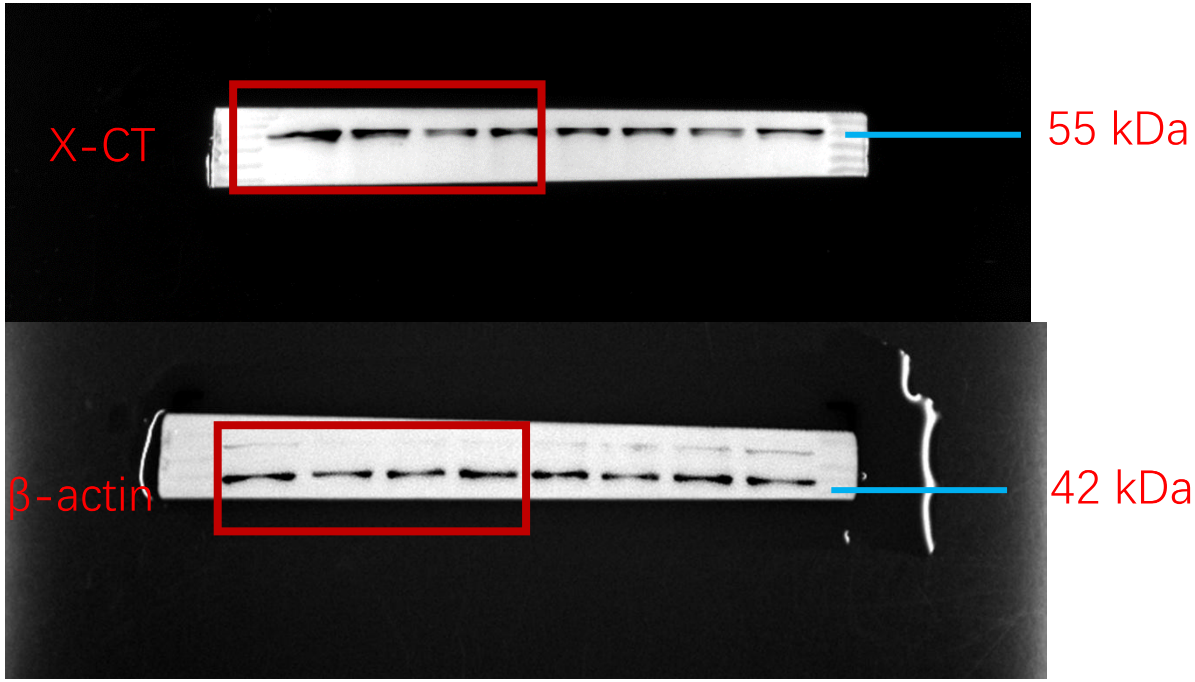

Fer-1 A549-GPX4

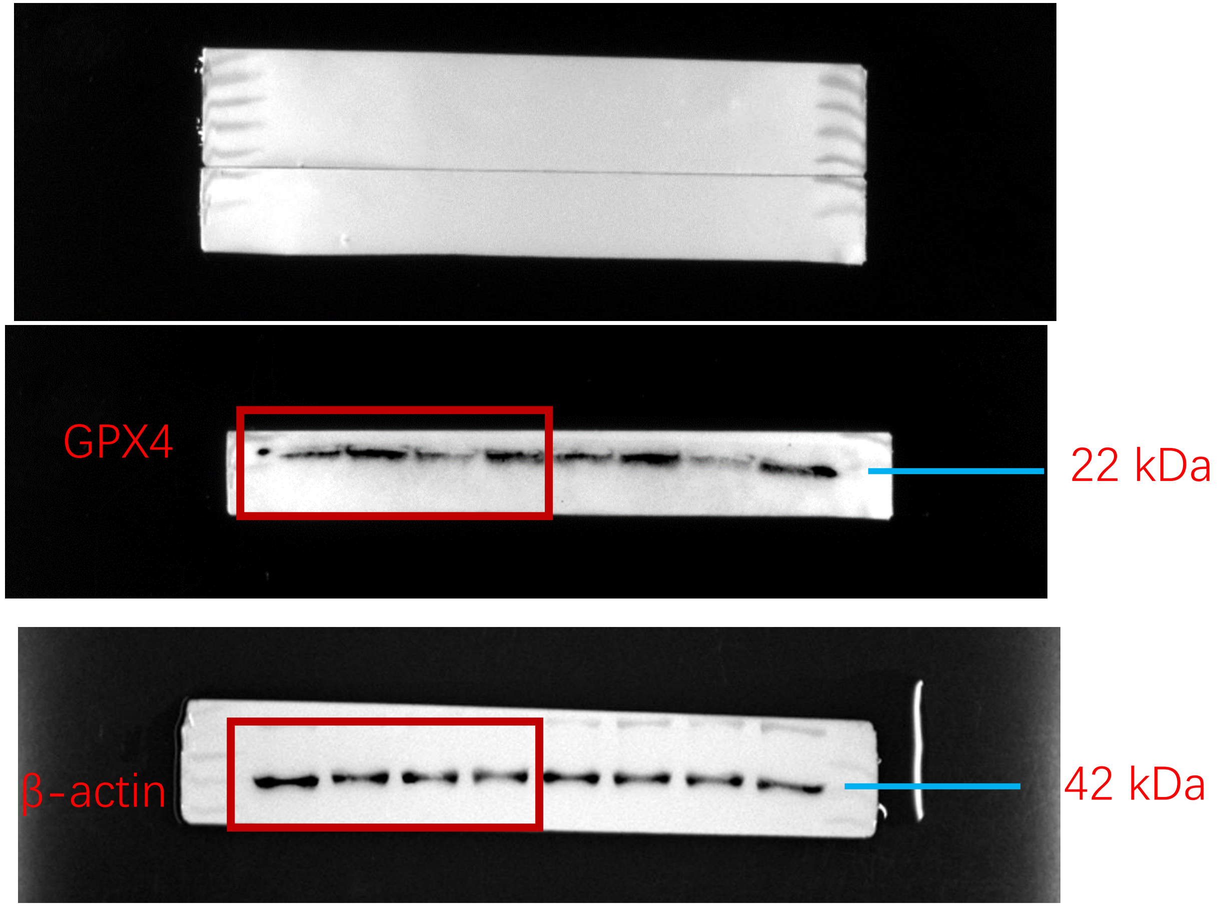

FIG4C-H460 PAMPK

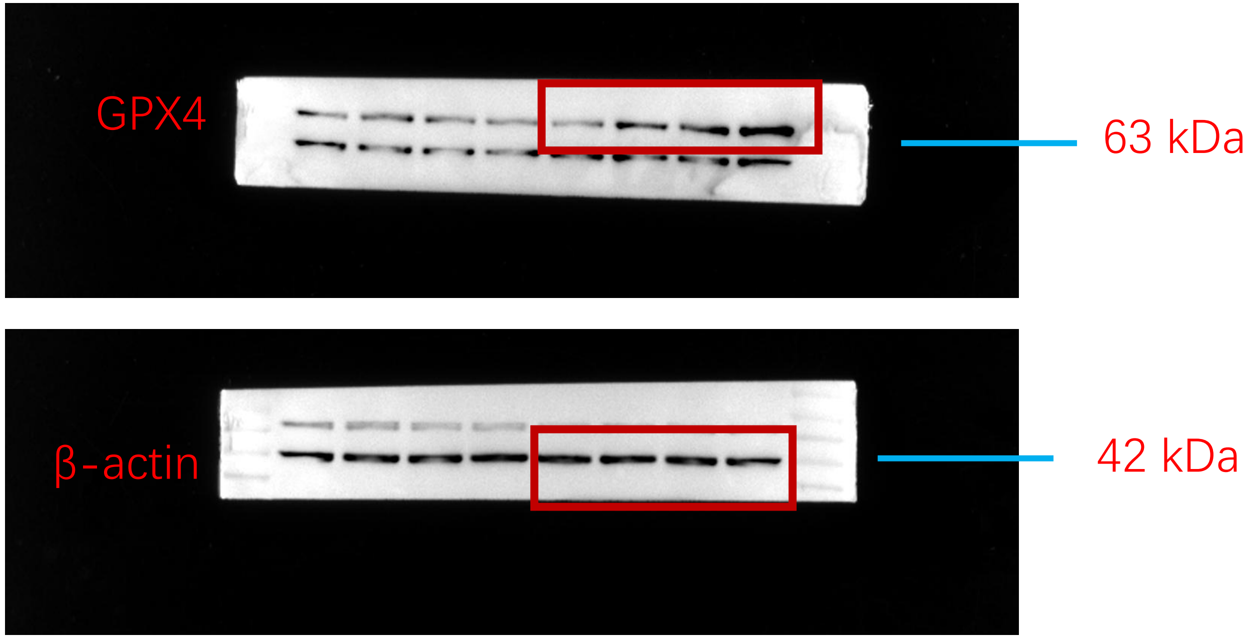

Supplement: Supplementary file 1 [file nutrients-18-01596-s001.zip › File S2.pdf]
