# Supplementary material for: Glutamine Starvation Induces Ferroptosis in NSCLC via AMPK/PDZD8-Mediated Ferritinophagy
Source: Nutrients. 2026 May 18;18(10):1596. doi: 10.3390/nu18101596 (PMC13209610; doi:10.3390/nu18101596)

original images

Figure 2

Figure 2A.A549-gapdh

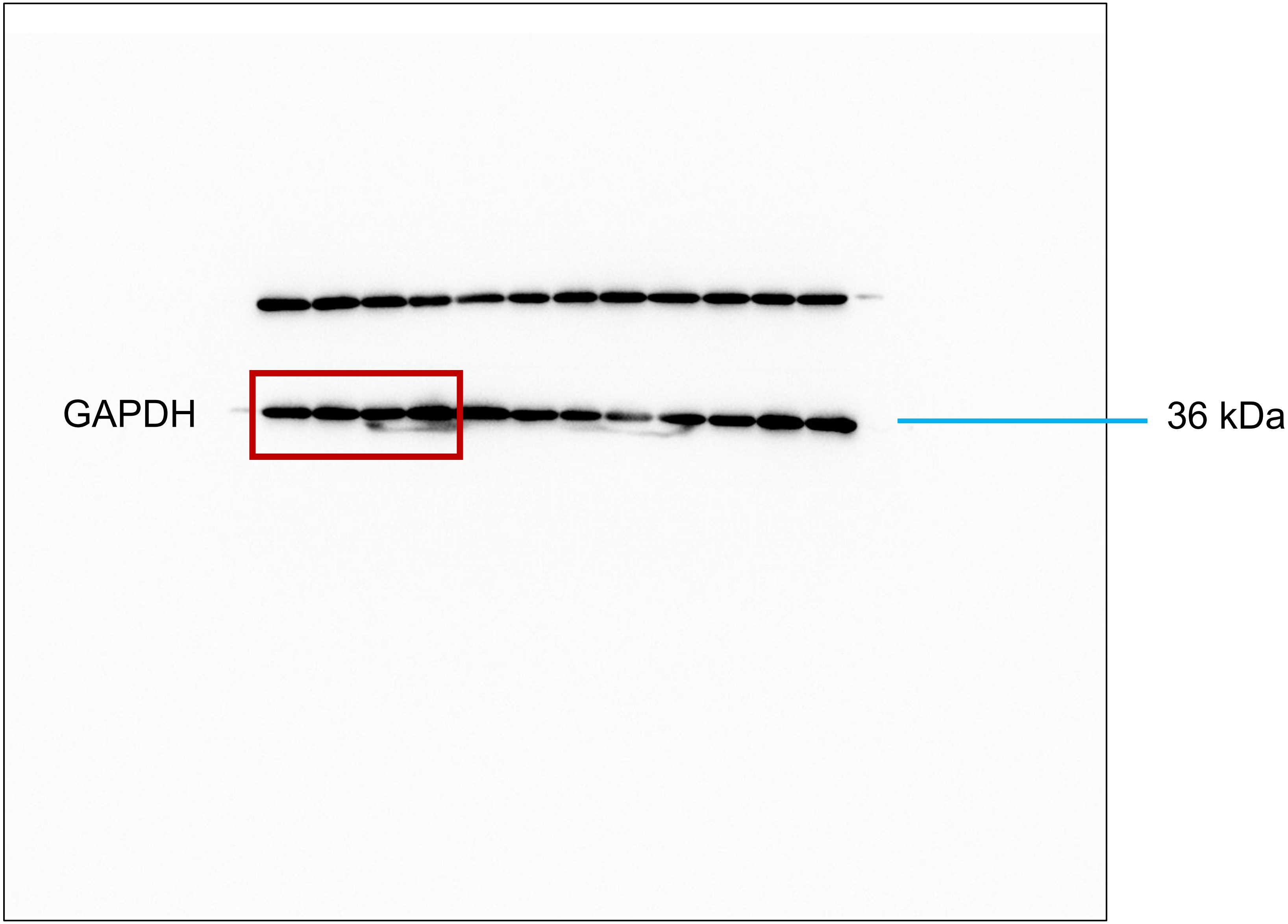

Figure 2A.A549-BECN1

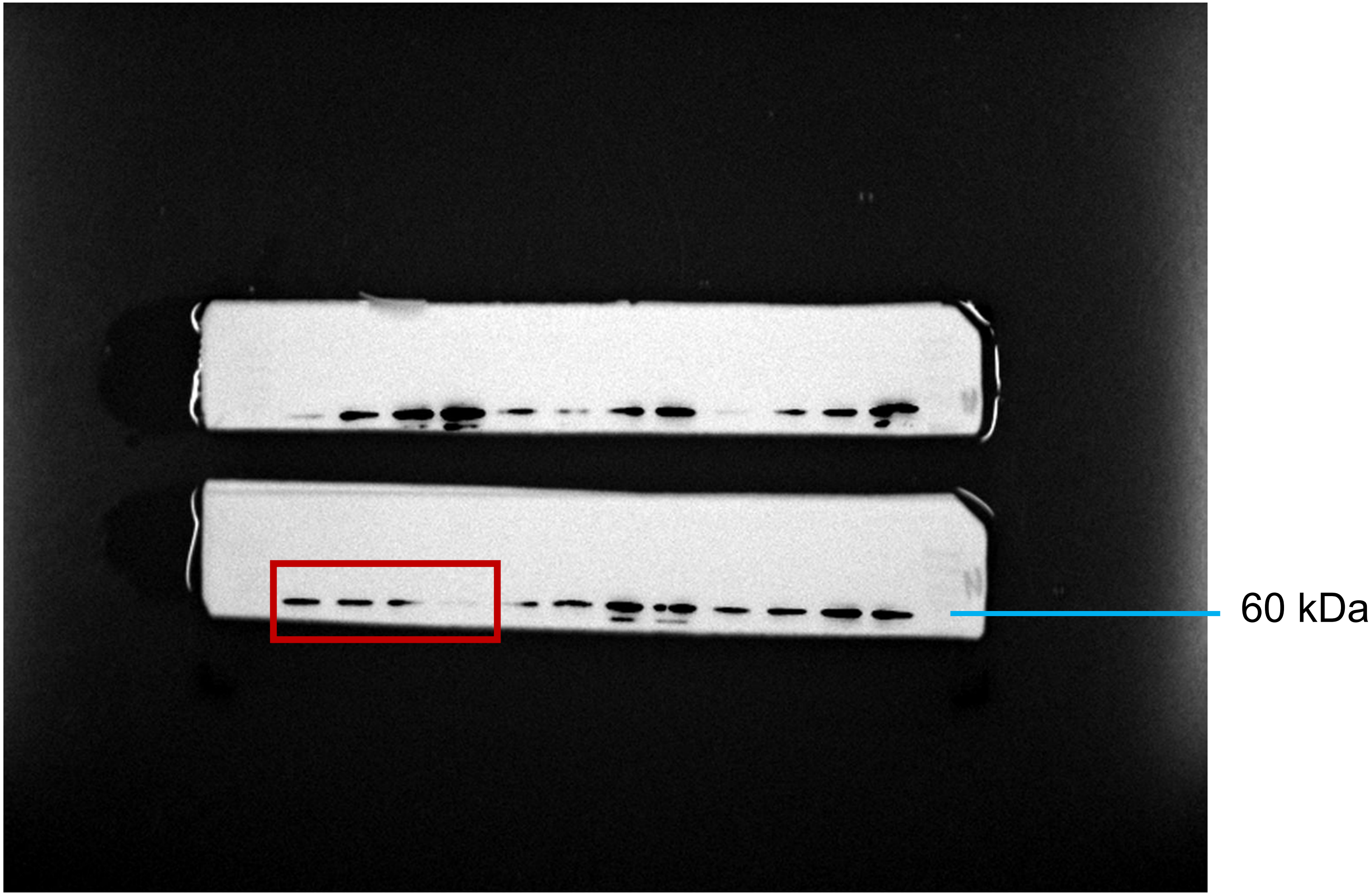

Figure 2A.A549-LC3B

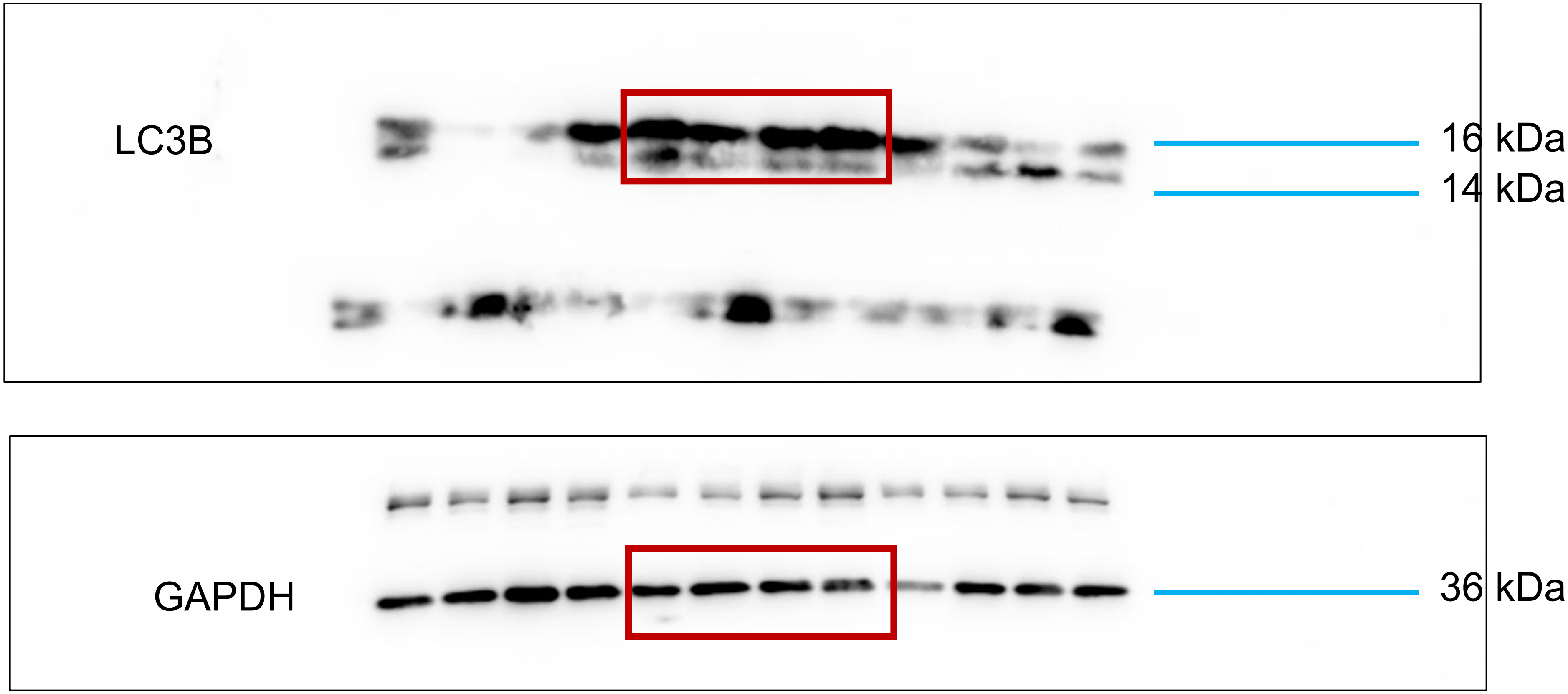

Figure 2A.A549-NCOA4

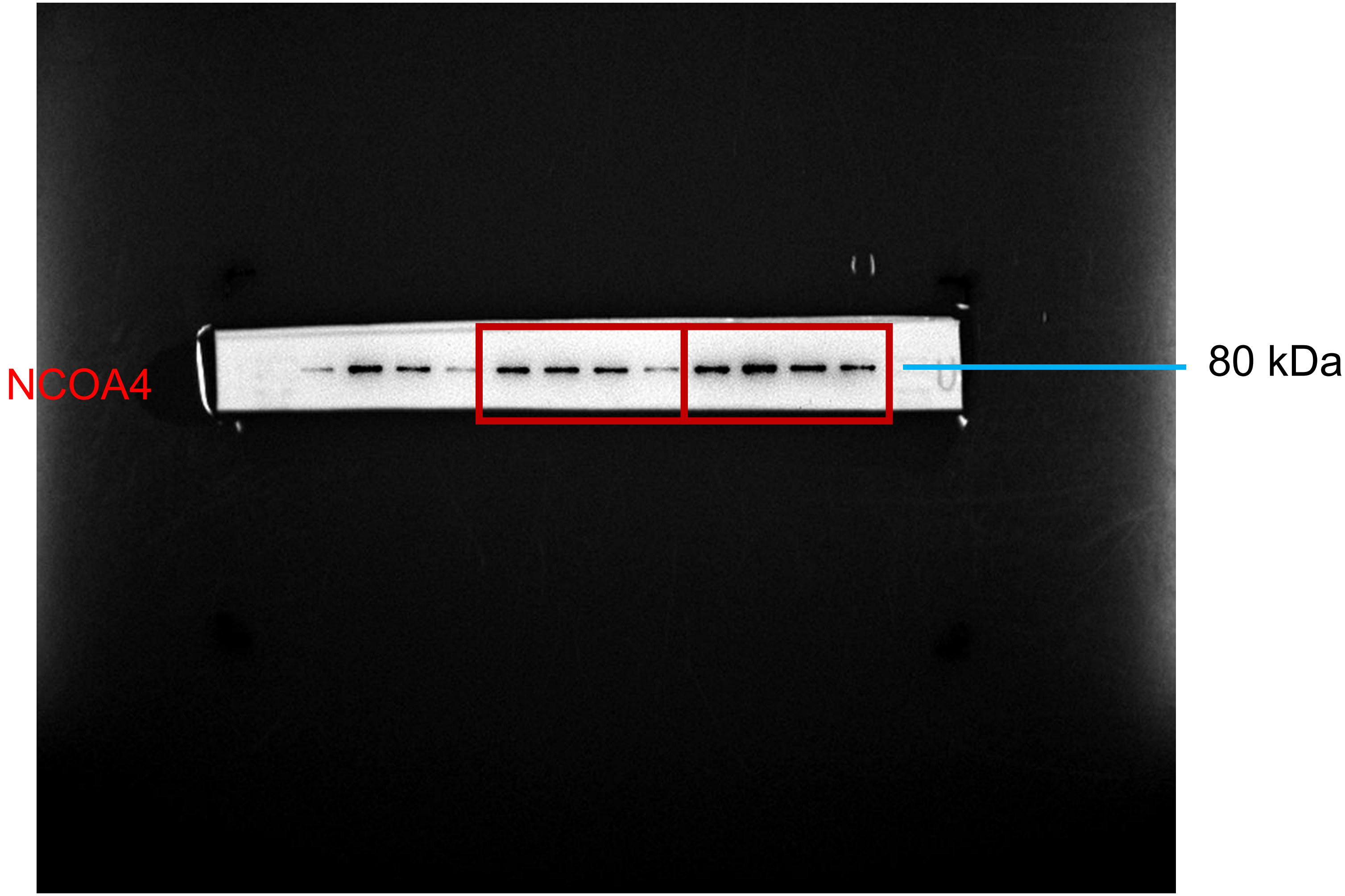

Figure 2A.A549-ULK1

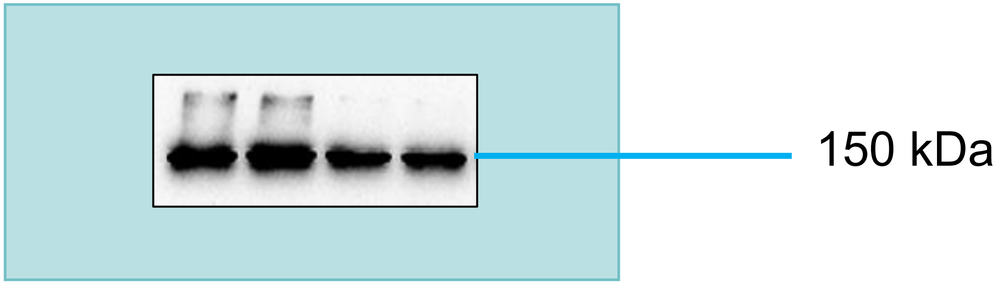

Figure 2A.H460-ULK1

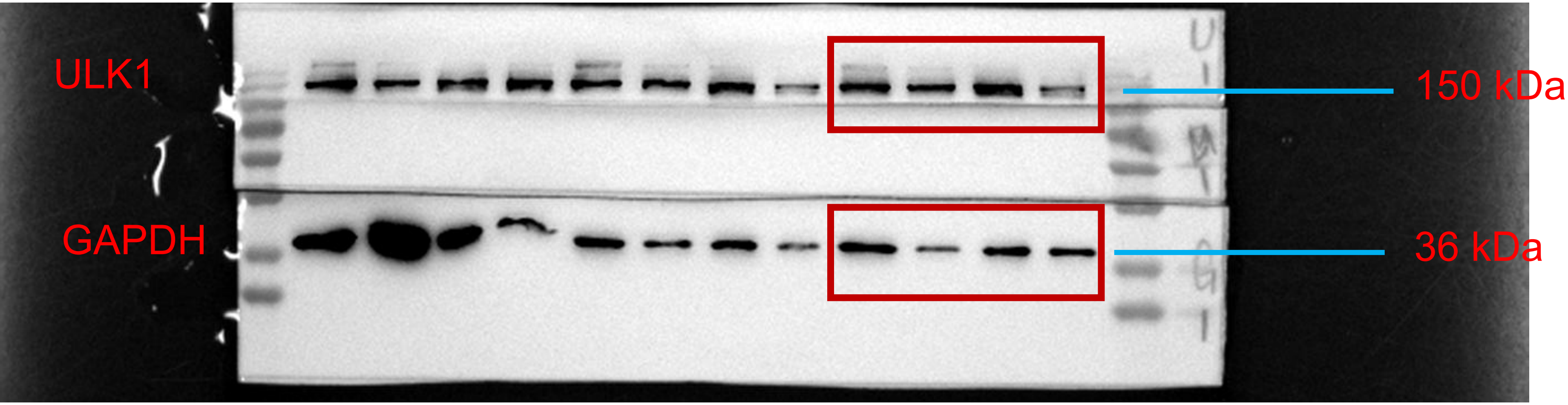

Figure 2A.H460-LC3B

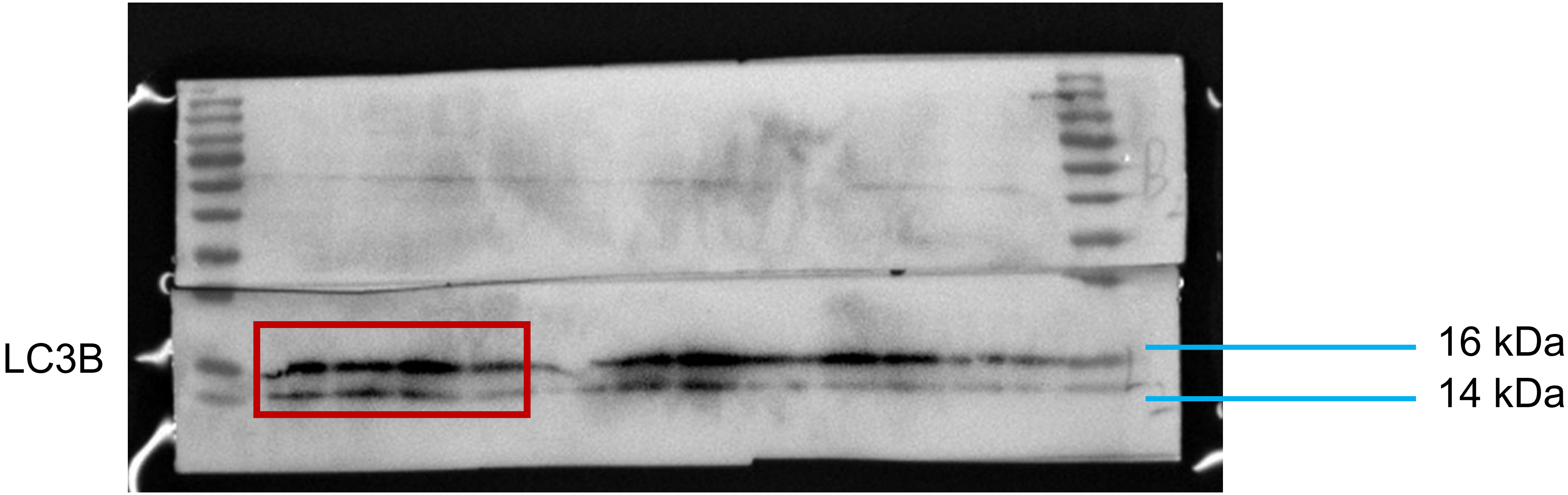

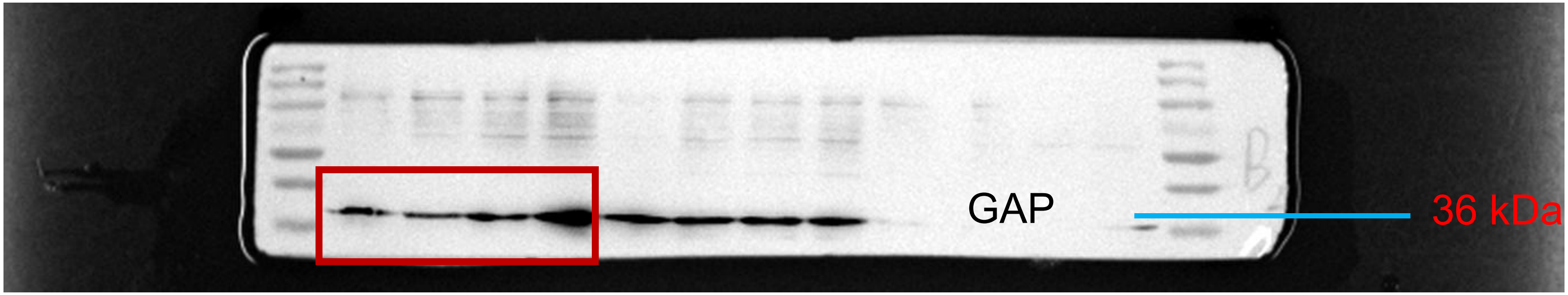

Figure 2A.H460-BECN1

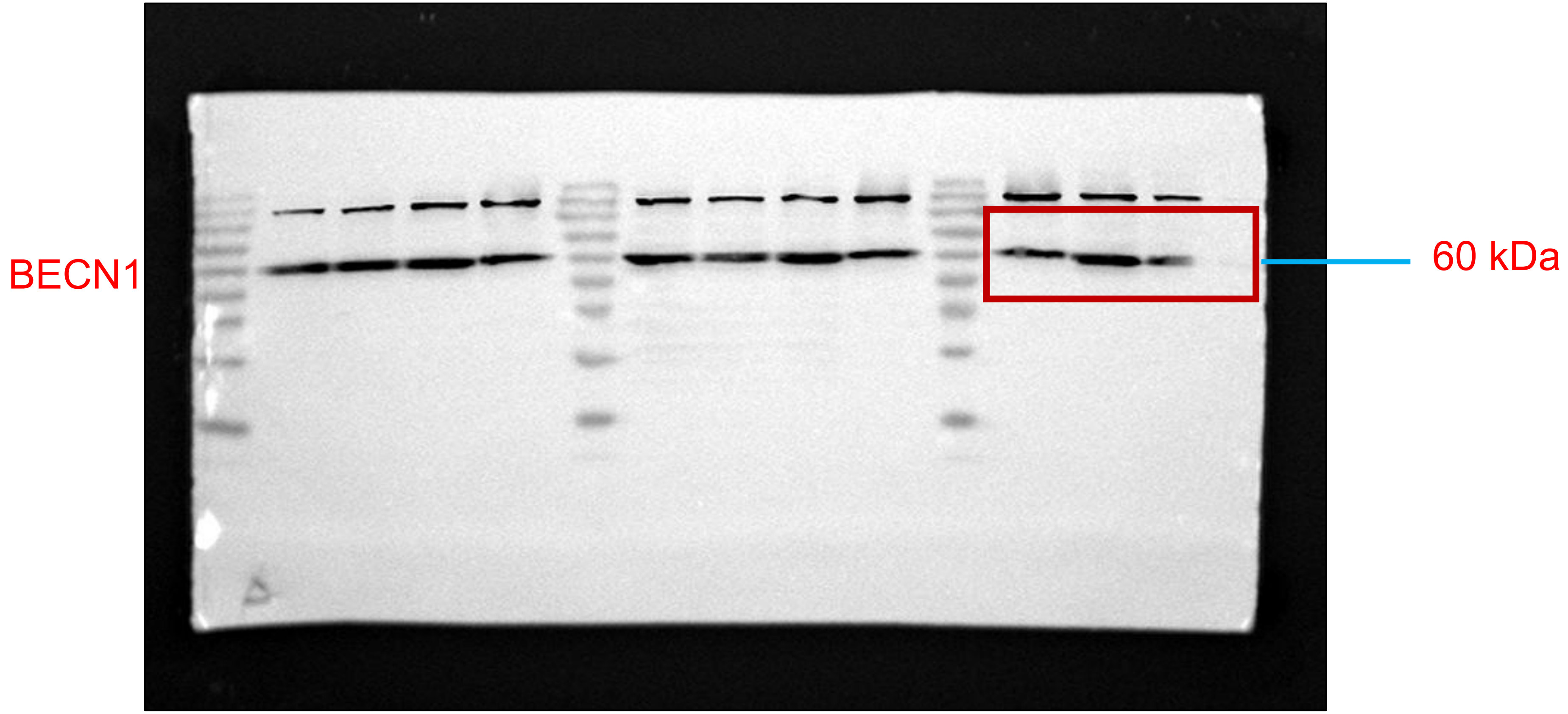

Figure 2AH460-NCOA4
24.12.9

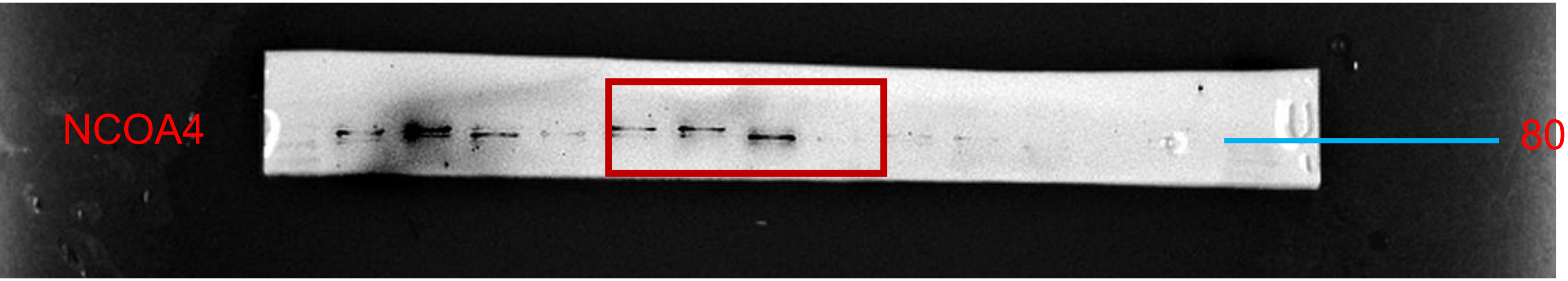

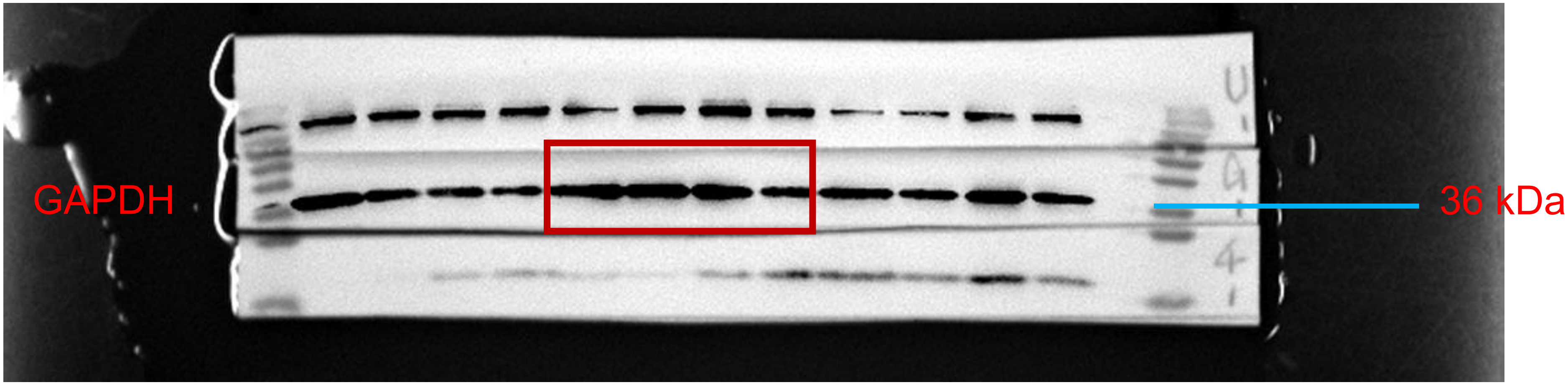

Figure 2C.A549-ULK1

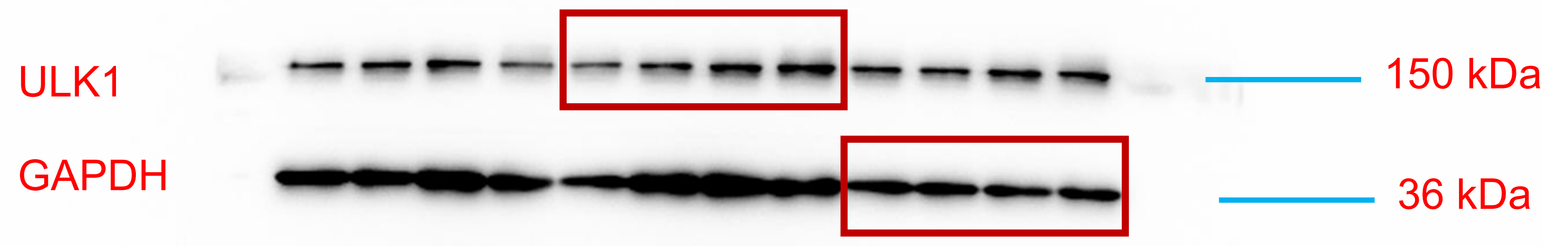

Figure 2C.A549-BECN1

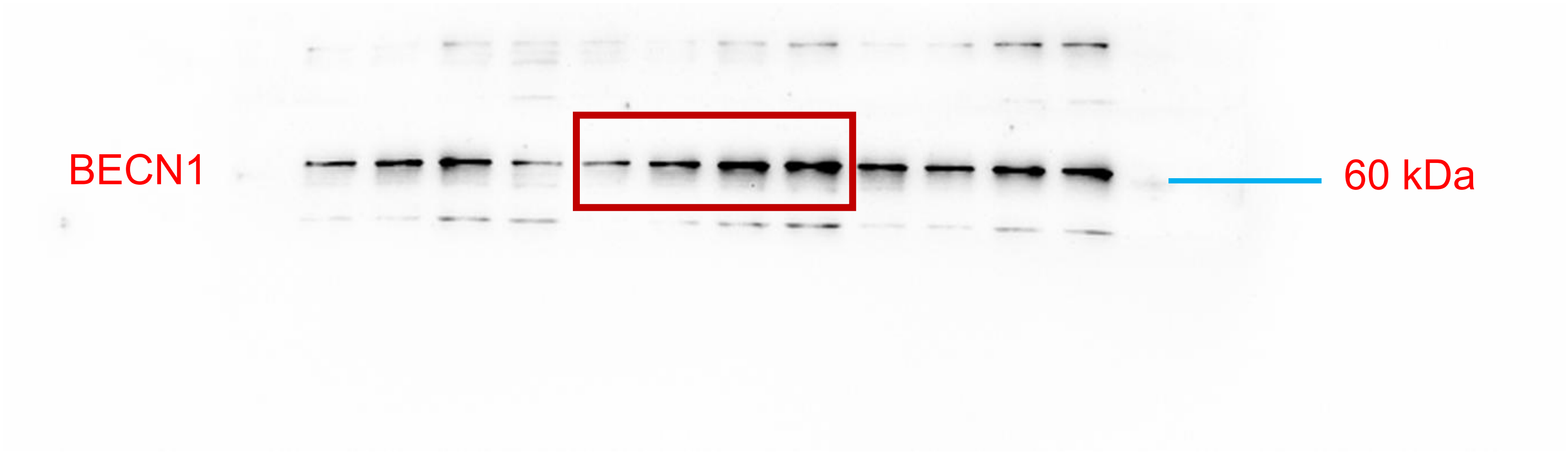

Figure 2C.A549-NCOA4

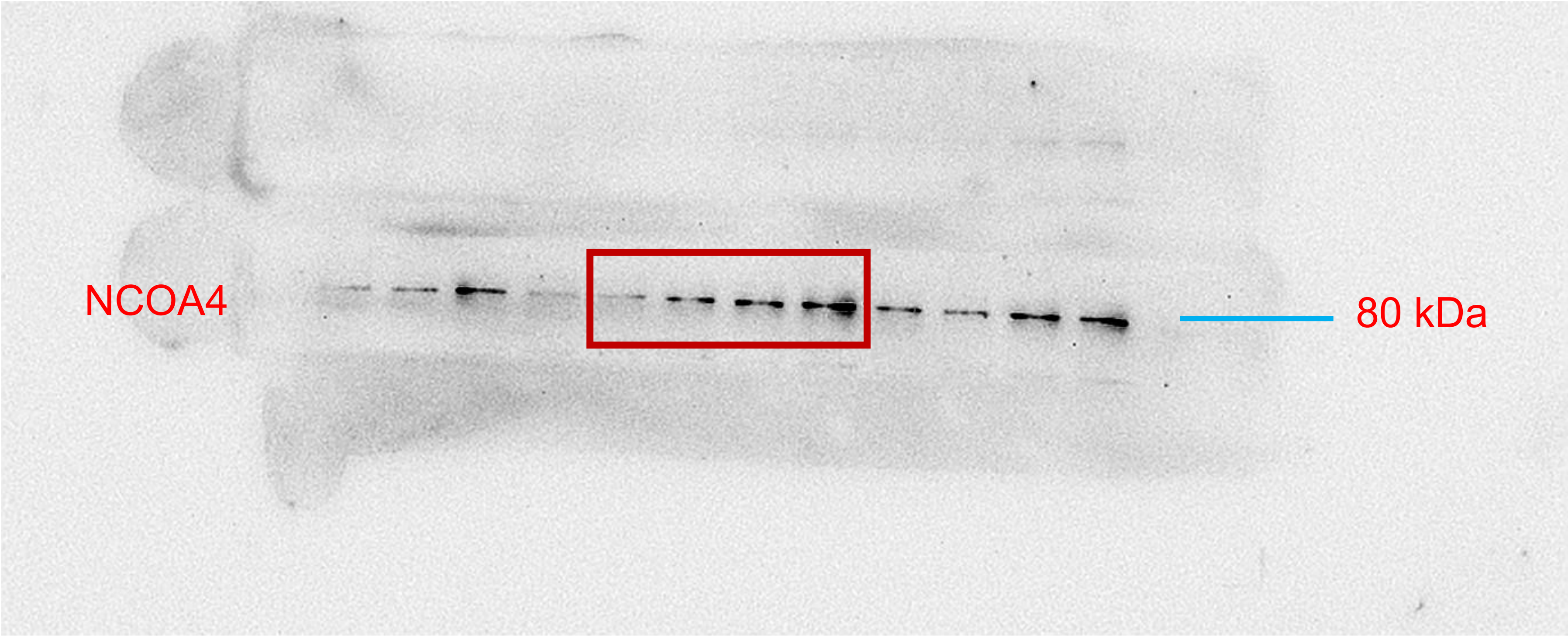

Figure 2C.A549-LC3B

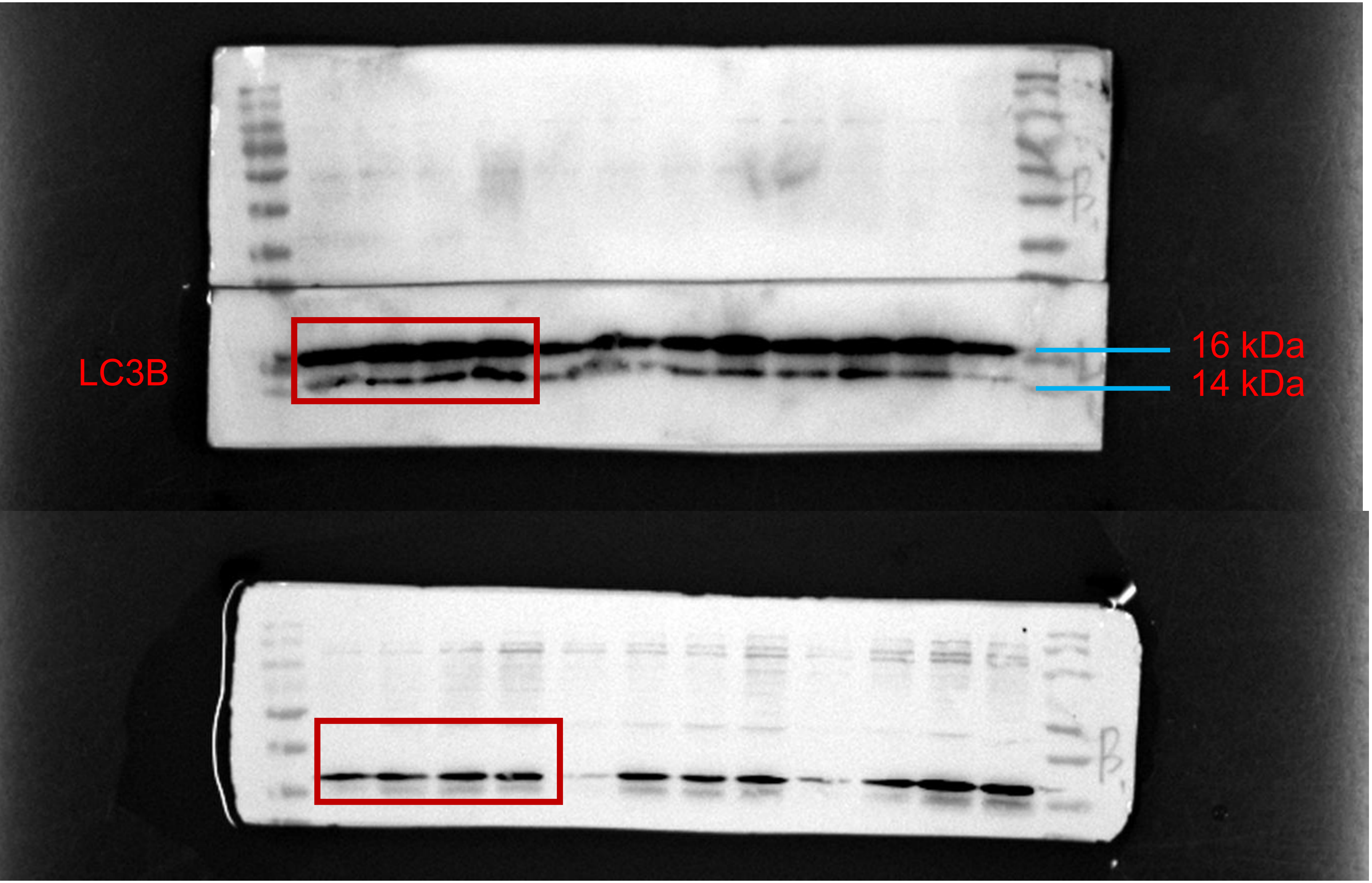

Figure 2C.H460-ULK1

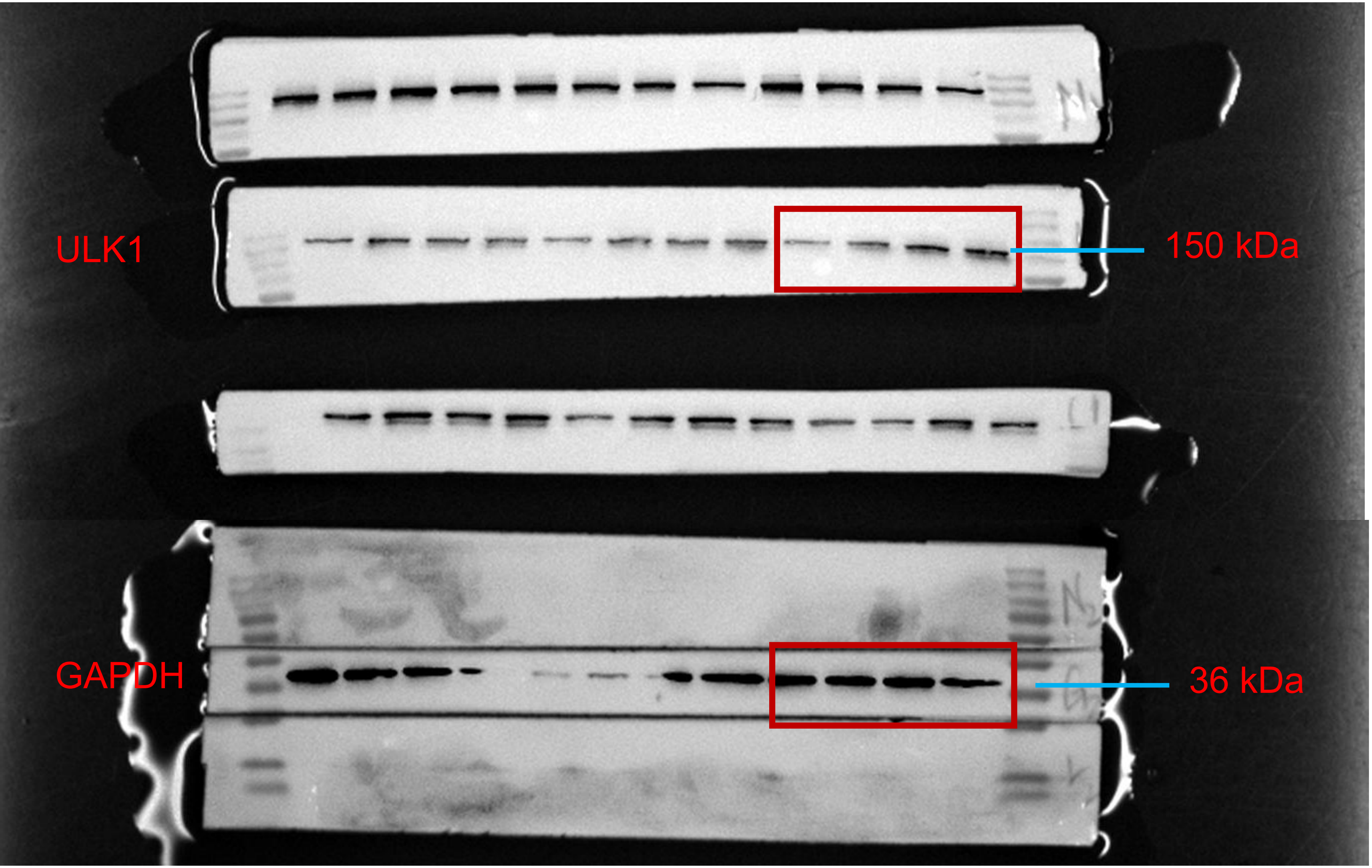

Figure 2C.H460-NCOA4

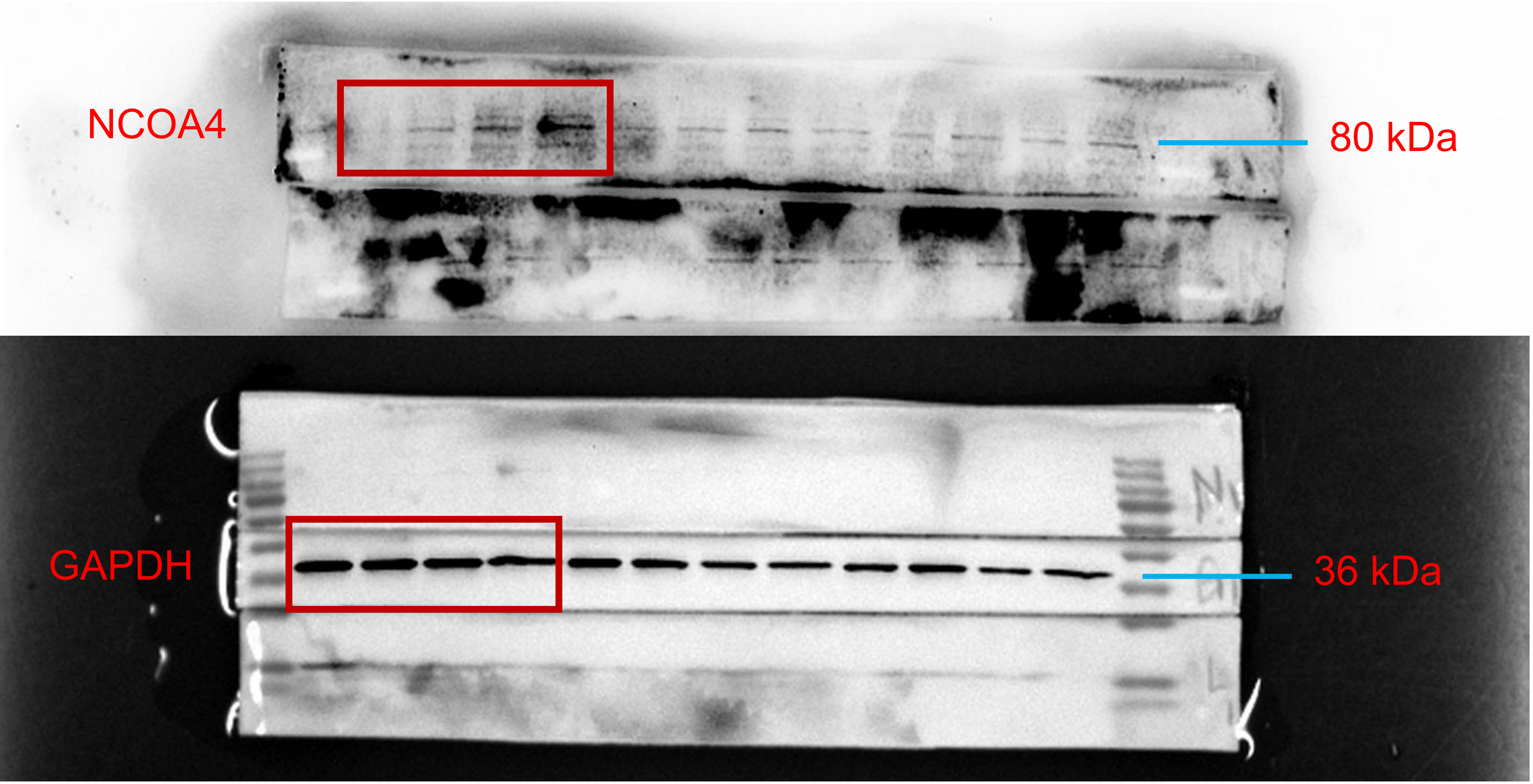

Figure 2A.H460-BECN1

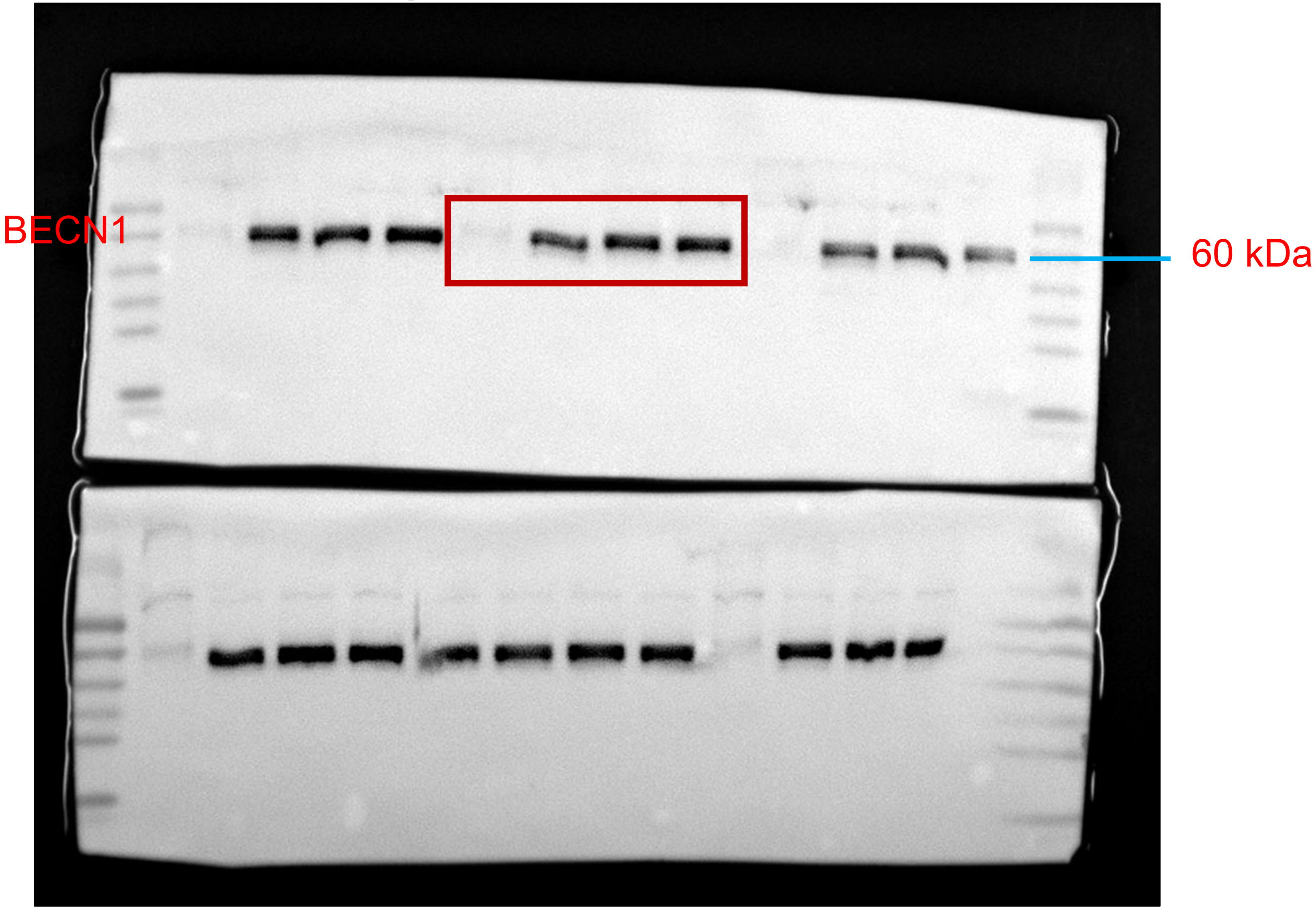

H460-LC3B

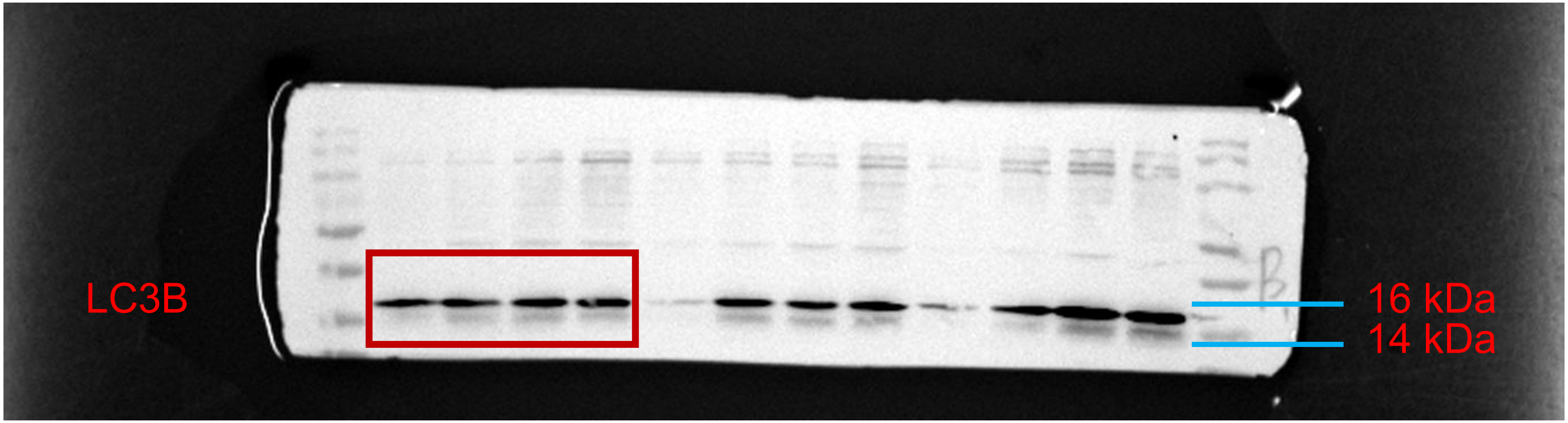

Figure 3

Figure 3A.A549-ACSL4

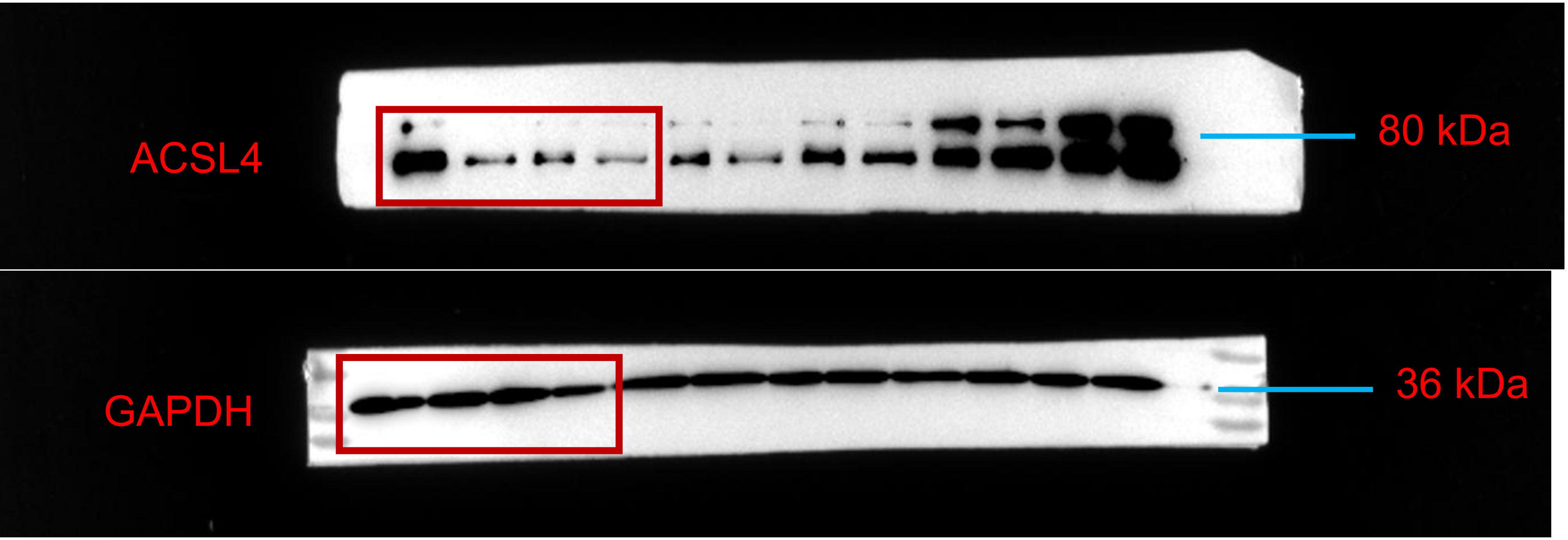

Figure 3A.A549-GPX4

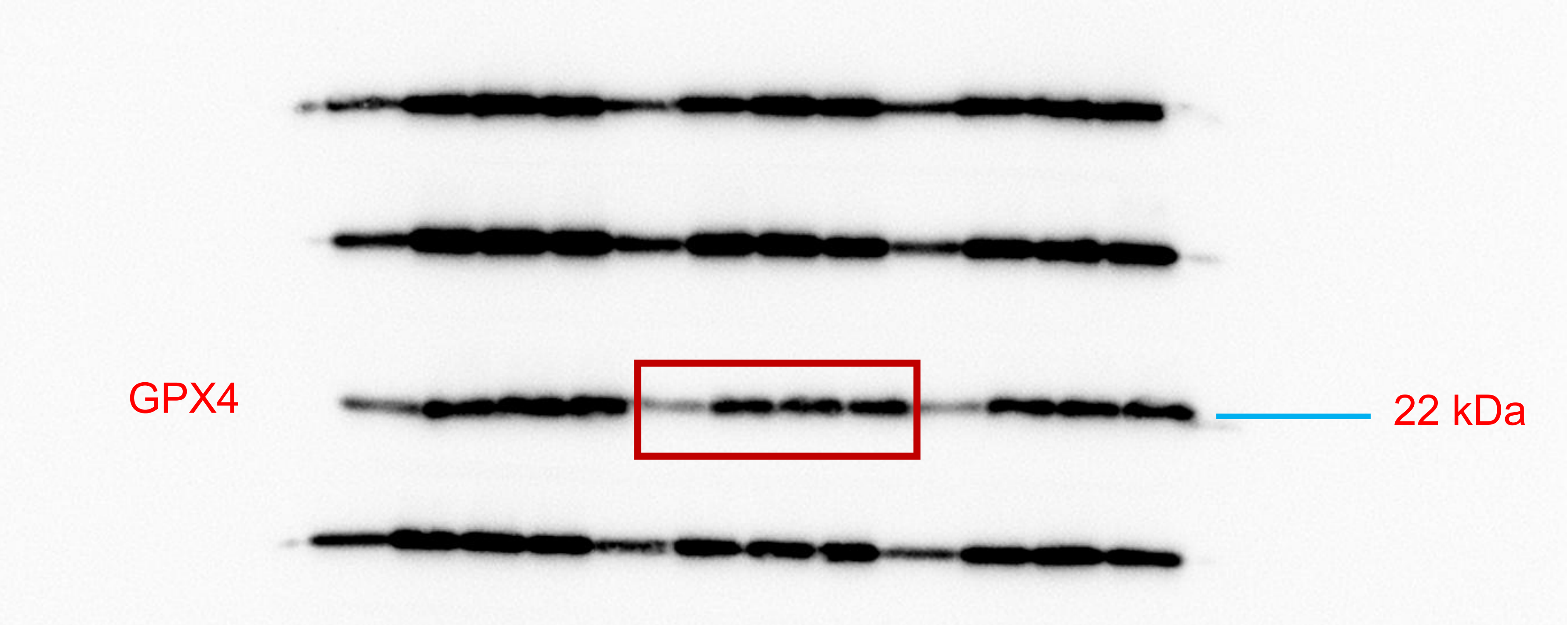

Figure 3A.A549-XCT

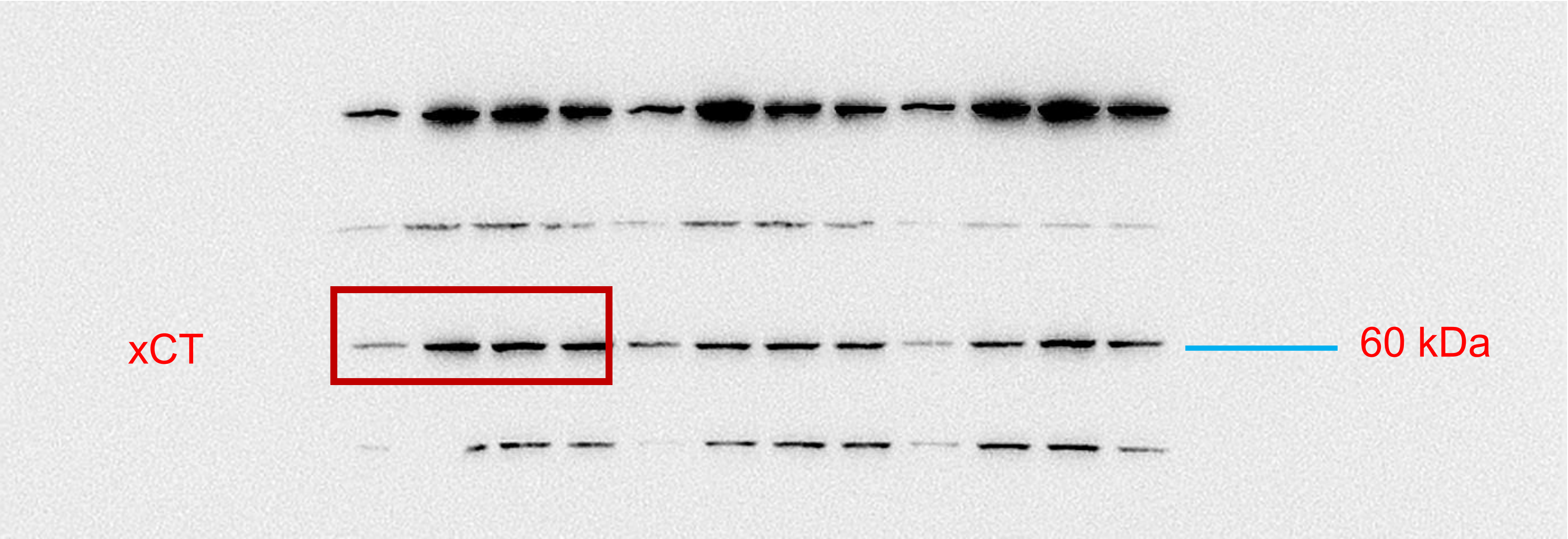

Figure 3A.H460-GPX4

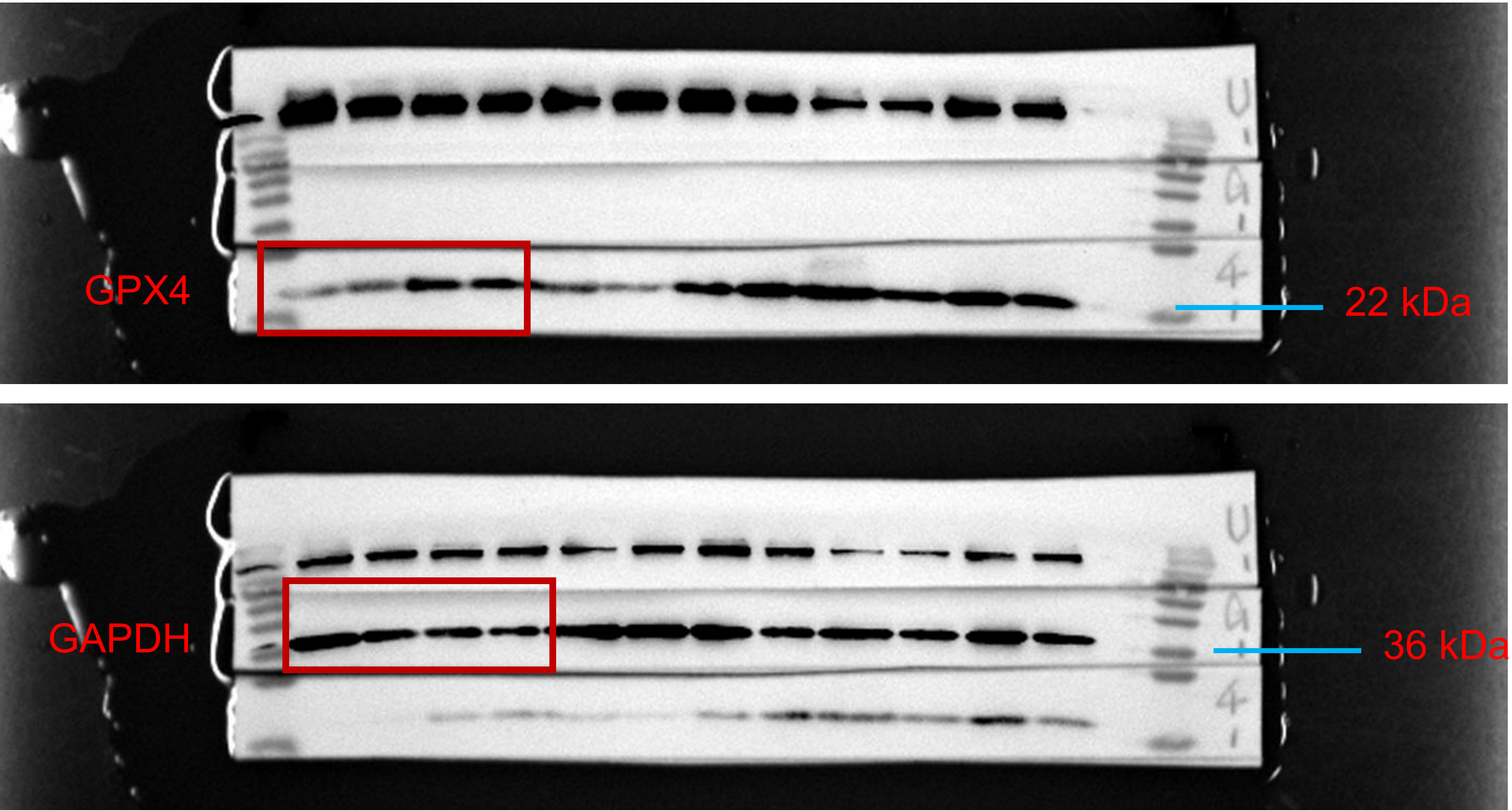

Figure 3A.H460-ACSL4

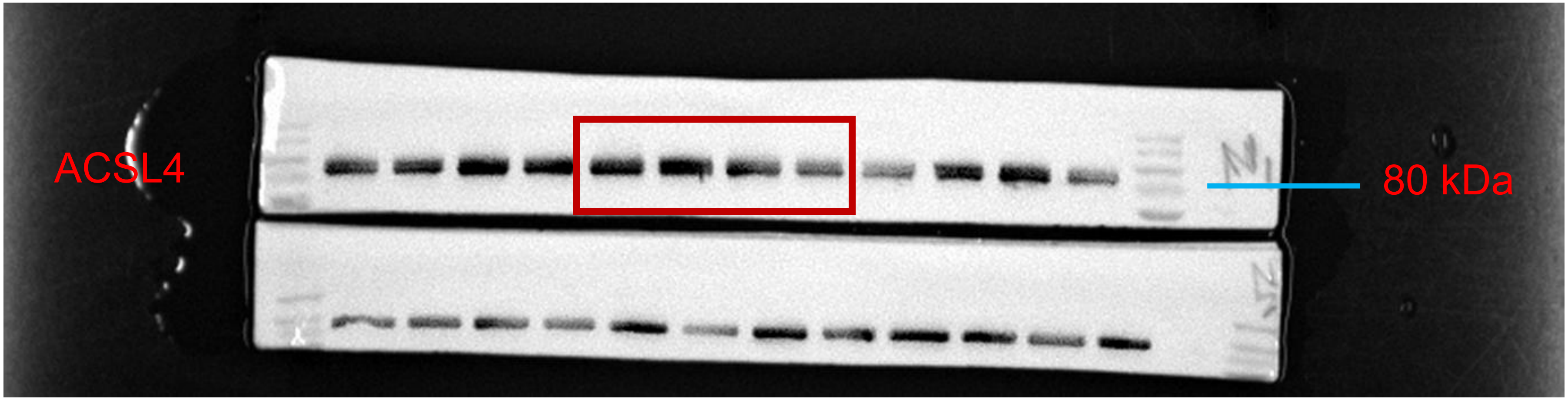

Figure 3A.H460-XCT

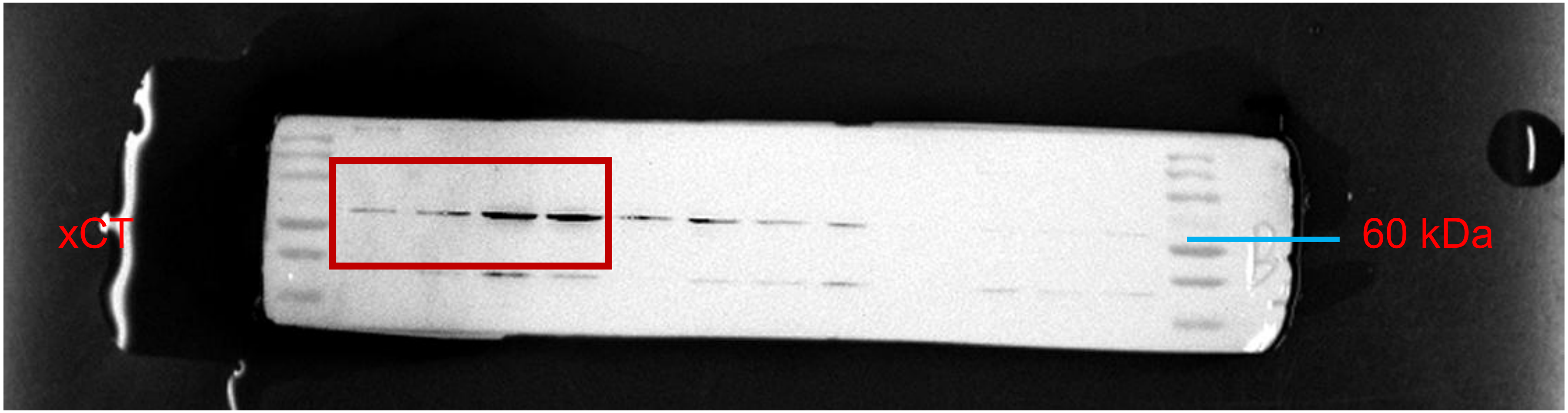

Figure 3C.A549-ACSL4

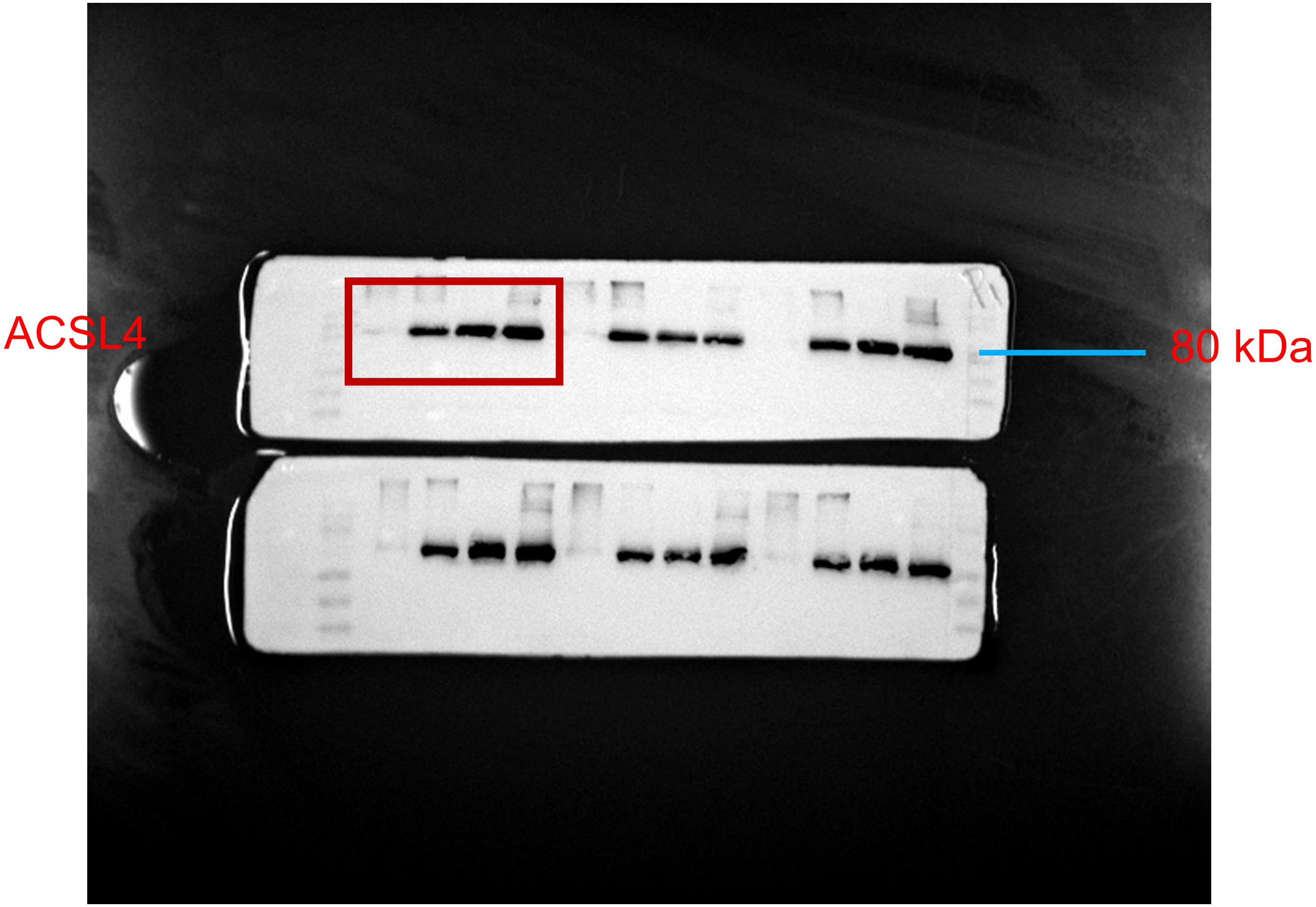

Figure 3C.A549-GPX4

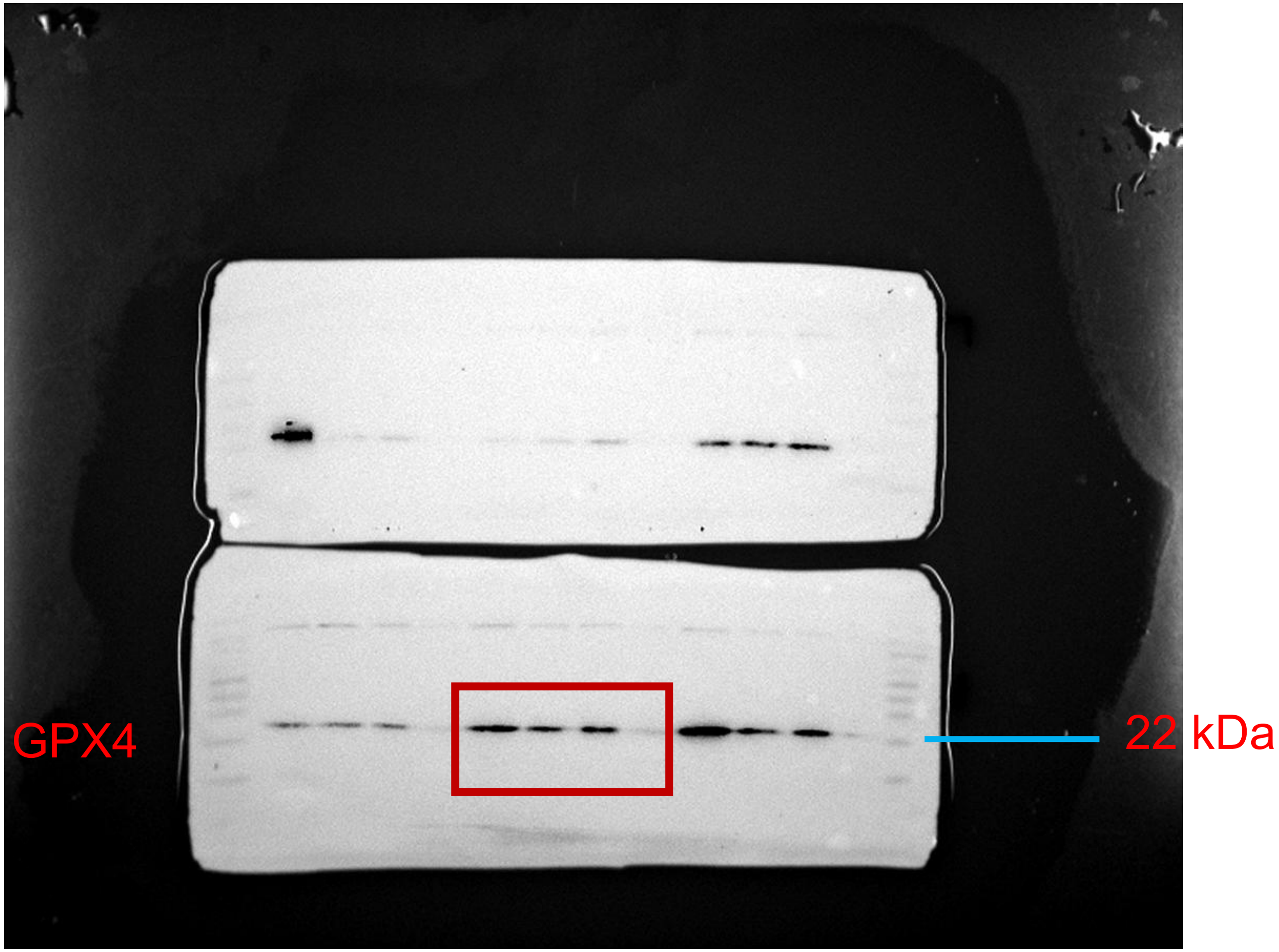

Figure 3C.A549-XCT 9.5

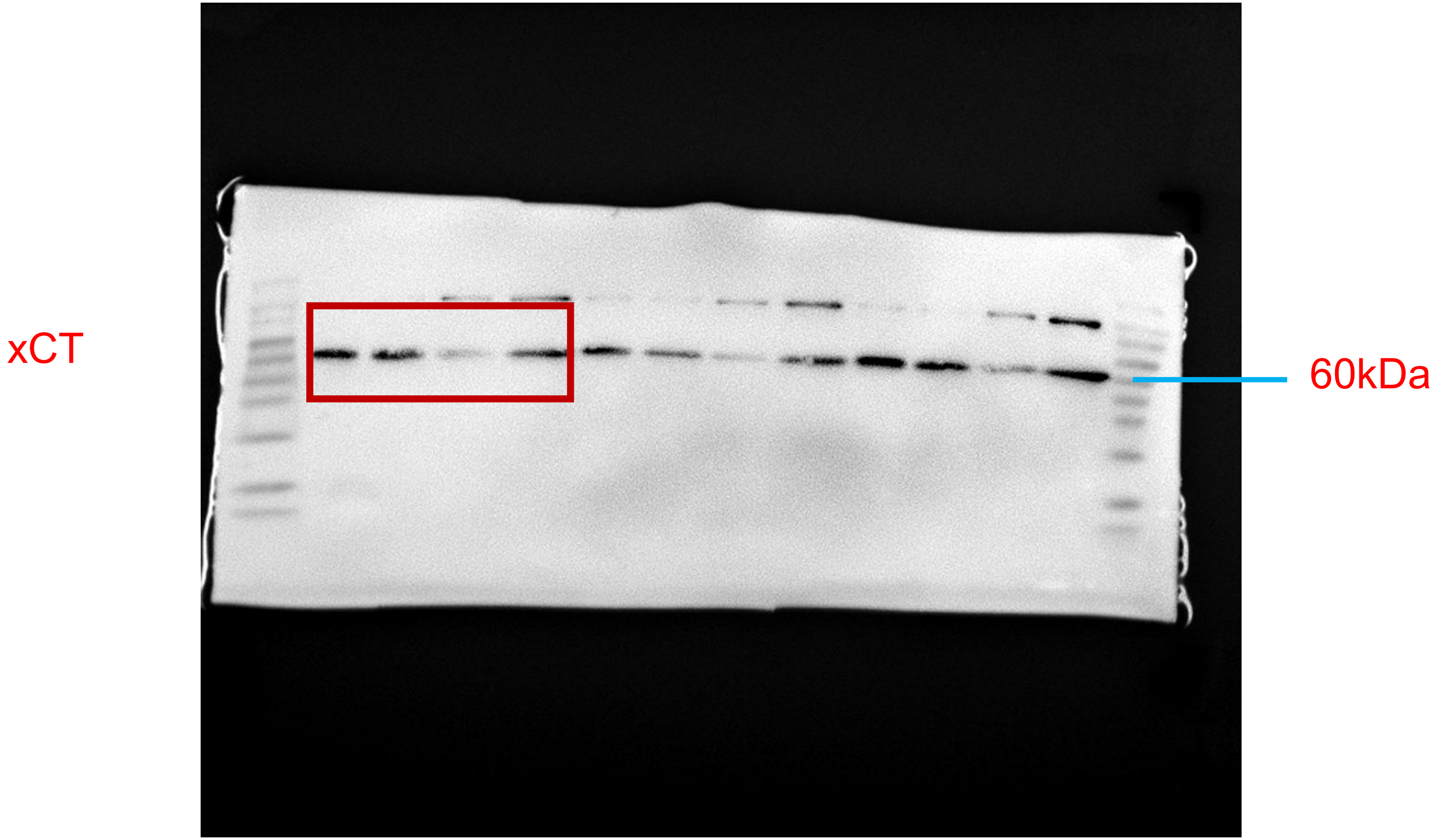

Figure 3C.H460-ACSL4

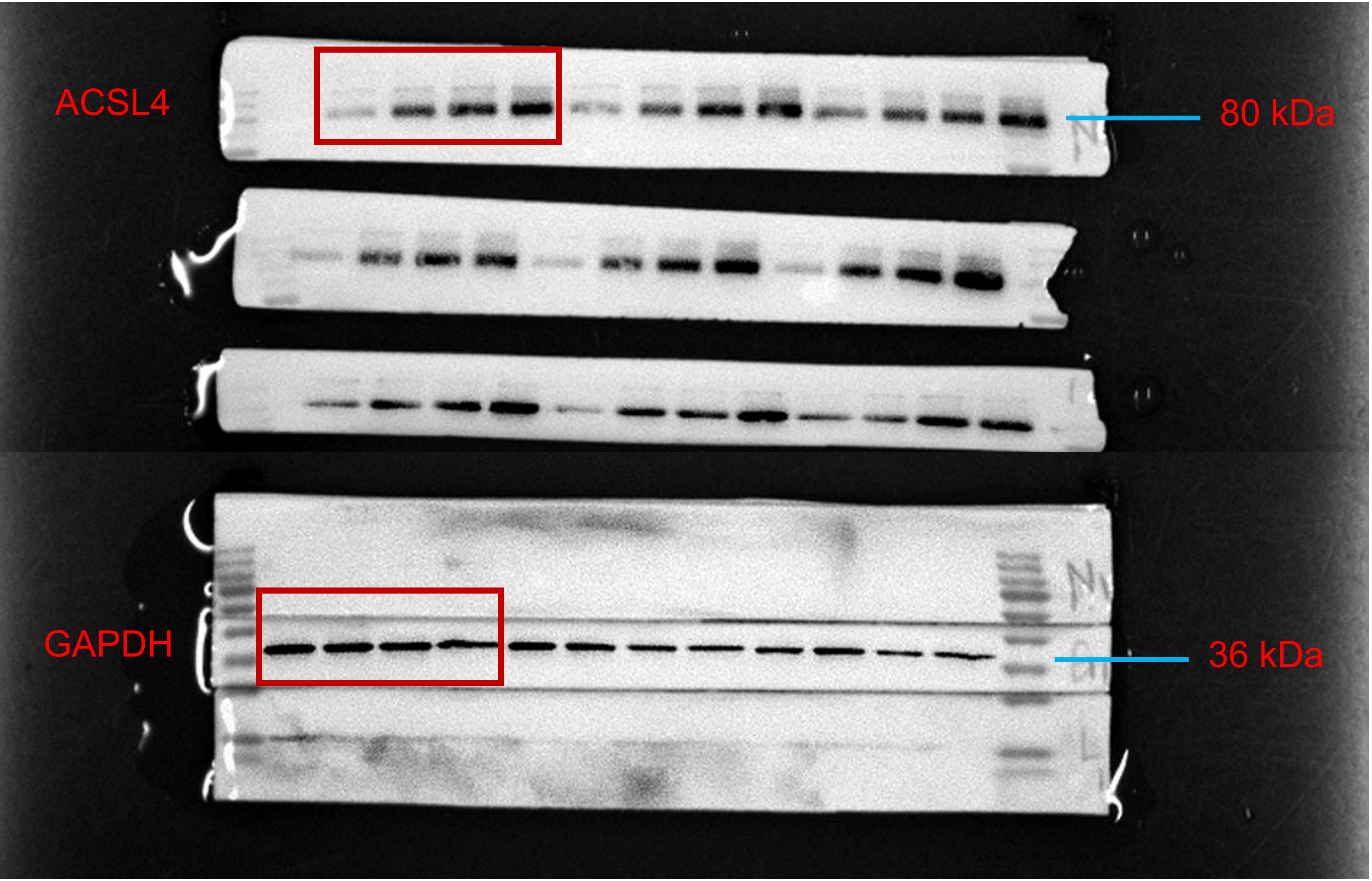

Figure 3C.H460-GPX4

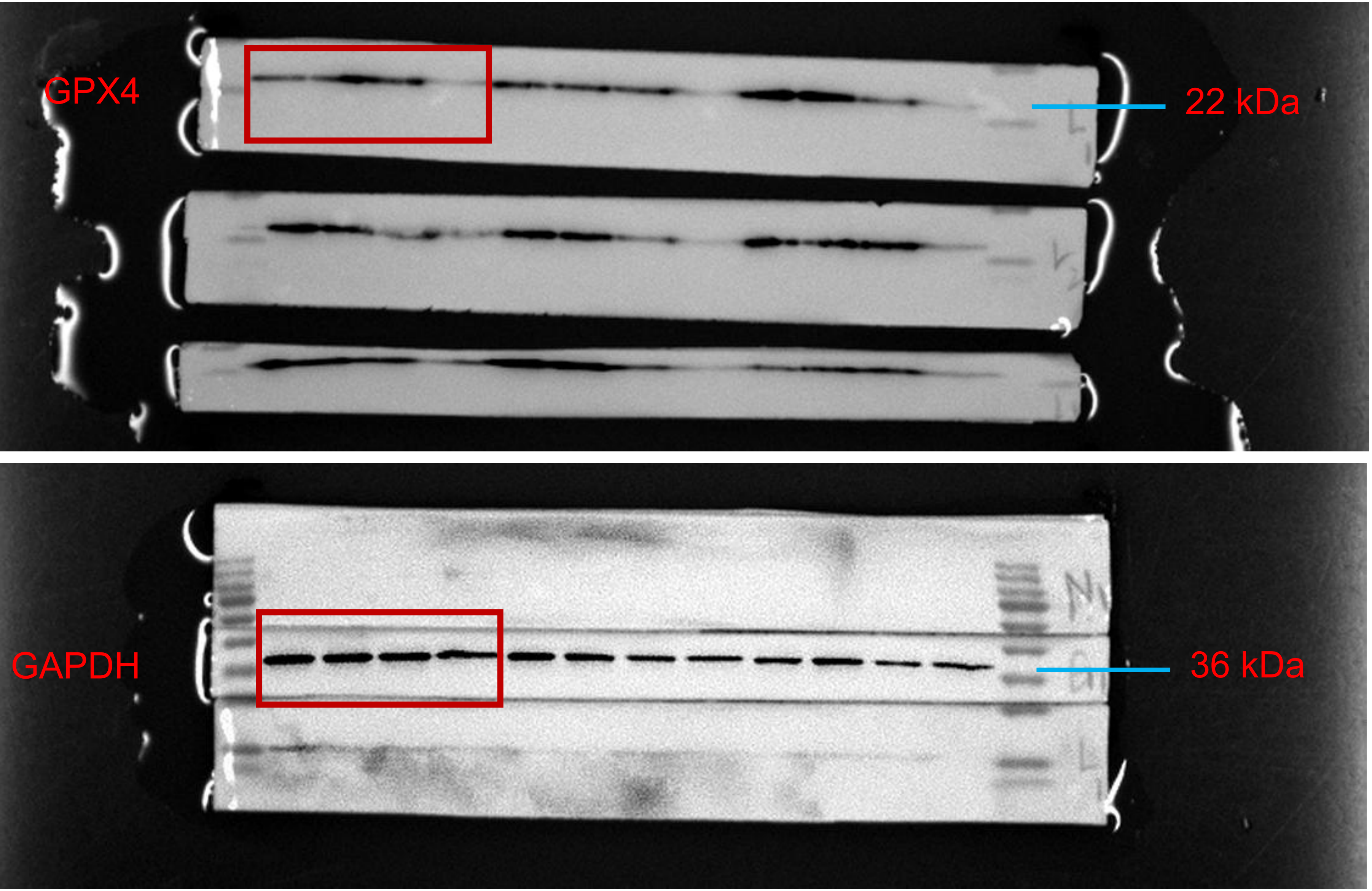

Figure 3C.H460-XCT

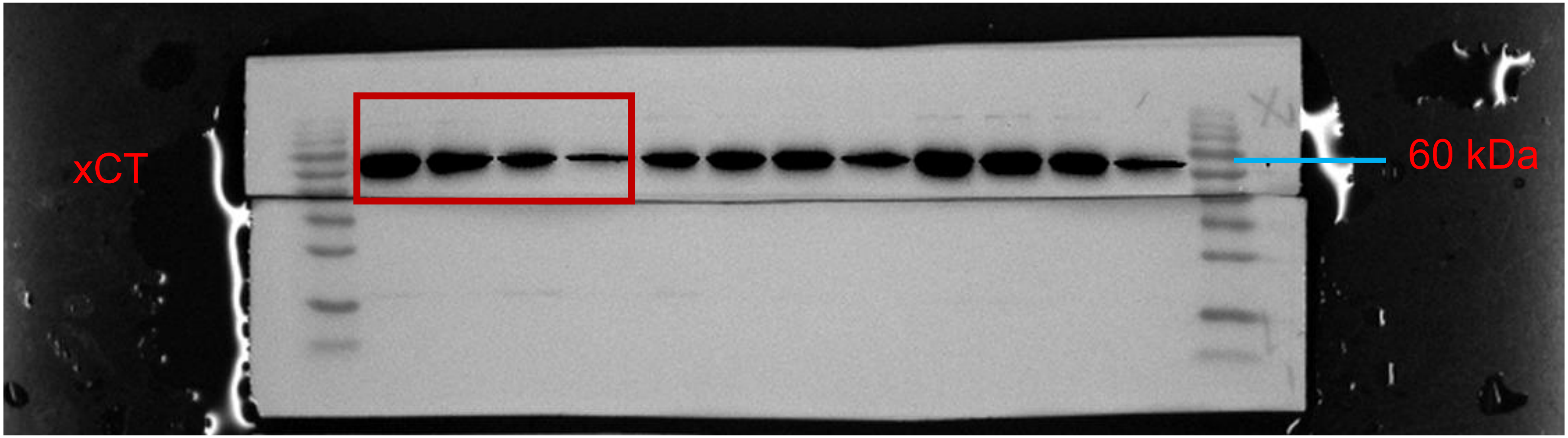

Figure 4

Figure 4A.A549—AMPK

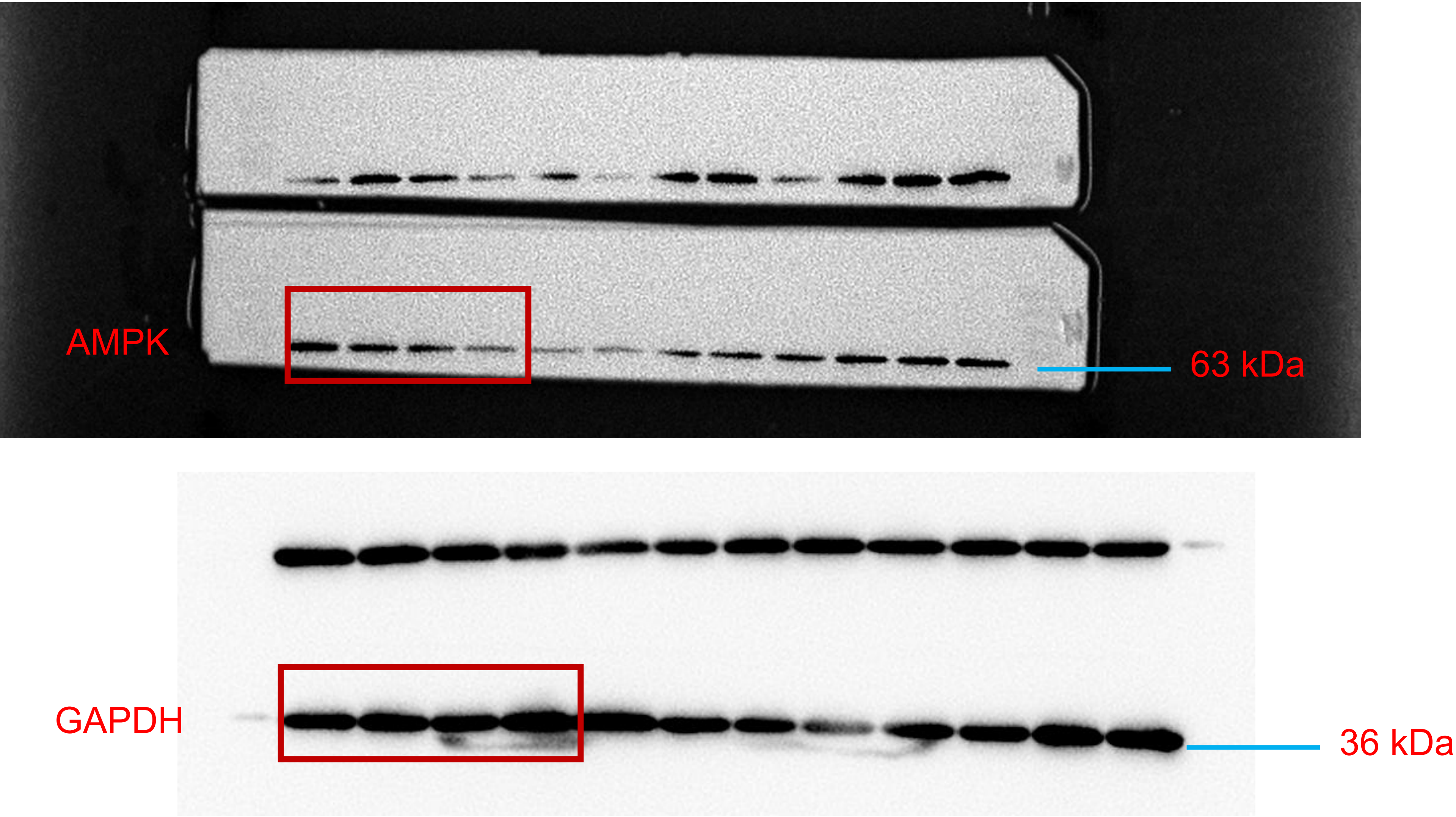

Figure 4A.A549-P-AMPK

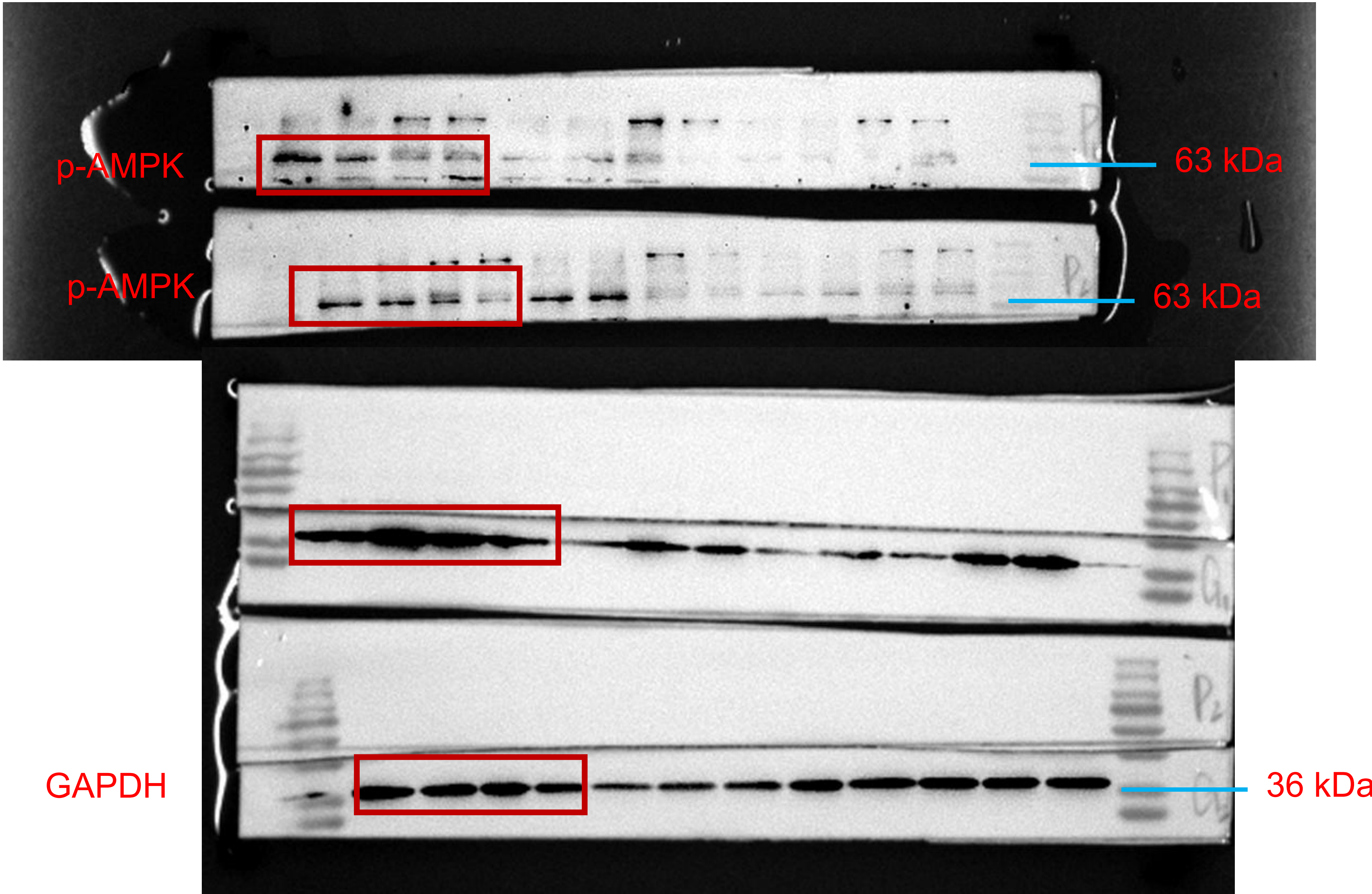

Figure 4A.H460-AMPK

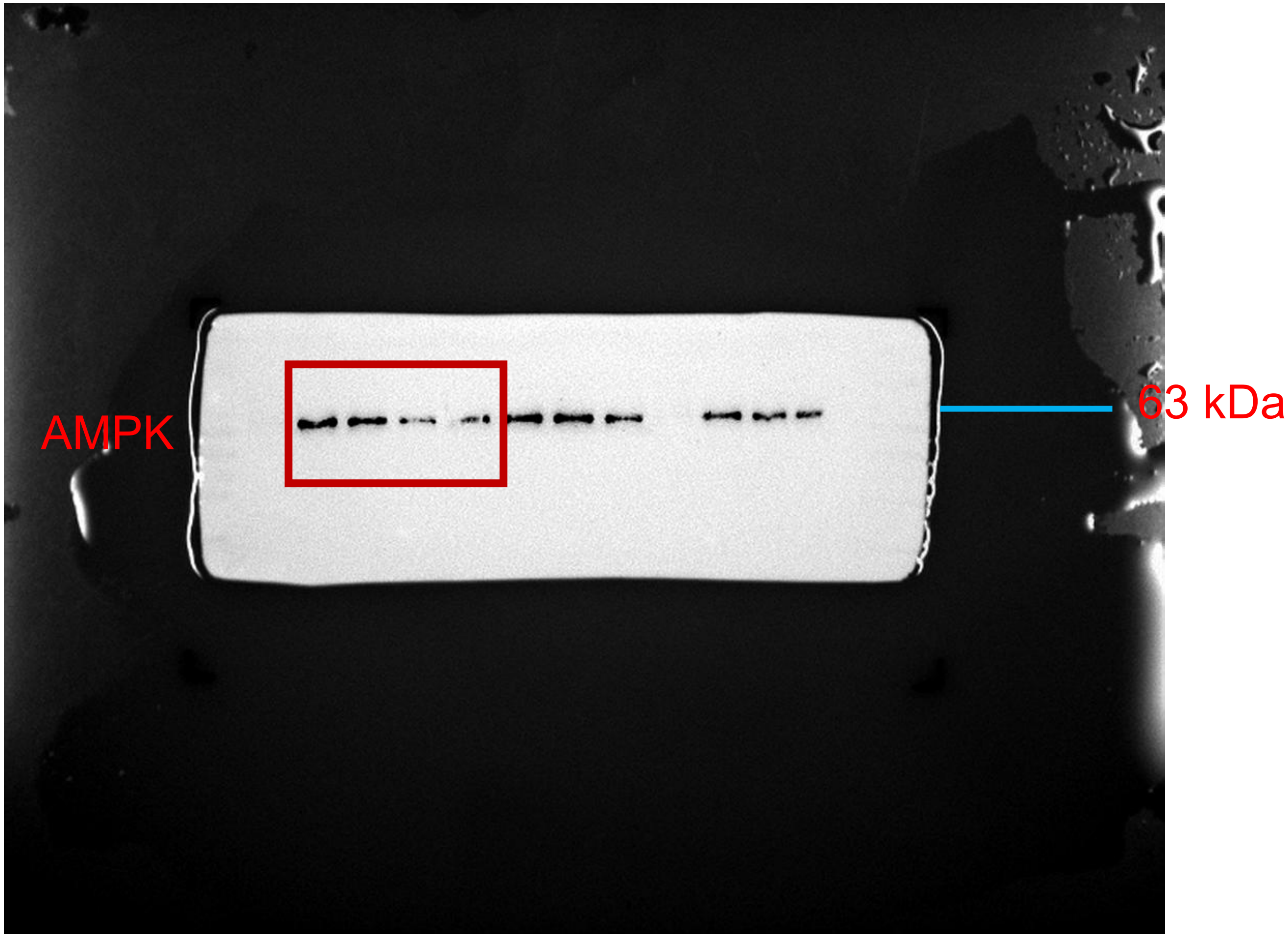

Figure 4A.H460-P-AMPK

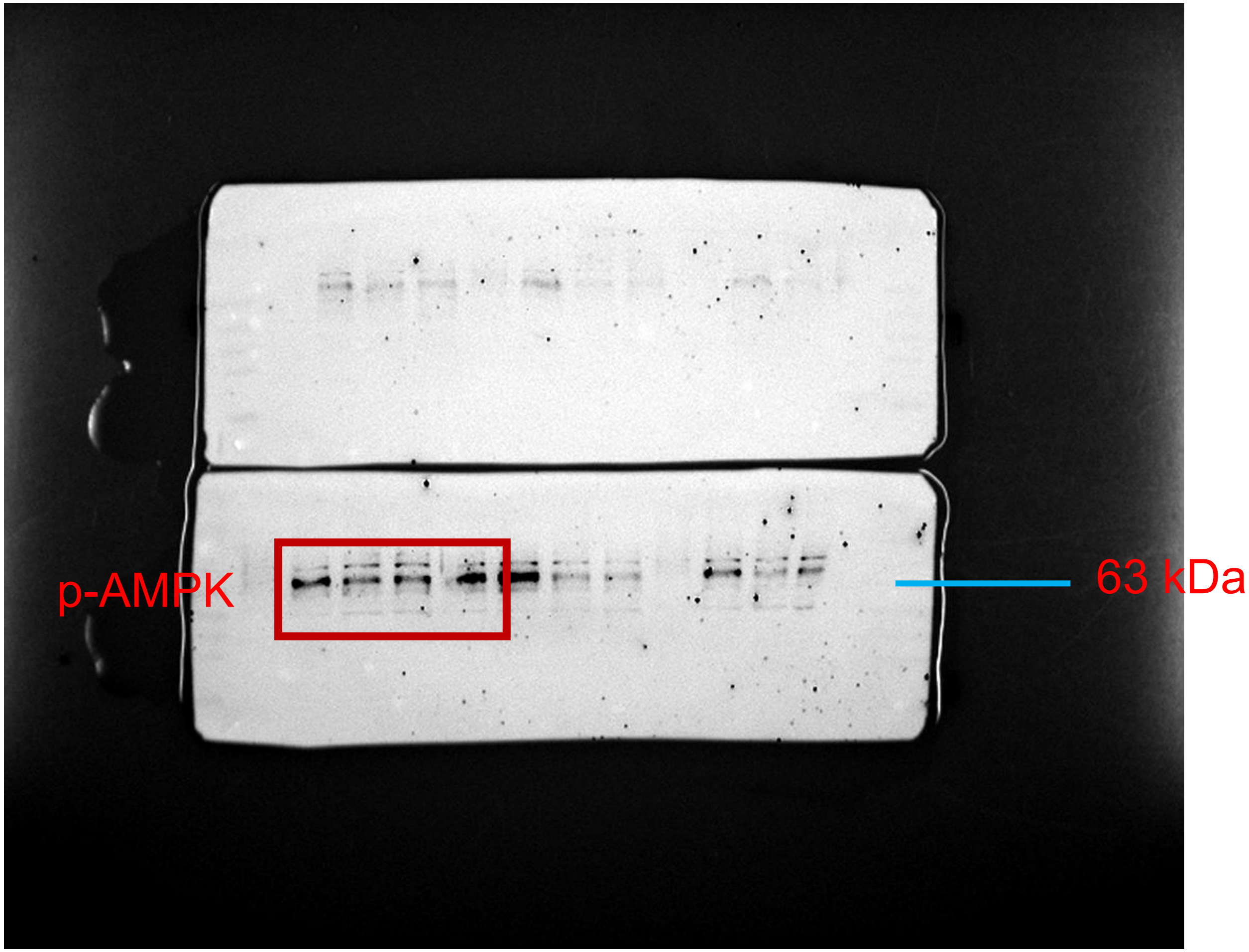

Figure 4C.A549-AMPK

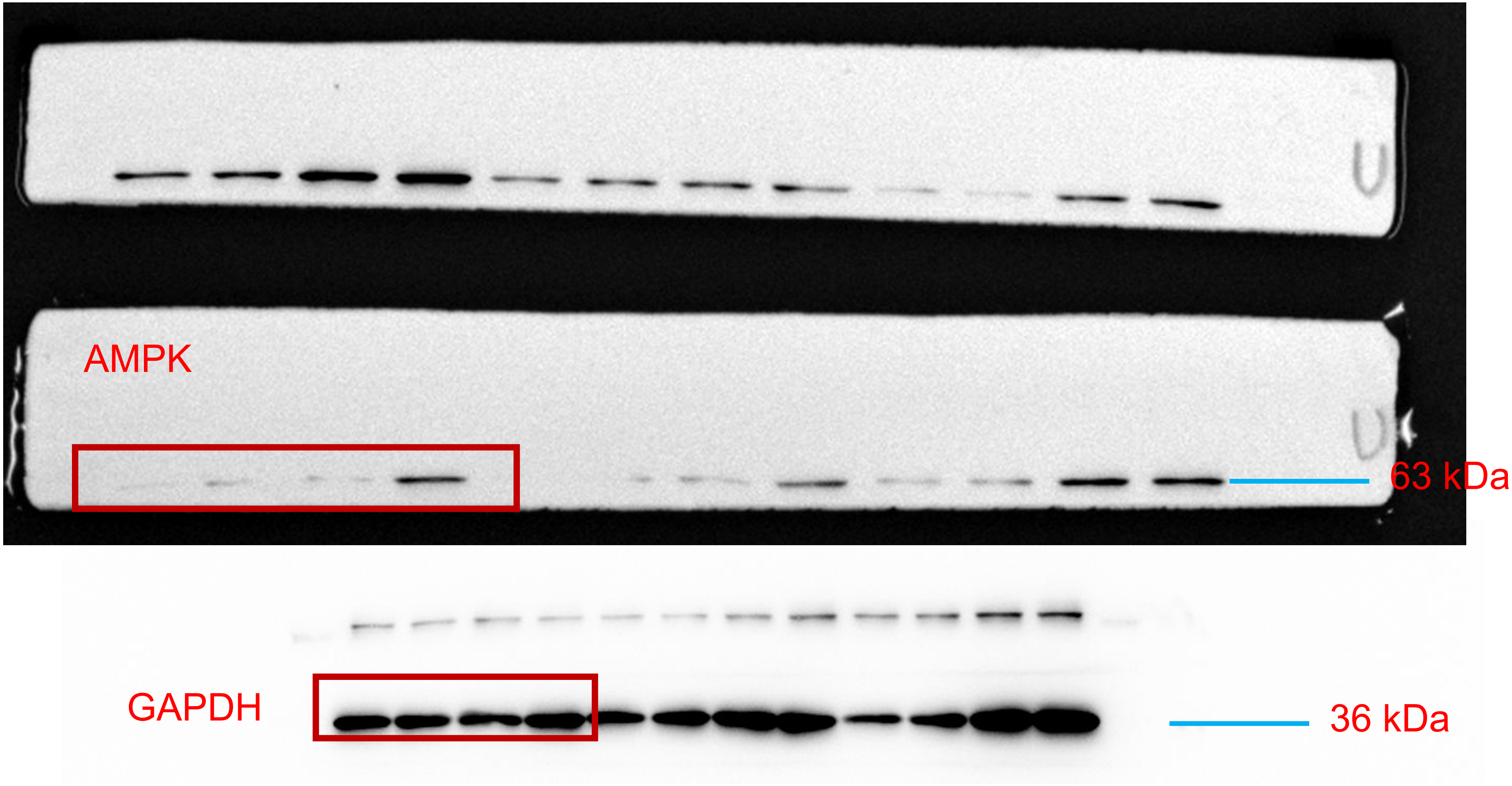

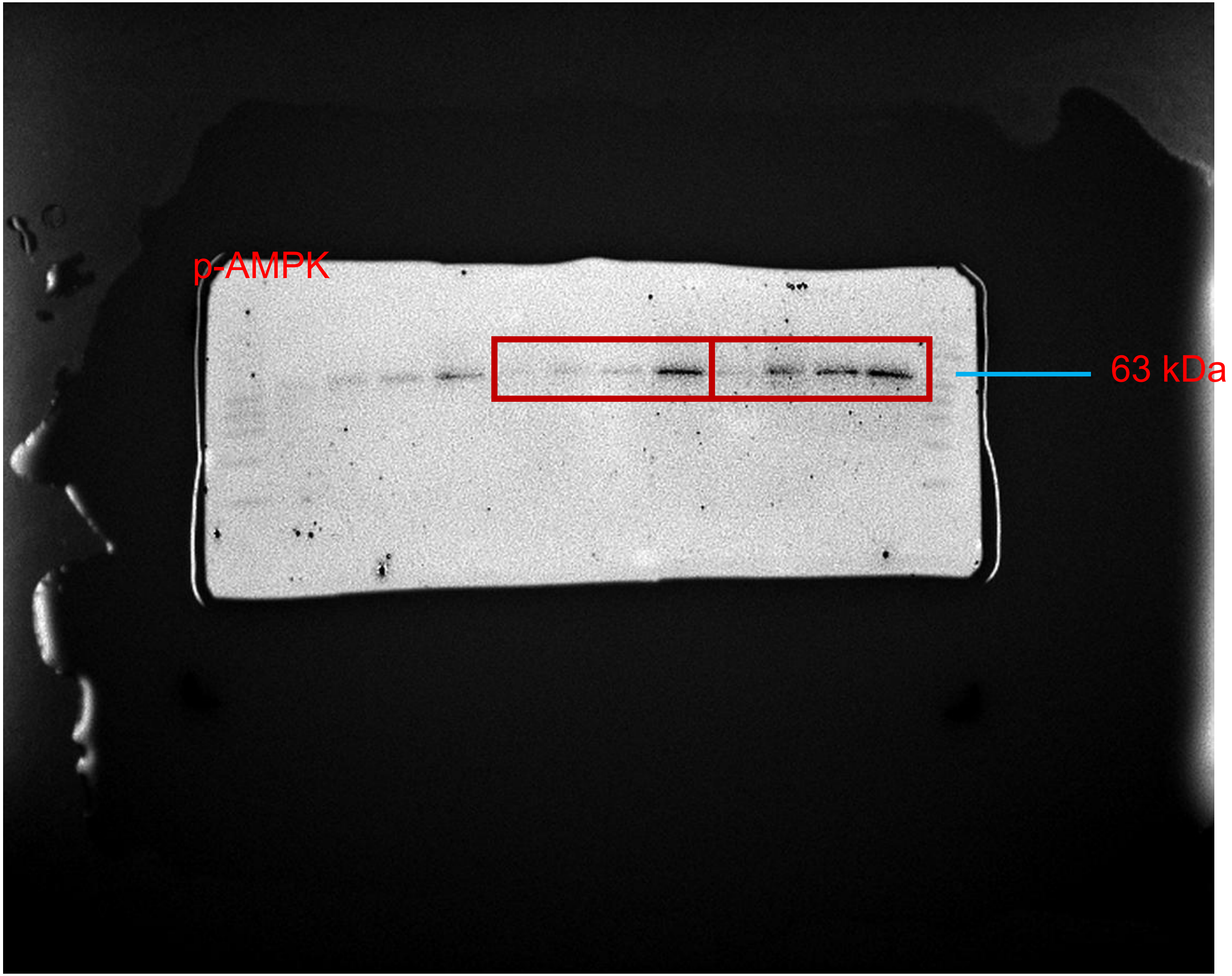
p-AMPK
63 kDa

Figure 4C.H460-AMPK

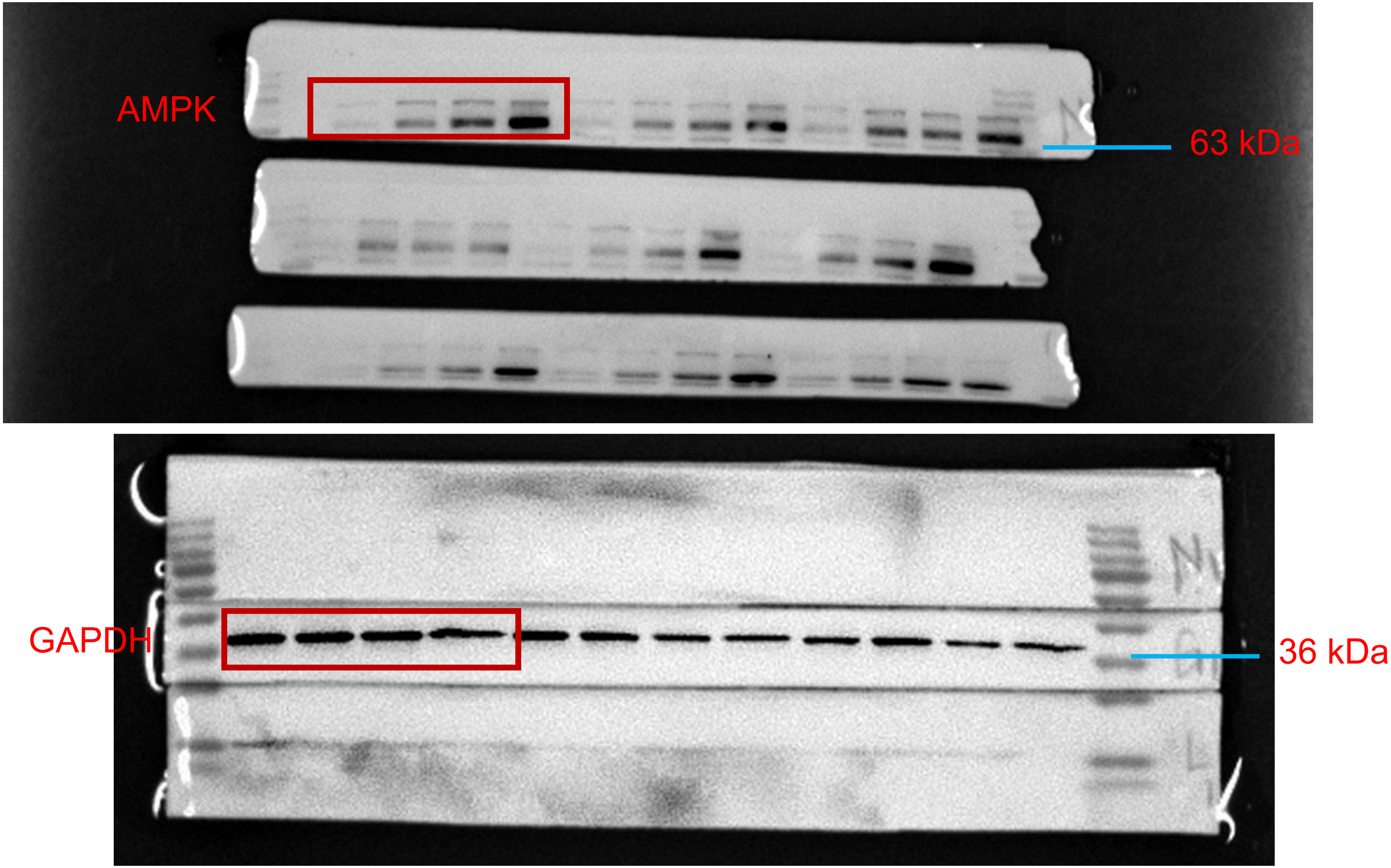

Figure 4C.H460-P-AMPK

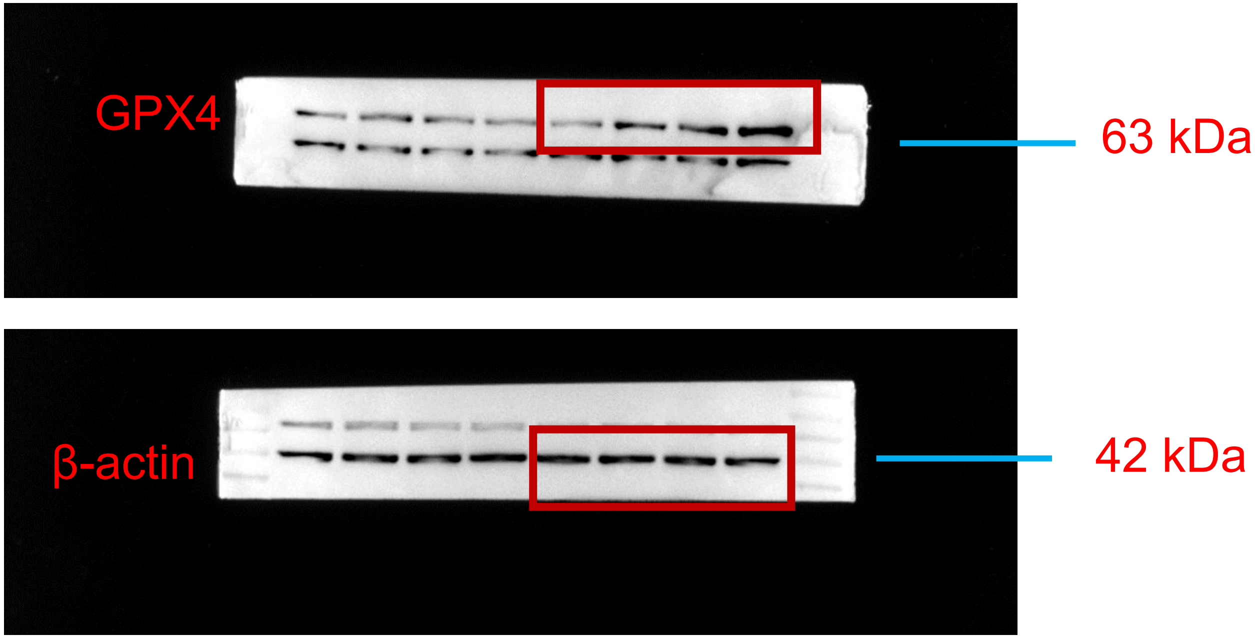

Figure 5

Figure 5A.A549-AMPK

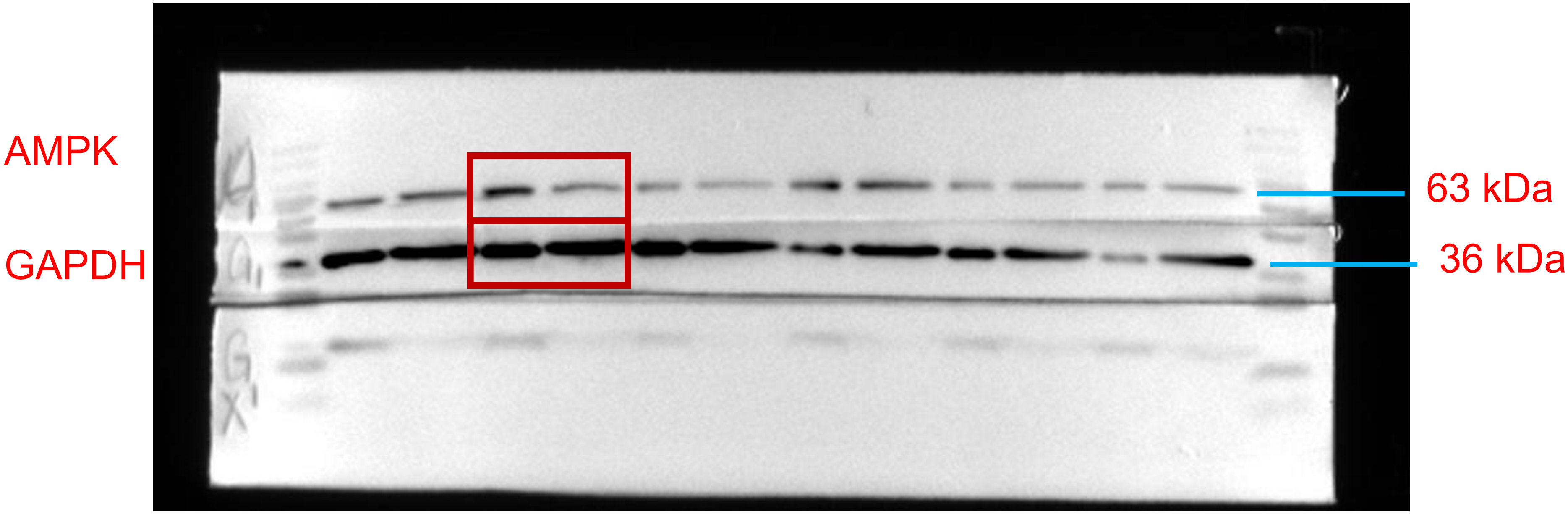

Figure 5A.A549-ULK1

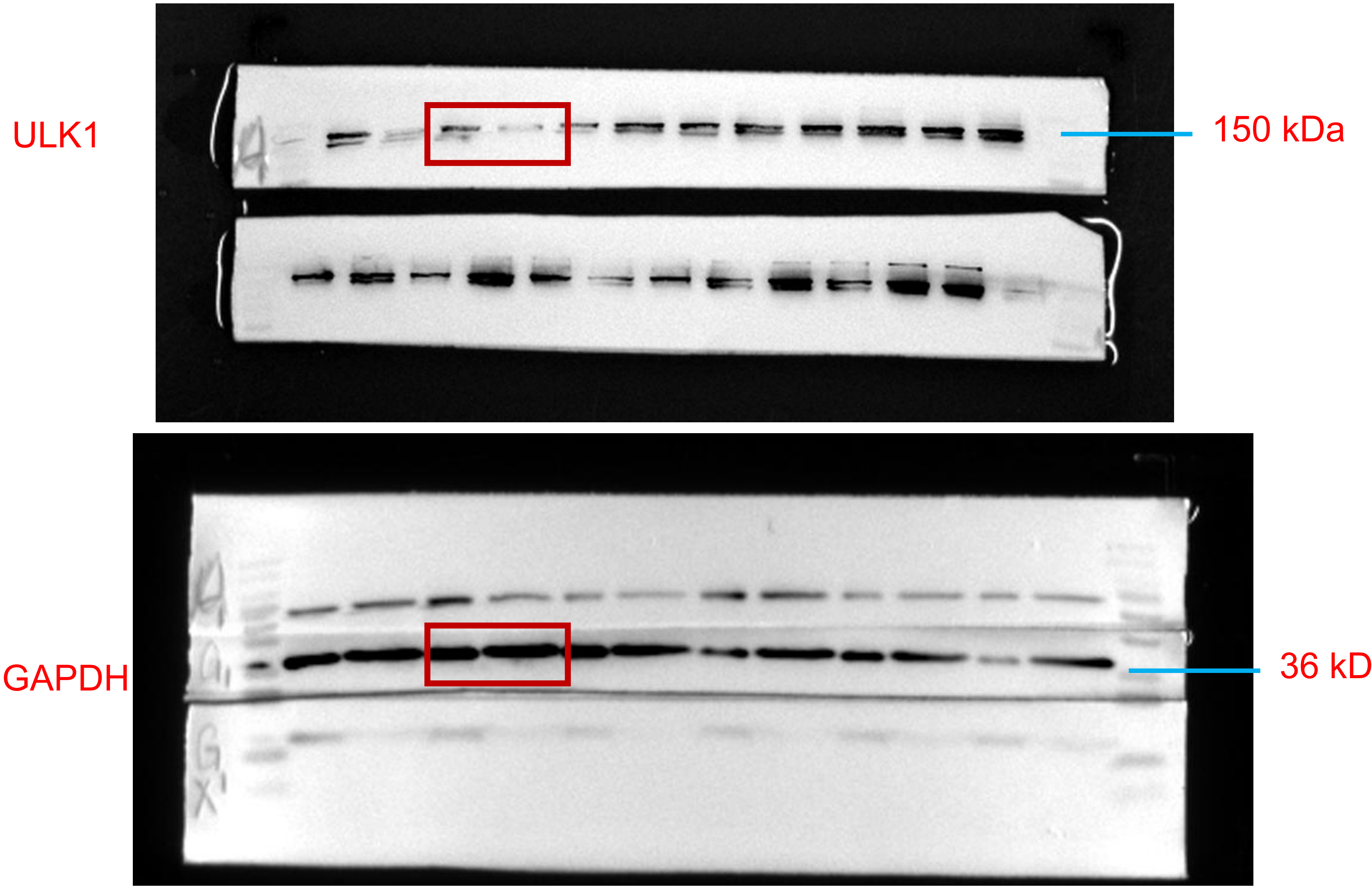

Figure 5A.A549-BECN1

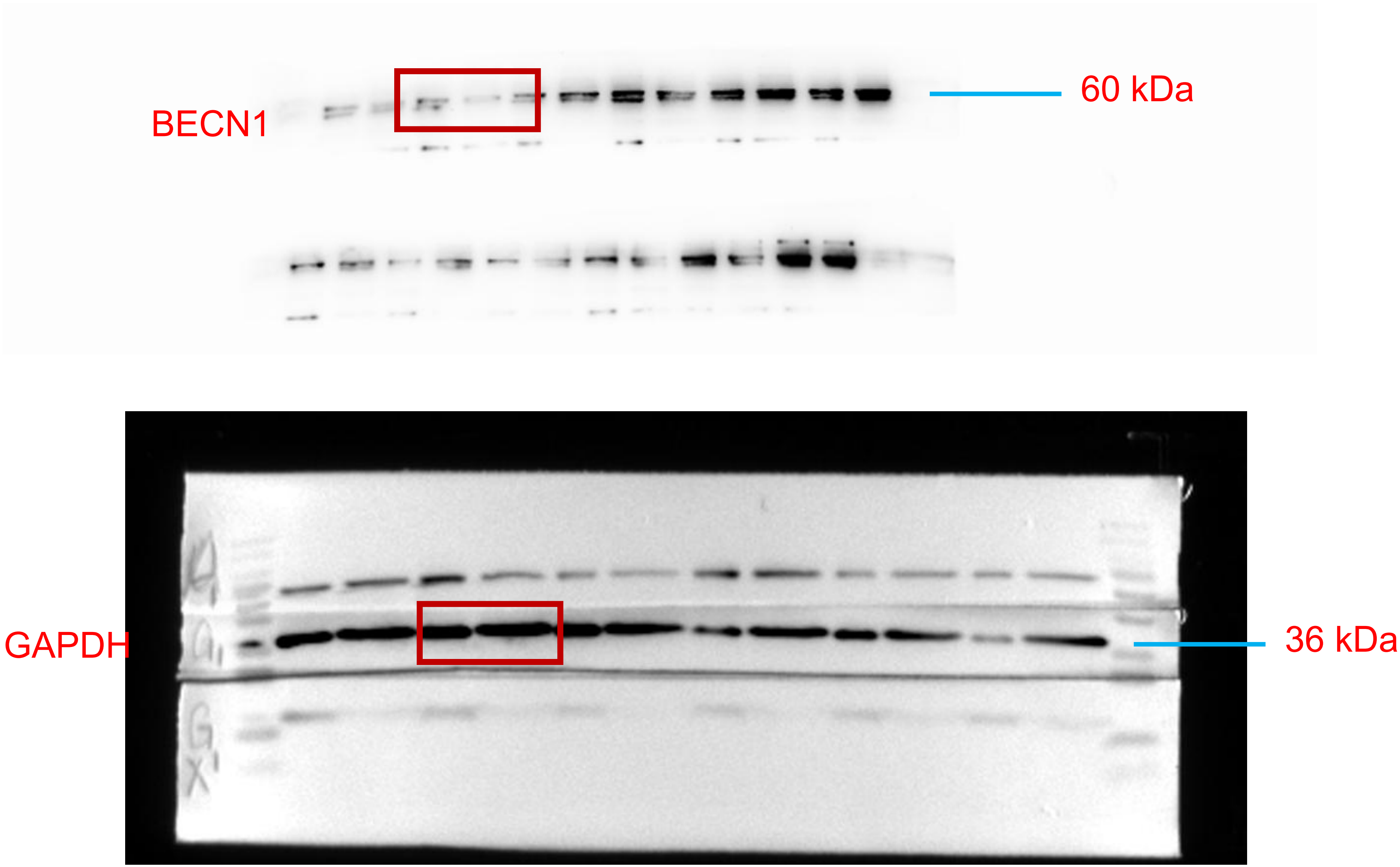

Figure 5A.A549-NCOA4

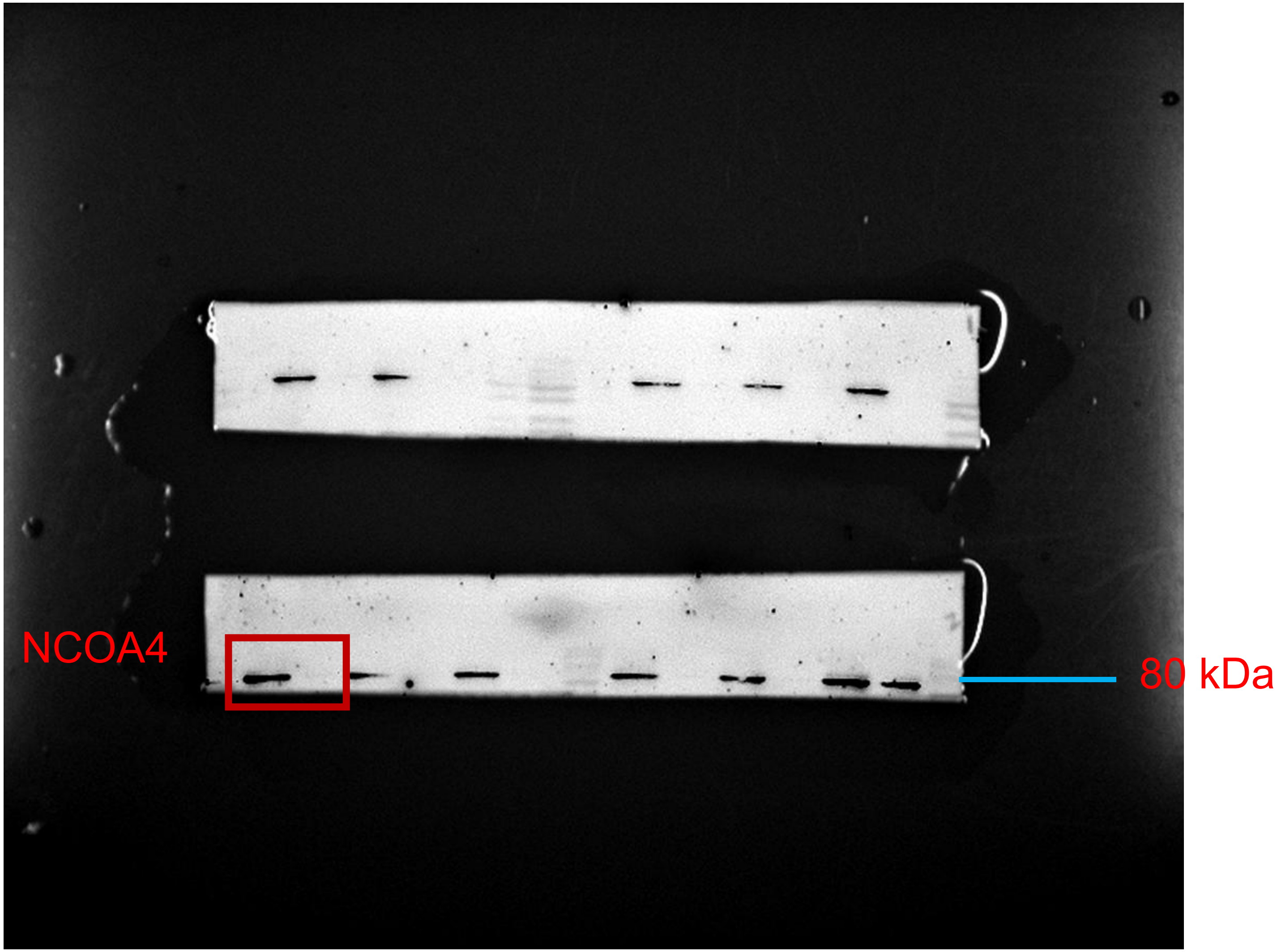

Figure 5A.A549-LC3B

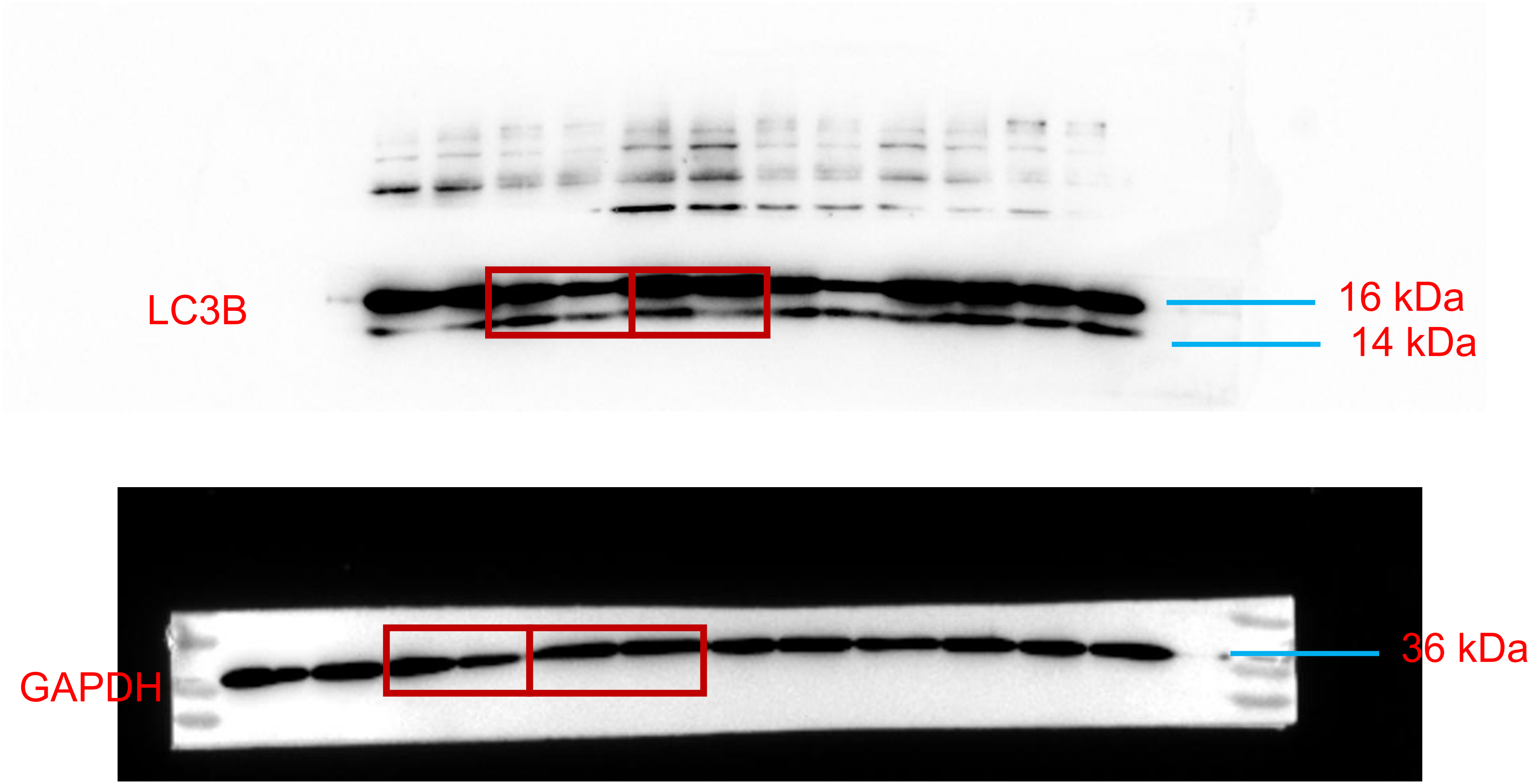

Figure 5A.H460-AMPK

AMPK

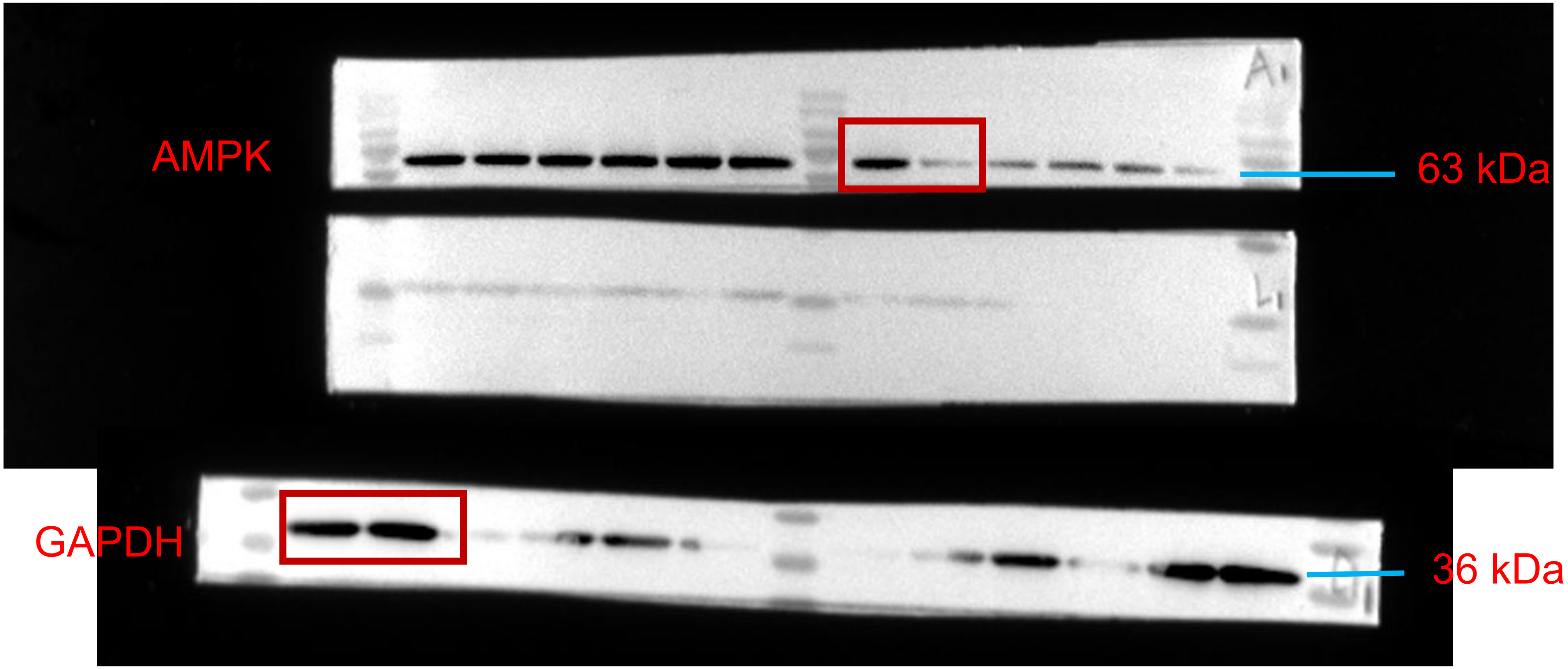

Figure 5A.H460-ULK1

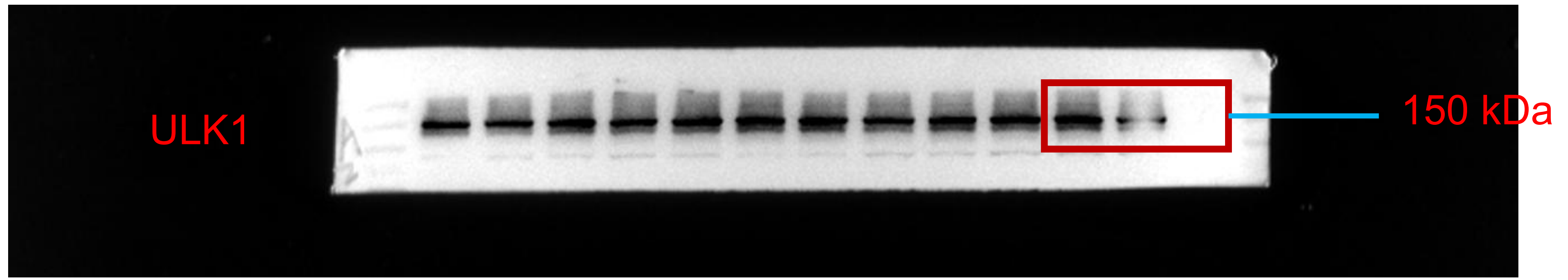

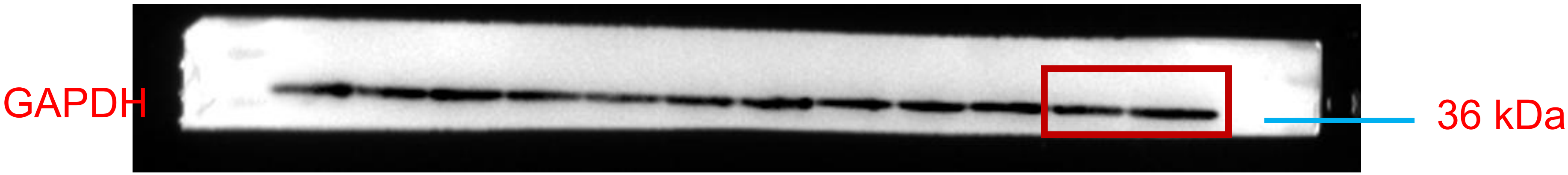

Figure 5A.H460-BECN1

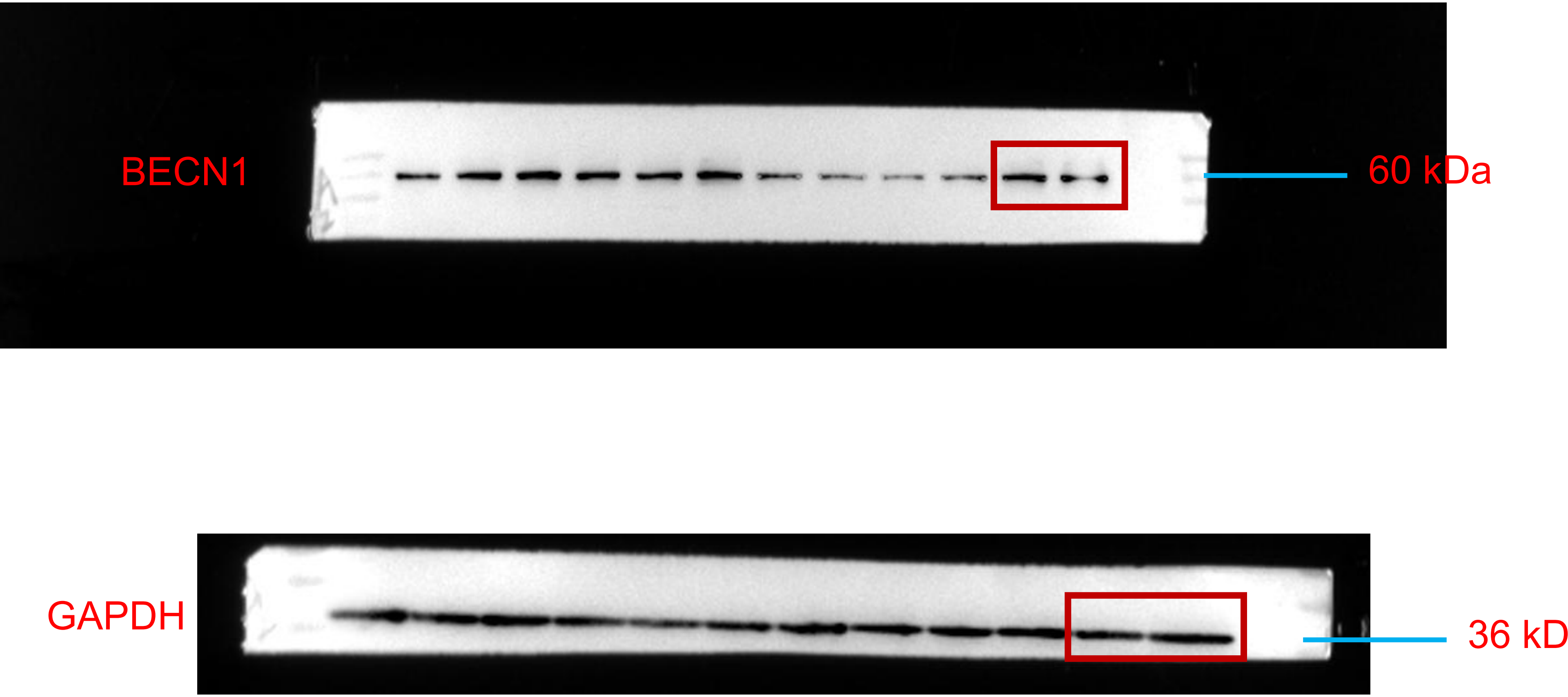

Figure 5A.H460-NCOA4

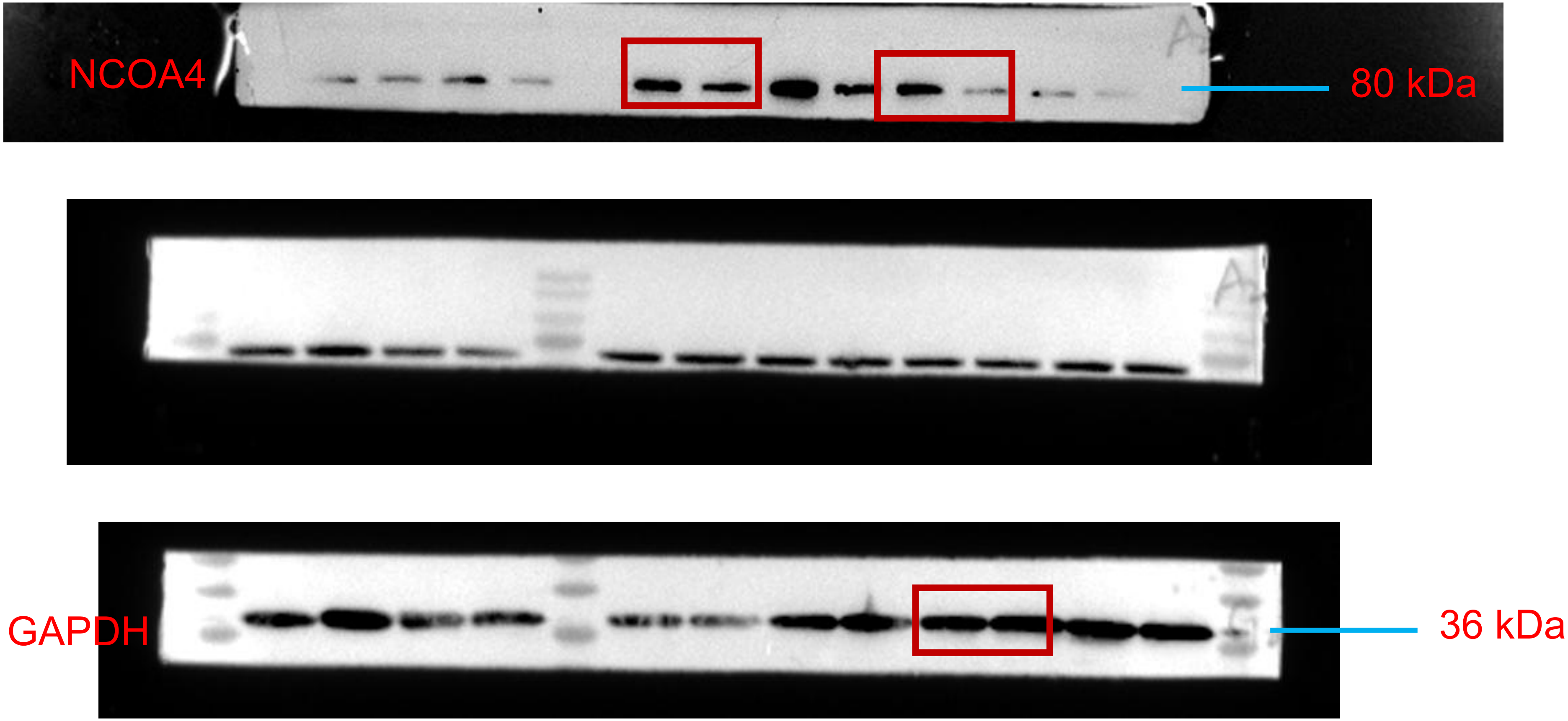

Figure 5A.H460-LC3B

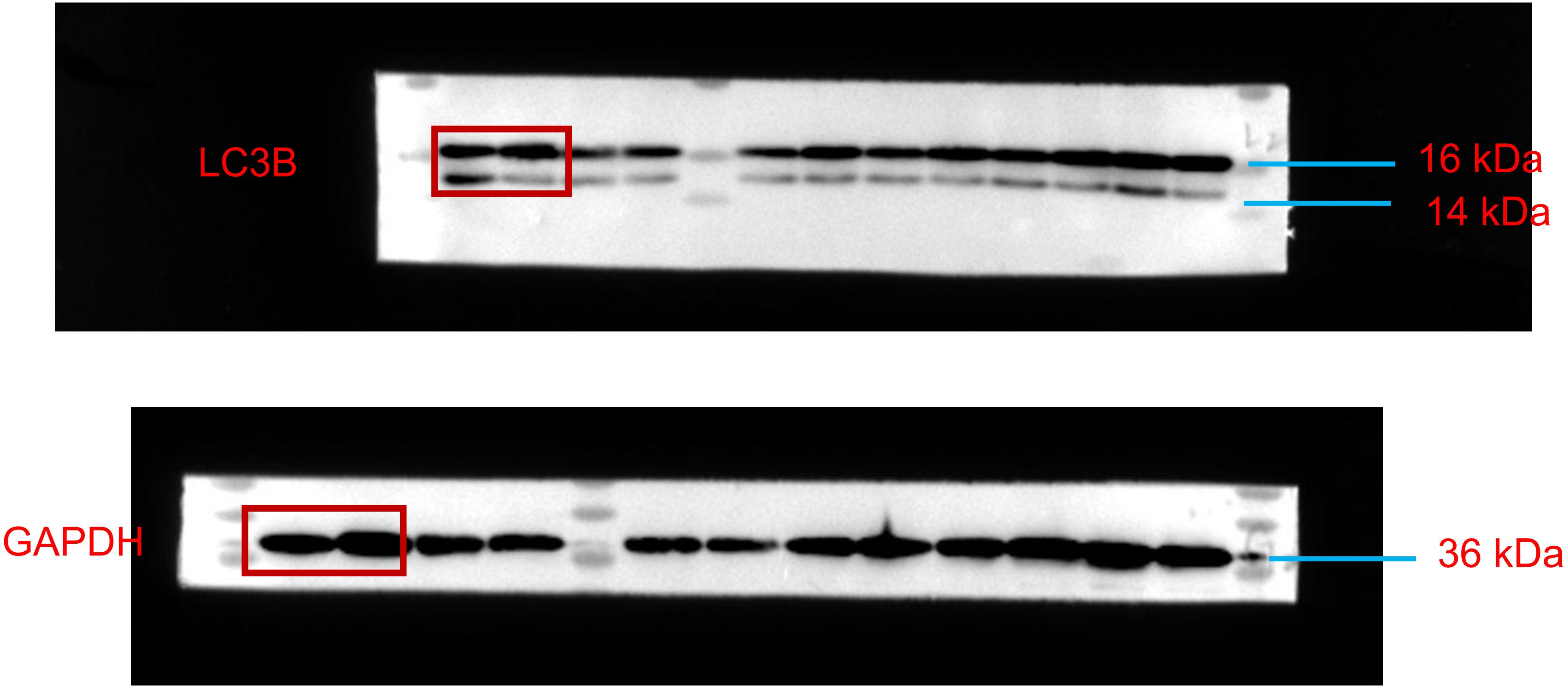

Figure 5C. A549-AMPK

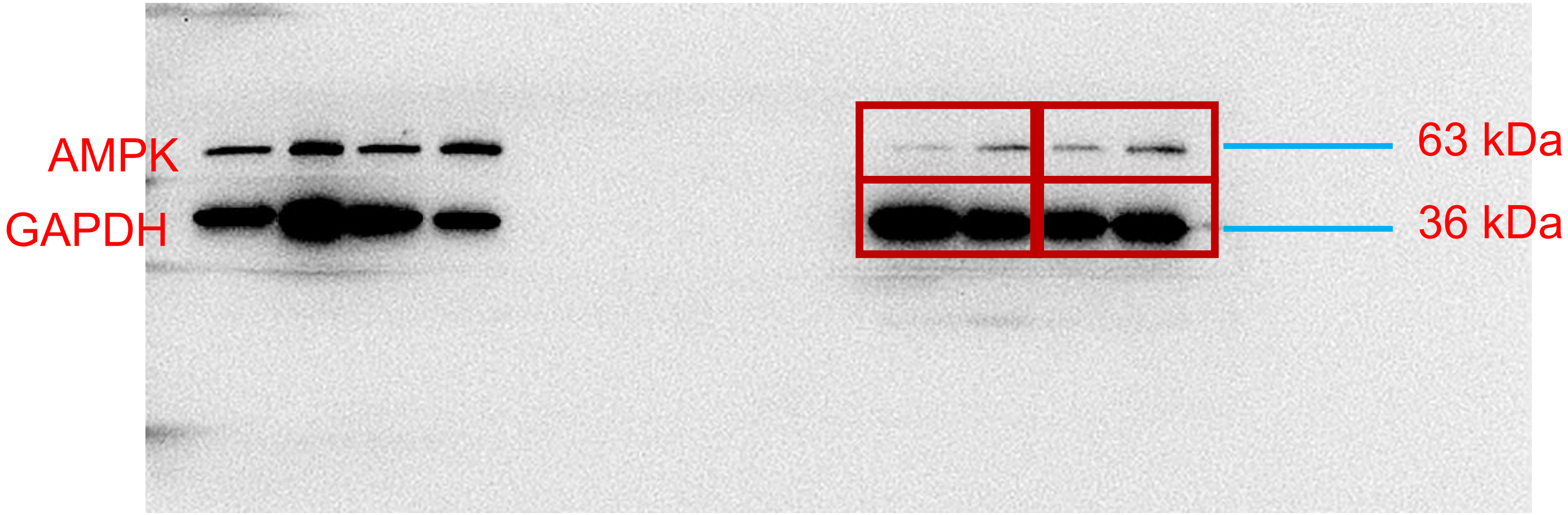

Figure 5C. A549-ULK1

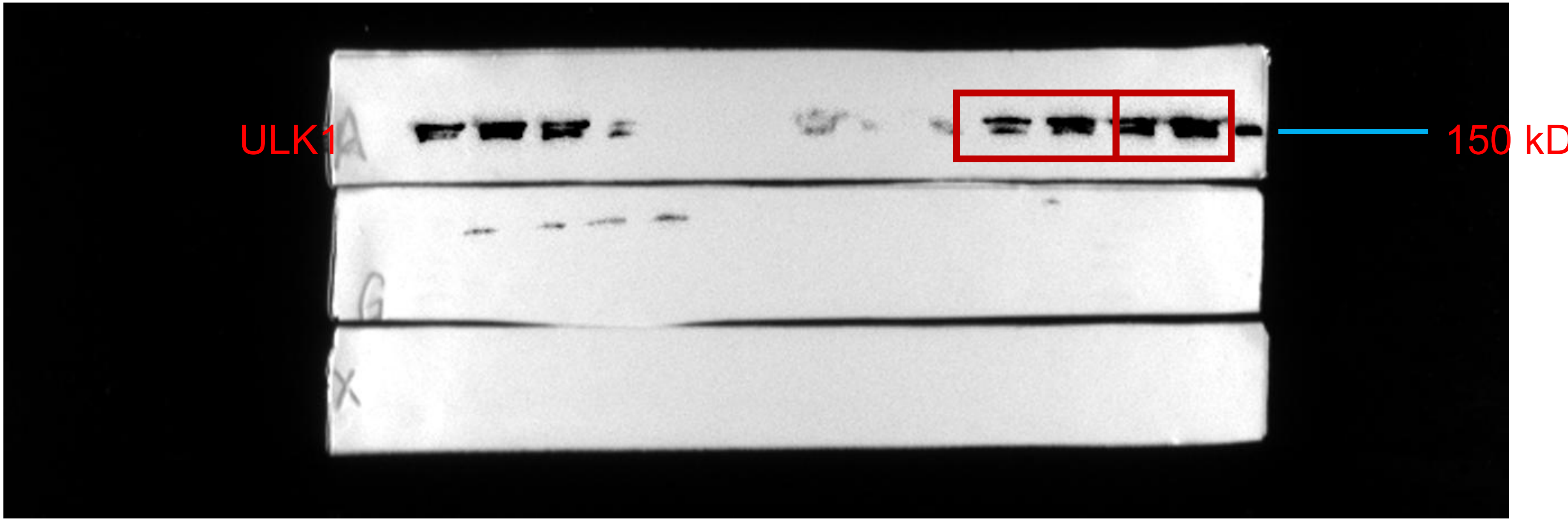

Figure 5C. A549-BECN1 9.28

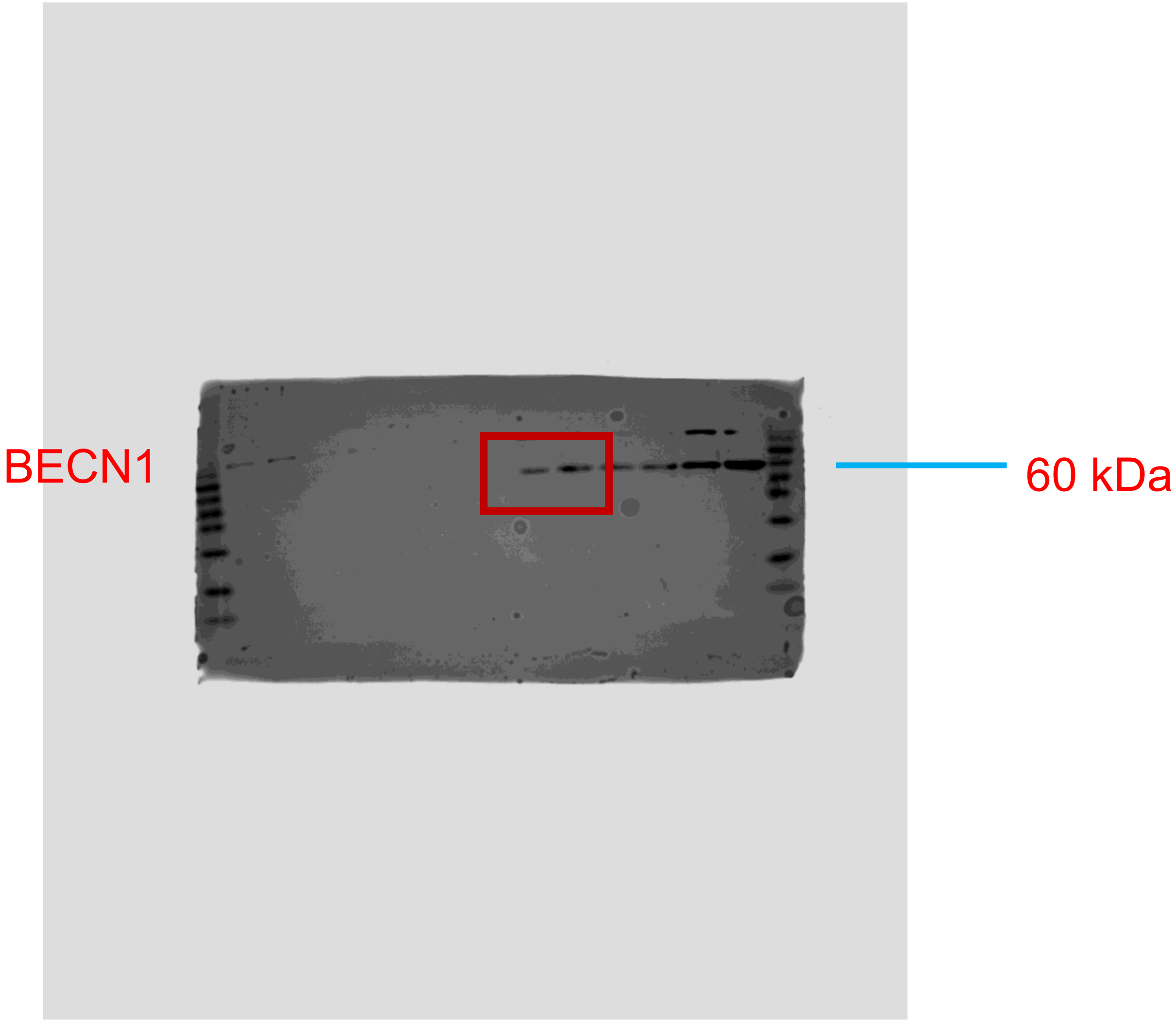

Figure 5C. A549-NCOA4

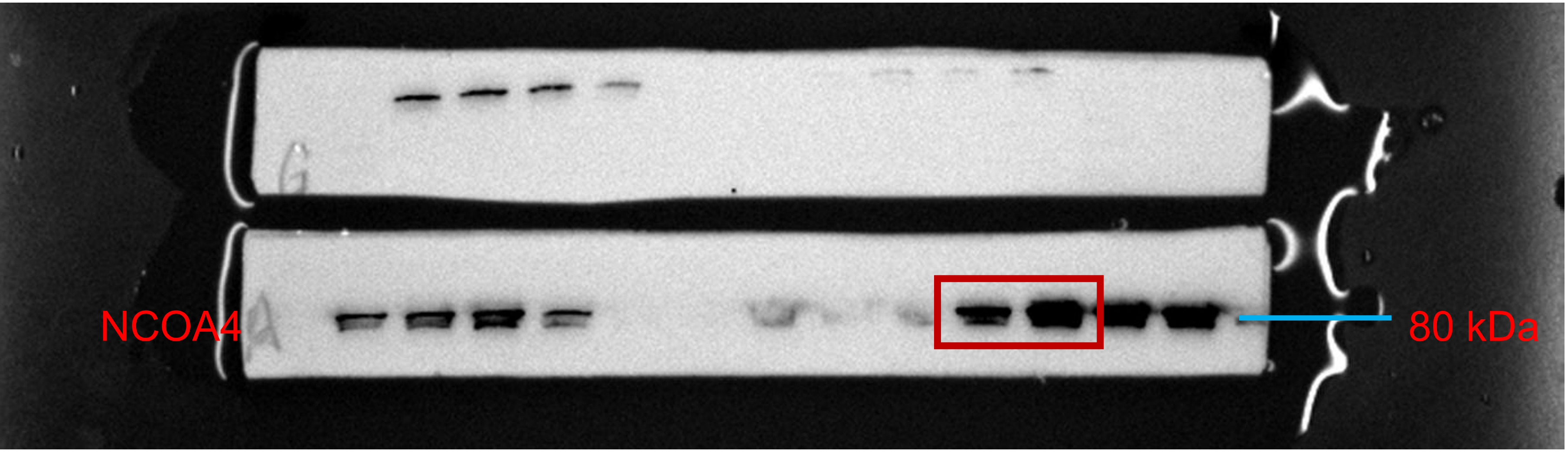

Figure 5C. A549-LC3B

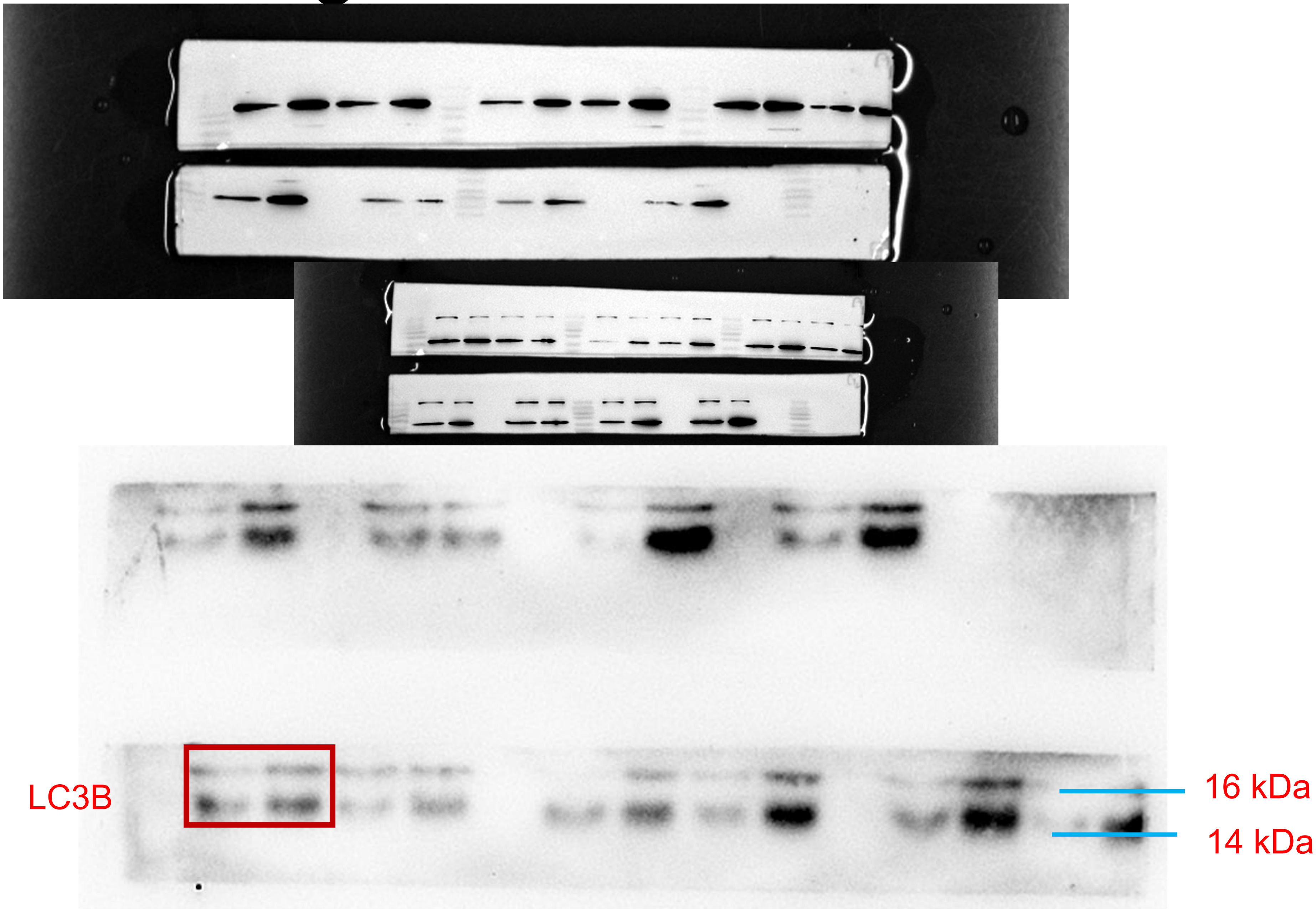

Figure 5C. H460-AMPK

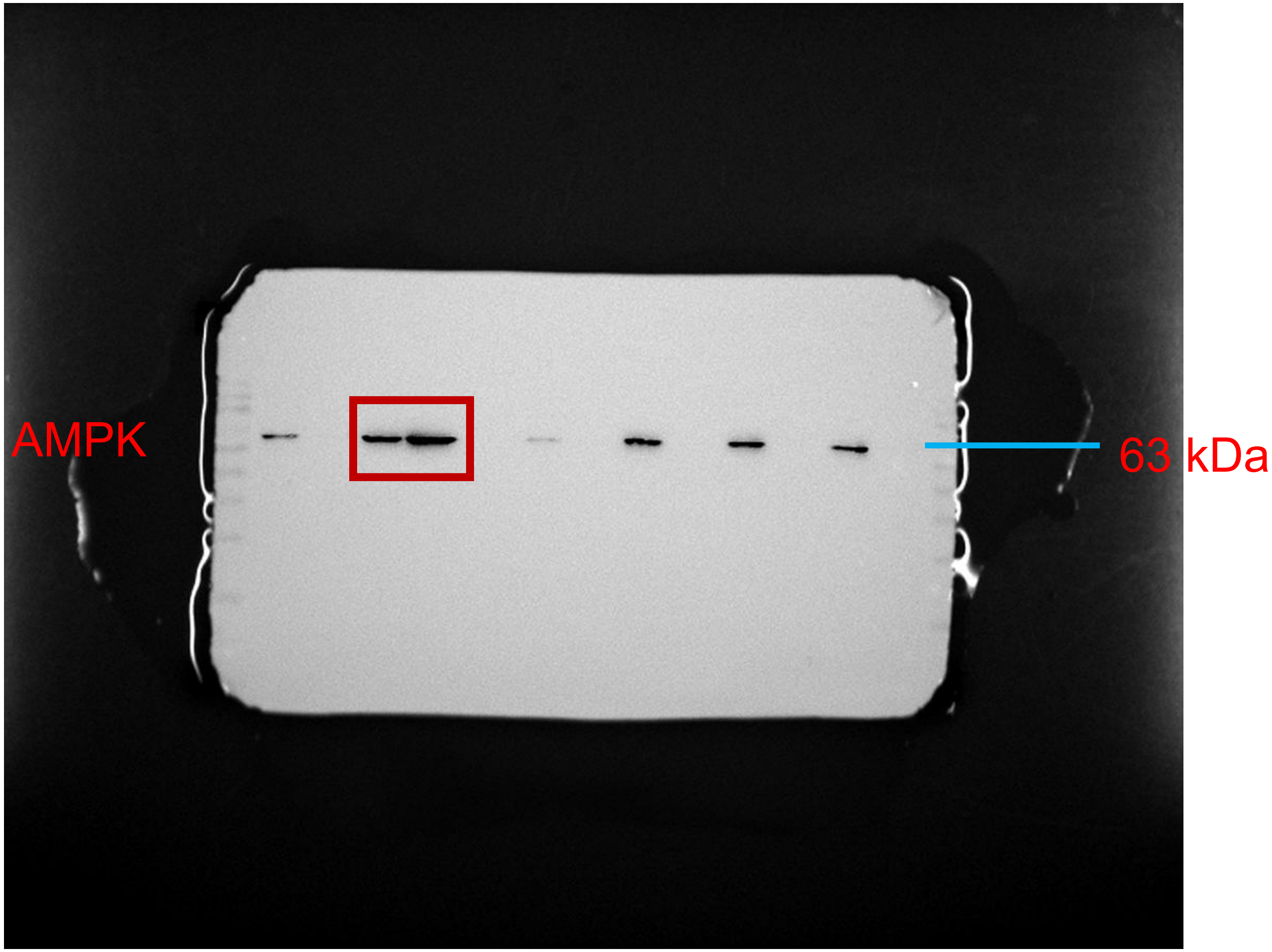

Figure 5C. H460-ULK110.20

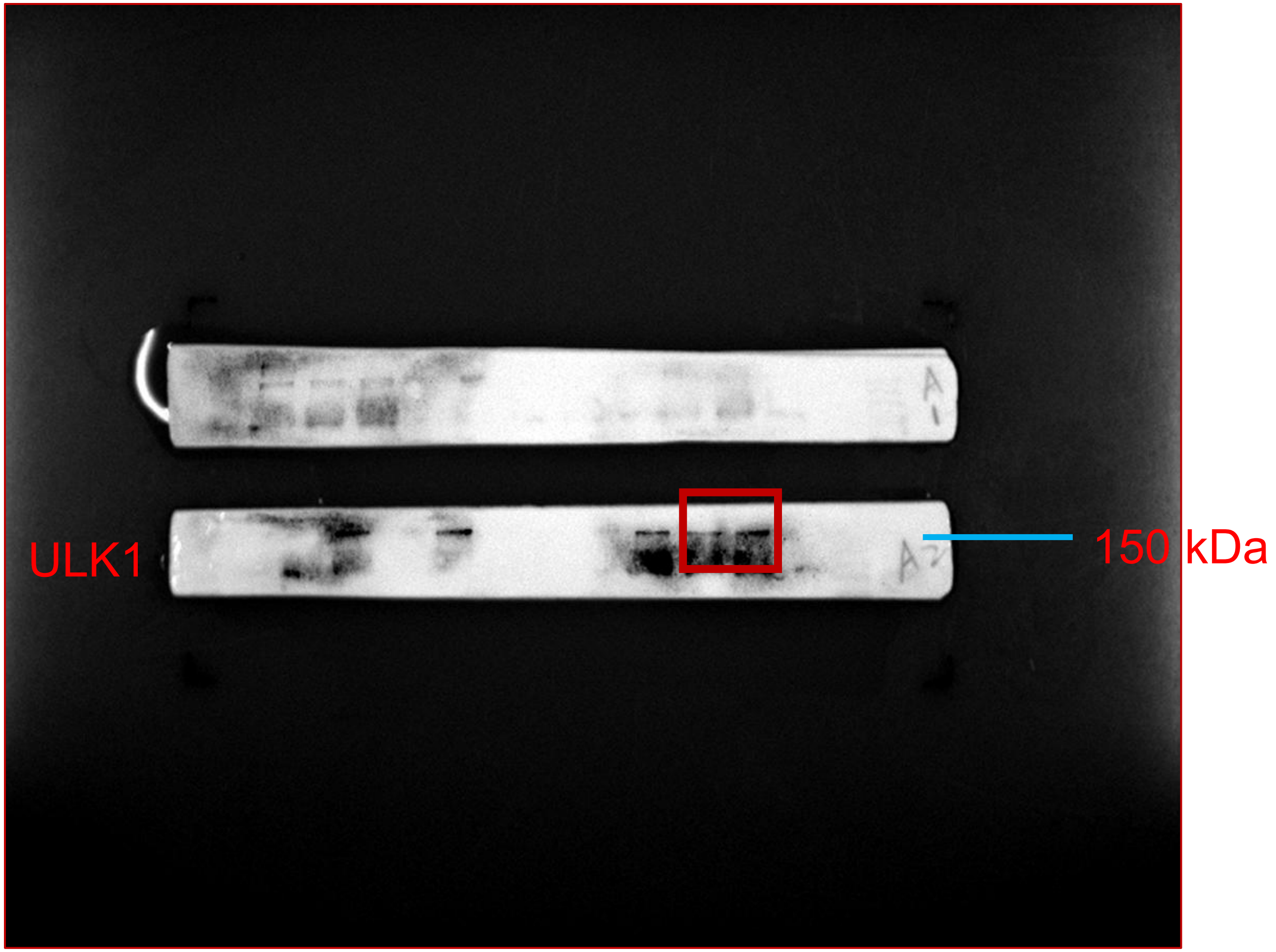

Figure 5C. H460-BECN1

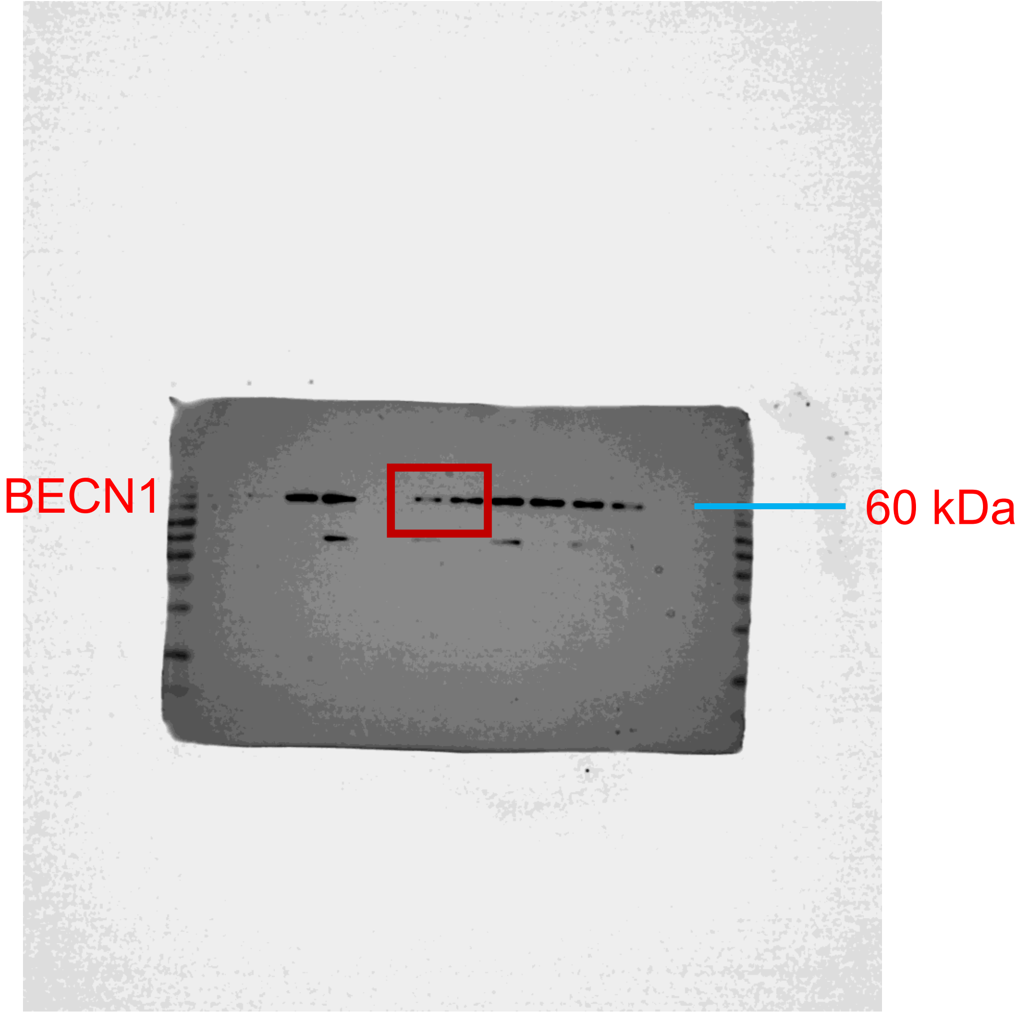

Figure 5C. H460-NCOA410.20

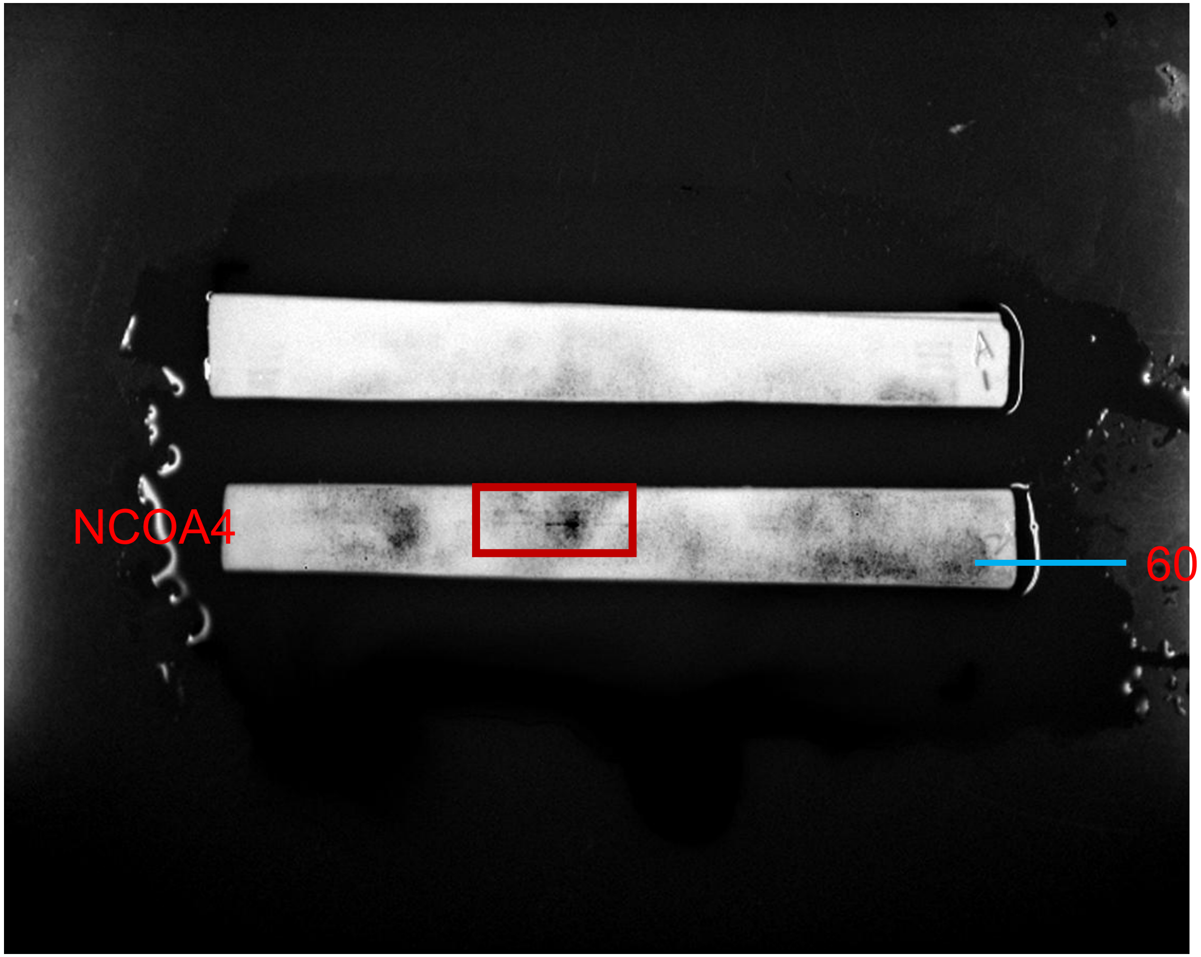

Figure 5C. H460-LC3B

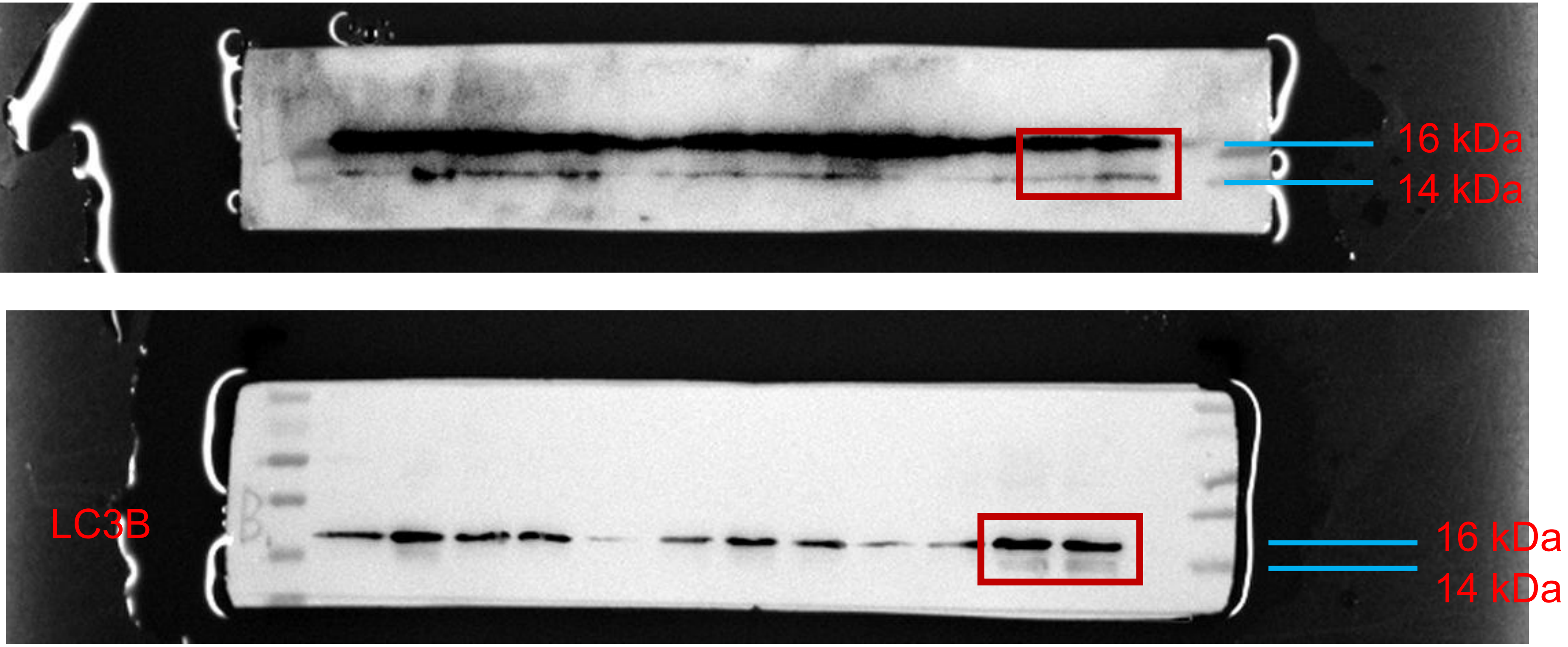

Figure 6

Figure 6.A A549-GPX4

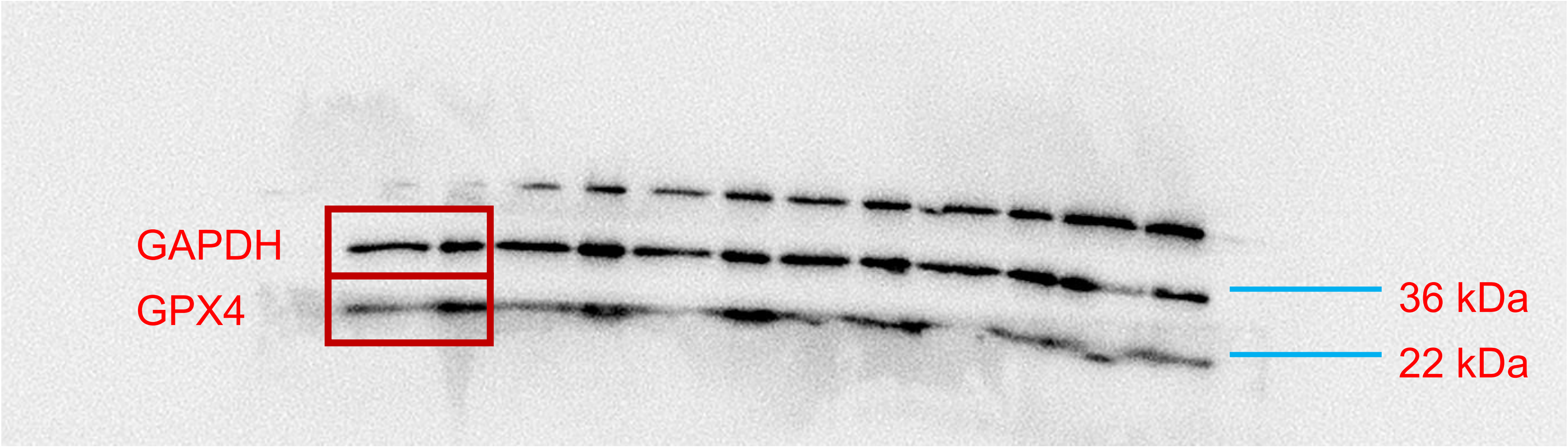

Figure 6.A A549-ACSL4

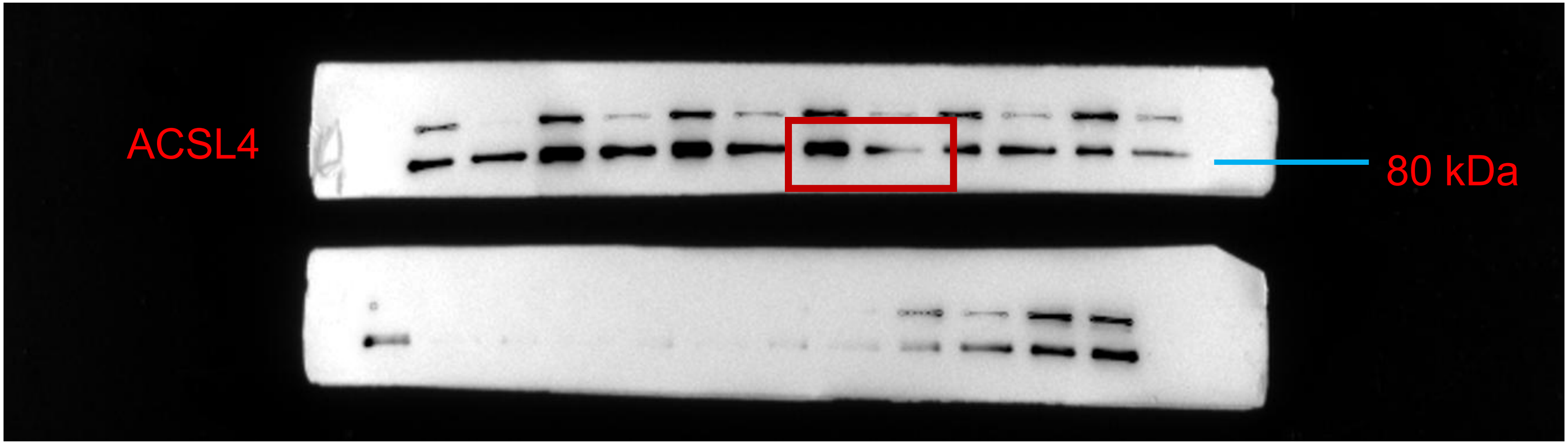

Figure 6.A A549-XCT

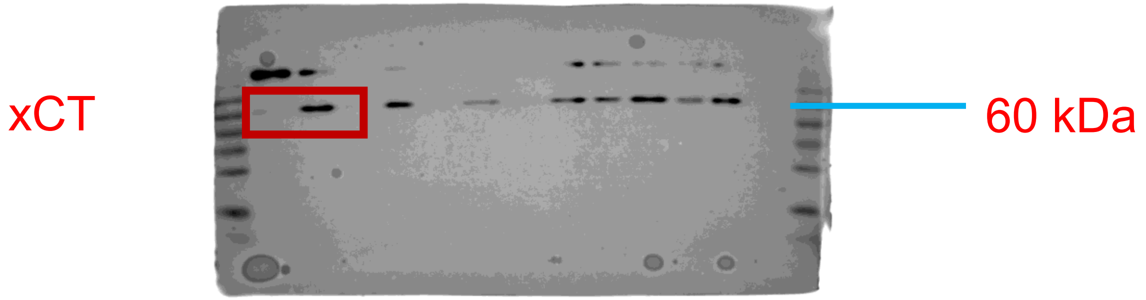

Figure 6.A H460-ACSL4

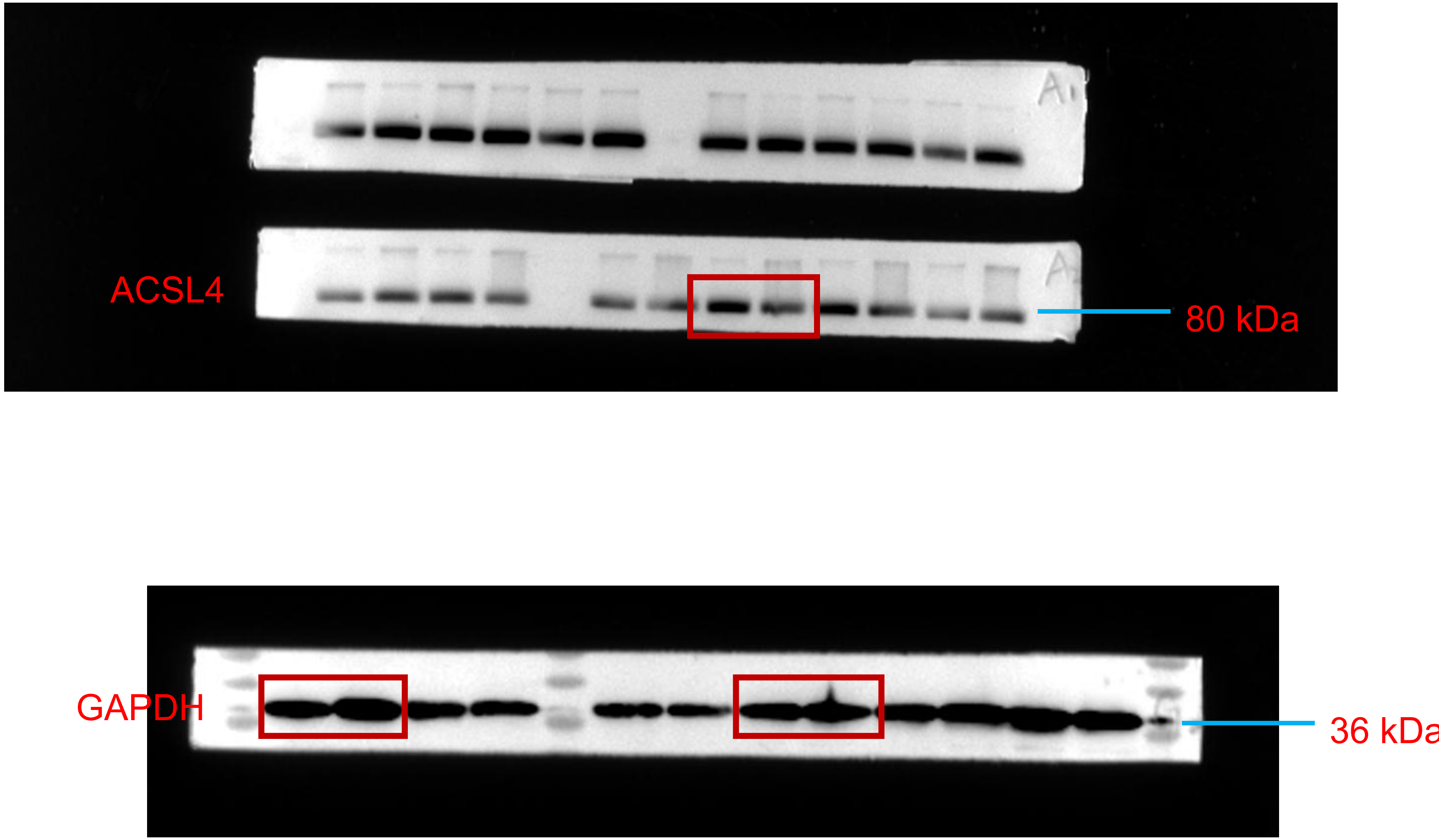

Figure 6.A H460-XCT

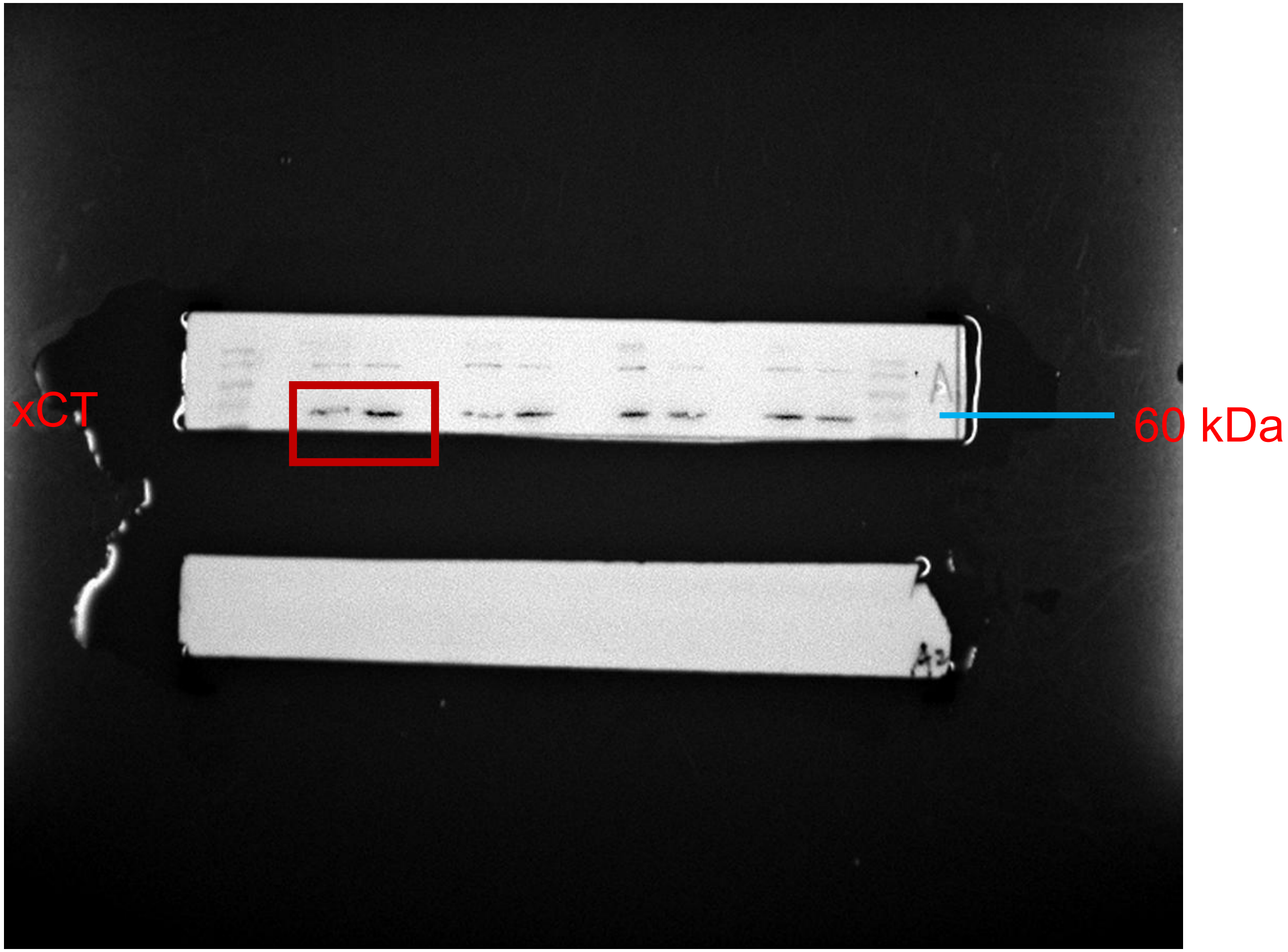

Figure 6.A H460-GPX4

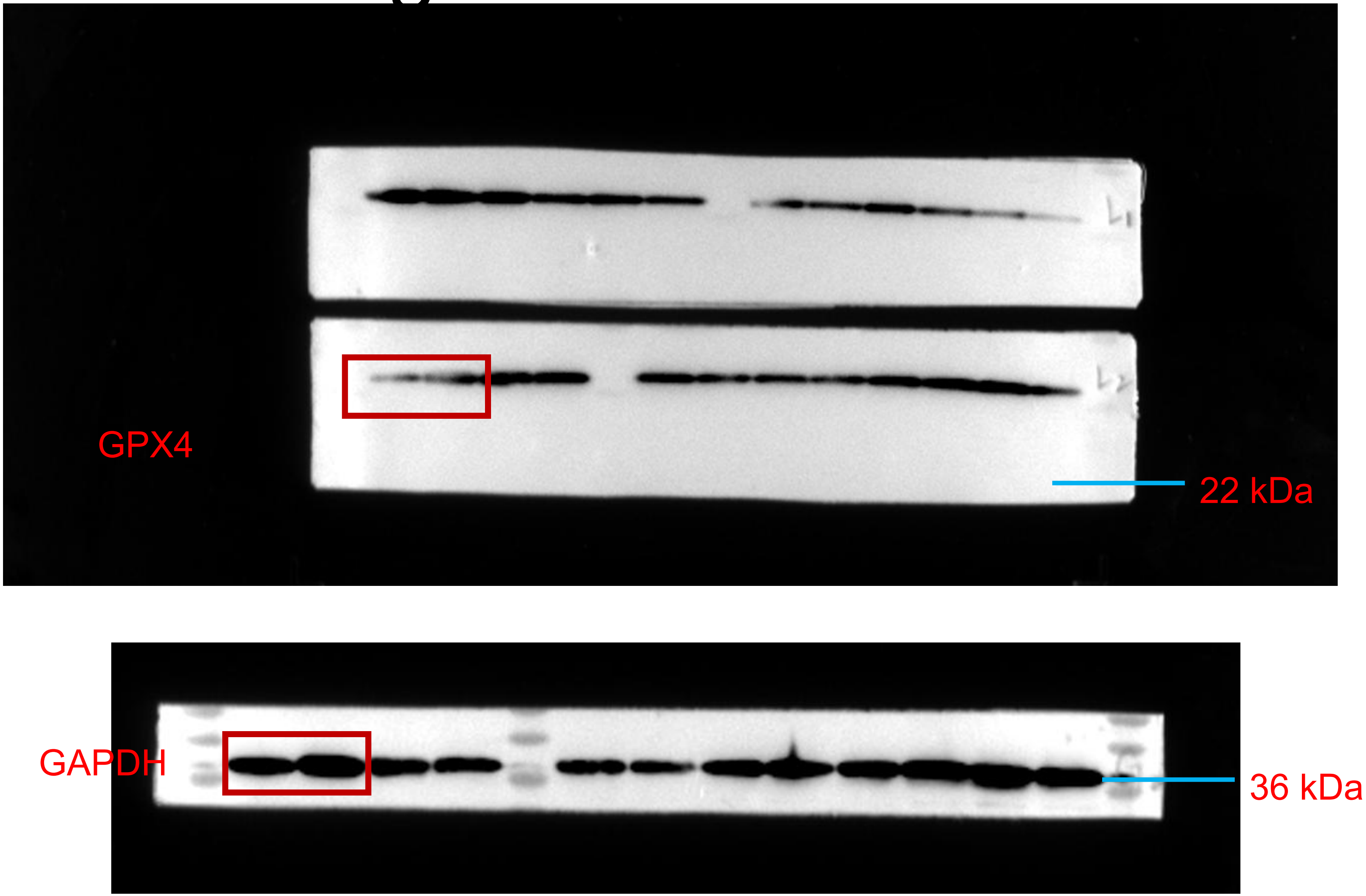

Figure 6.C A549-AMPK

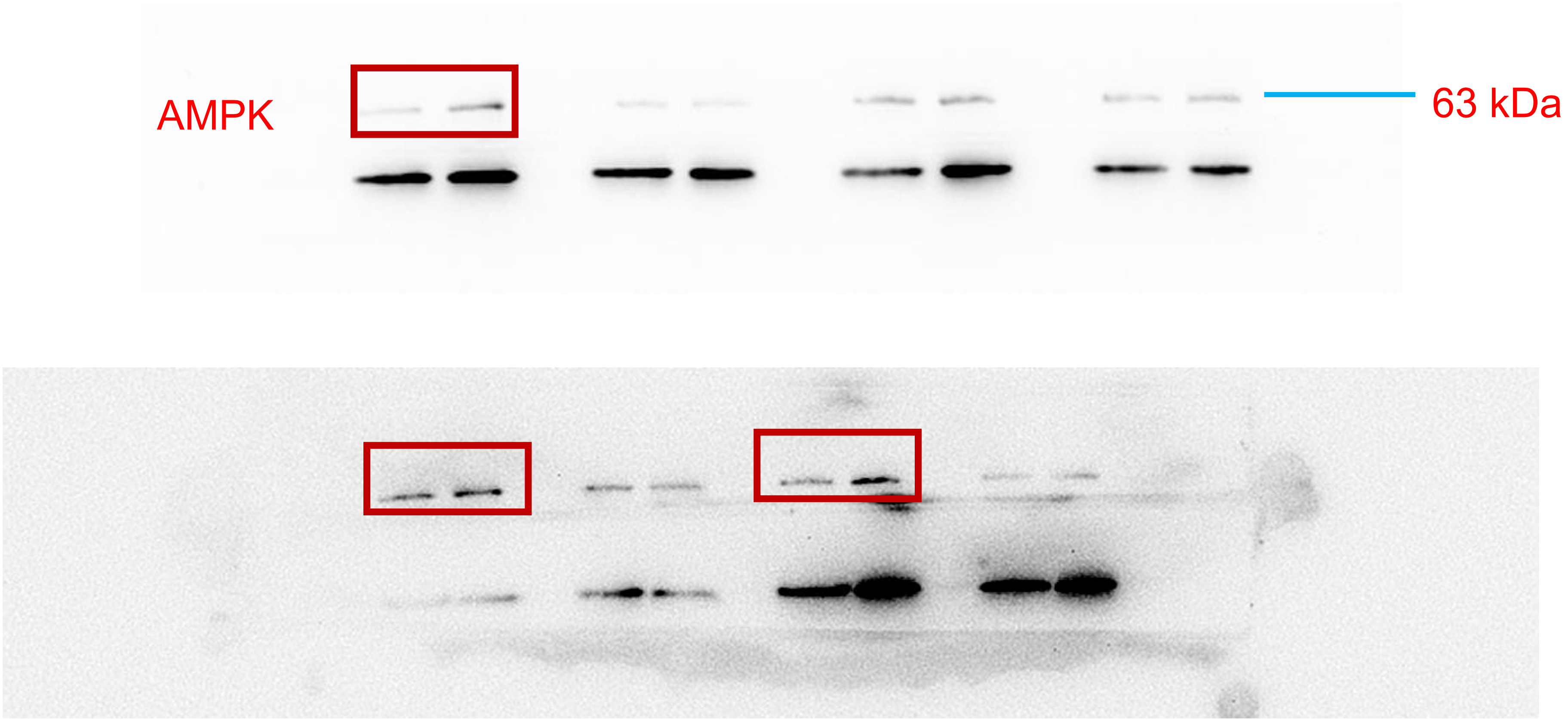

Figure 6.C A549-ACSL4

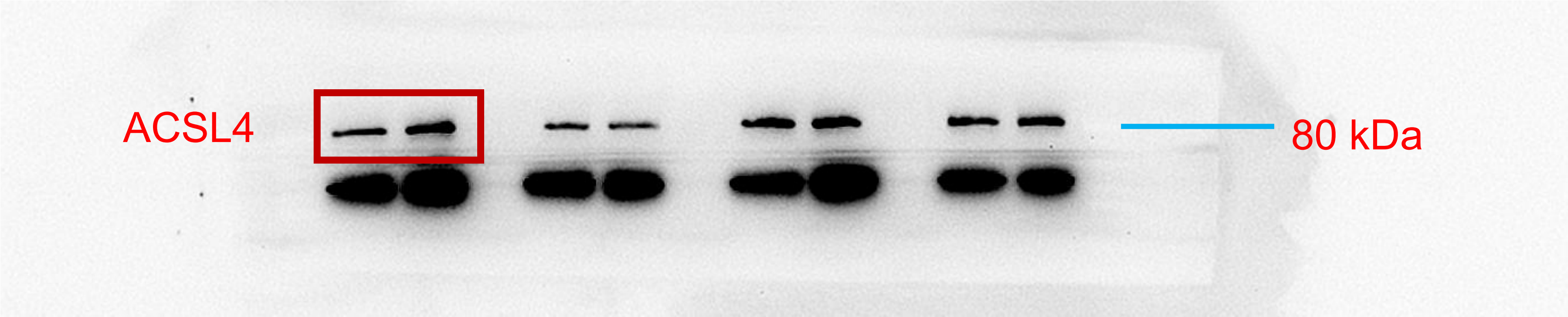

Figure 6.C A549-XCT

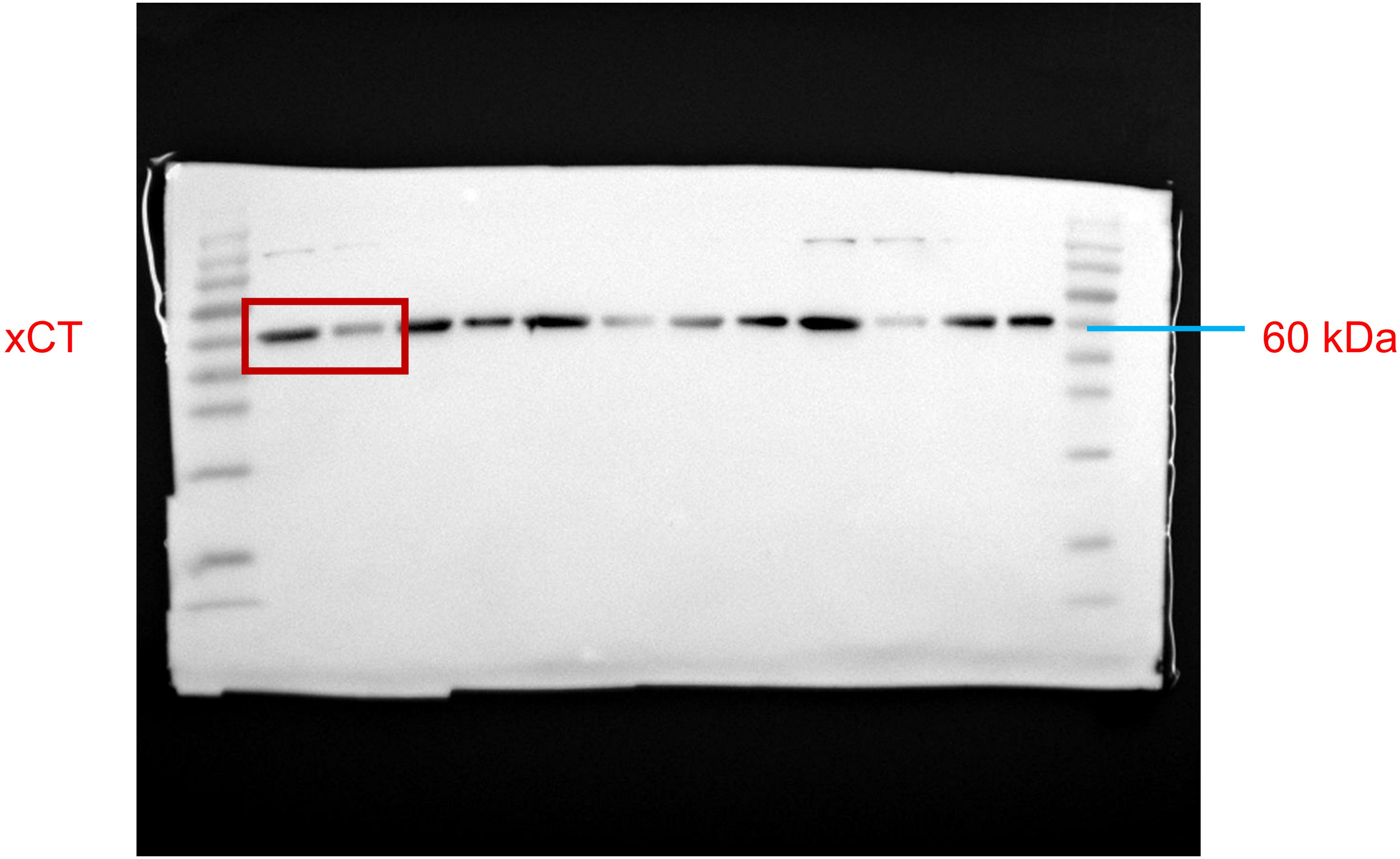

Figure 6.C A549-GPX4

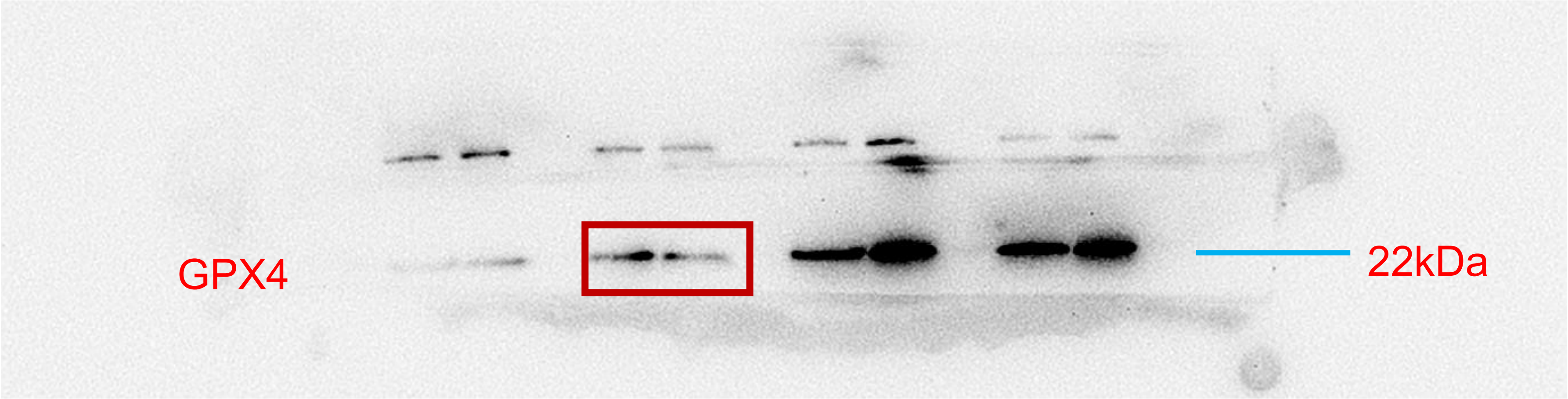

Figure 6.C H460-AMPK

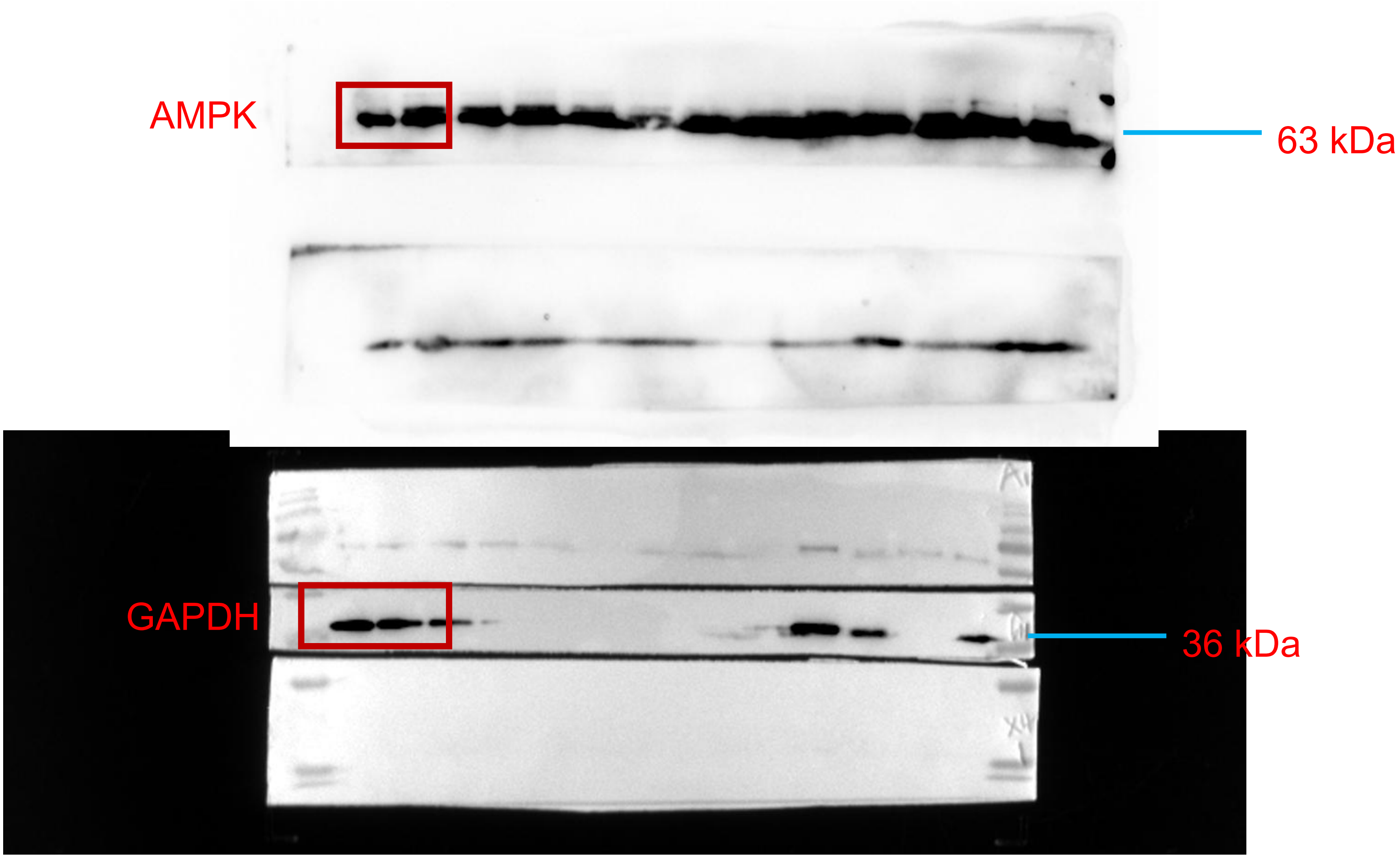

Figure 6.C H460-ACSL4

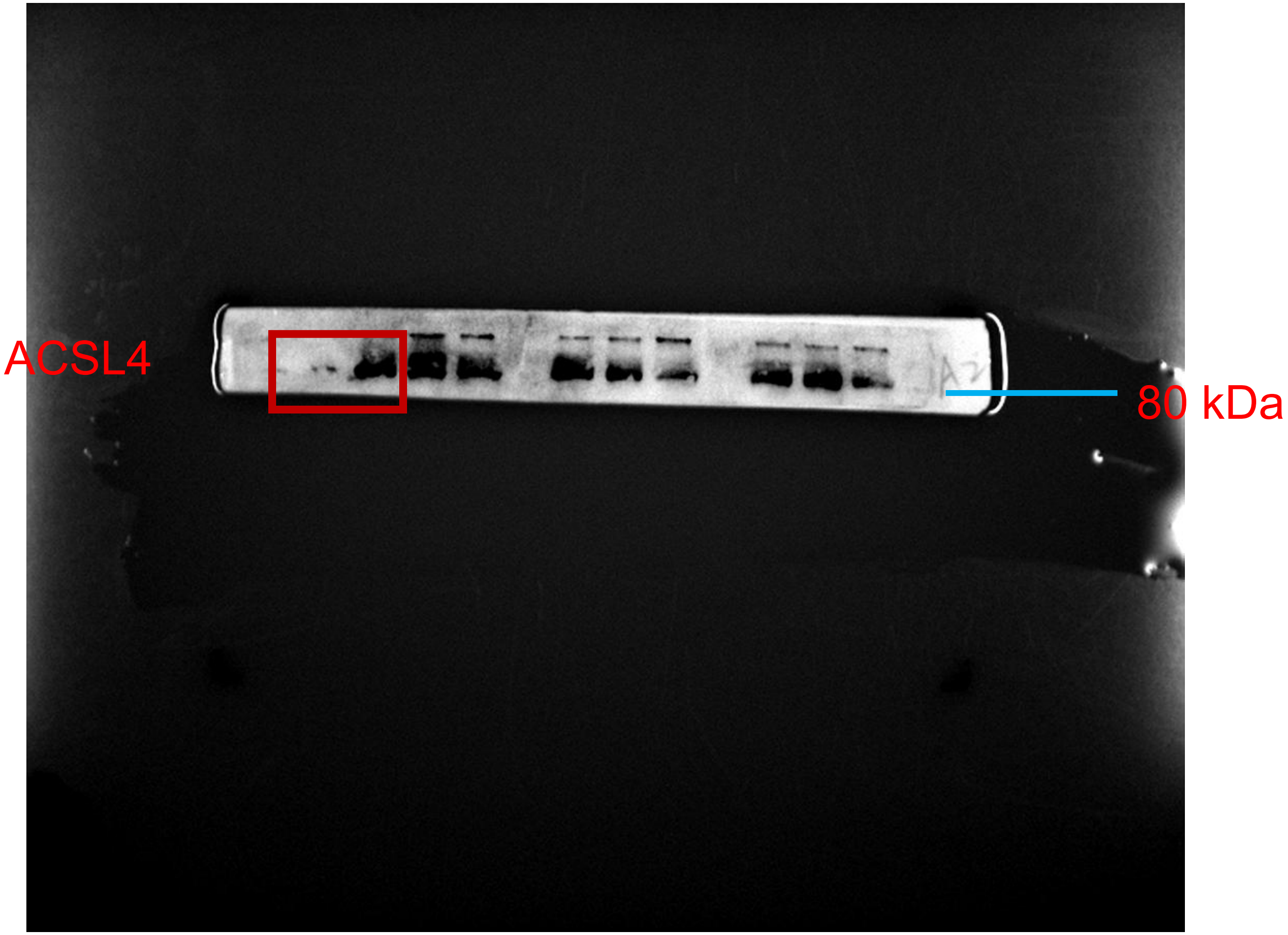

Figure 6.C H460-XCT

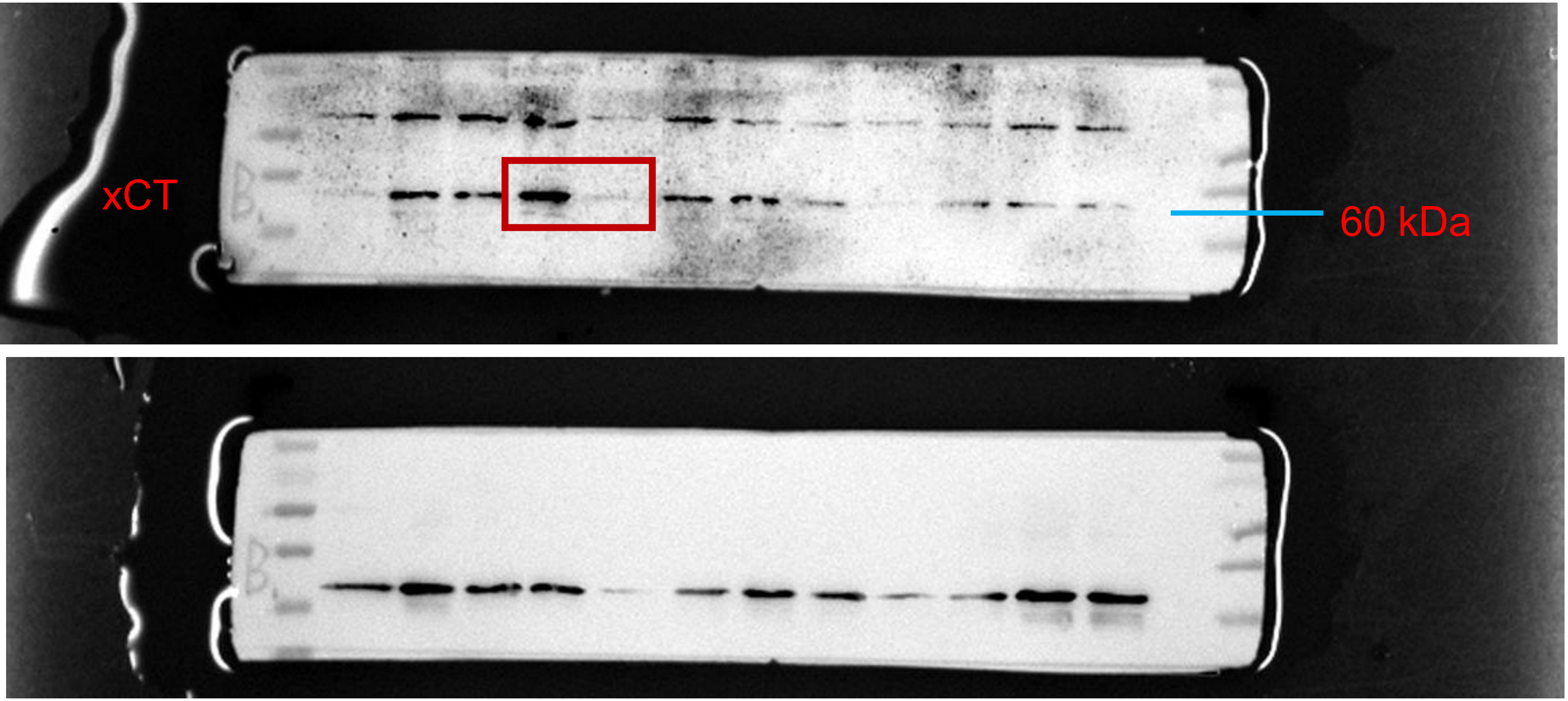

Figure 6.C H460-GPX4

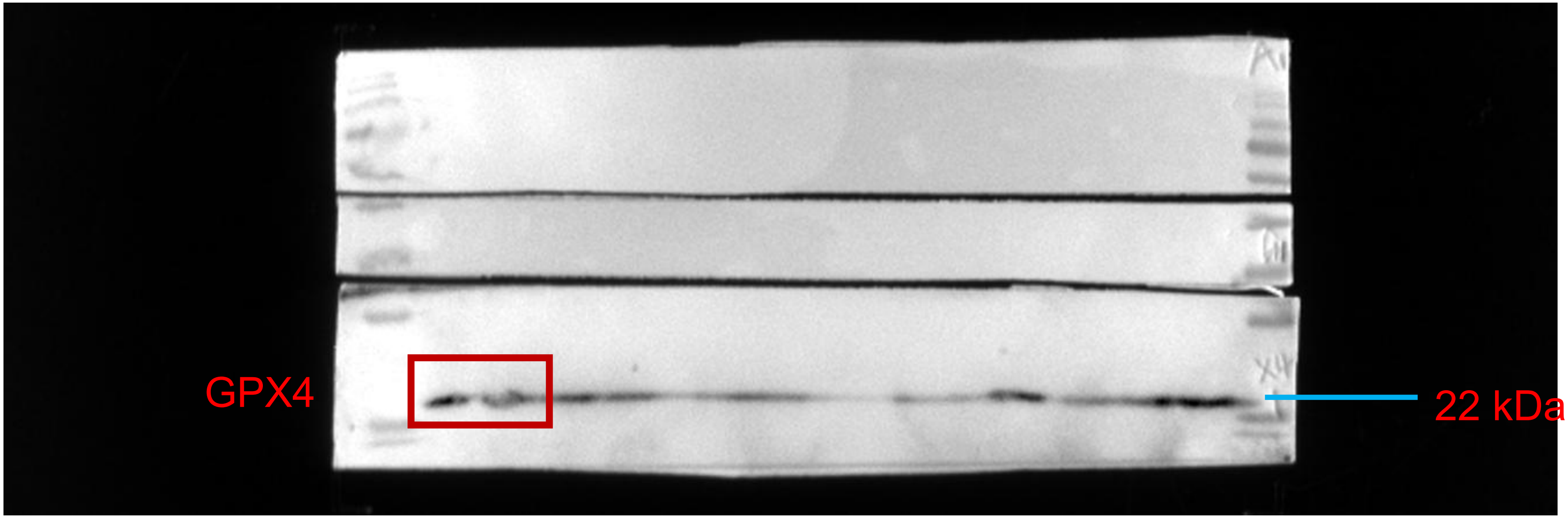

Figure 7

Figure 7A. A549-PDZD8

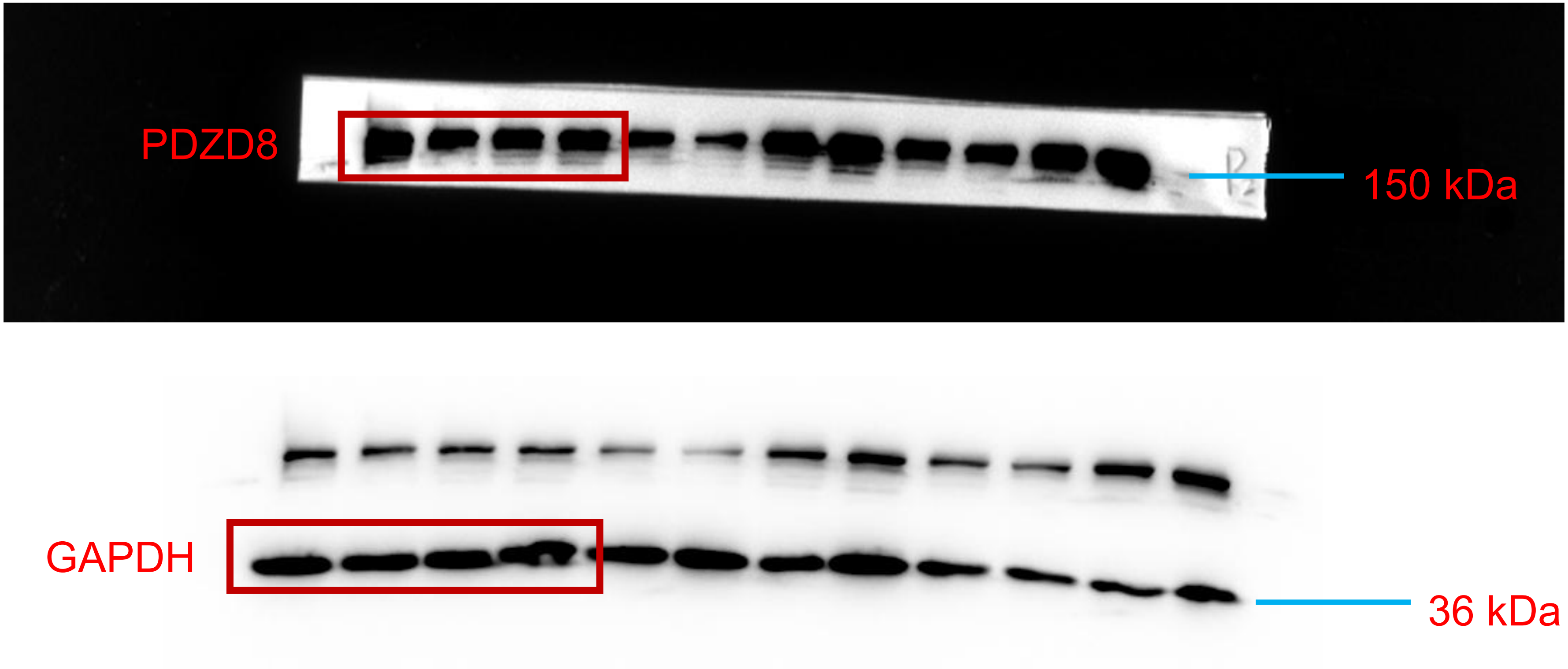

Figure 7A. H460-PDZD8

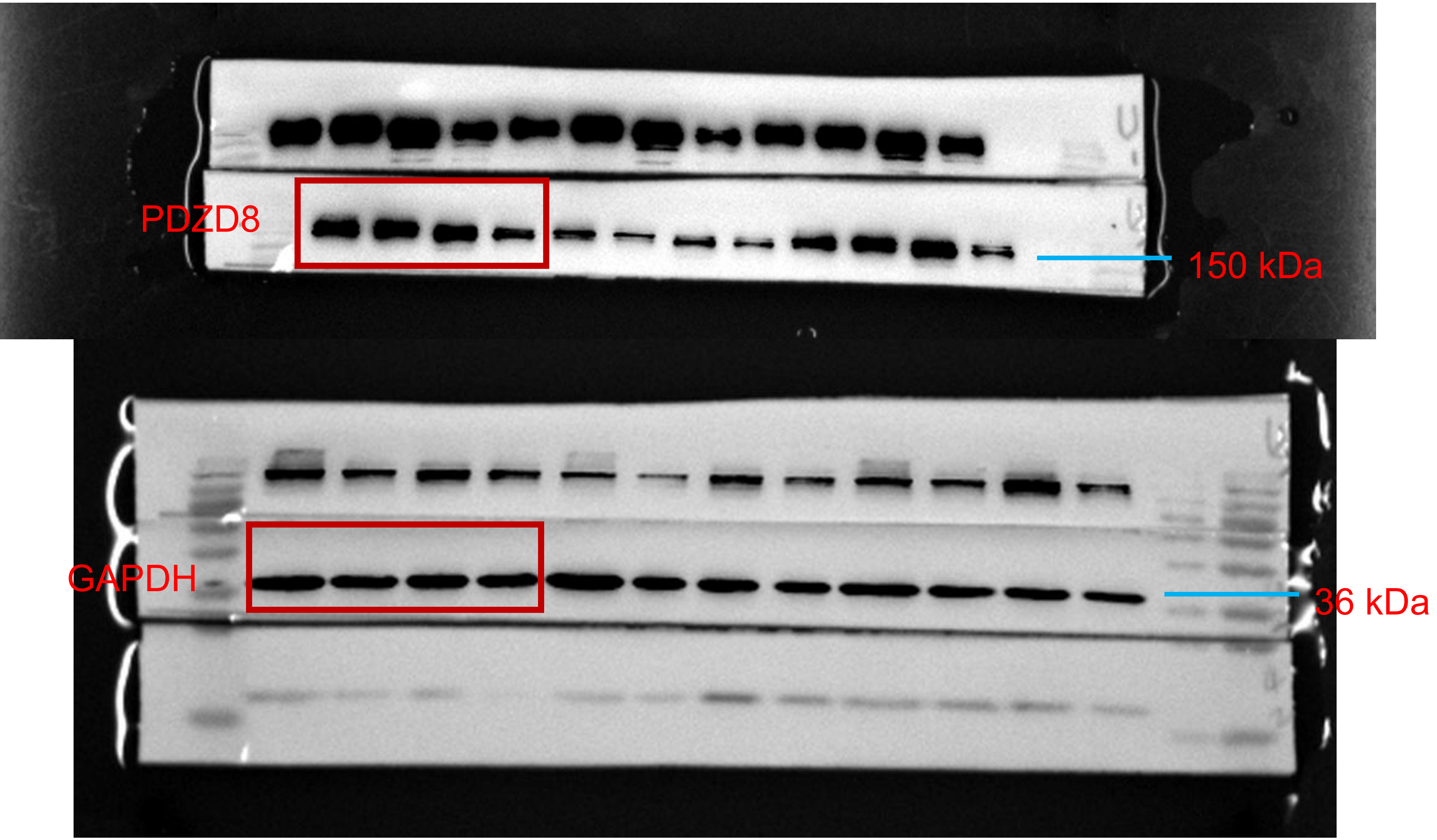

Figure 7B. A549-PDZD8

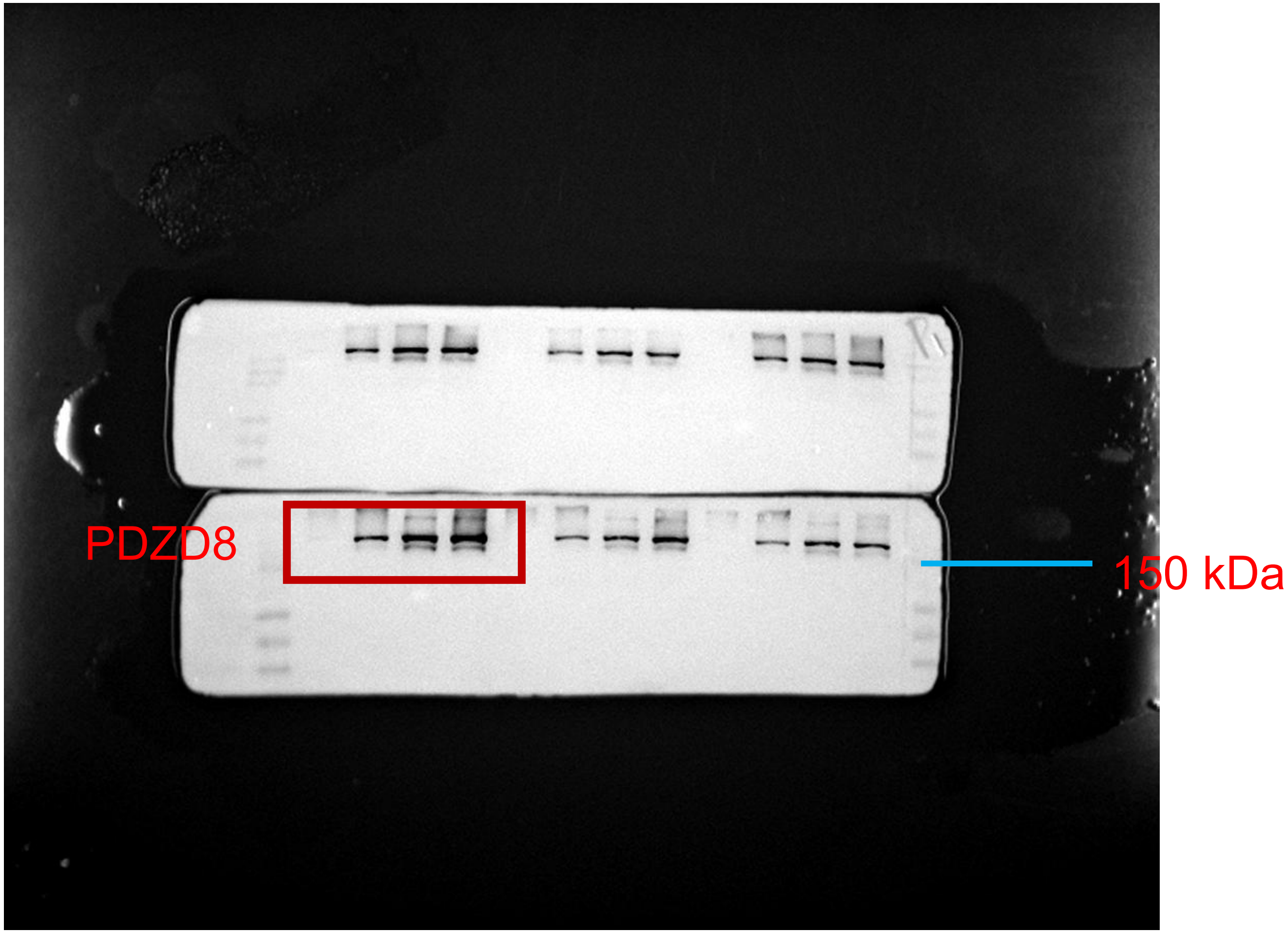

Figure 7B.H460-PDZD8

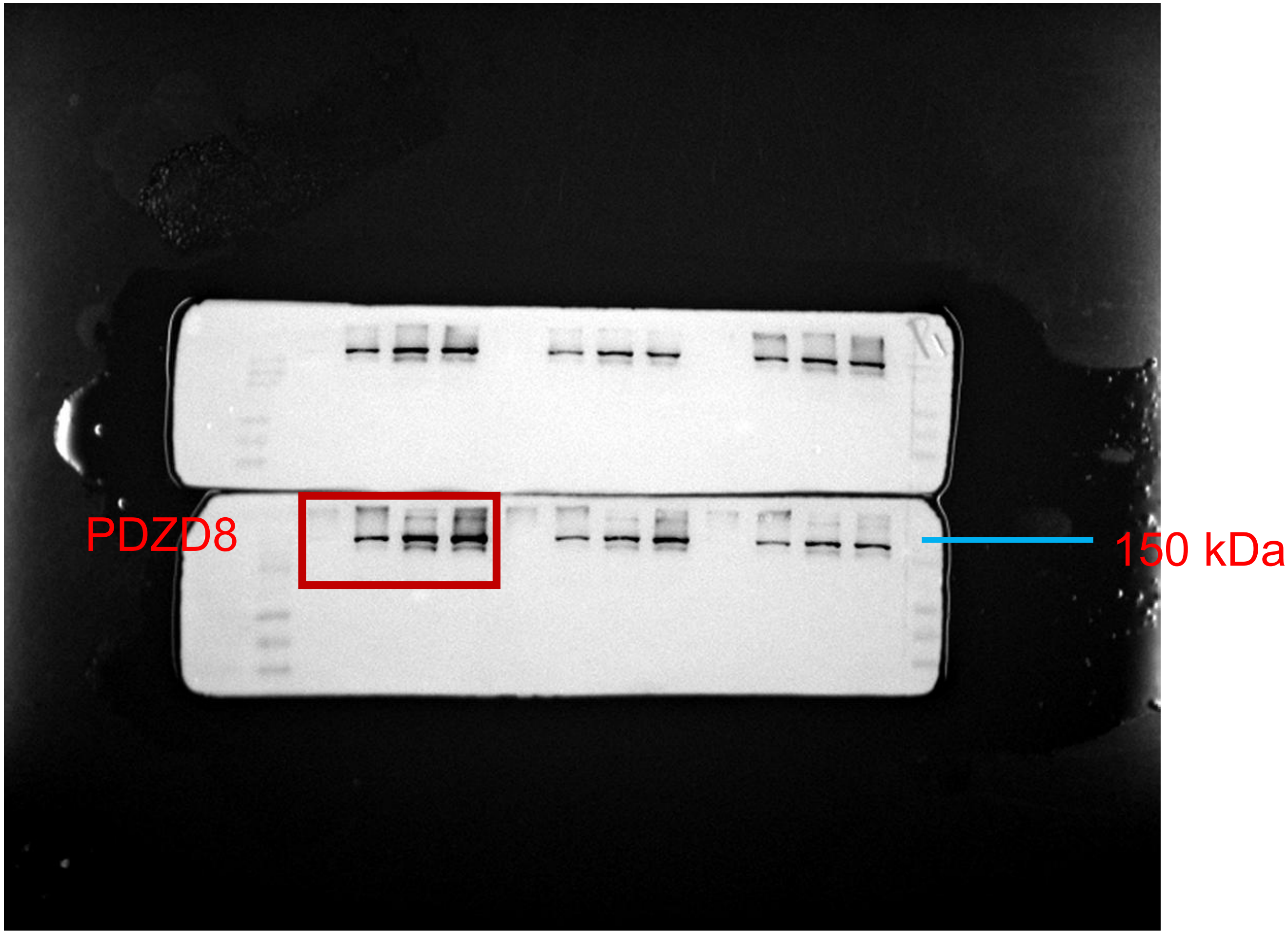

Figure 7C.A549-PDZD8

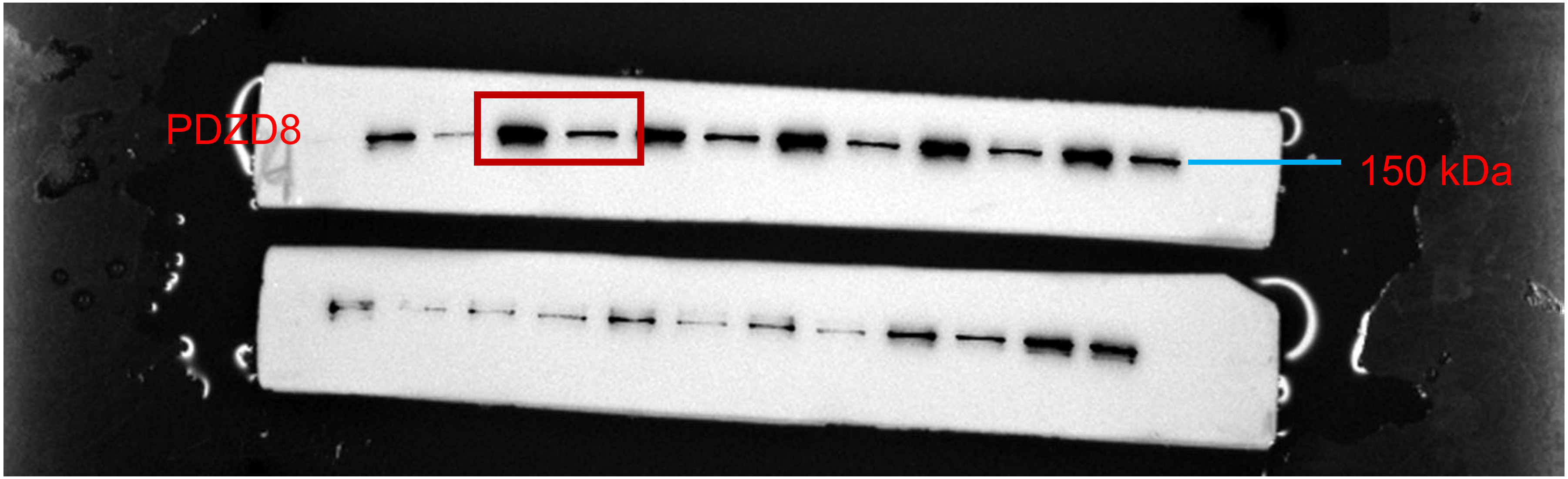

Figure 7C.H460-PDZD8 9.28

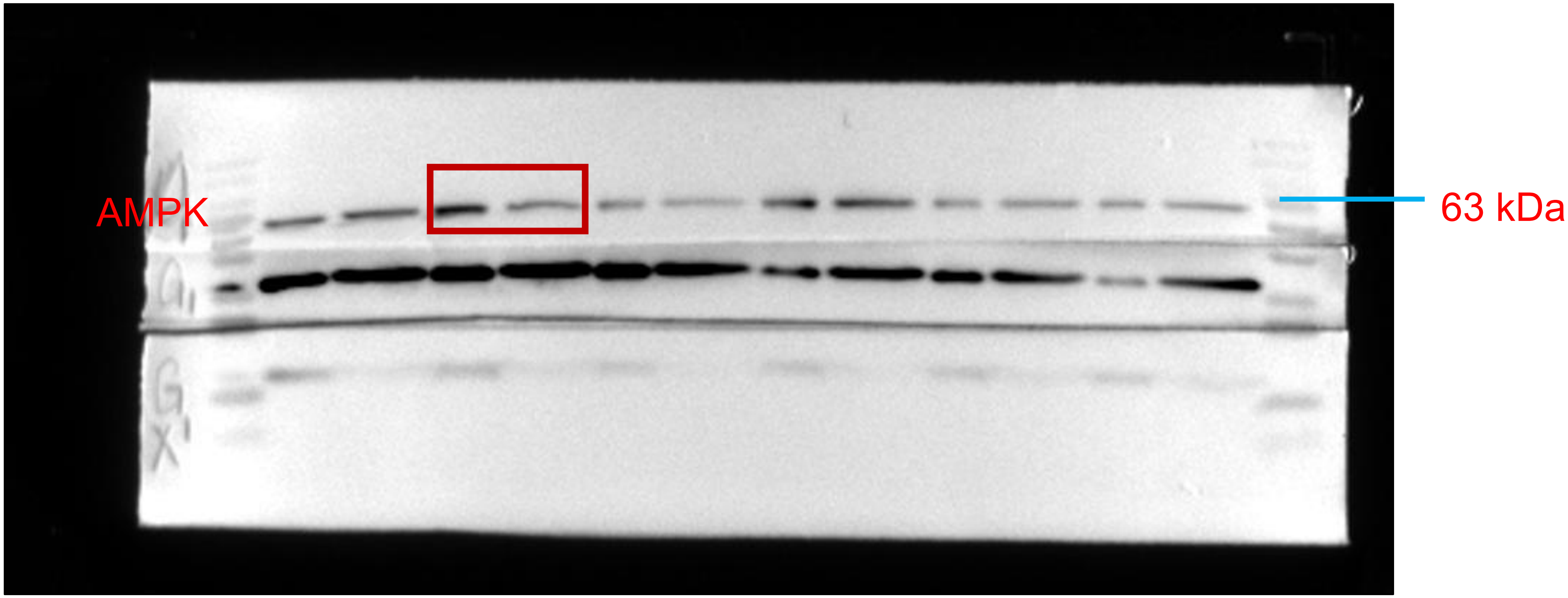

Figure 7D.A549-PDZD8

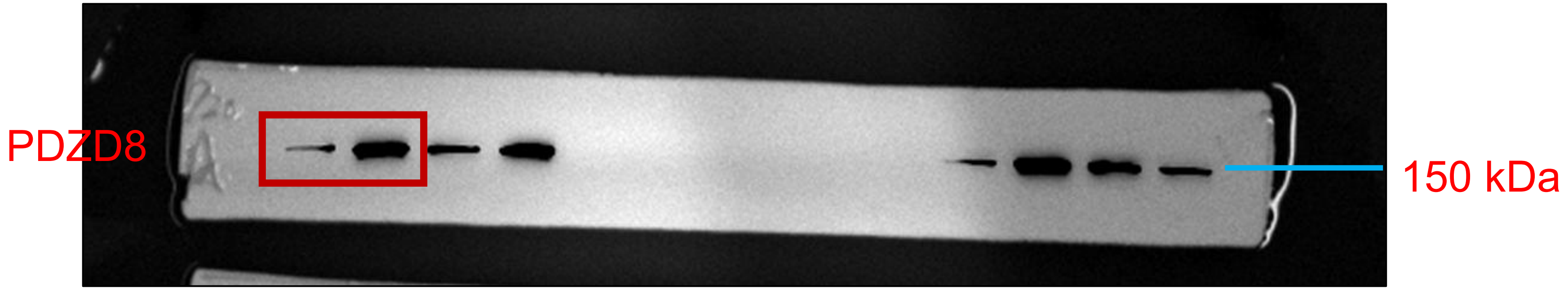

Figure 7D.H460-PDZD8

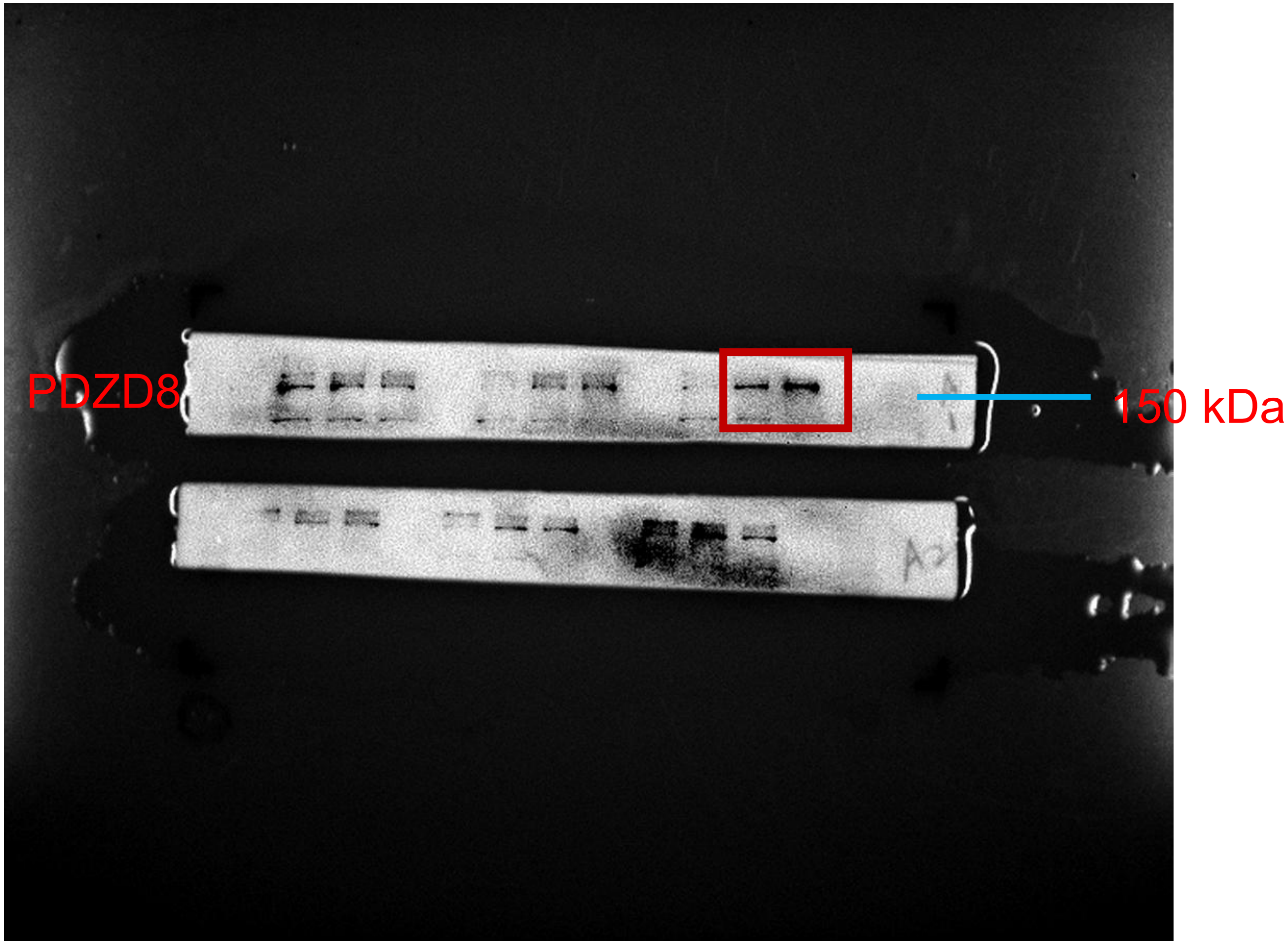

Figure 8

Figure 8A. A549-PDZD8

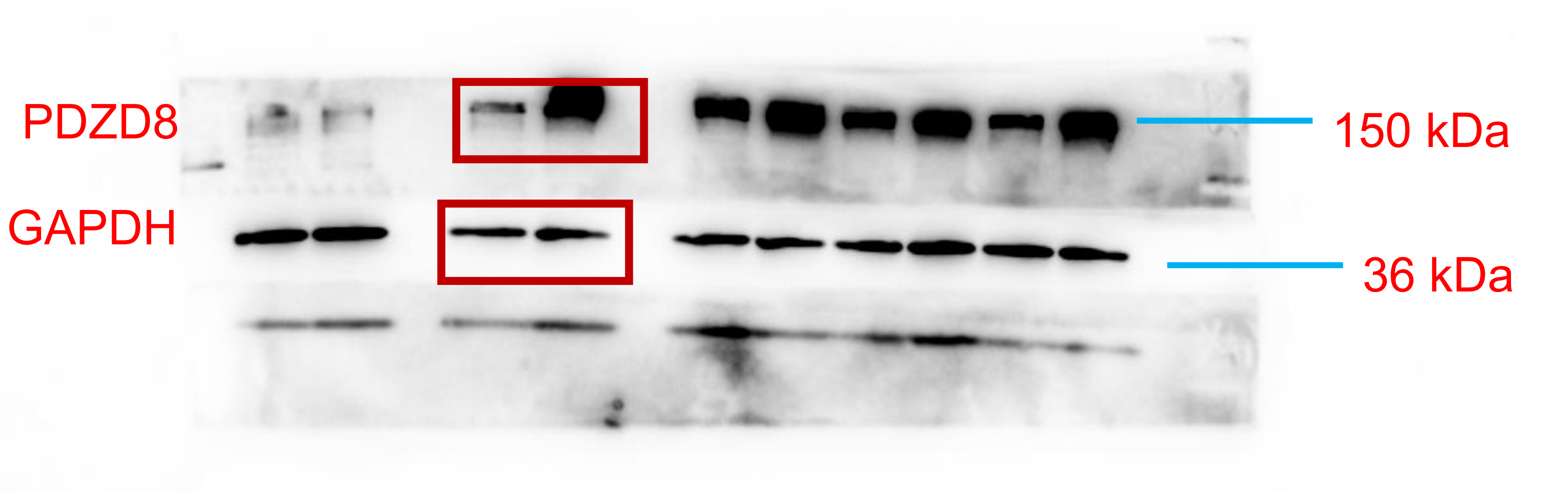

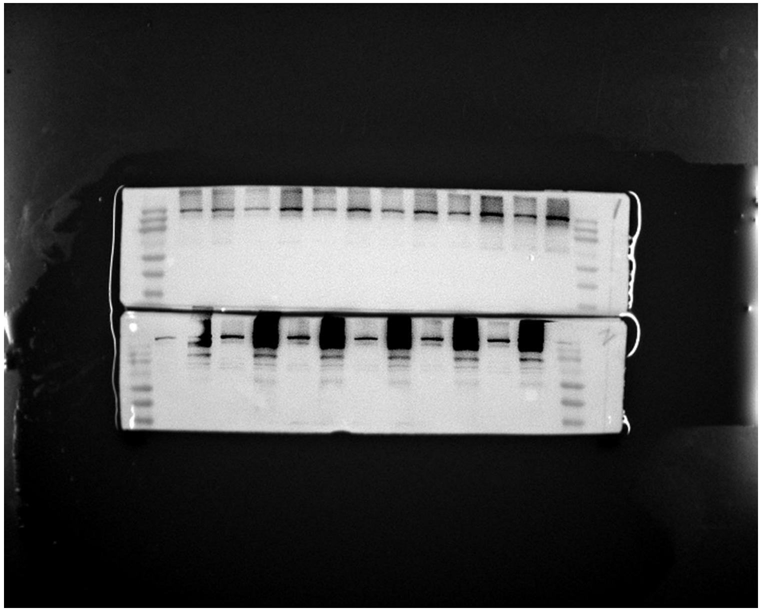

Figure 8A. A549-ULK1

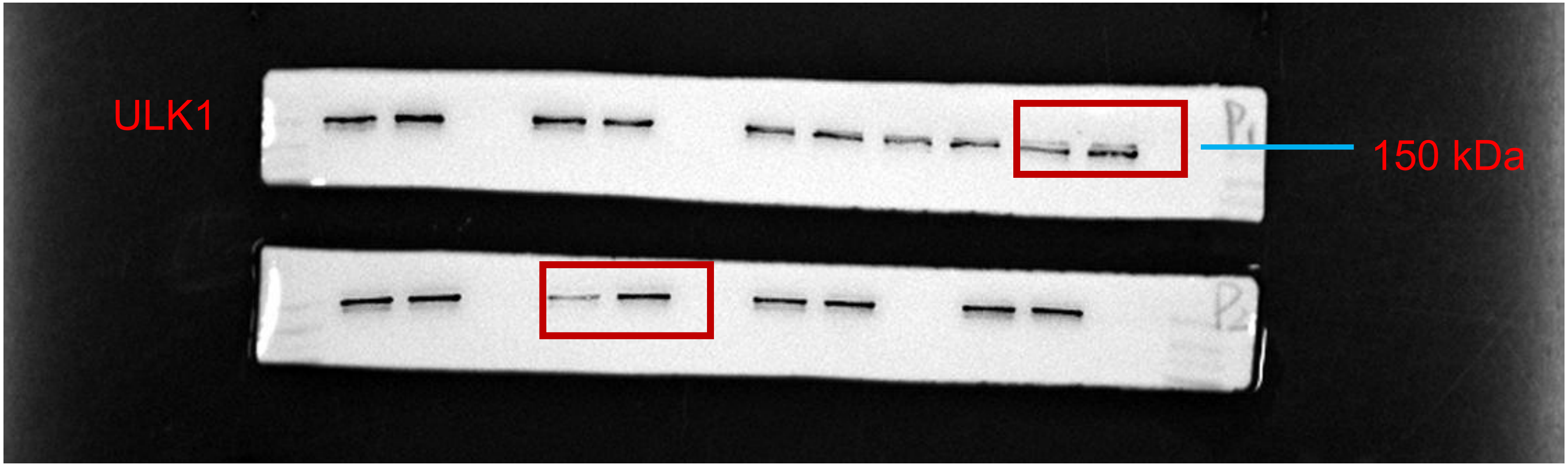

1

Figure 8A. A549-BECN1

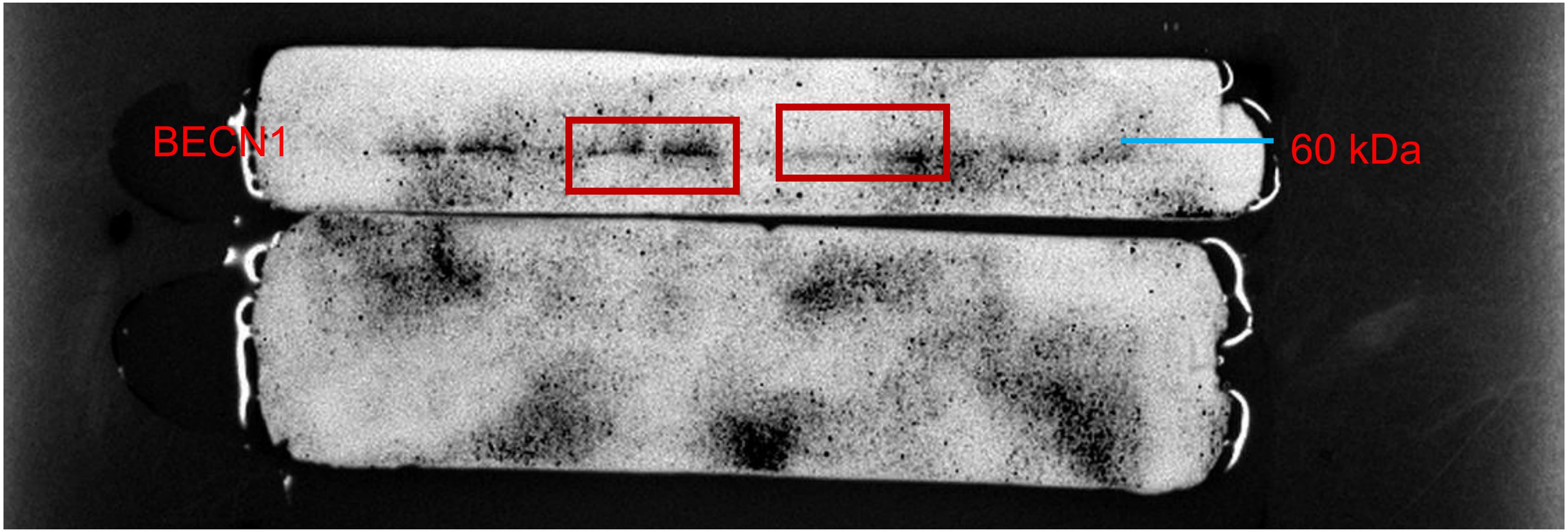

Figure 8A. A549-NCOA4

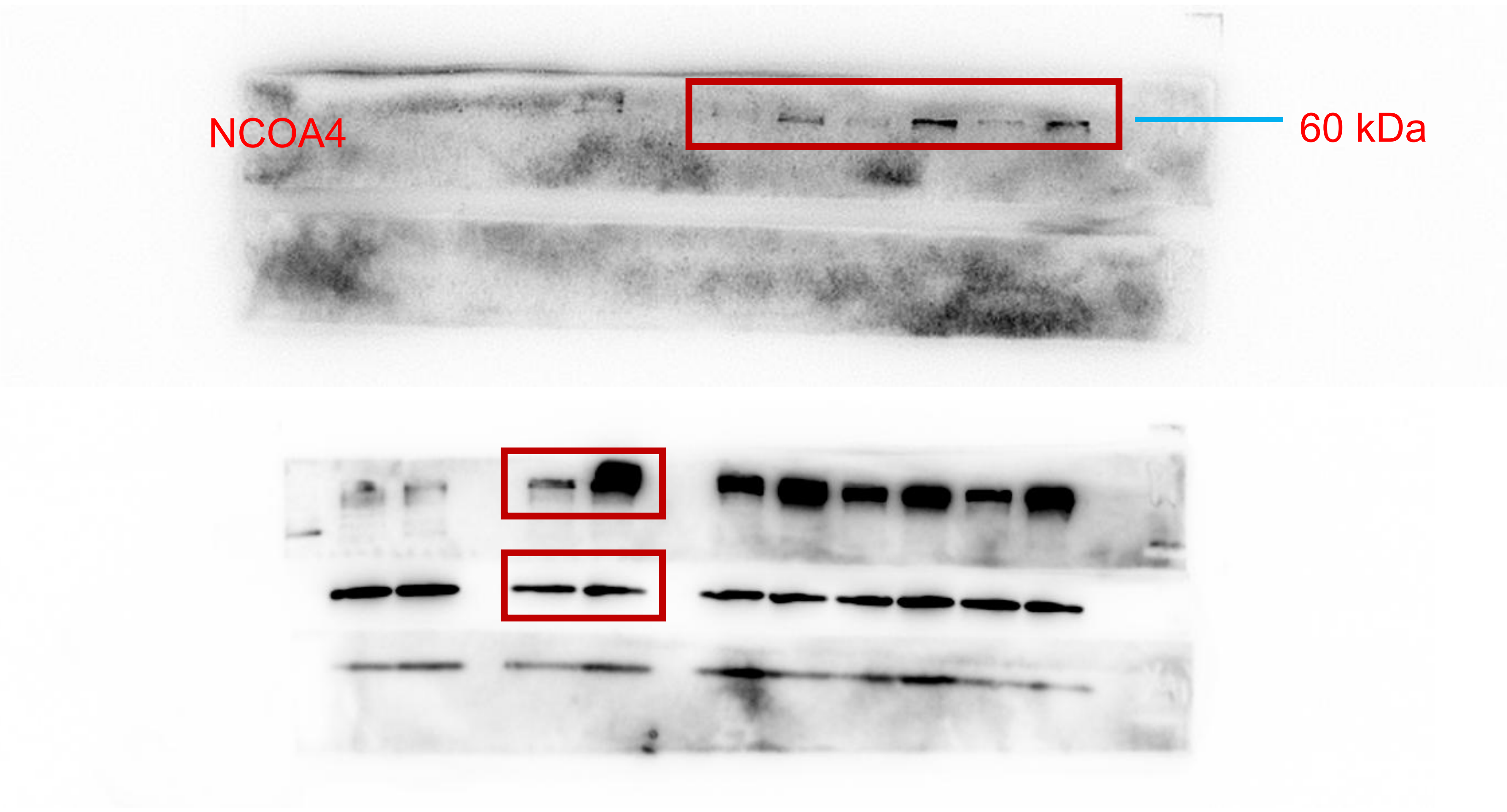

Figure 8A. A549-LC3B

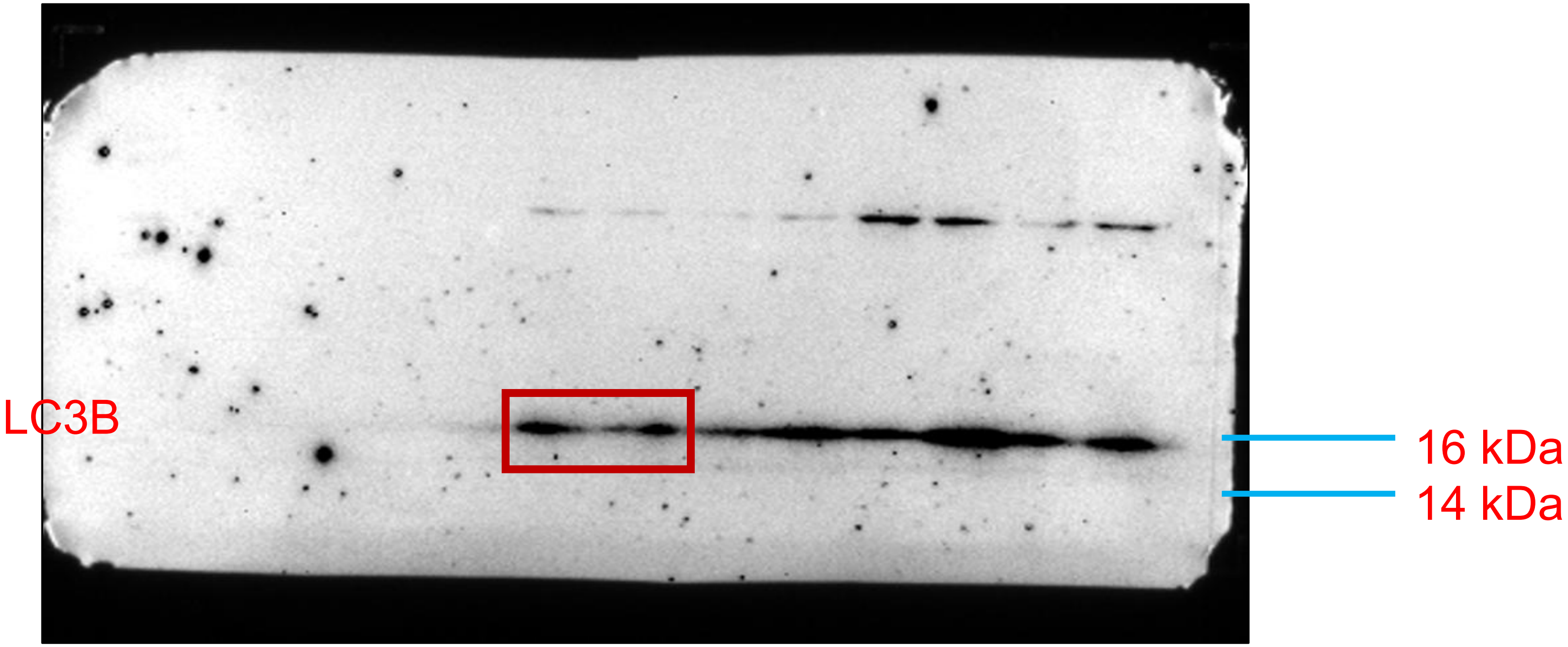

Figure 8A. H460-PDZD8

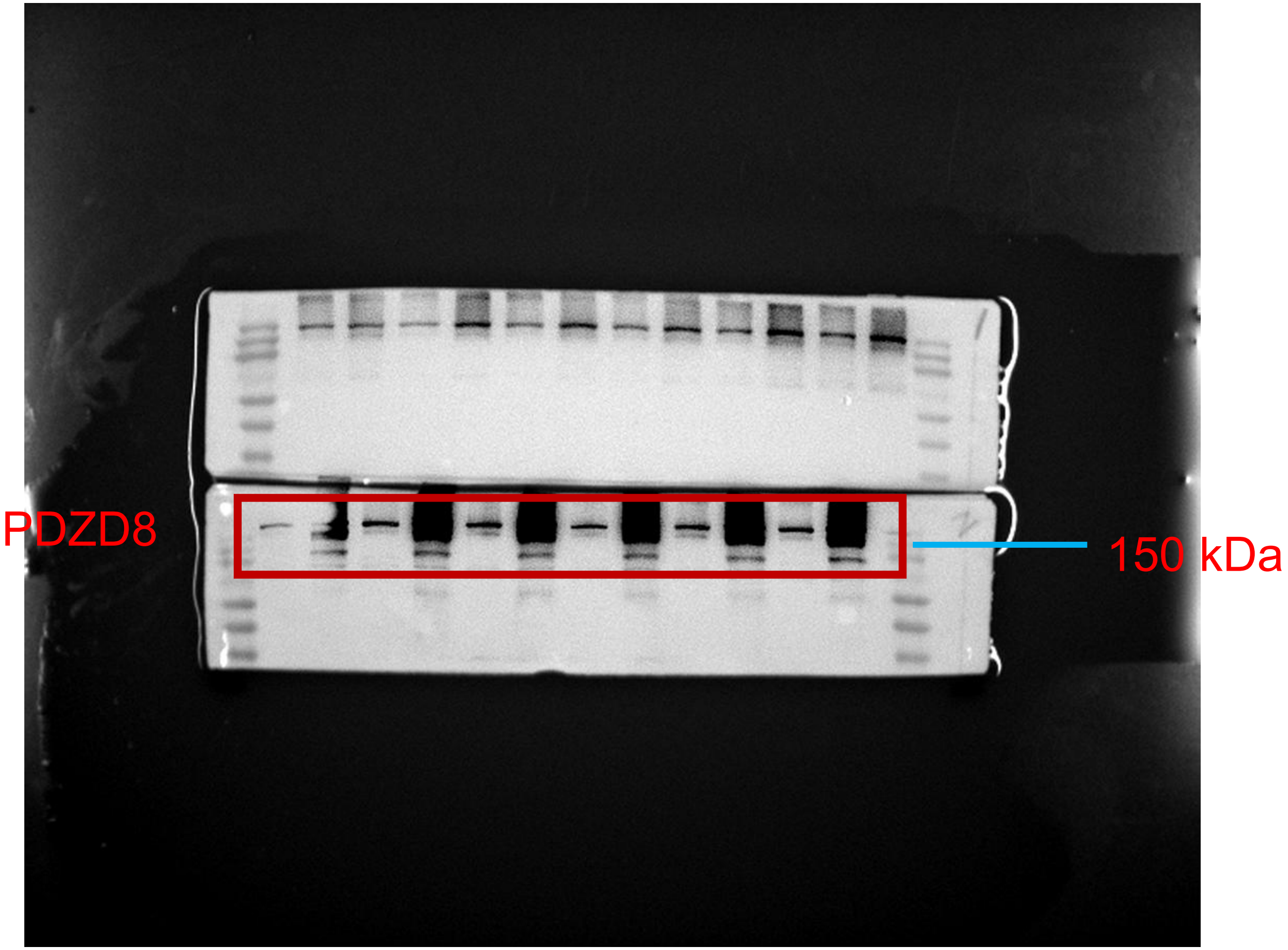

Figure 8A. H460-ULK1

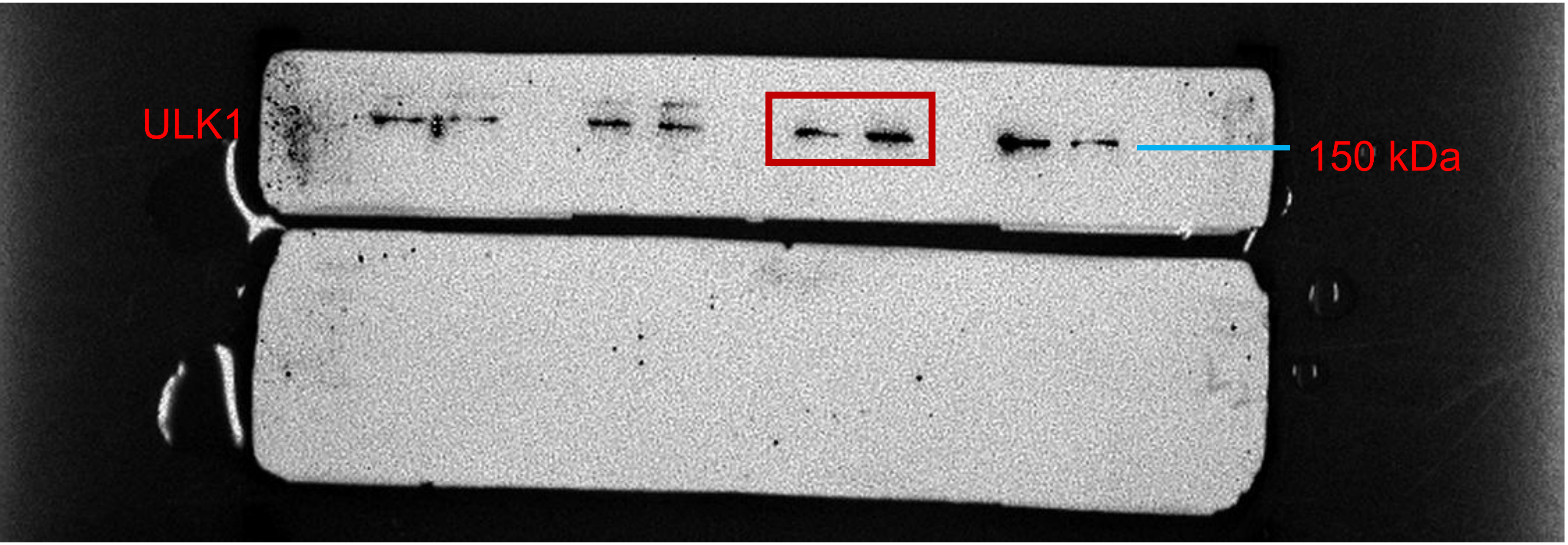

Figure 8A. H460-BECN1

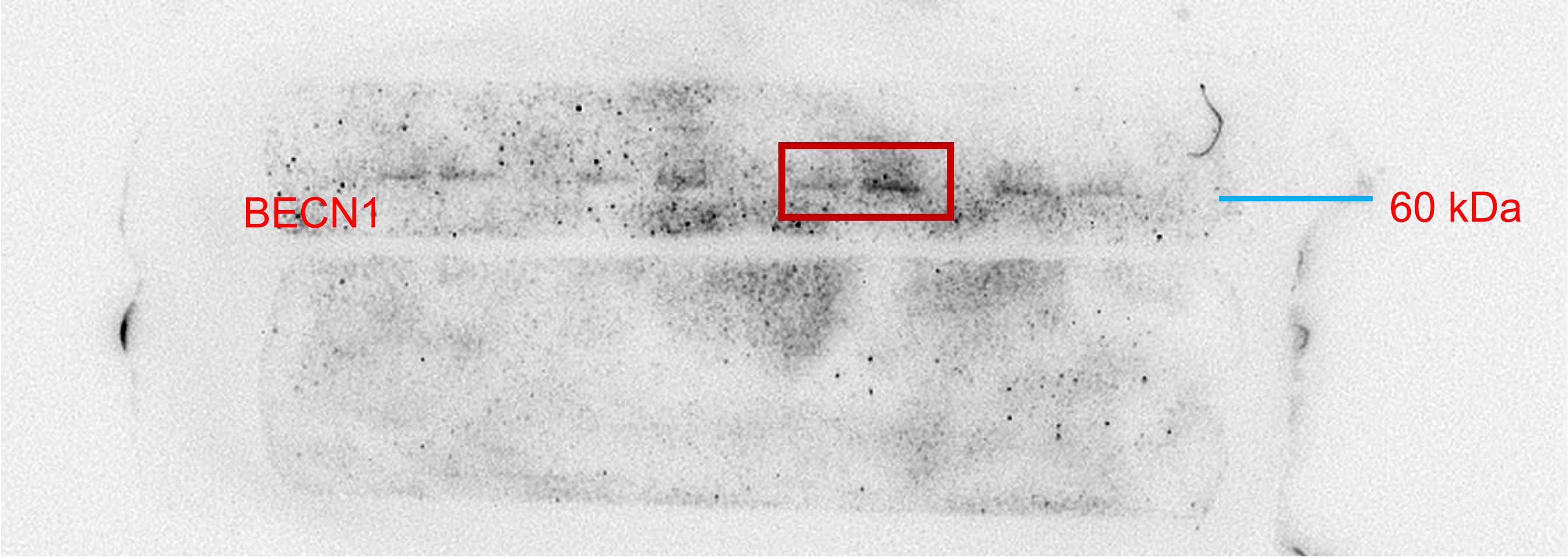

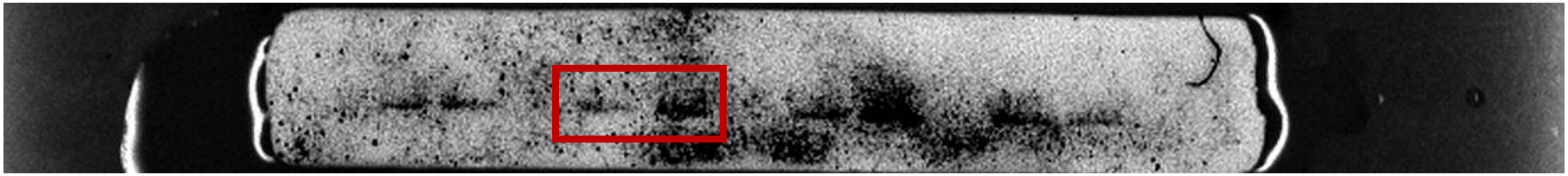

Figure 8A. H460-LC3B

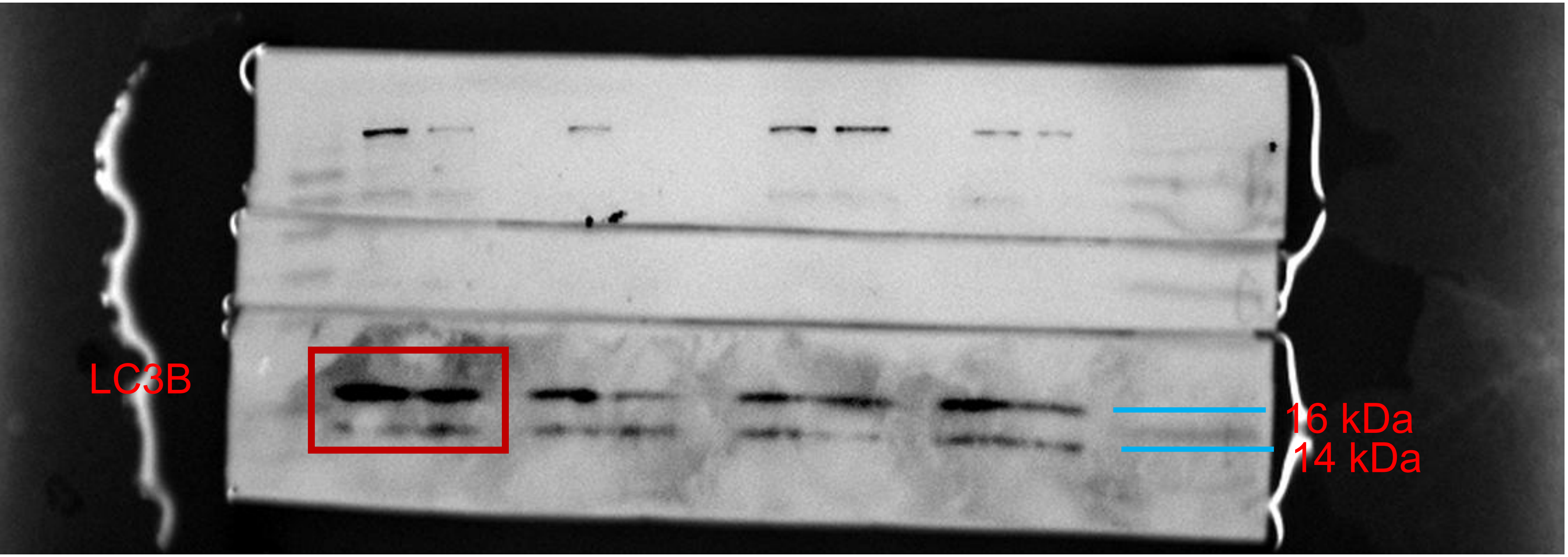

Figure 8A. H460-NCOA4

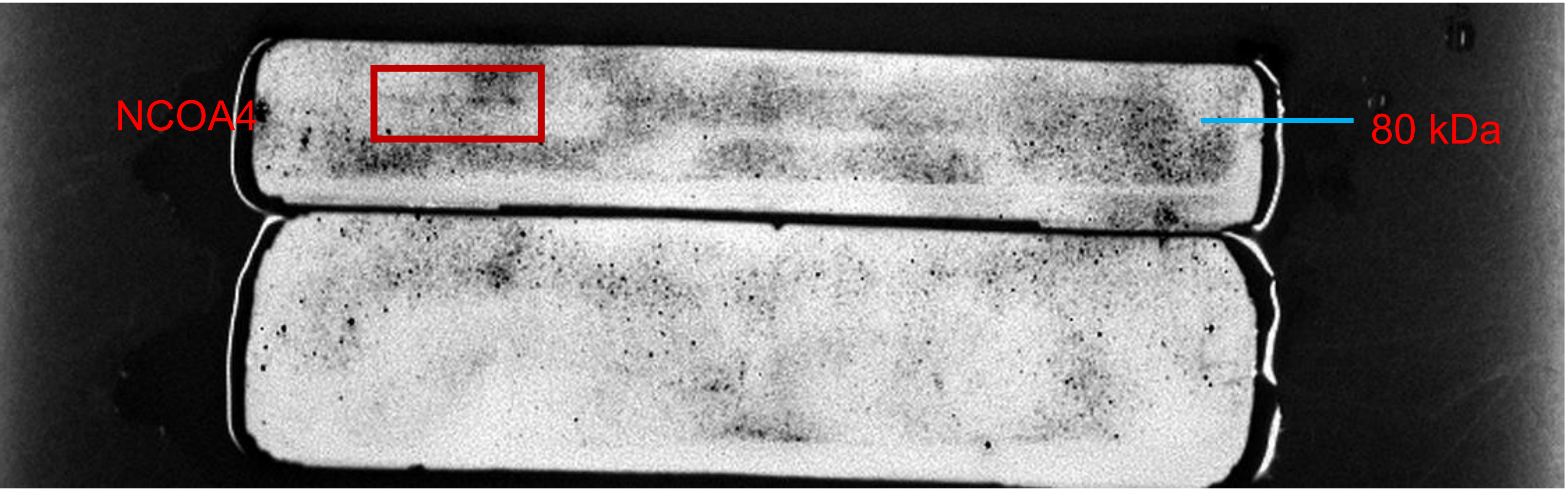

Figure 8B. A549-PDZD8

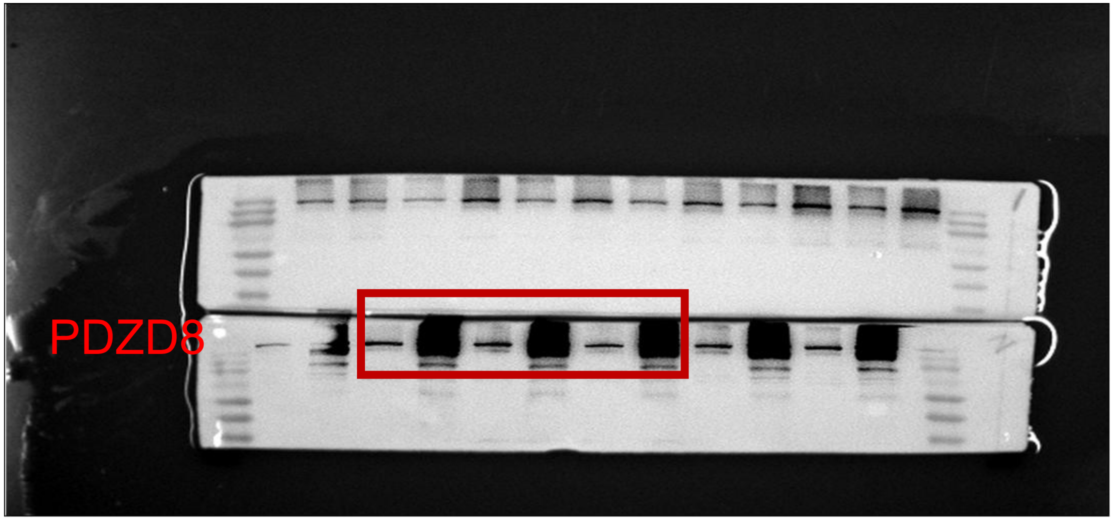

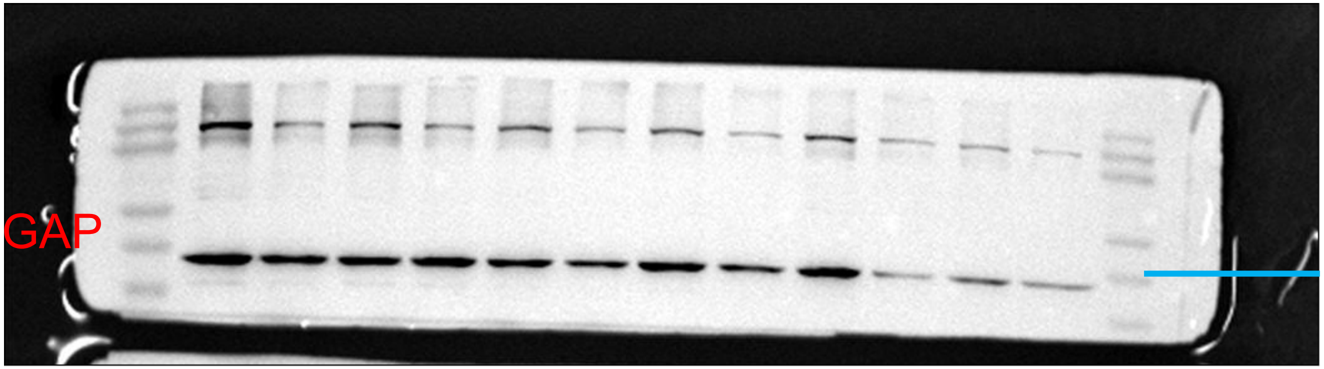

Figure 8B. A549-ACSL4

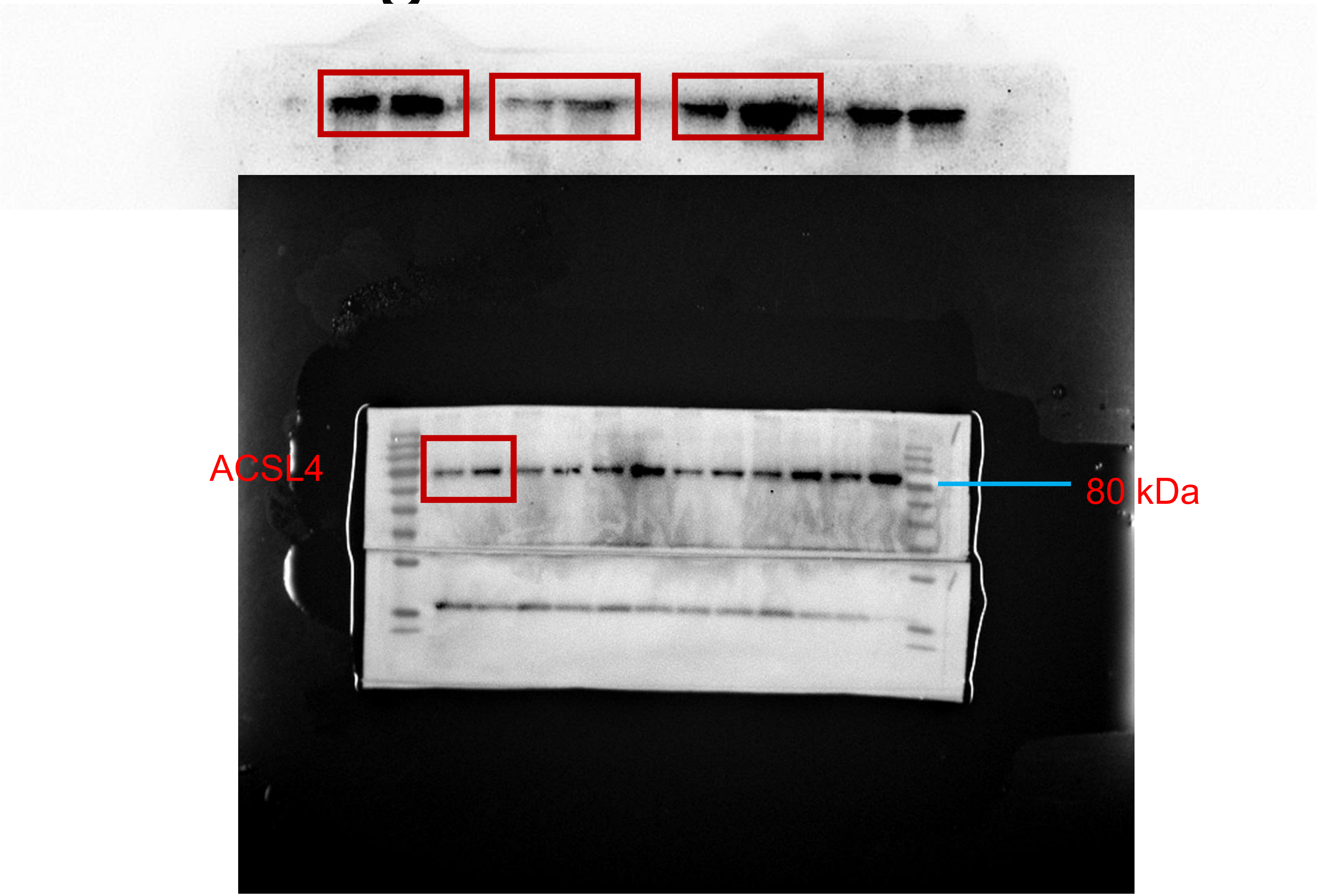

Figure 8B. A549-XCT

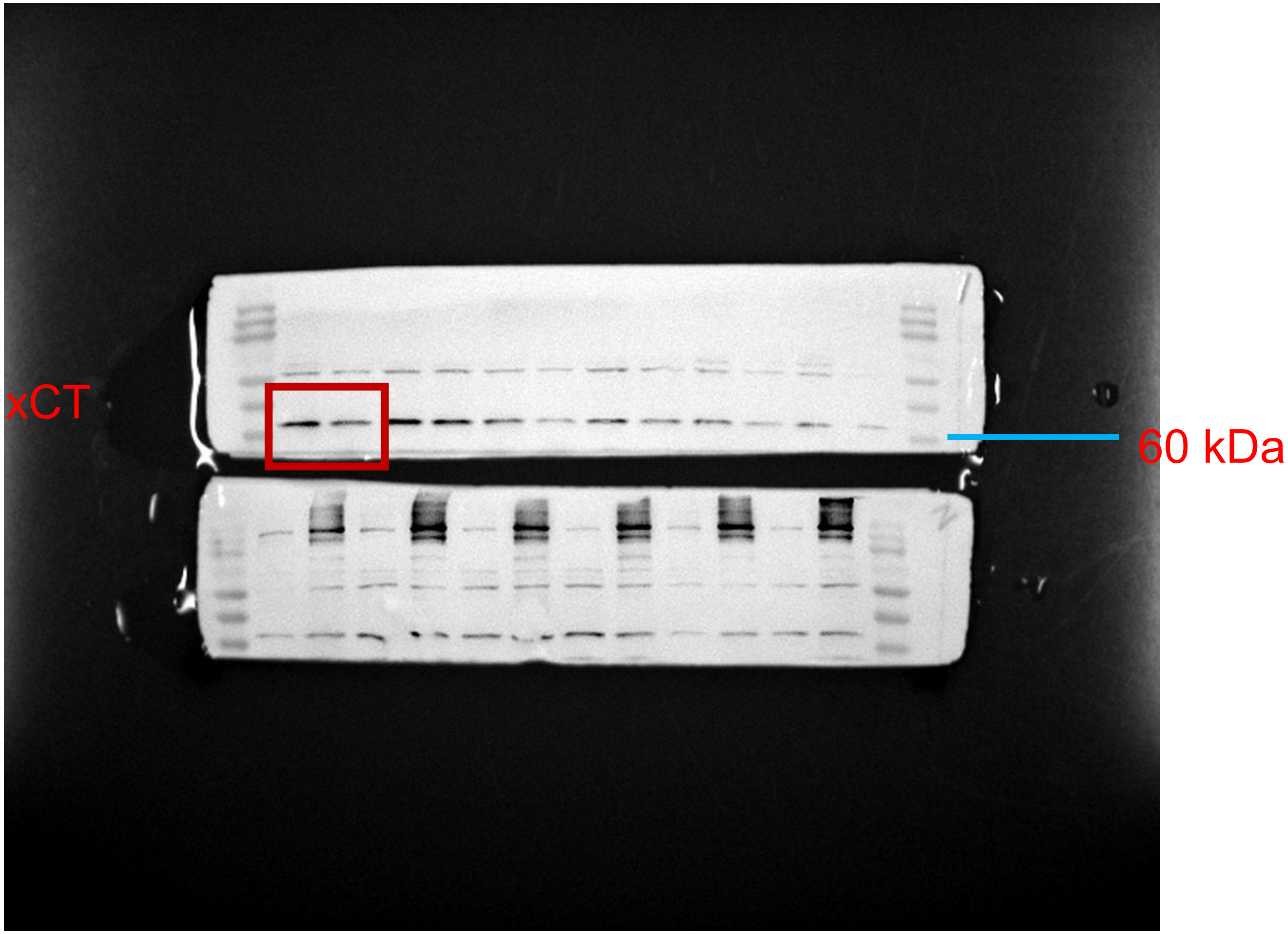

Figure 8B. A549-GPX4

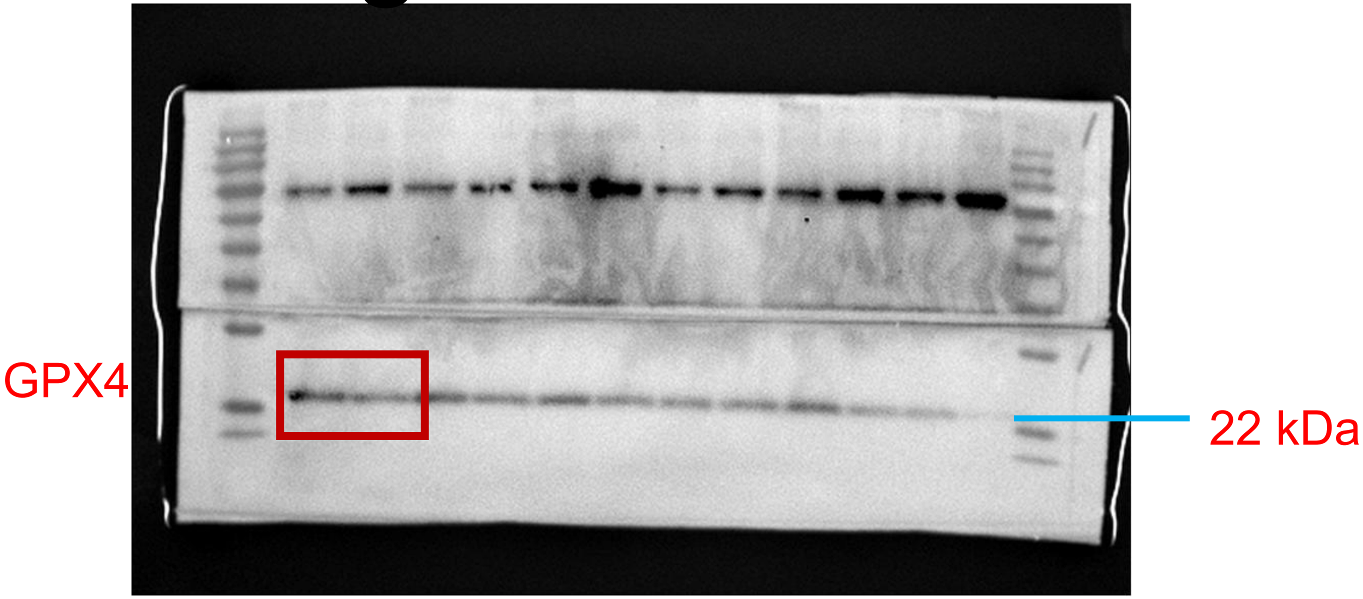

Figure 8B. H460-PDZD8

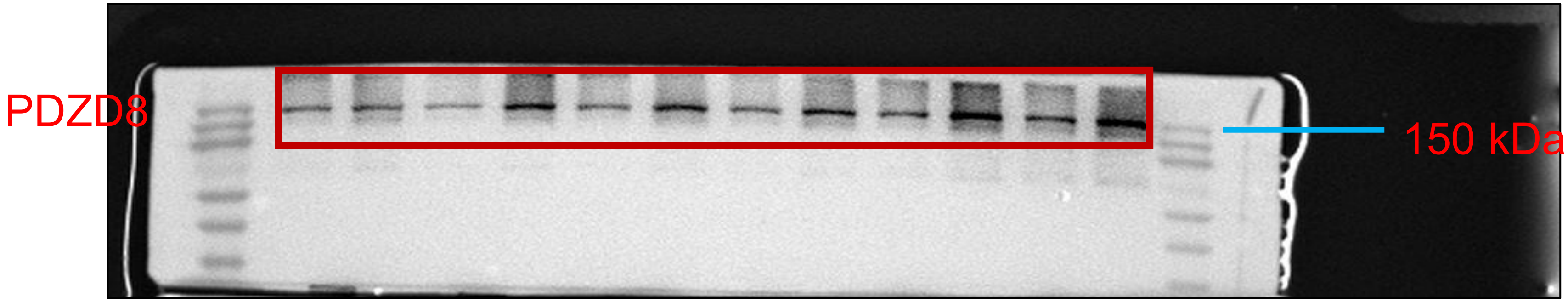

Figure 8B. H460-ACSL4

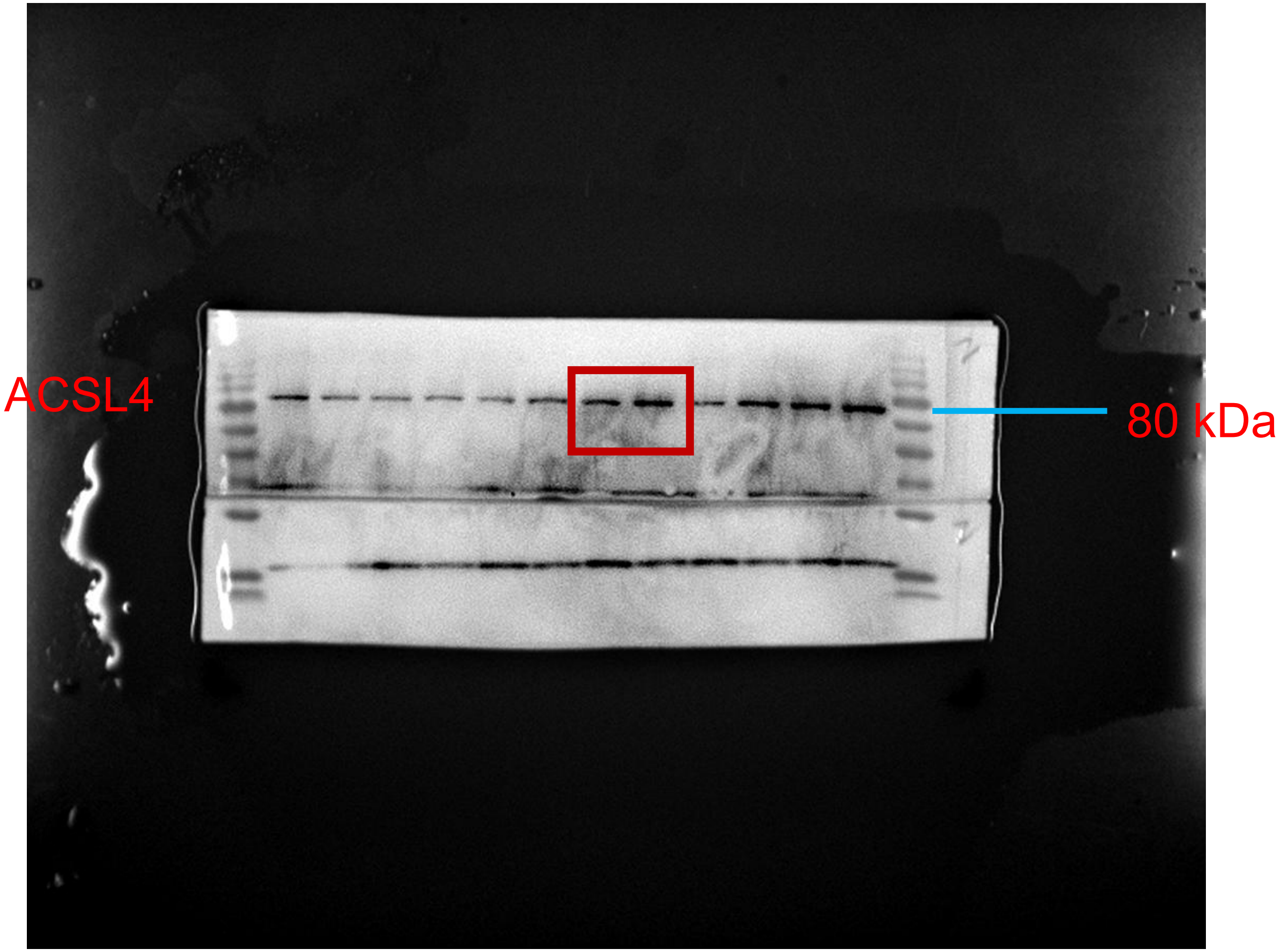

Figure 8B. H460-GPX4

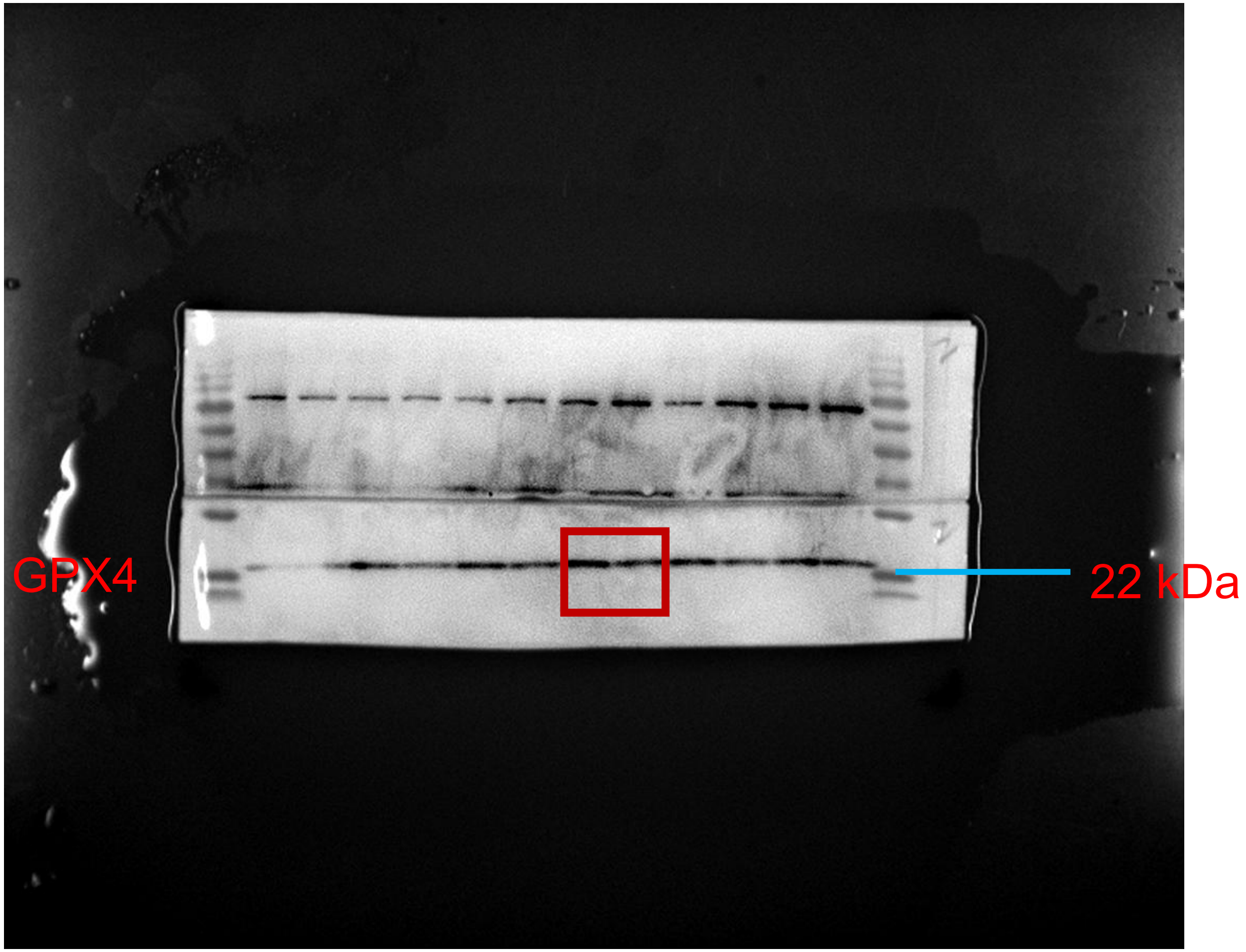

Figure 8B. H460-XCT

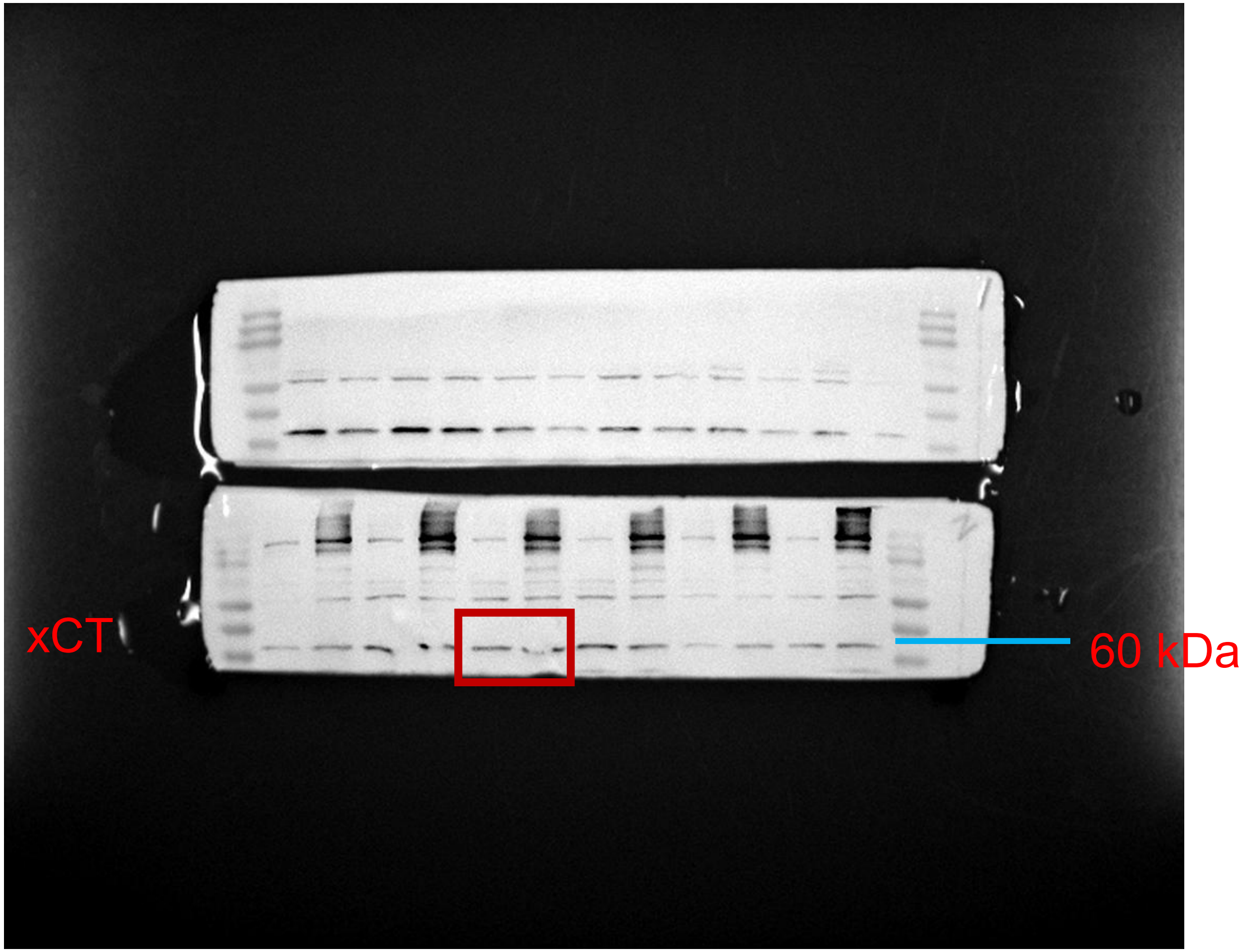

Figure 9

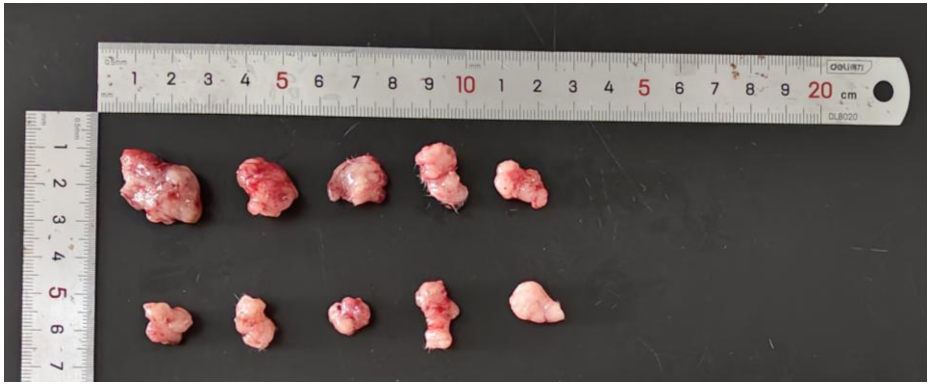

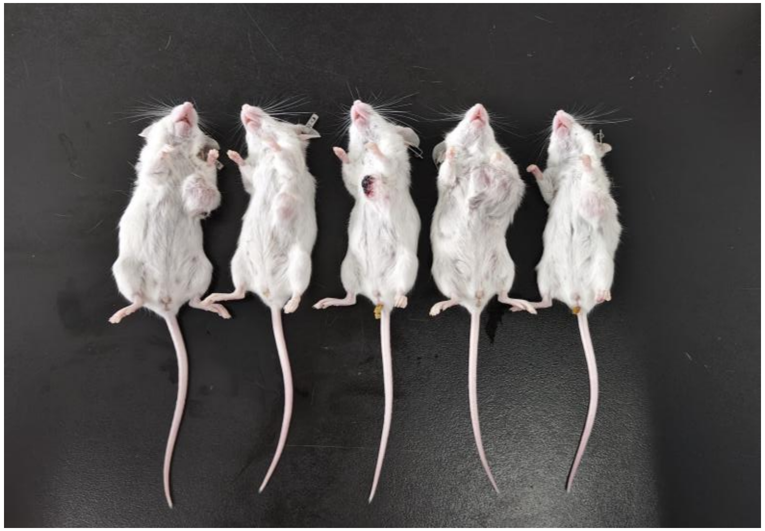
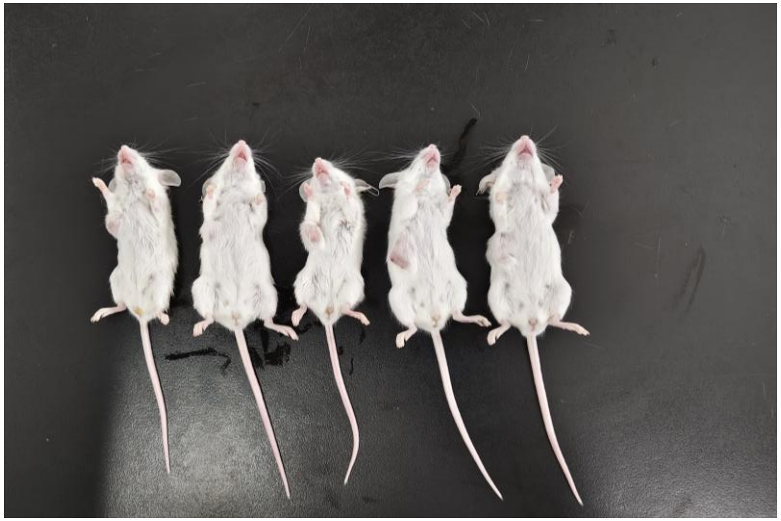

Figure 9 D- β-actin

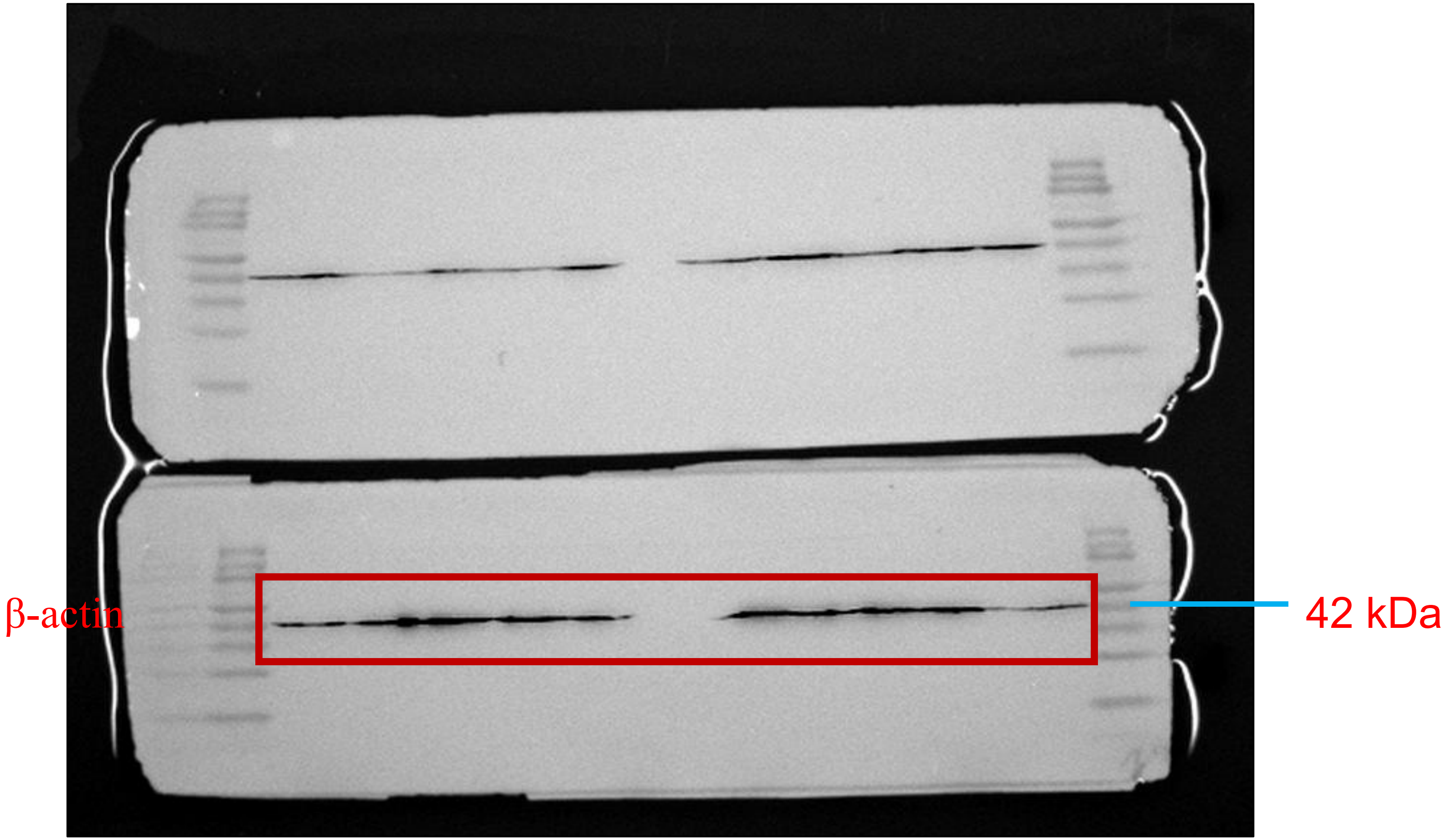

Figure 9 D-ULK1

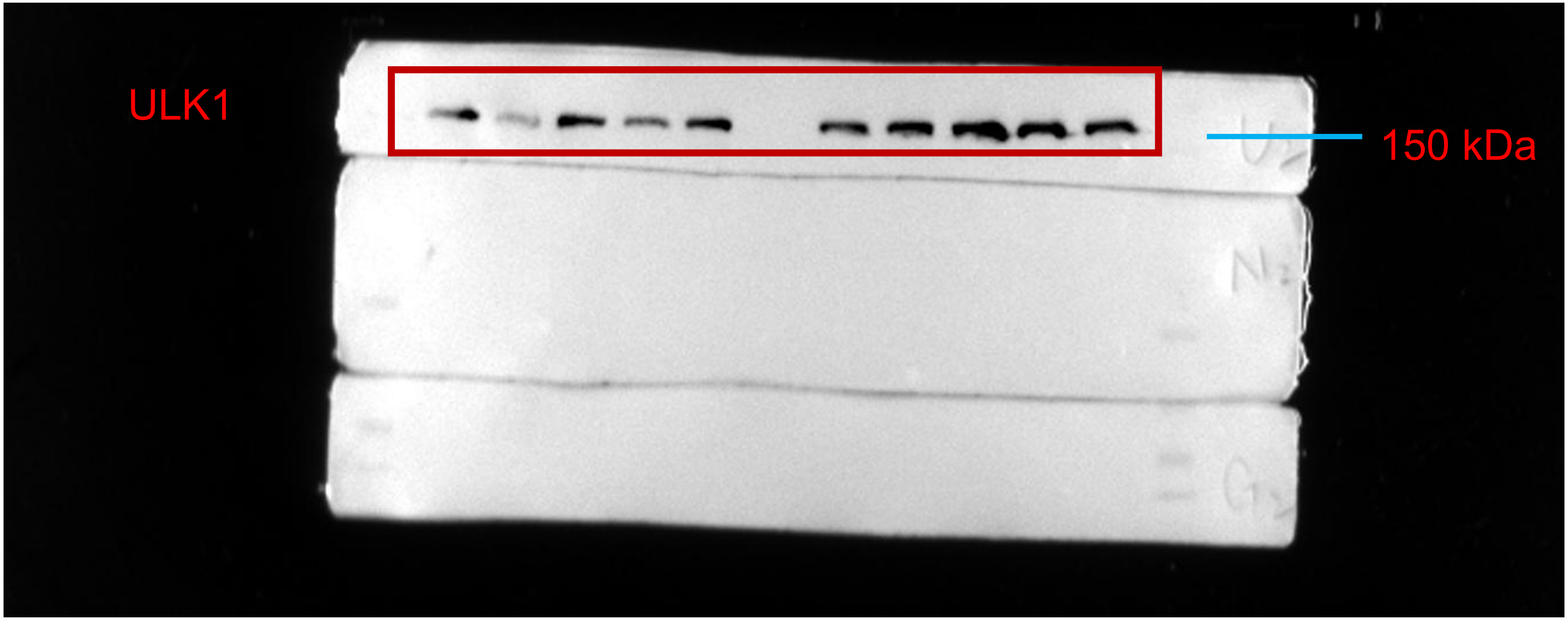

Figure 9 D-NCOA4

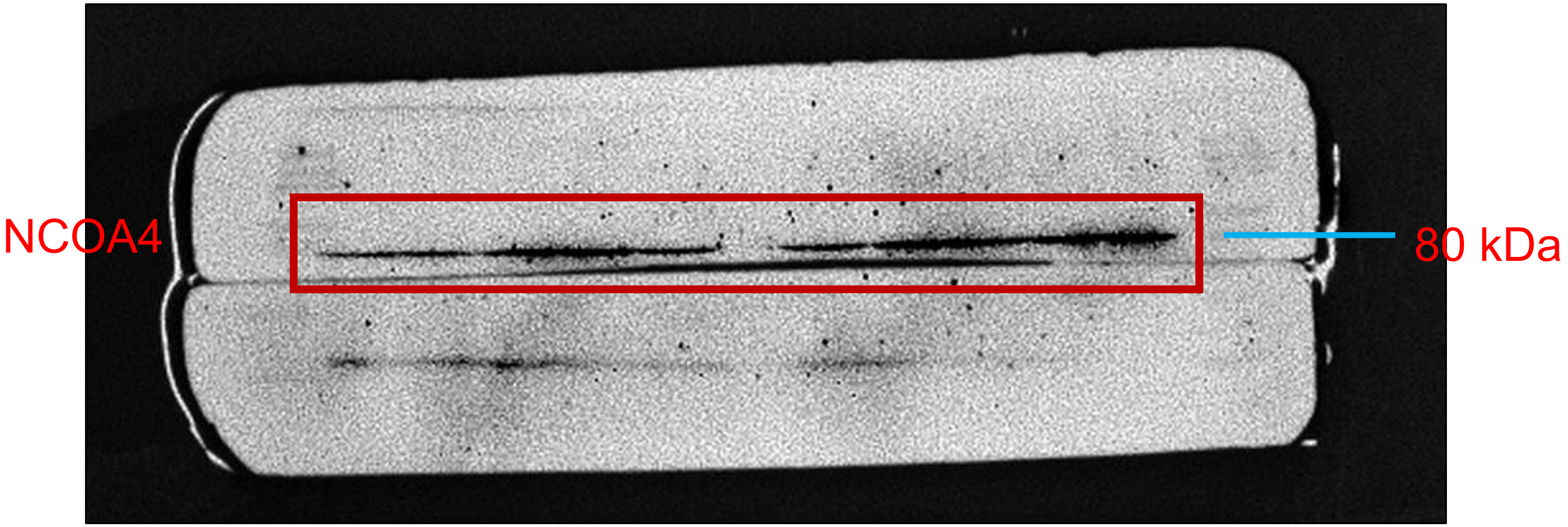

Figure 9 D-BECN1

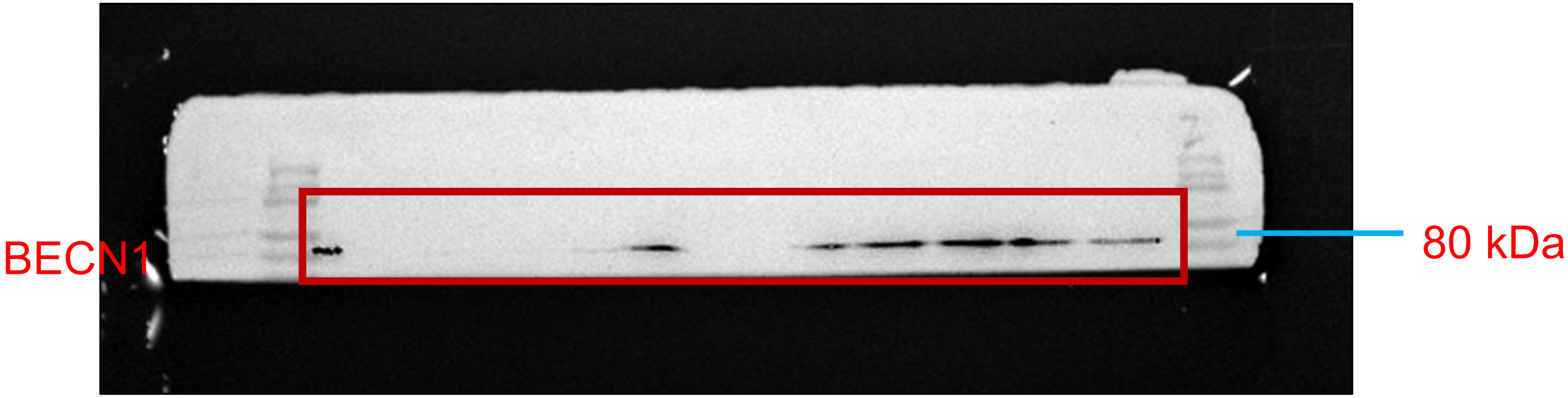

Figure 9 D-LC3B

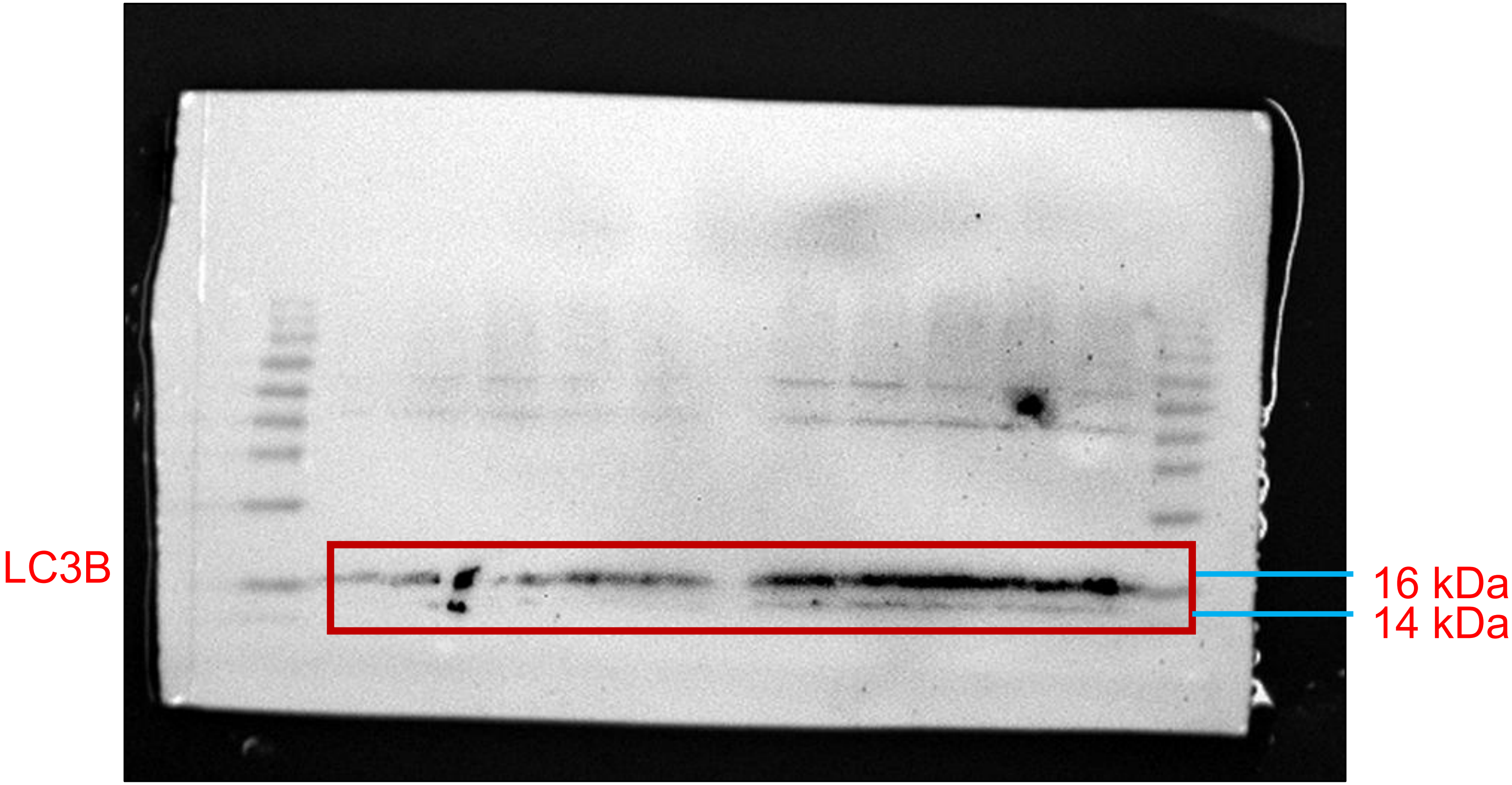

Figure 9 E-xCT

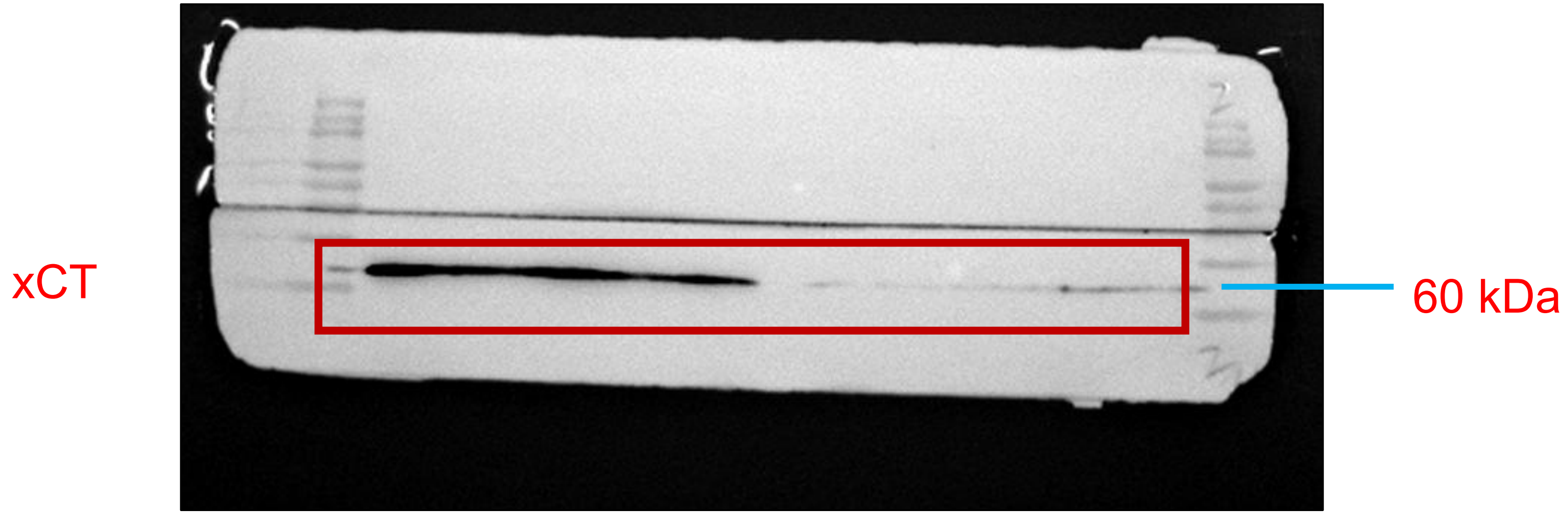

Figure 9 E-GPX4

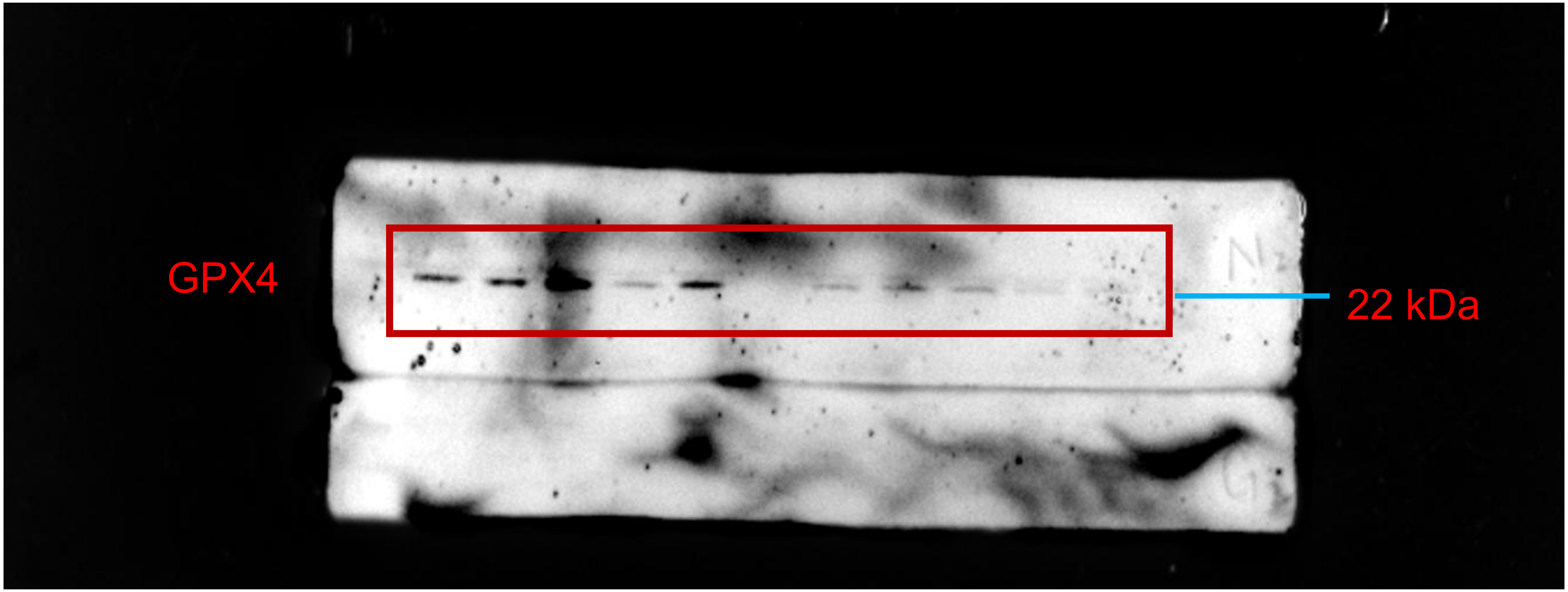

Figure 9 E-ACSL4

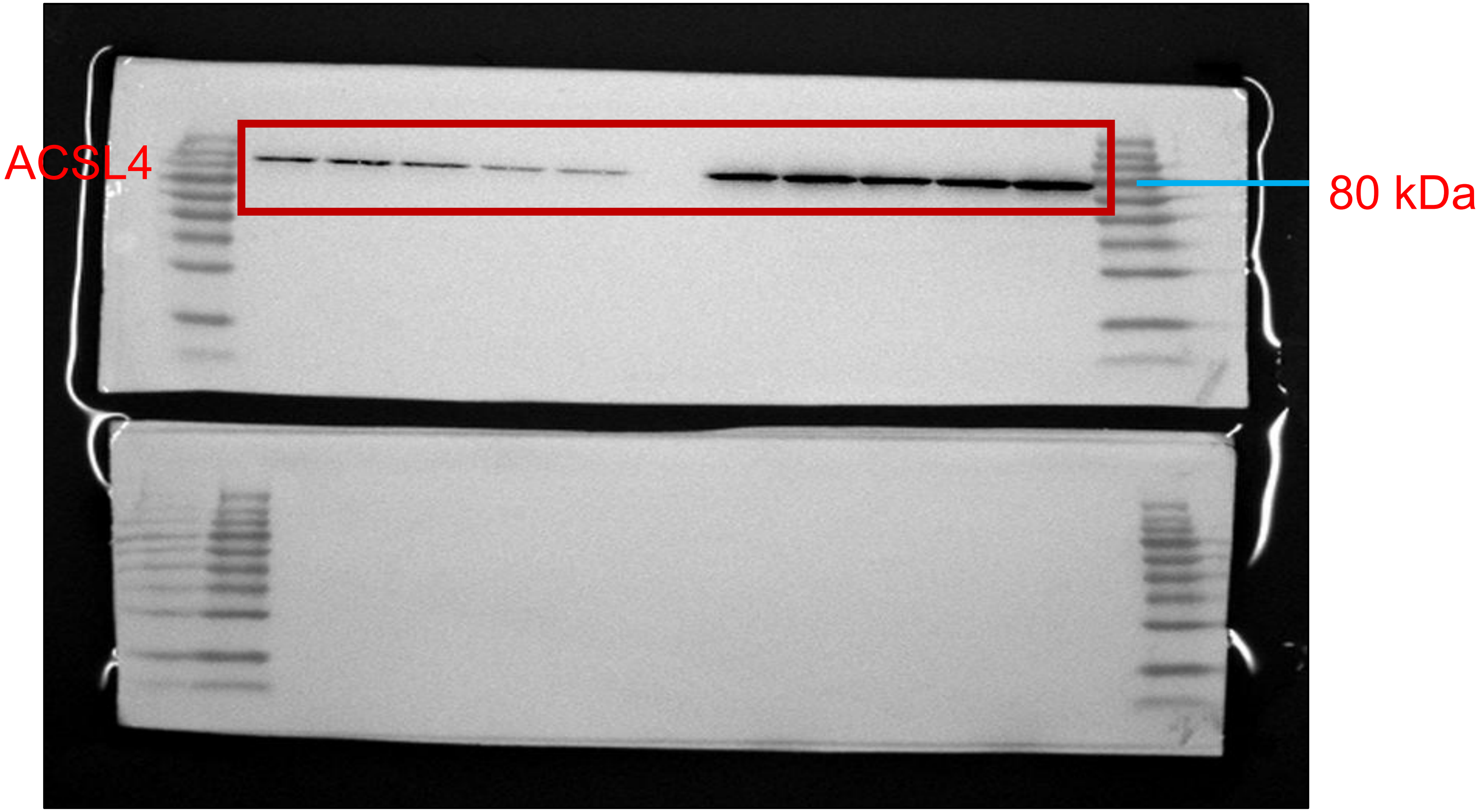

Supplement: Supplementary file 1 [file nutrients-18-01596-s001.zip › File S3.pdf]
